# *Fushenmu* treatment ameliorates RyR2 with related metabolites in a zebrafish model of barium chloride induced arrhythmia

**DOI:** 10.1186/s13020-023-00812-x

**Published:** 2023-08-19

**Authors:** Yan-Ting Zhao, Yan-Ru Liu, Ya-Feng Yan, Zhi-Shu Tang, Jin-Ao Duan, Hui Yang, Zhong-Xing Song, Xue-Lian You, Ming-Geng Wang

**Affiliations:** 1 Shaanxi Collaborative Innovation Center Medicinal Resource Industrialization, Shaanxi University of Chinese Medicine, No. 1 Weiyang Road, Qindu District, Xianyang, 712083 People’s Republic of China; 2 China Academy of Chinese Medical Sciences, No. 16, Nanxiao Street, Dongzhimen, Beijing, 100700 People’s Republic of China; 3 Nanjing University of Chinese Medicine, No. 138 Xianlin Road, Nanjing, 210023 People’s Republic of China; 4 Shandong Buchang Pharmaceutical Co. Ltd, Heze, 250000 Shandong People’s Republic of China

**Keywords:** Fushenmu, Arrhythmia, RyR2, cAMP, Adrenaline, Zebrafish embryo

## Abstract

**Background:**

Fushenmu (*Pini Radix in Poria*, FSM) is a folk parasitic herb that has been mainly used for palpitation and amnesiain in traditional Chinese medicine (TCM). Recently, as an individual herb or a component of formulations, Fushenmu exhibits therapeutic potential for the treatment of cardiac arrhythmias. Yet, how specific targets or pathways of Fushenmu inhibit arrhythmia has not yet been reported.

**Methods:**

Here, based on clinical functional genomics, metabolomics and molecular biologic technologies, a network construction strategy was adopted to identify FSM therapeutic targets and biomarkers that might explore its functions.

**Results:**

In this study, it was found that FSM recovered arrhythmia-associated heart failure in barium chloride (BaCl2) induced arrhythmic zebrafish embryos, as was evidenced by the shortened cardiac sinus venosus—bulbus arteriosus (SV-BA) distance, smaller cardiovascular bleeding areas, and reduced cardiomyocyte apoptosis. Moreover, analysis via ultra-high-performance liquid chromatography–tandem mass spectrometry (UPLC-QTOF-ESI-MS/MS) components identification and network pharmacology prediction showed that 11 main active components of FSM acted on 33 candidate therapeutic targets. Metabolomic analysis also suggested that FSM could rescue 242 abnormal metabolites from arrhythmic zebrafish embryos. Further analysis based on the combination of target prediction and metabolomic results illustrated that FSM down-regulated Ryanodine Receptor 2 (RyR2) expressions, inhibited adrenaline and 3',5'-Cyclic AMP (cAMP) levels in a dose-dependent manner, which was confirmed by metabolites quantification and quantitative reverse transcriptase polymerase chain reaction (qRT-PCR) assay.

**Conclusion:**

In summary, this study revealed that FSM mitigated BaCl2 induced cardiac damage caused by arrhythmia by suppressing RyR2 expressions, decreasing adrenaline and cAMP through the adrenergic signalling pathway.

**Supplementary Information:**

The online version contains supplementary material available at 10.1186/s13020-023-00812-x.

## Background

Arrhythmias, with a prevalence of approximately 3.4%, are a serious health issue afflicting millions of people, and contribute significantly to cardiovascular deaths and morbidity [1–5]. There are various types of arrhythmia, including premature ventricular contractions (PVCs), atrial fibrillation (AF), and tachycardia [6–9]. If left untreated, arrhythmias such as tachycardia or AF can cause heart failure (HF), cardiomyopathy (CM), even sudden cardiac death (SCD), which accounts for 25% of all deaths [10, 11]. Current treatment plans included drug therapy, catheter ablation, or device implantation [12, 13]. However, antiarrhythmic drugs (AADs) have a narrow therapeutic window and potential lethal proarrhythmia side effects. Several clinical trials have revealed that medical plants possess superior efficacy in treating arrhythmia induced syndromes compared to AADs [14–18].

*Fushenmu* (FSM), the pinewood in the inner part of *Poria cum Radix Pini* from *Poria cocos* (Schw.) Wolf (*Pini Radix in Poria*), also known as pine among the Tuckahoe, was first recorded in traditional Chinese medicine classics ‘Bencao Gangmu’. Thanks to its liver-calming and mind-tranquilizing effects, *FSM* has traditionally been used for treating cardiac and hepatic system related insomnia, vexation, and apoplexy [19]. Documents indicate that FSM`s original herbal material *Poria cocos* is effective for reducing hyperlipidaemia and improving chronic heart failure without disturbing body function [20, 21]. Specifically, the main component of FSM, pachymic acid (PA) was recommended to handle reactive oxygen species scavenging, a mechanism involved in oxidative stress-induced cardiovascular disease (CVD) [22, 23]. Tissue distribution reports also showed that the heart may be the primary target of PA’s pharmacological effects [24]. Importantly, PA exhibits promising therapeutic potential on cardiac junctophilin-2 (JP-2) preservation by suppressing miR-24, thereby modulating myocardial contractility in HF [25]. Despite the recent evidence that FSM may has potential effects on CVD, information on how FSM works in CVD, especially in arrhythmia and subsequent implications remains lacking.

Therefore, this study began by characterizing the main active components of FSM via UPLC-QTOF-ESI–MS/MS and screening its possible targets and metabolomes by network pharmacology and non-targeted metabolomics analysis, respectively. After that, a joint pathway analysis strategy was adopted to locate the essential metabolite pathway that contained the therapeutic targets by integrating results of screening. Finally, the potential regulation mechanism of FSM was explored by using targeted metabolomes determination and qRT-PCR assays.

## Methods

### Chemicals and reagents

Metoprolol tartrate was obtained from AstraZeneca Pharmaceutical Co., Ltd. (Jiangsu, China). Barium chloride was obtained from Maoye Chemical Reagent Co., Ltd. (Chongqing, China). LC grade acetonitrile was purchased from Merck (Merck, Darmstadt, Germany). Isopropanol, formic acid and metabolite standards were purchased from Sigma-Aldrich (Sigma-Aldrich, St Louis, MO, USA). Purified water (18.2 MΩ) was prepared from a Mill-Q purification system (Millipore Corp., MA, USA).

### FSM extracts preparation

FSM materials were obtained from Shaanxi Xingshengde Co., Ltd. (Shannxi, China) and identified by Professor Ji-Qing Bai. The voucher specimen was deposited in the Shaanxi Collaborative Innovation Center of Chinese Medicine Resources Industrialization, Shaanxi University of Chinese Medicine (Shaanxi, China).

0.4 g precisely weighted *FSM* powder was ultrasonically extracted with 25 mL methanol for 60 min. Then, the extract was centrifuged (6, 000 rpm, 10 min), 15 mL of supernatant was collected and vacuum lyophilized. For compound identification, the lyophilized extracts were re-dissolved in 5 mL methanol, centrifuged at 12, 000 rpm for 10 min before the supernatant was filtered through a 0.22 μm microporous membrane. For target validation on a zebrafish model, the lyophilized extracts were re-dissolved in zebrafish incubation solution and prepared for 1 mg/mL mother solution that was stored at 4 °C until use.

### Compounds identification

To identify the major compounds from FSM extracts, samples were separated on an ACQUITY UPLC^®^ BEH C_18_ (50 mm × 2.1 mm, 1.7 μm) column via a Waters ACQUITY H-CLASS UPLC/ AB Sciex 5600^+^ QTOF-ESI–MS/MS system (UPLC: Waters, Milford, MA, USA; QTOF-ESI-MS/MS: AB Sciex, Framingham, MA, USA). Mobile phase A was composed of water + 0.1% formic acid while mobile phase B was acetonitrile + 0.1% formic acid. The elution program was set as follows: 0–2 min, 10% B; 2–50 min, 10–100% B; and 50–55 min, 100% B. The flow rate and column temperature were set at 0.3 mL/min and 30 °C, respectively, and the injection volume was 5 µL. Full scan and tandem mass spectrometry experiments were performed via information-dependent data (IDA) acquisition under the positive (5500 V) or negative (− 4500 V) ion mode with the following parameters: declustering potential (DP), 80 V; collision energy (CE), 35 eV; collision energy spread (CES), 15 eV; ion source gas 1 (GS1) and ion source gas 2 (GS2), nitrogen gas, 50 psi; curtain gas (CUR), 35 psi; source temperature (TEM), 550 °C. MS spectra were acquired between 100 ~ 1500 m/z via 200 ms accumulation time, and the 10 most intense precursors per cycle were selected for fragmentation with a dynamic exclusion time of 30 s. Data acquisitions and analyses were performed by using Analyst^®^ TF software (version 1.6, AB Sciex). The MS/MS analysis was performed with Peakview software (version 2.2, AB Sciex). Component identification was conducted with TCM library (version 1.0) in Masterview software (version 1.1.0, AB Sciex).

### Selection of key targets for FSM therapy

#### Dataset of FSM therapeutic targets

To explore targets related to FSM therapy, data on main compounds collected from the identification process and databases were integrated to form the dataset of ‘FSM-compounds’ dataset that was then imported into the BATMAN-TCM (a Bioinformatics Analysis Tool for Molecular mechANism of Traditional Chinese Medicine, http://bionet.ncpsb.org/batman-tcm/) and ETCM databases (The Encyclopedia of Traditional Chinese Medicine, http://www.tcmip.cn/ETCM/) to obtain the ‘FSM-compounds-targets’ set with a cut off score ≥ 0.45.

#### Dataset of arrhythmia targets

Atrial fibrillation (AF) is the most common cardiac arrhythmia, which is characterized by an unorganized atrial rhythm [26]. To screen the arrhythmia targets, a clinical arrhythmia cohort with typical AF response was analyzed. The arrhythmia dataset (GSE41177) was collected from gene expression omnibus (GEO) databases (http://www.ncbi.nlm.nih.gov/geo/) [27]. Raw dataset included 16 AF patients and 3 sinus rhythm controls from pulmonary veins, the surrounding left atrial junction (LA-PV junction) and left atrial appendage (LAA) samples. Differentially expressed genes (DEGs) between the AF and control groups were identified using the Limma R package (version 3.6.3) [28]. Genes with |Log_2_-fold change (FC) |> 1 and adjusted *p*-value (adj. *p*) < 0.05 were considered DEGs. Gene set enrichment analysis (GSEA) and Gene Ontology (GO) analysis were performed on the DEGs using the clusterProfiler package (version 3.14.3) [29]. Gene set collections were from MSigDB (http://software.broadinstitute.org/gsea/msigdb). Furthermore, GO and Kyoto Encyclopedia of Genes and Genomes (KEGG) analysis were enriched with 1, 000 gene set permutations, a minimum gene set size of 10, and a maximum of 500. The Benjamini–Hochberg method was used to calculate false discovery rate-adjusted P-values. Then, arrhythmia-related targets were obtained from GeneCards database (https://www.genecards.org/). By these means, the arrhythmia target set was obtained via a combination of both GEO DEGs and the GeneCards database targets.

#### ‘FSM- Arrhythmia’ interaction target dataset

The common targets of the FSM component-target dataset and arrhythmia-target dataset were collected by VENNY 2.1.0 platform (https://bioinfogp.cnb.csic.es/tools/venny/), whose protein–protein relationship (*Homo sapiens*) was analyzed with STRING (https://string-db.org/) and the targets with a degree greater than the median score were screened into the ‘FSM—arrhythmia’ interaction targets set.

#### ‘FSM—compounds—arrhythmia—targets’ network

To predict the function and pathway of FSM therapy for arrhythmia, the ‘*FSM—compounds—arrhythmia—targets’ network* was constructed based on the ‘FSM-compound’ set and ‘FSM—arrhythmia’ interaction target set via Cytoscape software (version 3.8.2). The compounds and targets with a node degree greater than the median score were classified as the key elements. Then, the gene function and pathway of the key targets were analyzed by GO and KEGG enrichment using the R package clusterProfiler (version 3.6.3)and org.Hs.eg.db (version 3.10.0) [29].

### Selection of FSM therapeutic biomarkers

#### Arrhythmia model for zebrafish embryos

After the key targets were chosen, a BaCl_2_ induced arrhythmia model on zebrafish embryos was adopted to evaluate FSM effect and to explore its mechanism. Wild-type AB-strain zebrafish embryos were obtained from China Zebrafish Resource Center (production batch: 20,200,609, Wuhan, China), and kept following experimental protocols [30]. Embryos (72 hpf) were randomized and divided into six groups and placed on 48-well cell culture plates (eighty embryos per group, five embryos per well): control, model (BaCl_2_ 2.1 μg/mL), positive control (model + metoprolol 38.40 μg/mL), FSM extract treatment groups (FSM-H: model + FSM extract 47.70 μg/mL, FSM-M: model + FSM extract 23.85 μg/mL, FSM-L: model + FSM extract 15.90 μg/mL). The dose selections were based on the FSM extract LC_50_ value of 0.48 mg/mL in zebrafish embryos. After 24 h incubation with sterile saline solution of BaCl_2_, metoprolol, or FSM extract, the morphological observation, metabolomic analysis and targets verification were performed. All animal handling and experimental conditions were approved by the Laboratory Animal Care and Use Committee of the Shaanxi University of Chinese Medicine.

#### Morphological observation and sinus venosus—bulbus arteriosus (SV-BA) distance measurement

The SV-BA length is an important indicator of cardiovascular health of zebrafish. After 24 h of treatment, zebrafish embryos (ten embryos per group) were washed with phosphate-buffered saline (PBS, Servicebio, Wuhan, China) before being placed on a slide with 3% methylcellulose (Innochem, Beijing, China). The position of the zebrafish was adjusted so that the two sides of the eye and body coincided as much as possible. A BX51 electron fluorescence microscope with an Olympus DD71 camera (Olympus, Tokyo, Japan) was used to observe the morphology of hearts of the zebrafish at 20 × magnification. Then, the pericardial congestion area and the SV-BA straight line distance were measured by using ImageJ software (version 1.8.0, NIH, USA) [31–33]. Parallel experiments were repeated three times.

#### Observation of myocardial apoptosis

To assess the apoptosis in cardiomyocytes, the embryos were stained with 2.5 μg/mL acridine orange (AO) solution for 30 min (Alfa Aesar, Beijing, China). Then, embryos were washed three times in PBS. In acridine orange-stained cells, the normal cells will fluoresce homogeneously bright green, whereas the apoptotic cells will fluoresce roughly yellow or orange. Stained embryos were imaged using a fluorescence microscope (Olympus ABX51, Olympus Life Science, Japan) with blue excitation (460 nm ~ 490 nm) and green emission (510 ~ 530 nm) filters. The mean density was determined in control and treated embryos. The density of the fluorescence was measured using ImageJ software [34, 35].

#### Metabolomic analysis for selection of FSM therapeutic markers

After dosing and incubation of 24 h, embryos from each group (30 embryos per group) were transferred into a 2 mL centrifuge tube and washed repeatedly with PBS. Subsequently, 1 mL of 80% methanol aqueous solution (pre-frozen at −80 °C for 30 min) was added into the centrifuge tube before the sample was homogenized and centrifuged at 12,000 rpm for 10 min until the supernatant was collected and lyophilized (vacuum lyophilization). For metabolomic analysis, the freeze-dried sample was dissolved in 250 μL of 50% aqueous solution of acetonitrile.

Untargeted metabolite profiling was acquired by using Analyst^®^ TF software (version 1.6, AB Sciex) on a Waters H-class UPLC coupled with a high-resolutionAB SCIEX 5600^+^ QTOF-ESI–MS/MS instrument. Samples were separated on an ACQUITY UPLC^®^ HSS T3 (100 mm × 2.1 mm, 1.8 μm) column. The gradient elution was operated at a 0.3 mL/min flow rate composed of Milli—Q water (A) and acetonitrile (B) with 0.05% formic acid and 2 mM ammonium acetate. The elution programs were as follows: 0 ~ 23 min: 1–60% B; 23 ~ 24 min: 60–98% B; 24 ~ 27 min: 98% B; 27 ~ 27.1 min: 98–1% B; 27.1–33 min: 1% B. The flow rate and column temperature were set at 0.3 mL/min and 30 °C, respectively, and the injection volume was 5 µL. Full scan and tandem mass spectrometry experiments were performed via information-dependent data (IDA) acquisition under the positive (5500 V) or negative (− 4500 V) ion mode with the following parameters: DP, 40 V; CE, 35 eV; CES, 15 eV; GS1 and ion GS2, 50 psi; CUR, 35 psi; TEM, 550 °C. MS spectra were acquired between 70 and 1200 m/z via 200 ms accumulation time, and the 12 most intense precursors per cycle were selected for fragmentation with a dynamic exclusion time of 30 s. Metabolite identification was carried out by matching accurate mass and experimental MS/MS fragmentation patterns against Accurate mass metabolite HR-MS/MS library (version 2.0) in Peakview software (version 2.2, AB SCIEX), Masterview software (version 1.1, AB SCIEX) [36]. The area matrix of metabolites was calculated by Multiquant database (version 3.0.2, AB SCIEX). Then, data on the area matrix of grouping metabolites was subjected to dimensionality reduction techniques and significance analysis on the MetaboAnalyst (https://www.metaboanalyst.ca/) platform. The metabolites in control and model groups with a *p*-value of less than 0.05, fold change (FC) above 2.0, and variable importance in projection (VIP) value larger than 1 were regarded as statistically significant biomarkers via student *t* test, principal components analysis (PCA), patterns-partial least squares discriminant analysis (PLS-DA). The biomarkers in the six groups with significant restored trends (*p* < 0.05) were selected as FSM therapeutic markers via one-way analysis of variance (ANOVA) before being mapped to pathway maps for visualization analysis by ‘Pathway analysis’ on MetaboAnalyst. Pathways with adjusted p-value (Holm p) < 0.05 and Impact > 0.1 were defined as significantly enriched pathways.

### Integration of FSM therapeutic candidate targets and metabolomics data

To find out more about the functional relationship between FSM therapeutic targets and changes at the level of metabolites, a joint pathway analysis was conducted on MetaboAnalyst (https://www.metaboanalyst.ca/) for therapeutic pathway location. To this end, all highly enriched targets and significantly perturbed metabolites were inputted before those over-enriched in KEGG were searched for. All pathway (integrated) enrichment analysis was conducted using ‘Hypergeometric test’. The topology measure was set at ‘Degree Centrality’, and integration method was set at ‘combine p values (pathway-level)’. Pathways with Holm p < 0.05 and Impact > 0.2were defined as significantly enriched pathways. Then, the FSM therapeutic key targets and markers were screened from the identified pathway.

### FSM therapeutic mechanism confirmation

#### Determination of FSM therapeutic metabolomic markers

After integration analysis, quantitative analysis of the FSM therapeutic markers was conducted on an Agilent 1290 Infinity UPLC- AB Sciex Q-TRAP 4500 ESI–MS/MS instrument (UPLC: Agilent technologies, MS: AB Sciex). Samples were separated on an ACQUITY UPLC^®^ HSS T3 (100 mm × 2.1 mm, 1.8 μm) column composed of Milli—Q water (A) and acetonitrile (B) with 0.2% formic acid. The gradient elution was operated at a 0.3 mL/min flow rate. The elution programs were as follows: 0 ~ 23 min: 1–60% B; 23 ~ 24 min: 60–98% B; 24 ~ 27 min: 98% B; 27 ~ 27.1 min: 98–1% B; 27.1—33 min: 1% B. The flow rate and column temperature were set at 0.3 mL/min and 30 °C, respectively, and the injection volume was 2 µL. Positive-ESI–MS analysis was performed in a multiple reaction monitoring (MRM) mode. The ionization parameters were as follows: GS1 and ion GS2, 50 psi; CUR, 40 psi; TEM, 500 °C. FSM therapeutic markers were analyzed via optimized parameters including DP, CE and collision cell exit potential (CXP) (detailed optimized MRM parameters of therapeutic markers are shown in Additional file 7: Table S22. The total ion chromatograms of the markers are shown in Additional file 7: Figure S4). The FSM therapeutic metabolomic markers were determined according to established methodologies (detailed quantification methodology of therapeutic metabolite markers are shown in Additional file 7: Tables S23–S25).

#### Verification of FSM therapeutic key targets

For verification of key therapeutic targets, qRT-PCR analysis was performed. At the end of the administration, zebrafish embryos (thirty embryos per group) were homogenized in TRIzol reagent (Servicebio Technology Co., Ltd., Wuhan, China) to extract total RNA. Following extracted protocols of the Servicebio^®^ RT First Strand cDNA Synthesis Kit (G3330, Servicebio, Wuhan, China). qRT-PCR assays were conducted on an ABI 7900HT Fast Real-Time PCR system (Bio-Rad Bole Gradient, California, America). The oligonucleotides for PCR were as follows: zebrafish RyR2 (ref. XM_021480192.1, 156 bp, 60 °C, Servicebio): 5'-AAGACGCAACAGGTGAGGCA-3' (forward) and 5'-CGTGTAAACTGCCGTTCCCATA-3' (reverse). The RyR2 mRNAs level was normalized to the GAPDH mRNA and determined using the 2^−ΔΔCt^ method [37].

### Statistical analysis

All experiments were performed in triplicate. All statistical analyses were conducted with GraphPad Prism (Version 6.0) and SPSS (Version 18.0) software. Data are presented as the mean ± s.d. or mean ± s.e.m. one-way ANOVA was used to analyze multiple comparisons, followed by least significant difference (LSD) multiple-range tests. In all statistical comparisons, p < 0.05 was considered a significant difference.

## Results

### FSM therapeutic target set

**Fig. 1:**
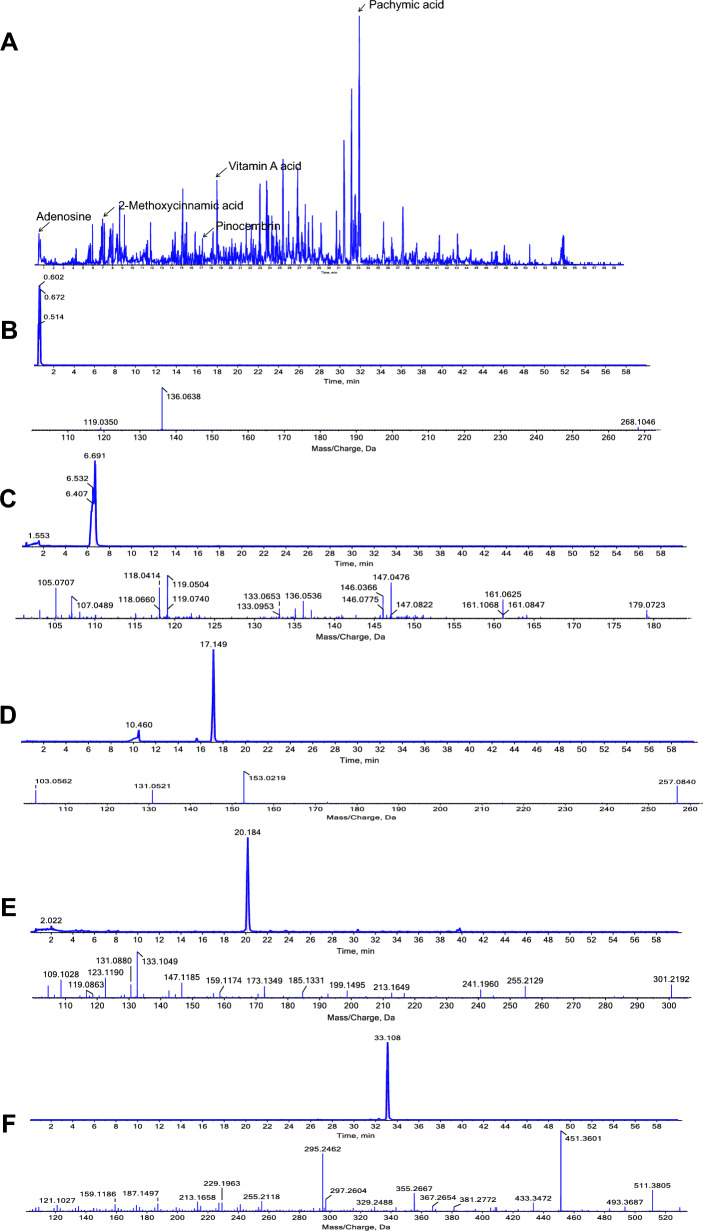
UPLC-TOF–MS results in positive ion mode. **A** TIC in positive ion mode. The MS/MS spectra of the 5 compounds identified in Fushenmu, **B** adenosine (the retention time was 0.602 min, 268.1046 m/z → 136.0638 m/z), **C** 2-methoxycinnamic acid (the retention time was 6.691 min, 179.0723 m/z → 146.0366 m/z), **D** pinocembrin (the retention time was 17.149 min, 257.0840 m/z → 153.0219 m/z), **E** vitamin A acid (the retention time was 20.184 min,301.2192 m/z → 133.1049 m/z), **F** pachymic acid (the retention time was 33.108 min, 529 m/z → 511 m/z)

In order to extract key target information from the ‘FSM—compounds—arrhythmia—targets’ network, we first organized the targets set corresponding to the FSM mainly compounds. Based on data from literature, tentative identification of the main compounds from FSM was performed by comparing the QTOF accurate m/z values with the main MS/MS fragments. Then, a total of 17 compounds were obtained: pachymic acid, adenosine, vitamin A acid, pinocembrin, 2-methoxycinnamic acid, choline, turanose, ergotamine, palmitic acid, lauric acid, caprylic acid, tumulosic acid, dehydroeburicoic acid, nardosinone, lauric aldehyde, ergosterol, L-uridine. Among them, 5 of them were confirmed by standards via QTOF-ESI–MS/MS and were further quanlitfied by ESI–MS/MS via multiple reaction monitoring (MRM) mode. Representative total ion chromatograms (TIC) of QTOF-ESI–MS/MS for the 5 compounds and the extracted ion chromatograms of each identified compound are showed in Fig. 1 (detailed MS identification results are shown in Additional file 1: Table S1-1). These 5 compounds with retention time were: adenosine (0.62 min, Fig. 1B), 2-methoxycinnamic acid (6.69 min, Fig. 1C), pinocembrin (17.15 min, Fig. 1D), vitamin A acid (20.18 min, Fig. 1E), and pachymic acid (33.11 min, Fig. 1F). Moreover, the simultaneous quantification contents for the 5 compounds were: pachymic acid (0.811 mg/g), adenosine (0.0004 mg/g), vitamin A acid (0.017 mg/g), pinocembrin (0.036 mg/g), and 2-methoxycinnamic acid (0.018 mg/g) (Fig. 1, detailed MS quantification results are shown in Additional file 1: Figure S1, Tables S1-2 to S1-5,). To form a compound-associated target set, a total of 990 FSM compound related targets were obtained from BATMAN-TCM and ETCM databases (detailed ‘compound-target’ set is shown in Additional file 1: Table S2).

### Dataset for arrhythmia associated targets

**Fig. 2:**
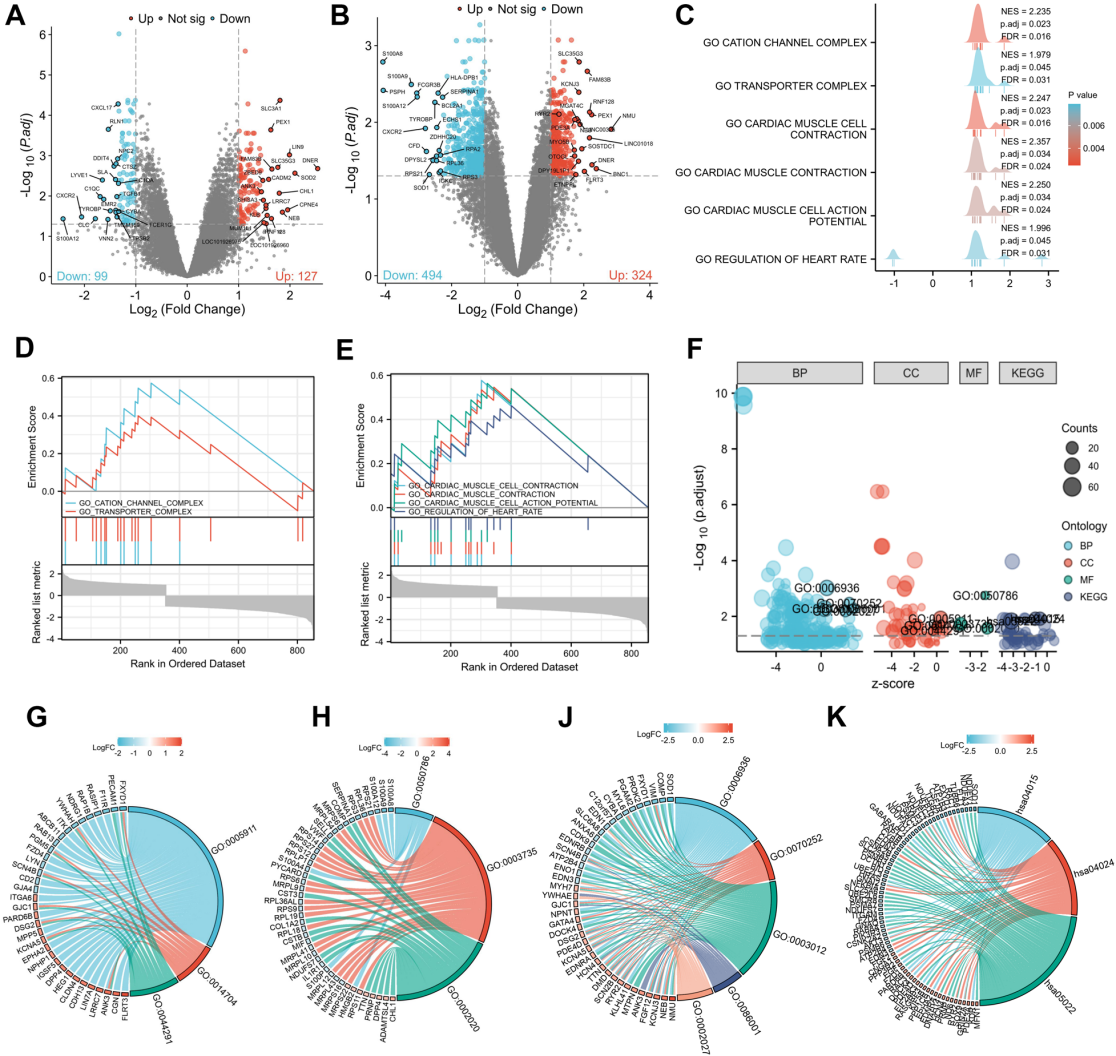
Analysis of differentially expressed genes between AF patients and control ones using GSEA, GO and KEGG analysis. **A** Genes differentially expressed between AF patients and controls of the LA-PV junction samples. **B** Genes differentially expressed between AF patients and controls of the LAA samples. Top20 significant target genes were labeled. Blue nodes represent down-regulation, red nodes represent up-regulation, and grey nodes represent no significant difference from controls (|Log_2_-fold change (FC) |> 1 and adjusted *p* value < 0.05). **C** Ridgeline plot across the GSEA C5 ontology gene analysis colored according to p adjust value (Top). X axis represents density of top genes` Log_2_ FC in enriched terms, y axis represents significantly enriched terms (gene sets). The values of false discovery rate (FDR), *p* adjust and normalized enrichment scores (NES) are depicted on the right side of the plot. **D** Enrichment analysis of the gene set ‘cation channel complex’ and ‘transporter complex’ in cellular component (CC) categories of GSEA C5 ontology gene sets. **E** Enrichment analysis of the gene set ‘muscle contraction’, ‘actin-mediated cell contraction’, ‘muscle system process’, ‘cardiac muscle cell action potential’, and ‘regulation of heart rate’ in biological process (BP) categories of GSEA C5 ontology gene sets. Genes were ranked by log_2_ FC. **F** Bubble plot for gene ontology analysis of significant regulated genes. Genes were enriched by log_2_ FC. **G** Chord plot shows the three most enriched GO CC terms and enriched genes for each term. **H** Chord plot shows the three most enriched GO MF terms and enriched genes for each term. **J** Chord plot shows the five most enriched GO MF terms and enriched genes for each term. **K** Chord plot shows the five most enriched KEGG terms and enriched genes for each term

A total of 226 DEGs in LA-PV junction and 818 DEGs in LAA samples were obtained from the GSE41177 dataset (|Log_2_FC |> 1 and adj.* p* < 0.05, Fig. 2A, B, detailed arrhythmia associated targets in LA-PV junction set are shown in Additional file 2: Table S3). A total of 932 DEGs were combined from two samples (Additional file 2: Table S3). To identify the genomic expression of DEGst, GSEA was conducted with reference to C5 ontology gene sets and gene ontology (GO) analysis (Fig. 2C, F, detailed GSEA C5 ontology gene analysis results are shown in Additional file 2: Table S4, detailed GO analysis in Additional file 2: Table S5). C5 ontology gene pathway analysis indicated ‘cation channel complex’ and “transporter complex” as top enriched cellular location. In addition, pathway analysis identified ‘cardiac muscle cell contraction’, ‘cardiac muscle contraction’, ‘cardiac muscle cell action potential’ and ‘regulation of heart rate’ among the most-regulated pathways. Consistent with this, the GSEA on the ontology gene set database revealed significant regulation of the gene set in arrhythmia patients (Fig. 2C, Additional file 2: Table S4). Cellular component (CC) categories of GSEA indicated a significant up-regulation of gene set from the terms of cation channel complex (*adj.p* = 0.016) and transporter complex (*adj.p* = 0.033) in arrhythmia patients (Fig. 2D). While GO CC categories were enriched in the cell–cell junction (*adj.p* = 0.012), intercalated disc (*adj.p* = 0.021), and cell–cell contact zone (*adj.p* = 0.032) (Fig. 2G). GO molecular function (MF) categories were enriched in RAGE receptor binding (*adj.p* = 0.002), structural constituent of ribosome (*adj.p* = 0.019), and protease binding (*adj.p* = 0.027) (Fig. 2H). GSEA biological process (BP) categories were enriched in cardiac muscle cell contraction (*adj.p* = 0.021), cardiac muscle contraction (*adj.p* = 0.034), cardiac muscle cell action potential (*adj.p* = 0.034), and regulation of heart rate (*adj.p* = 0.044) (Fig. 2E), while GO BP categories were enriched in muscle contraction (*adj.p* = 0.001), actin-mediated cell contraction (*adj.p* = 0.003), muscle system processes (*adj.p* = 0.005), cardiac muscle cell action potential (*adj.p* = 0.005), and regulation of heart rate (*adj.p* = 0.007) (Fig. 2J). Then, GO KEGG documented signaling switch between the Rap1 signaling pathway (*adj.p* = 0.012), cAMP signaling pathway (*adj.p* = 0.012), and pathways of neurodegeneration (*adj.p* = 0.015) (Fig. 2K). Among them, the chord plots of GO analysis that depicted RyR2, FN1, FGF2, PDE4D, KCNA5, and SCN4B in the gene signature were recognized as key genes (Fig. 2G–K). Furthermore, a total of 708 arrhythmia related targets were extracted from GeneCards databases (Additional file 2: Table S3). Finally, an arrhythmia associated targets set that consisted of 1597 targets was established by a combination of both GEO DEGs and the database targets (Additional file 2: Table S3).

### ‘FSM—compounds—arrhythmia—targets’ network construction

**Fig. 3:**
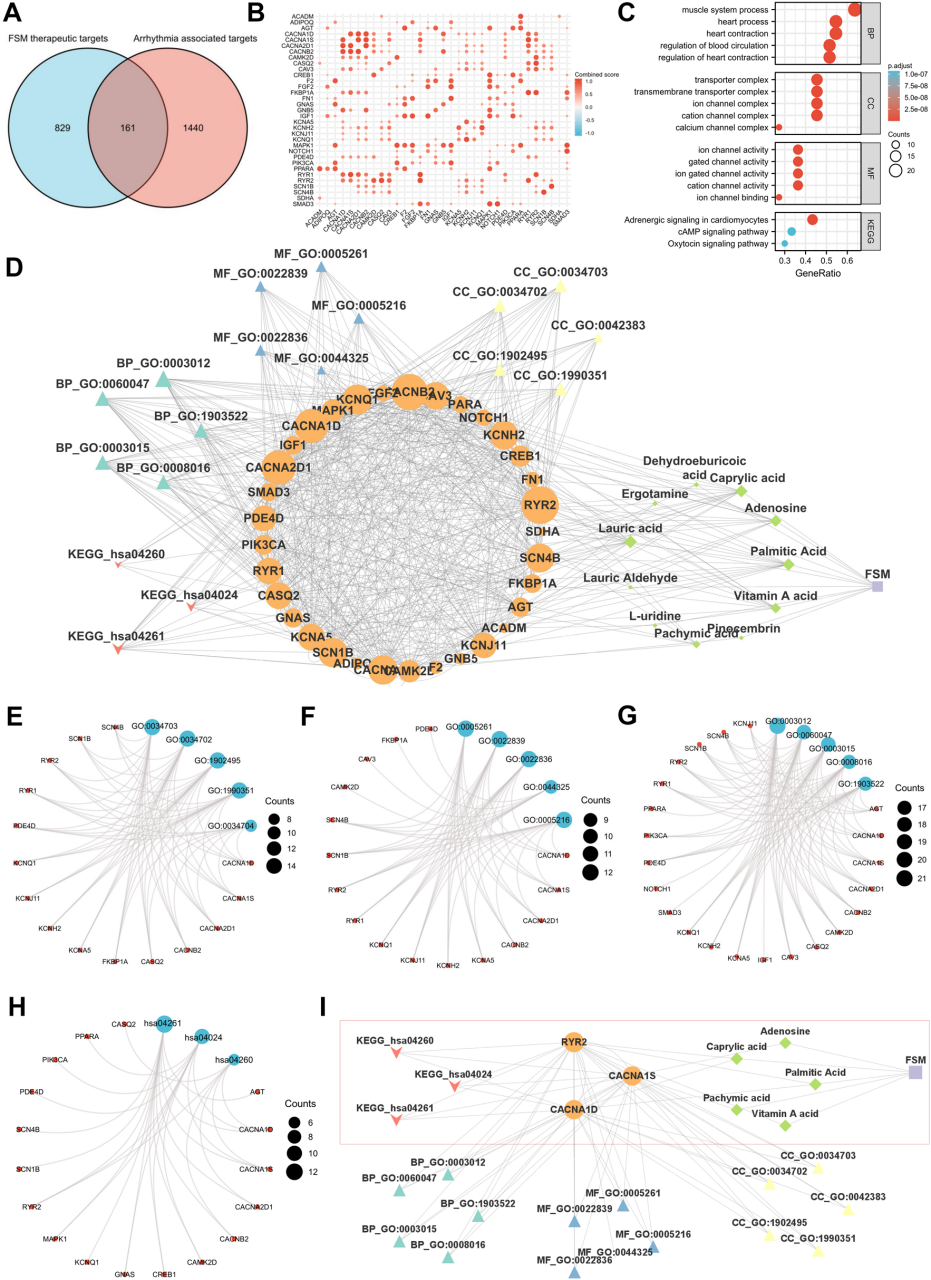
‘FSM—compounds—arrhythmia—targets’ network construction. **A** Venn diagram for intersections of target genes from the FSM therapeutic targets dataset and arrhythmia associated targets set. **B** Heatmap depicting the correlation of combined score among the 33 candidate targets. **C** Bubble plot for GO and KEGG enrichment analysis of 33 candidate targets in the network. The bubble plot showed the top 5 enriched GO terms and 3 KEGG pathways involved in arrhythmia. **D**‘FSM—compounds—arrhythmia- targets’ network. Different shapes represent different modules. Rectangle: FSM, diamond: compounds, ellipse: candidate targets, triangle: GO terms, arrow: KEGG pathways. **E** The cnetplot for top 5 GO CC terms of candidate genes. **F** The cnetplot for top 5 GO MF terms of candidate genes. **G** The cnetplot for top 5 GO BP terms of candidate genes. **H** The cnetplot for top 3 KEGG pathways of candidate genes. **I** Crosstalk for prominent targets and their related compounds from enriched KEGG pathways from ‘FSM—compounds—arrhythmia—targets’ network. Diagrams were constructed using ggplot2 package (version 3.3.3) R software

By using the intersection tool, 990 targets from FSM therapeutic target set and 1597 targets from the arrhythmia-target set were analyzed, and a total of 161 common targets were obtained (Fig. 3A, detailed common targets set are shown in Additional file 3: Table S6). Then, connections between these targets were analyzed by STRING and 33 candidate targets were screened according to the connected degree value (*cut off* by median) (Fig. 3B, detailed degree and interaction results are shown in Additional file 3: Tables S7, S8, the ‘33 candidate targets-FSM compounds’ datasets are shown in Additional file 3: Table S9). To analyze the landscape of interactions between the *FSM* and the arrhythmia in depth, we characterized a ‘*FSM*—compounds—arrhythmia- targets’ network comprising 11 of the 17 main compounds, 33 ‘*FSM*-arrhythmia’ candidate targets, 15 candidate targets enriched GO terms and 3 KEGG pathways (Fig. 3C, D, the results of GO analysis and network analysis of the 33 candidate targets are shown in Additional file 3: Tables S10, S11). It was found that ‘FSM-arrhythmia’ GO and KEGG enriched terms of 33 candidate targets shared significant similarity with those in clinical reports in terms of interactions. CC categories indicating ‘FSM-arrhythmia’ candidate targets were mostly located in the cation channel complex, ion channel complex, transmembrane transporter complex, transporter complex, and calcium channel complex related to 15 candidate targets (*adj.p* < 0.001) (Fig. 3E, Additional file 3: Table S10). MF of 16 of these candidate targets focused on cation channel activity, ion gated channel activity, and gated channel activity (*adj.p* < 0.001) (Fig. 3F, Additional file 3: Table S10). Meanwhile, 22 candidate targets were involved in 5 of the BP terms among cation channel activity, ion gated channel activity, gated channel activity, ion channel binding, and ion channel activity (*adj.p* < 0.001) (Fig. 3G, Additional file 3: Table S10). KEGG was mainly enriched in three pathways including 17 candidate targets. Interestingly, the enriched pathways and GEO clinical reports had similar enriched outcomes (adrenergic signaling in cardiomyocytes and cAMP signaling pathway) (Fig. 3H, Additional file 3: Table S10). In particular, RyR2, CACNA1S, and CACNA1S were highly enriched and identified as prominent targets in arrhythmia treatment among these three pathways. Moreover, these three targets were related to 5 of the 11 compounds: palmitic Acid, vitamin A acid, pachymic acid, caprylic acid, adenosine (Fig. 3I, Additional file 3: Table S12). These findings highlighted these candidate targets as FSM therapeutic hubs for arrhythmia treatment. In addition, multiple FSM compounds interacted with these candidate targets and eventually acquired the ability to combat arrhythmia.

### Protective action of FSM against the BaCl2 induced arrhythmia phenotypes

#### Pericardia edema measurement

**Fig. 4:**
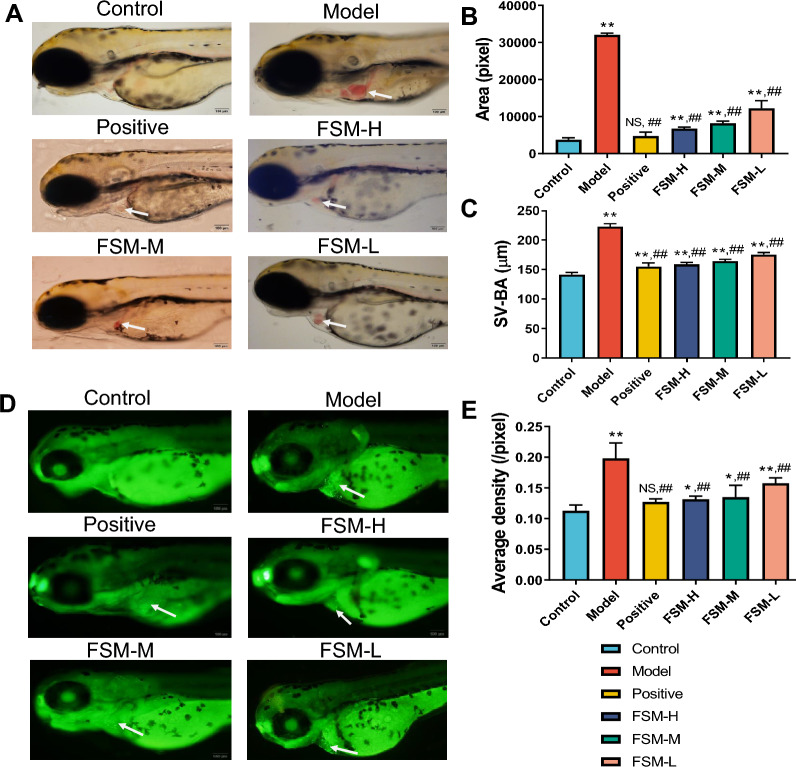
Protection effects of FSM on BaCl_2_ induced arrhythmia phenotypes. **A** Cardiac morphology observation of live zebrafish embryos after 24 h FSM treatment. **B** Pericardial congestion area and **C** the SV-BA straight line were measured in the heart. **D** Acridine orange (AO) staining  and **E** AO staining fluorescence density of live zebrafish embryos after 24 h FSM treatment. AO-positive fluorescent staining was observed in the heart. White arrowheads: representative regions of pericardia edema area and AO-positive cells. The results of the statistical analysis are presented in bar charts (n = 6, mean ± s.d.).*p < 0.05, **p < 0.005 compared with control gruop; #p < 0.05, ##p < 0.005 compared with model group. NS, not significant

Hallmark characteristics of BaCl_2_-induced arrhythmia included pericardia edema, whose phenotypes could be qualified by measuring the pericardial area and heart length from the sinus venosus (SV) to the bulbous arteriosus (BA) [30, 38]. Throughout the experiment, the control group was found to be well developed, and no abnormal signs in the heart, pericardial edema, haemorrhage or long and narrow hearts were observed in the model group after treatment with BaCl_2_ (Fig. 4A, white arrowheads). ImageJ software was used to quantify the SV-BA spacing, bleeding area and fluorescence density. The SV-BA spacing and bleeding area of the model group increased significantly compared with the control group (p < 0.01). FSM shortened the SV-BA and decreased the bleeding area in a dose-dependent manner (Fig. 4B, C, detailed measured data are shown in Additional file 4: Table S13).

#### Cardiomyocytes apoptosis observation

Cell death was assayed with cardiomyocytes with acridine orange (AO). Compared to the control group, epifluorescence pictures of lateral views of whole embryos confirmed the apoptotic phenotype in the model group (Fig. 4D, white arrowheads). By contrast, treatment with FSM led to reduced AO staining fluorescence density in a dose-dependent manner (Fig. 4E, Additional file 4: Table S13). These results suggested that FSM improved the phenotype of arrhythmia and inhibited cardiomyocyte apoptosis.

### *Selection of FSM therapeutic biomarkers based on its dose-dependent manner *via* metabolomic approaches*

**Fig. 5:**
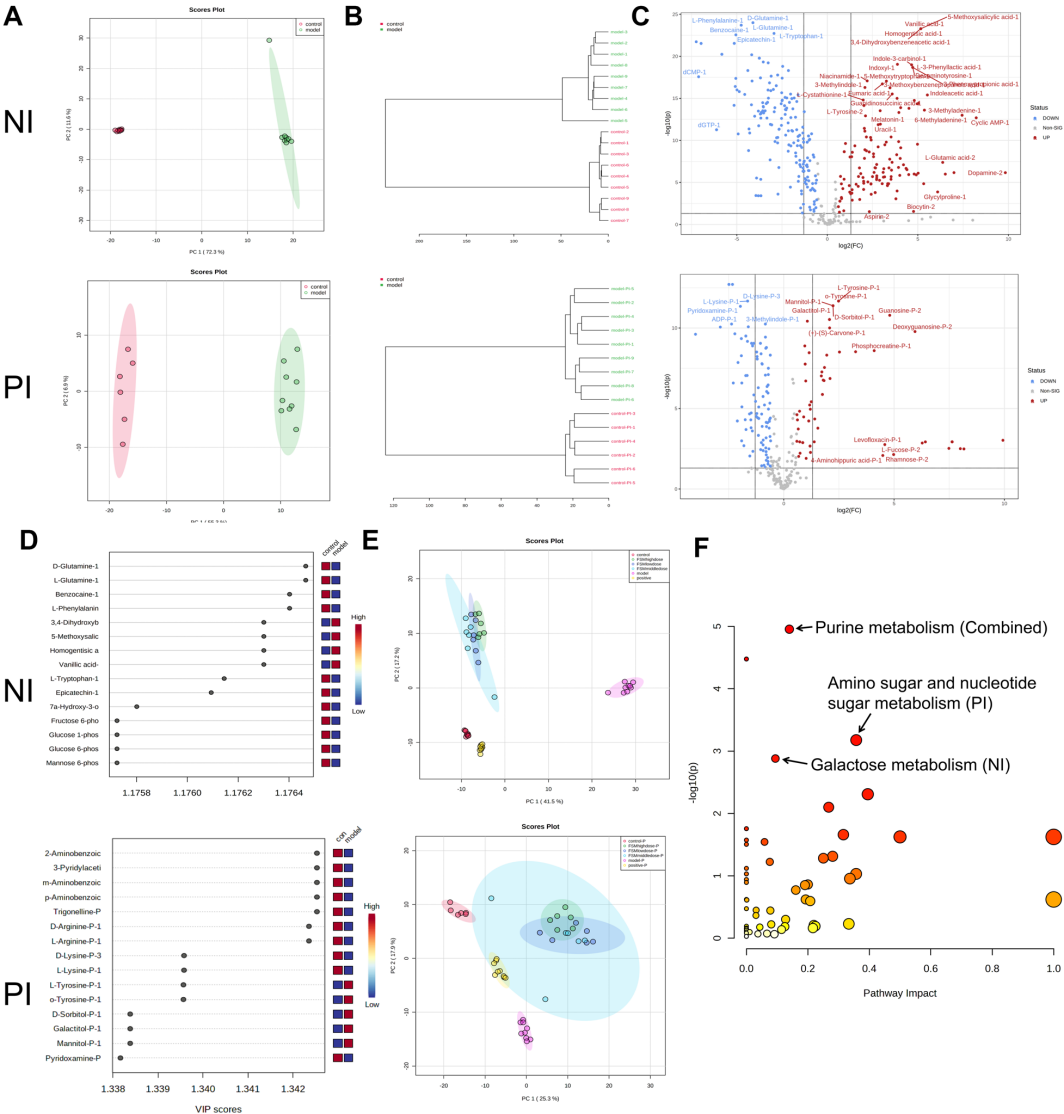
FSM rescues the metabolic state induced by BaCl_2_ of the zebrafish embryos. **A** PCA scores plot (component 1 and component 2) for biomarkers selection between control group and model group. **B** Unsupervised hierarchical clustering dendrogram by Euclidean distance for groups’ separation analysis. **C** Volcano plot of differentially metabolites between control group and model group (model/control) using an adjusted p-value < 0.05 and |Log_2_FC |> 1 as cut off. **D** PLS-DA variable importance in projection (VIP) score plot with the top 15 important features ranked by their PLS-DA VIP scores. The colored boxes on the right represent the relative concentrations of the corresponding metabolite in each group. **E** PCA comparison for treatment effects trends of FSM among the six groups. The clustered colored dots represent the samples from each group. **F** Pathway analysis of FSM therapeutic markers. Annotated bubbles represent the important pathways from combined metabolite markers (194 markers, adjusted *p*-value (Holm *p*) < 0.05 and Impact > 0.1), negative ion (NI) metabolite markers (172 markers, Holm *p* < 0.05), and positive ion (PI) metabolite markers (44 markers, Holm *p* < 0.05)

Based on these phenotype results and the canonical role that FSM played in regulating the metabolism of the cardiovascular system, metabolomic analysis related to FSM treatment was conducted by comparing six groups: control, model, positive control, and three FSM treatment groups. Control and model samples were used for arrhythmia biomarker analysis and the six groups for selection of FSM therapeutic markers. A series of analyses followed to ensure the robustness of the data. First, PCA was performed in control and model groups in two scan types. It was found that both the control and model groups were well separated (Fig. 5A). As the hierarchical clustering dendrogram shows, the model group and control group showed a good trend of intragroup aggregation (Fig. 5B). After PLS-DA and selection of biomarkers using such indexes as a cutoff value of |Log_2_FC |> 1, p < 0.05, and VIP > 1, a total of 224 metabolites in the negative ion (NI) scan mode and 109 metabolites in the positive ion (PI) mode were screened (Fig. 5C, D, detailed metabolomic matrix are shown in Additional file 5: Tables S14, S15). Principal component (PC) analysis can be used to reveal dominant modes of similarity among the different groups. PCA was also performed in these six groups for selection of FSM therapeutic markers. PCA comparison results of negative and positive ion on the multi-group samples are shown in Fig. 5E. One noticeable difference was that negative ion data separated individuals from model and FSM rescue group more clearly than the positive ion data. Moreover, the three dosage groups of FSM in the negative ion data were positioned more separately than positive ion data. Taken together, although there was some overlap among the three groups of FSM in two ion data, there was significantly separation for the control, model and FSM treatment. Compared to the model group, FSM-treated embryos formed a distinct rescue trending cluster, suggesting that FSM could facilitate the recovery of these biomarkers (Fig. 5E). Specifically, according to statistical one-way ANOVA analysis among these groups, a total of 194 metabolites (172 in the NI mode and 44 in the PI mode) were selected as FSM therapeutic markers that were related to significant rescue manners (Additional file 5: Tables S16, S17, S18). Pathway enrichment analysis of FSM therapeutic metabolite markers in negative ion mode (172 metabolites, Holm p < 0.05) was mainly focused in two pathways: purine metabolism (Holm *p* = 0.003, including GDP, D-Ribose 5-phosphate, cAMP, ATP, ADP, dAMP, Xanthosine, Inosine, Allantoate, GTP, Guanosine, ITP, and dGTP) and galactose metabolism (Holm *p* = 0.041, alpha-D-Glucose, D-Glucose 1-phosphate, UDP-glucose, alpha-D-Galactose, Galactitol, D-Sorbitol, and myo-Inositol). Meanwhile, pathway enrichment analysis of FSM therapeutic metabolite markers in positive ion mode (44 metabolites, Holm p < 0.05) was mainly focused in galactose metabolism (Holm *p* = 0.004, alpha-D-Glucose, alpha-D-Galactose, Galactitol, D-Sorbitol, and myo-Inositol) and amino sugar and nucleotide sugar metabolism (Holm *p* = 0.027, N-Acetyl-D-glucosamine, N-Acetyl-D-mannosamine, alpha-D-Glucose, alpha-D-Galactose, and beta-D-Fructose) (Additional file 5: Table S19, Figure S2). Interestingly, the pathway enrichment of combined FSM therapeutic metabolite markers (Holm *p* < 0.05 and Impact > 0.1) focused on ‘purine metabolism’ (Holm *p* = 0.001, Impact = 0.139, Fig. 5F, Additional file 5: Table S19). These results suggest that overall the impact of FSM was mainly focused on purine metabolism, followed by galactose metabolism, as well as amino sugar and nucleotide sugar metabolism.

### Location of FSM therapeutic pathways

**Fig. 6:**
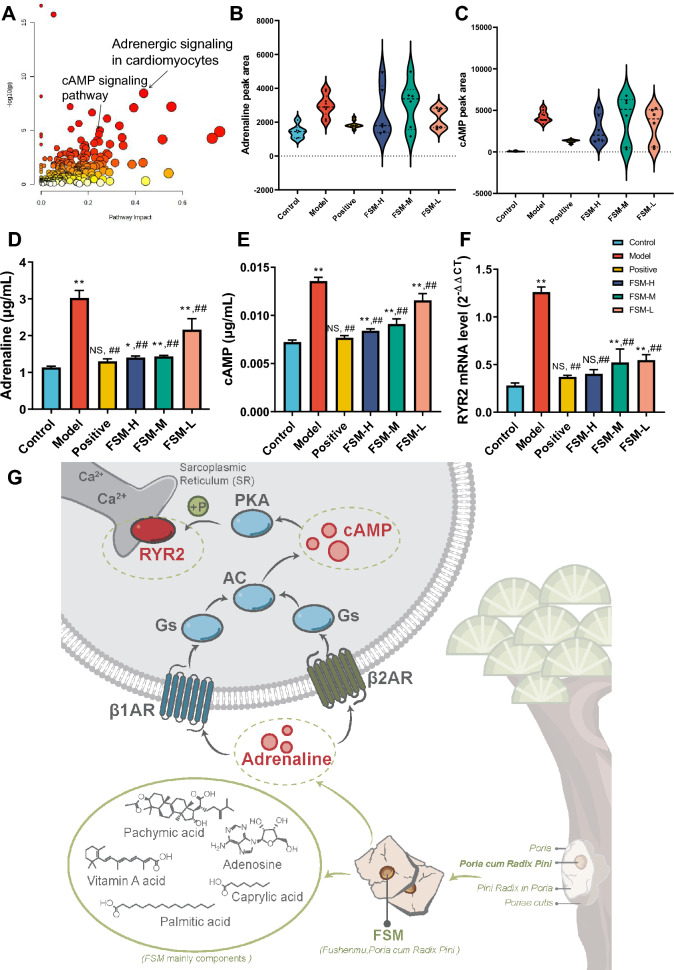
Effect of FSM from arrhythmia zebrafish exposure to BaCl_2_ and therapeutic markers (targets and metabolites) verification. **A** FSM Joint pathways analysis for FSM therapeutic targets and metabolomes. Annotated bubbles represent the important joint pathways using Holm p < 0.05 and Impact > 0.2 as cut off. Violin plots of (**B**) the adrenaline peak area and **C** the cAMP peak area of the QTOF-MS/MS spectra for selected FSM therapeutic metabolites in located pathways. Quantified FSM therapeutic biomarkers levels of **D** the adrenaline and **E** the cAMP (n = 6, mean ± s.d.). **F** qRT-PCR verification of FSM therapeutic target RYR2 mRNA expression (n = 3, mean ± s.e.m.). The results of the statistical analysis are presented in bar charts.*p < 0.05, **p < 0.005 compared with control gruop; #p < 0.05, ##p < 0.005 compared with model group. NS, not significant. **G** FSM exerts a significant rescue effect on BaCl_2_ induced arrhythmia characterized by elevated RYR2, adrenaline and cAMP in zebrafish embryos

After pooling of FSM therapeutic targets and markers, for identified regulatory pathways dissecting, integration analysis was conducted between the 33 candidate targets and 194 metabolites. Such integration of the data could help obtain more information than from target prediction and metabolomics separately. According to joint pathway analysis (Holm p < 0.05, Impact > 0.2, disease terms were excluded), FSM affected genes or metabolites involved in the signal transduction (adrenergic signaling in cardiomyocytes, cAMP signaling pathway, glucagon signaling pathway, Insulin secretion, oxytocin signaling pathway), purine metabolism, galactose metabolism, citrate cycle (TCA cycle), dopaminergic and cholinergic synapse, and tyrosine metabolism (Fig. 6A, Additional file 6: Table S20). It was evident that FSM regulation of arrhythmia was highly concentrated in adrenergic signaling in cardiomyocytes and cAMP signaling pathway (Fig. 6B, C, Additional file 6: Table S21). Notably, FSM played a pivotal role in regulating the RyR2 as well as adrenaline, cAMP in the two pathways (Additional file 6: Figures S3, S4).

### FSM therapeutic actions on RyR2 and its related metabolites

#### FSM rescues arrhythmia-induced adrenaline and cAMP abnormality

On this basis, a simultaneous quantitative method was established for adrenaline and cAMP involved in adrenergic signaling in the cardiomyocyte pathway and cAMP signaling pathway (detailed LC–MS/MS MRM conditions and determined methodologies are shown in Additional file 7: Table S22. The total ion chromatograms of the markers are shown in Additional file 7: Figure S5). After BaCl_2_ exposure, the levels of adrenaline and cAMP in the model group were 1.2 times and 0.8 times higher than those in the control group, respectively. Interestingly, the FSM administration group had an obvious recovery effect on adrenaline and cAMP (Fig. 6D, E, Additional file 7: Table S23–S25). These data proved FSM to be effective in improving abnormal elevation of adrenaline and cAMP in case of arrhythmia.

#### FSM inhibits arrhythmia-induced high RyR2 mRNA expression

The qRT-PCR results showed that the RyR2 mRNA expression in the model group was three times that of the control group, and FSM had a recovery effect on the abnormal increase in RyR2 mRNA (Fig. 6F, Additional file 7: Table S26). Therefore, it was assumed that abnormal opening of RyR2 channels was one of the pathogenic mechanisms of arrhythmia caused by BaCl_2_, and that the improvement of RyR2 channel function by inhibiting the overexpression of RyR2 mRNA might be a potential mechanism of the antiarrhythmic action of FSM.

## Discussion

According to statistics, arrhythmia is the most important cause of sudden cardiac death, which poses a serious threat to human life and health [26, 39]. Recently, Chinese medicine has attracted increasing attention because of its relatively few side effects and the property of “multicomponent, multitarget, multipathway” treatment. Traditionally, FSM is generally reserved for arrhythmia developed palpitation symptoms. However, its mechanisms have remained unclear. Our study was intended to unravel the molecular mechanisms of FSM on arrhythmia. We began by identifing changes in gene functions between arrhythmia classic AF patients and controls. Then, network was constructed between FSM, arrhythmia and function targets to screen therapeutic targets. The network revealed that 11 main active components of FSM acted on 33 candidate therapeutic targets. Furthermore, the changes in phenotype and metabolic functions associated with these targets were analyzed from the arrhythmia zebrafish model. Here, arrhythmia developed phenotype like pericardia edema, and ardiomyocyte apoptosis was significantly rescued, while most of the abnormal metabolites were restored.

To gain keen insights into the contributions of FSM to arrhythmia, integrated prediction of targets and metabolomics analysis were performed to locate FSM therapeutic pathways. The analysis focused on pathways in adrenergic signaling in cardiomyocytes and cAMP signaling pathway that revealed the key targets and metabolites. Correspondingly, we focused on the target RyR2 and its related metabolites-adrenaline as well as cAMP. Corresponding analytical methods were adopted to determine the changes in RyR2 gene expression and its related metabolites.

We investigated RyR2 mRNA expression by RT-PCR and found that the RyR2 mRNA level was closely related to the occurrence of arrhythmia caused by BaCl_2_. The RyR2 mRNA level in the model group was 3.3 times higher than that in the control group and was significantly decreased by FSM. RyR2’s intraluminal loop in sarcoplasmic reticulum (SR), composed of amino acids 4789–4844, is responsible for luminal Ca^2+^ sensing [40]. As the main Ca^2+^ release channel from the SR, the cardiac RyR2 provides a major pathway for untimely Ca^2+^ release that can precipitate Ca^2+^-dependent ventricular tachyarrhythmias, which can cause sudden cardiac death [41–43]. It is well known that increased oxidative stress in cardiovascular disease enhances RyR2 activity through reversible posttranslational modifications such as oxidative modifications and CaMKII (Ca /calmodulindependent protein kinase II)-dependent phosphorylation [44–48]. Abnormal expressions of RyR2 often lead to an abnormal opening frequency of its channel and ultimately calcium leakage in the diastolic phase, causing ectopic discharge, which leads to the occurrence and maintenance of arrhythmia [49–51]. Documents also demonstrated that CaMKII phosphorylation of RyR2 at serine 2814 unmasks the RyR2 mutations’ latent arrhythmogenic potential [52].

Because overexpression of RYR2 may regulates the disease development through modulating the metabolism pathway. We further observed a series specific changes in metabolites profiling from the FSM intervention zebrafish model. In the process of detecting the RyR2 mRNA levels, cAMP and adrenaline closely related to arrhythmia in this pathway were quantitatively determined. Adrenaline (also known as epinephrine), is a hormone that is released by adrenal glands. In cardiovascular system, adrenaline can stimulate both β1 and β2 adrenergic receptors (β1AR and β2AR), which belong to superfamily of G protein-coupled membrane receptors (GPCR) [53]. Subsequently, the β-AR activates the Gs-adenylyl cyclase (AC) -cAMP-protein kinase A signaling cascade, which converts ATP to cAMP[54, 55]. cAMP is a major second messenger downstream of β–adrenergic receptors in discrete subcellular microdomains. Activating protein kinase A (PKA), it can regulate calcium release from the sarcoplasmic reticulum (SR) via ryanodine receptors (RYRs) and responsible for cardiac function and disease [42, 56]. Since aberrant increased local cAMP signals can cause RyR2 hyperphosphorylation by PKA that drive excessive increases in calcium wave generation and ultimately result in arrhythmias [57]. Thus, exposure to BaCl_2_ in zebrafish, Ba^2+^ promotes the secretion of adrenaline, that might generate abnormal metabolic signals, leading to increased RyR2 expression and result in arrhythmia. In this work, we show that FSM exerted recovery effect on abnormally elevated cAMP and adrenaline induced by BaCl_2_ (Fig. 6G). A comparison of the levels of adrenaline and cAMP between the control group, model group and administration group found that adrenaline and cAMP levels in the model group were 1.2 times and 0.8 times higher than those in the control group, respectively.

## Conclusion

It has long been believed that FAM performs a particularly critical function in arrhythmia treatment. However, its mechanisms and molecular consequences have remained obscure. As noted above, our data suggest that RyR2 may be a promising FSM therapeutic target to arrhythmogenesis in that it can improve cardiac function by restoring the adrenaline and cAMP levels in arrhythmia linked pathways. Importantly, by integrating targets data digging and metabolomic analysis, our present study has helped elucidate the contributions of FSM to arrhythmia in the signaling pathway. It is hoped that the methodologies adopted here will help explore the signaling pathways and metabolism pathways, including therapeutic markers, which participate in multiple, seemingly unrelated biological processes.

### Supplementary Information

**Additional file 1: Table S1-1.** The 17 compounds in FSM extract. **Table S1-2.** LC/MS–MS MRM optimized parameters for the 5 compounds from FSM. Figure S1 MRM chromatograms of the quantifier transitions of adenosine, 2-methoxycinnamic acid, pinocembrin, vitamin A acid, and pachymic acid. **Table S1-2.** Linear equations, linear ranges, quantitative limits, and detection limits for 5 mainly compounds from FSM. **Table S1-3.** Precision, stability and repetitive results of 5 mainly compounds from FSM. **Table S1–4.** Recovery results of 5 mainly compounds from FSM. **Table S1–5.** Quantification results of 5 mainly compounds from FSM. **Table S2.** FSM compounds related targets set.

**Additional file 2: Table S3.** Arrhythmia associated targets dataset in LA-PV junction and LAA samples. **Table S4.** GSE41177 GSEA C5 ontology gene analysis (p.adjust value < 0.05, arrhythmia related top terms). **Table S5.** GSE41177 GO analysis (p.adjust value < 0.05, arrhythmia related top terms).

**Additional file 3: Table S6.** 161 common targets between ‘FSM—compounds—targets’ and ‘arrhythmia—targets’. **Table S7.** Node degree results from 161 targets. **Table S8.** Interaction combined score results of 161 targets. **Table S9.** ‘FSM—compounds – arrhythmia– targets’ network node. **Table S10.** GO analysis for 33 candidate targets (p.adjust value < 0.05, arrhythmia related top terms). **Table S11.** ‘FSM—compounds – arrhythmia– targets’ network node. **Table S11.** Most enrichment targets and compounds from ‘FSM—compounds – arrhythmia– targets’ network.

**Additional file 4: Table S13.** FSM effects on BaCl2 induced pericardia edema and cardiomyocytes apoptosis (n = 6, mean ± s.d.).

**Additional file 5: Table S14.** Biomarkers selection between control group and model group from negative ion mode (p < 0.05, (|Log2-fold change |> 1, VIP > 1). **Table S15.** Biomarkers selection between control group and model group from positive ion mode (p < 0.05, (|Log2-fold change |> 1, VIP > 1). **Table S16.** FSM rescued effects among the control, model and treatment groups from negative mode. **Table S17.** FSM rescued effects among the control, model and treatment groups from positive mode. **Table S18.** 194 FSM therapeutic markers. **Table S19.** Pathway analysis from the FSM therapeutic metabolomic markers (Top 10 pathway, the pathway of either negative or positive ion metabolite markers with the cutoff value of Holm p < 0.05 is highlighted in yellow. The pathway of combined metabolite markers with the cutoff value of Holm p < 0.05 and Impact > 0.1 is highlighted in yellow). **Figure S2.** Pathway analysis of FSM therapeutic markers of (A) negative ion and (B) positive ion.

**Additional file 6: Table S20.** Joint pathway analysis from the FSM therapeutic ‘target- metabolite’ markers (Top15, Holm p < 0.05, Impact > 0.2). **Table S21.** Metabolites levels of adrenaline and cAMP. **Figure S3.** Adrenergic signaling in cardiomyocytes. Highly enrichment modules were pointed in red. C00575: Cyclic AMP, C00788: adrenaline. **Figure S4.** cAMP signaling pathway. Highly enrichment modules were pointed in red. C00575: Cyclic AMP, C00788: adrenaline.

**Additional file 7: Table S22.** Optimized MS parameters for the adrenaline and cAMP. **Figure S5.** The total ion chromatograms for adrenaline and cAMP. **Table S23.** Linear, repeatability, precision, and stability for adrenaline and cAMP. **Table S24.** Matrix effect for adrenaline and cAMP. **Table S25.** Simultaneous determination for adrenaline and cAMP in zebrafish embryos (n = 6, mean ± s.d.). **Table S26.** qRT-PCR analysis for RYR2 mRNA (n = 3, mean ± s.d.).

## Data Availability

Some or all data, models, or code generated or used during the study are available from the corresponding author upon request.

## Abbreviations

AADs: Antiarrhythmic drugs
AC: Adenylyl cyclase
AF: Atrial fibrillation
ANOVA: Analysis of variance
AO: Acridine orange
BaCl_2_: Barium chloride
CaMKII: Ca/calmodulindependent protein kinase II
cAMP: 3',5'-Cyclic AMP
CE: Collision energy
CES: Collision energy spread
CM: Cardiomyopathy
CUR: Curtain gas
CVD: Cardiovascular disease
CXP: Collision cell exit potential
DEGs: Differentially expressed genes
DP: Declustering potential
FC: Fold change
FDR: False discovery rate
FSM: Fushenmu
GEO: Gene expression omnibus
GO: Gene ontology
GS1: Ionsource gas 1
GS2: Ionsource gas 2
GSEA: Gene set enrichment analysis
HF: Heart failure
JP-2: Junctophilin-2
KEGG: Kyoto encyclopedia of genes and genomes
LAA: Left atrial appendage
LA-PV junction: Left atrial junction
LSD: Least significant difference
MRM: Multiple reaction monitoring
NES: Normalized enrichment scores
NI: Negative ion
PA: Pachymic acid
PCA: Principal components analysis
PI: Positive ion
PKA: Protein kinase A
PLS-DA: Patterns-partial least squares discriminant analysis
PVCs: Premature ventricular contractions
qRT-PCR: Quantitative reverse transcriptase polymerase chain reaction
RyR2: Ryanodine Receptor 2
SCD: Sudden cardiac death
SV-BA: Sinus venosus–Bulbus arteriosus
TCM: Traditional Chinese medicine
TEM: Source temperature
UPLC-QTOF-ESI–MS/MS: Ultra-high-performance liquid chromatography–tandem mass spectrometry
VIP: Variable importance in projection
