# Supplementary material for: Fushenmu treatment ameliorates RyR2 with related metabolites in a zebrafish model of barium chloride induced arrhythmia
Source: Chin Med. 2023 Aug 19;18:103. doi: 10.1186/s13020-023-00812-x (PMC10439546; doi:10.1186/s13020-023-00812-x)
Supplement: Supplementary file 1 — Additional file 1: Table S1-1. The 17 compounds in FSM extract. Table S1-2. LC/MS–MS MRM optimized parameters for the 5 compounds from FSM. Figure S1 MRM chromatograms of the quantifier transitions of adenosine, 2-methoxycinnamic acid, pinocembrin, vitamin A acid, and pachymic acid. Table S1-2. Linear equations, linear ranges, quantitative limits, and detection limits for 5 mainly compounds from FSM. Table S1-3. Precision, stability and repetitive results of 5 mainly compounds from FSM. Table S1–4. Recovery results of 5 mainly compounds from FSM. Table S1–5. Quantification results of 5 mainly compounds from FSM. Table S2. FSM compounds related targets set. [file 13020_2023_812_MOESM1_ESM.docx]

**ADDITIONAL FILE 1 MATERIAL**

***Fushenmu* Treatment Ameliorates RyR2 with Related Metabolites in a Zebrafish Model of Barium Chloride induced Arrhythmia**

Yan-Ting Zhao^1,+^, Yan-Ru Liu^1,+ ,*^, Ya-Feng Yan^1,+^, Zhi-Shu Tang^1,*^, Jin-Ao Duan^2^, Hui Yang^1^, Zhong-Xing Song^1^, Xue-Lian You^1^, Ming-Geng Wang^3^

^1^ Shaanxi University of Chinese Medicine, Shaanxi Collaborative Innovation Center Medicinal Resource Industrialization, No. 1 Weiyang Road, Qindu District, Xianyang 712083, P.R. China

^2^ Nanjing university of Chinese medicine, No. 138 Xianlin Road,Nanjing 210023, P.R. China

^3^ Shandong Buchang Pharmaceutical Co. Ltd, 250000, Shandong, P.R. China

^*^Corresponding authors:

Yan-Ru Liu, Tel: +86 029 38182201, Email: yanzi_2203@aliyun.com;

Zhi-Shu Tang, Tel: +86 029 38183461, Email: [tzs6565@163.com](mailto:tzs6565@163.com);

^+^These authors contributed equally to this paper

**Abstract**

*Fushenmu* (*Pini Radix in Poria*, FSM) has been mainly used for palpitation and amnesiain in traditional Chinese medicine (TCM), especially for arrhythmia. However, there have been no reports on its antiarrhythmic mechanism. In this study, it was found that *FSM* recovered arrhythmia-associated heart failure in barium chloride (BaCl_2_) induced arrhythmic zebrafish embryos, as was evidenced by the shortened cardiac sinus venosus - bulbus arteriosus (SV-BA) distance, smaller cardiovascular bleeding areas, and reduced cardiomyocyte apoptosis. Moreover, analysis via ultra-high-performance liquid chromatography–tandem mass spectrometry (UPLC-QTOF-ESI-MS/MS) components identification and network pharmacology prediction showed that 11 main active components of FSM acted on 33 candidate therapeutic targets. Metabolomic analysis also suggested that FSM could rescue 242 abnormal metabolites from arrhythmic zebrafish embryos. Further analysis based on the combination of target prediction and metabolomic results illustrated that FSM down-regulated Ryanodine Receptor 2 (RyR2) expressions, inhibited adrenaline and 3',5'-Cyclic AMP (cAMP) levels in a dose-dependent manner, which was confirmed by metabolites quantification and quantitative reverse transcriptase polymerase chain reaction (qRT-PCR) assay. In summary, this study revealed that FSM mitigated BaCl_2_ induced cardiac damage caused by arrhythmia by suppressing RyR2 expressions, decreasing adrenaline and cAMP through the adrenergic signalling pathway.

**Additional file 1 File 1 for FSM therapeutic targets set**

*1.Liquid chromatography and mass spectrometry method used for detecting the major components in the FSM extract.*

The gradient program was as follows: 0-2 min, 10 % B; 2-50 min, 10 %-100 % B; and 50-55 min, 100 % B. The flow rate and column temperature were set at 0.3 mL/min and 30 °C, respectively, and the injection volume was 5 µL.

The collection parameters under positive (5500 V) or negative (−4500 V) ion mode were as follows: IDA experiments; DP, 80 V; CE, 35 eV; CES, 15 eV; ionsource gas 1 (GS1) and ion source gas 2 (GS2), nitrogen gas, 50 psi; curtain gas (CUR), 35 psi; TEM, 550 °C. A full scan was run in both positive and negative modes (mass range: m/z 100 to 1500 amu; accumulation time: 200 ms). The data were acquired using Analyst® TF 1.6 software (AB Sciex). Peakview software 2.2 was used to visualize the MS/MS data. Component identification was conducted by TCM library 1.0 in Masterview 1.1.0.0.

*2.The information of the 17 chemical constituents in FSM extract and 5 mainly constituents determination.*

*2.1 The information of the 17 chemical constituents in FSM extract*

**Table S1-1** The 17 compounds in FSM extract

| **No.** | **Name** | **Formula** | **Mass** | **RT (min)** | **Area** |
| --- | --- | --- | --- | --- | --- |
| 1 | Pachymic acid | C_33_H_52_O_5_ | 528.3815 | 33.11 | 13032 |
| 2 | Adenosine | C_10_H_13_N_5_O_4_ | 267.0967 | 0.62 | 1681 |
| 3 | Vitamin A acid | C_20_H_28_O_2_ | 300.2089 | 20.18 | 12844 |
| 4 | Pinocembrin | C_15_H_12_O_4_ | 256.0736 | 17.15 | 2887 |
| 5 | 2-Methoxycinnamic acid | C_10_H_10_O_3_ | 178.063 | 6.69 | 832 |
| 6 | Choline | C_5_H_14_NO^+^ | 104.17 | BATMAN database | |
| 7 | Turanose | C_12_H_22_O_11_ | 342.3 |  |  |
| 8 | Ergotamine | C_33_H_35_N_5_O_5_ | 581.7 |  |  |
| 9 | Palmitic Acid | C_16_H_32_O_2_ | 256.42 | ETCM database | |
| 10 | Lauric acid | C_12_H_24_O_2_ | 200.32 |  |  |
| 11 | Caprylic acid | C_8_H_16_O_2_ | 144.21 |  |  |
| 12 | Tumulosic Acid | C_31_H_50_O_4_ | 486.7 |  |  |
| 13 | Dehydroeburicoic acid | C_31_H_48_O_3_ | 468.7 |  | |
| 14 | Nardosinone | C_15_H_22_O_3_ | 250.16 |  | |
| 15 | Lauric Aldehyde | C_12_H_24_ | 184.32 | TCMSP database | |
| 16 | Ergosterol | C_28_H_44_O | 396.6 |  |  |
| 17 | L-uridine | C_9_H_12_N_2_O_6_ | 244.2 |  | |

*2.2 Quantificaiton methodology of 5 mainly constituents from FSM determination.*

2.2.1 Materials

Reference substances included Adenosine, 2-Methoxycinnamic acid, Pinocembrin, Vitamin A acid, Pachymic acid, and all had >98% purity that were purchased from ichuan Weikeqi Bio-technology company (Chengdu, China). The solid reference substances were used to generate standard stock solutions. An appropriate amount of each reagent was precisely weighed and dissolved in 50% methanol to obtain Adenosine, Coniferaldehyde, Pinocembrin, Vitamin A acid, Pachymic acid concentration were 0.015 μg/mL, 0.24 μg/mL, 0.48 μg/mL, 0.37 μg/mL, 16.07 μg/mL of standard stock solution. The stock solution was filtered through a 0.22-μm membrane filter, and the resulting filtrate was collected and stored at 4°C. Mixed liquid samples were precisely acquired. The blank solution showed no interference peak at the retention times of the characteristic ion mass chromatographic peaks of the 5 standards. The blank interference was subtracted to calculate the 5 compounds contents.

2.2.2 Preparation of sample solutions

FSM materials were obtained from Shaanxi Xingshengde Co., Ltd. (Shannxi, China) and identified by Professor Ji-Qing Bai. The voucher specimen was deposited in the Shaanxi Collaborative Innovation Center of Chinese Medicine Resources Industrialization, Shaanxi University of Chinese Medicine (Shaanxi, China).

0.4 g precisely weighted FSM powder was ultrasonically extracted with 25 mL methanol for 60 min. Then, the extract was centrifuged (6, 000 rpm, 10min), 15 mL of supernatant was collected and vacuum lyophilized. For compound identification, the lyophilized extracts were re-dissolved in 5 mL methanol, centrifuged at 12, 000 rpm for 10 minutes before the supernatant was filtered through a 0.22 μm microporous membrane. For target validation on a zebrafish model, the lyophilized extracts were re-dissolved in zebrafish incubation solution and prepared for 1 mg/mL mother solution that was stored at 4°C until use.

2.2.3 LC-MS conditions

2.2.3.1 LC condition

LC analysis was performed on an Agilent 1290 UPLC system (Agilent Corp.) with a ACQUITY UPLC® BEH C18 (50 mm×2.1 mm, 1.7 μm) column. The mobile phase was a water (A)–acetonitrile (B) solution. Mobile phase A was composed of water +0.1% formic acid while mobile phase B was acetonitrile +0.1% formic acid. The elution program was set as follows: 0-2 min, 10 % B; 2-50 min, 10 %-100 % B; and 50-55 min, 100 % B. The column temperature was 30°C, the flow rate was 0.3 mL/min, and the injection volume was 5 μL.

2.2.3.2 MS condition

Mass spectrometry experiment was carried on an ABI-Sciex 4500 QTRAP hybrid, triple quadrupole, linear ion trap mass spectrometer equipped with ion electrospray ionization (ESI) (AB sciex). Negtive-ESI-MS analysis was performed in multiple reaction monitoring (MRM) mode. The ionization parameters were as follows: collision gas was nitrogen, ion spray voltage, 5.5 kV, turbo ion spray source temperature, 500°C auxiliary gas temperature, 50°C for nebulizer gas (GS1) and 50°C for turbo-gas (GS2), and curtain gas temperature, 35°C. Compounds were analysed via multiple reaction monitoring (MRM) that declustering potentials (DP), collision energies (CE) and collision cell exit potential (CXP) were optimized and parameters were shown in **Table S1-2**. The ion chromatograms of the standard solutions and the total chromatograms of the samples are shown in **Figure S1**. Data were acquired and quantified by Analyst 1.6.2 and Multiquant software, respectively.

**Table S1-2** LC/MS–MS MRM optimized parameters for the 5 compounds from FSM

| **Compound Name** | **Retention**  **Time** | **Molecular Formula** | **Precursor ion (Da)** | **Product ion (Da)** | **Declustering potential (V)** | **Collision Energy (eV)** | **Collision Cell Exit Potential** |
| --- | --- | --- | --- | --- | --- | --- | --- |
| Adenosine | 1.31 | C_10_H_13_N_5_O_4_ | 266.0 | 133.9 | -72.53 | -23.66 | -9.17 |
| 2-Methoxycinnamic acid | 7.84 | C_10_H_10_O_3_ | 176.8 | 161.9 | -55.35 | -19.2 | -10.88 |
| Pinocembrin | 15.57 | C_15_H_12_O_4_ | 255.0 | 151.0 | -114.5 | -29.81 | -9.14 |
| Vitamin A acid | 19.91 | C_20_H_28_O_2_ | 299.5 | 255.1 | -150.28 | -19.78 | -7.99 |
| Pachymic acid | 30.5 | C_33_H_52_O_5_ | 527.0 | 465.3 | -196.27 | -51.13 | -15.18 |


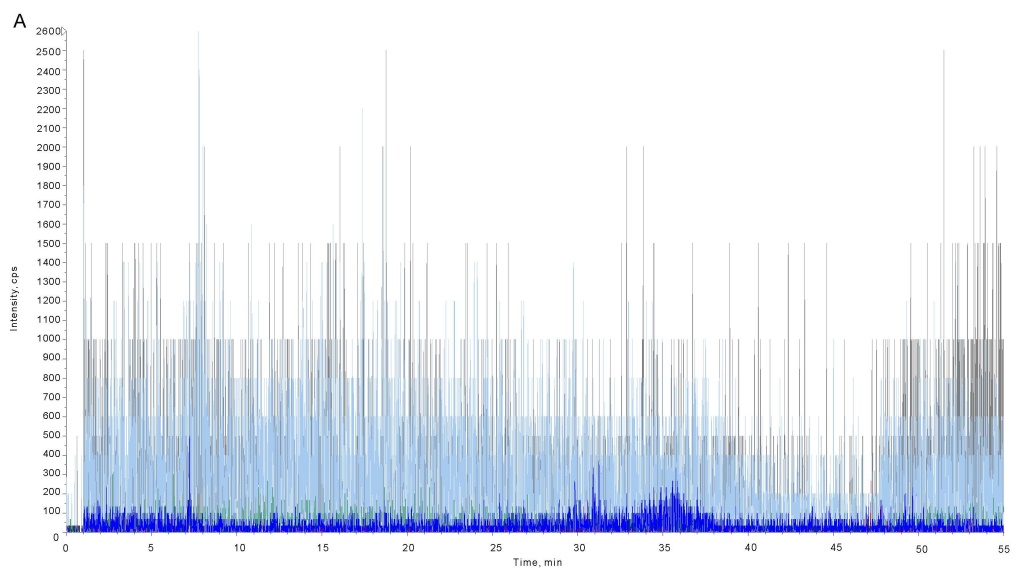


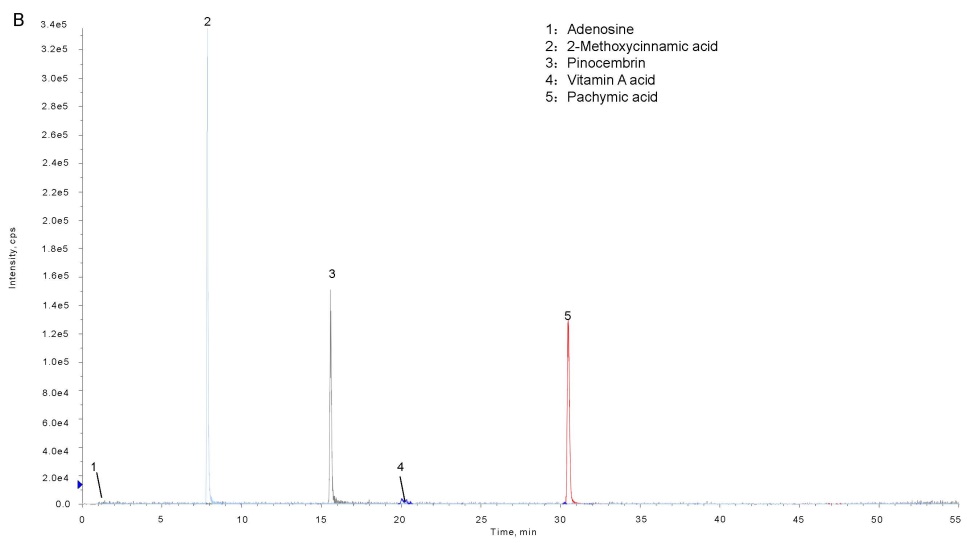


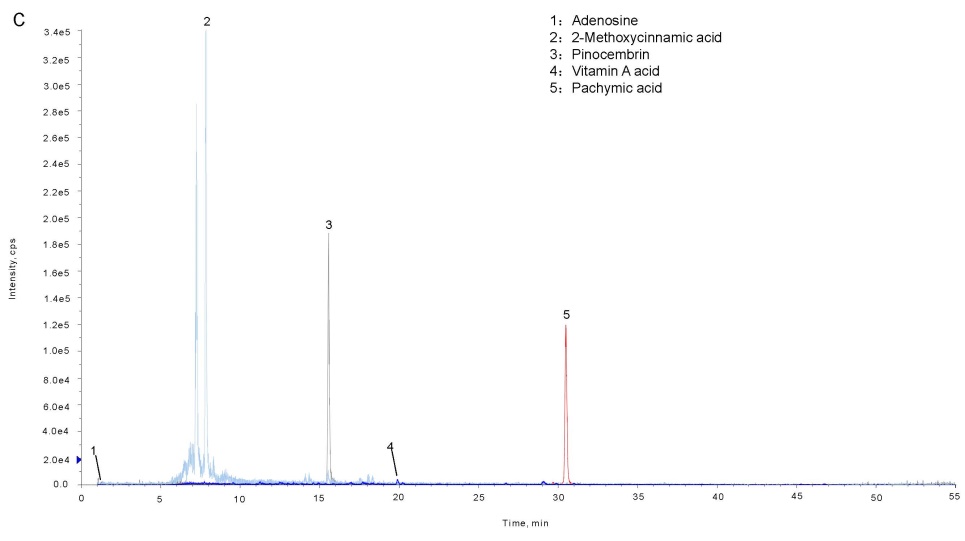


**Figure S1** MRM chromatograms of the quantifier transitions of adenosine, 2-methoxycinnamic acid, pinocembrin, vitamin A acid, and pachymic acid

A. Blank solution B. Mixed standard solution C. FSM sample solution

2.2.3 Standard curve

Different volumes of mixed standard solution were added to the blank samples, and the resulting samples were analysed using the optimized conditions determined in this study. The limits of detection (LOD, signal/noise [S/N] ≥ 3) and quantitation (LOQ, S/N ≥ 10) were calculated. The LOD ranged between 1.45E-03 and 6.09E-04 µg/mL, and the LOQ ranged between 4.34E-03 and 9.14E-04 µg/mL.

The mixed standard working solution prepared in section 2.2.1 that containing 5 compounds was precisely measured and diluted to seven gradient volumes. Then, 5 μL of each solution was injected, and the analysis was performed using the optimal conditions selected. The standard working curve was plotted with the peak area (Y) as the ordinate and the alkaloid concentration (X, μg/mL) as the abscissa. The standard curve equation and range of each compounds were then obtained. The results showed that for concentrations in the linear range of 0.0071~30.0 μg/mL, the 5 compounds had an average correlation coefficient (r) > 0.999, indicating a good linear relationship between X and Y. The linear equations and correlation coefficients of the 5 compounds are listed in **Table S1-2**.

**Table S1-2** Linear equations, linear ranges, quantitative limits, and detection limits for 5 mainly compounds from FSM

| **Compound name** | **Linear Equation** | **Linear range (μg/ml)** | **Detection limit（μg/ml）** | **Quantification limit （μg/ml）** |
| --- | --- | --- | --- | --- |
| Adenosine | y = 2E+08x - 669.03；R² = 0.9992 | 0.0071~0.03 | 6.09E-04 | 9.14E-04 |
| 2-Methoxycinnamic acid | y = 6E+06x + 266037；R² = 0.9995 | 0.11~0.45 | 1.86E-04 | 6.20E-04 |
| Pinocembrin | y = 2E+06x - 147514；R² = 0.999 | 0.22~0.9 | 2.17E-03 | 4.34E-03 |
| Vitamin A acid | y = 4E+07x + 2575.5；R² = 0.9991 | 0.17~0.69 | 2.37E-03 | 7.10E-03 |
| Pachymic acid | y = 5E+07x + 465287；R² = 0.9994 | 7.3~30 | 1.45E-03 | 4.34E-03 |

2.2.4 Precision

The mixed standard working solution prepared in section 2.2.1 was precisely measured and continuously injected six times. The precision test was performed under the experimental conditions determined in this study. The response signals of the 5 compounds had relative standard deviations (RSDs) between 0.35% and 2.67% indicating good precision (**Table S1-3**).

2.2.5 Stability

The same batch of samples was tested at 0, 3, 6, 9, 12, and 24 h after preparation. The stability test was conducted under the experimental conditions determined in this study. The respective signal areas of the 5 compounds had RSDs between 1.00% and 2.25%. These results indicate that the sample solutions were stable for 24 h (**Table S1-3**).

2.2.6 Repeatability

Six replicates of the same sample were prepared as described in section 2.2.2. The repeatability was tested under the experimental conditions determined in this study. The respective signal intensities of 5 compounds were recorded. The results showed that the signal response areas of the compounds had RSDs between 1.66% and 2.87% indicating that the proposed approach has good repeatability (**Table S1-3**).

**Table S1-3** Precision, stability and repetitive results of 5 mainly compounds from FSM

| **Compound name** | **Precision RSD (%)** | **Stability RSD (%)** | **Repeatability RSD (%)** |
| --- | --- | --- | --- |
| Adenosine | 2.67 | 1.07 | 2.87 |
| 2-Methoxycinnamic acid | 0.41 | 1.93 | 1.83 |
| Pinocembrin | 1.98 | 2.25 | 2.27 |
| Vitamin A acid | 2.04 | 1.75 | 1.78 |
| Pachymic acid | 0.35 | 1.00 | 1.66 |

2.2.7 Recovery

Six parallel samples of medicinal material with known contents were precisely weighed and combined with reference substance solutions containing 100% mixed standards. The samples were injected under the conditions determined in this study, and the signal intensities of the compounds were recorded. The results showed that the average recoveries of spiked compounds ranged between 102.93% and 112.27%, with RSDs of 1.37%–2.81%. These values indicate that the proposed approach has good accuracy (**Table S1-4**).

**Table S1-4** Recovery results of 5 mainly compounds from FSM

| **Compound name** | **Results of recovery rate of sample addition (%)** | **RSD (%)** |
| --- | --- | --- |
| Adenosine | 102.93 | 1.37 |
| 2-Methoxycinnamic acid | 111.62 | 1.80 |
| Pinocembrin | 107.28 | 2.81 |
| Vitamin A acid | 112.27 | 1.41 |
| Pachymic acid | 105.16 | 2.42 |

2.2.8 Determination of 5 compounds from FSM

Three batches of medicinal material were tested using the established approach. The sample solutions were prepared as described in section 2.2.2 and injected under the experimental conditions determined in this study. The signal intensities of the 5 compounds were recorded. The active ingredient contents were calculated by the external standard method (**Table S1-5**).

**Table S1-5** Quantification results of 5 mainly compounds from FSM

| **Compound name** | **Adenosine** | **2-Methoxycinnamic acid** | **Pinocembrin** | **Vitamin A acid** | **Pachymic acid** |
| --- | --- | --- | --- | --- | --- |
| Content (mg/g) | 0.0004 | 0.018 | 0.036 | 0.017 | 0.8105 |

*3. FSM‘compound-targets’ set*

Table S2 FSM compounds related targets set

| **NO.** | **FSM compounds** | **Compounds related targets** |
| --- | --- | --- |
| 1 | Vitamin A acid | RXRB,RARRES1,NR0B1,ALDH1A1,RARG,RARA,GPRC5A,ALDH1A2,RARB,RXRG,ALDH1A3,ALDH2,RXRA,ALDH1B1,ALDH3A1,COL27A1,FKBP1B,RDH11,RBP3,RETSAT,RDH13,DHRS3,DHRS4,RDH5,RBP1,RLBP1,RDH14,RDH8,LRAT,RDH12,GPRC5D,FKBP1A,MTOR,S100P,KCNMA1,FADS1,PPARD,SLC8A1,SLC6A8,PTGS1,GAMT,CKMT1B,ACSL4,CKMT2,PTGS2,CKMT1A,ELOVL4,TRPV1,CKB,FFAR1,CKM,ACSL3,FADS2,APOA2,SC5D,SLC16A12,LPCAT1,SLC9A1,IL13,GATM,SERPINB7,PGR,IHH,LARP4B,ESRRG,FGF4,GATA3,ZNF536,TBC1D32,MED1,FMR1,RET,FOXA1,NUFIP2,MEX3C,NOTCH1,RPL7A,EIF2AK1,WDR77,TSPO,GDF5,ALDH8A1,SHBG,FGF10,ABCF1,SERPINH1,NPPC,SOX9,AR,DHRS9,VDR,MATN1,CYP1A1,FGF2,CYR61,TCF3,PAX2,ASIC1,KCNQ3,SCN4A,PLA2G2A,ADRA2C,ALOX5,ADRA2A,KCNQ2,NOS2,ANXA1,NR3C1,ADRA2B,CD55,NR2F1,ALDH9A1,ALDH1L2,NR2F6,NR2F2,HNF4G,ALDH1L1,CREBBP,LSR,UBE3A,DNAJB14,HSP90B1,PRKCA,EIF3A,SP1,NCOA3,MED25,PPP3CA,TRIP4,ITGB3BP,NCOR2,CCND3,NR1I2,NR1H2,UBE2D4,PNRC1,NR0B2,ADPRHL2,POU2F1,RAB3GAP1,COPS2,CLOCK,BTG1,PML,MRPL12,NR1H3,TMEM57,FAU,MED6,IRX4,CLTA,EZH2,NSD1,RHPN2,CPLX1,NCOR1,SUV39H1,ACY3,GADD45A,NKX2-1,GATA2,ESR2,MTMR6,NCOA1,PRDX6,IL24,MBD1,SPHK1,TBCA,KLHL11,THRB,MAP6,LIMD1,KDM5A,ZNF148,TADA3,HMGA1,AJUBA,SMAD3,WTIP,RORA,NCOA2,HNRNPL,NR4A1,NUDT21,KAT2B,BAG1,PNRC2,ESR1,PARP1,GADD45G,ITGB1BP2,SSB,ARNTL,STAT5B,NPAS2,TDG,PNO1,SGSM2,KIF5A,ZDHHC12,NR5A1,CDK7,SPEN,ESRRA,MBP,PPARA,MCFD2,NR4A2,KLF5,NFE2L2,CNOT1,SKI,ZBTB16,RPAP2,MAGEB2,FOXO1,LRSAM1,APPL1,NR2E3,TACC2,ZNF423,SPOP,HDAC4,APCDD1,SRF,NRBF2,VCP,HDAC3,SOS2,AKAP13,STX18,PTMS,FN1,ZNF576,MECR,SNW1,POT1,SORBS3,OIP5,PCSK6,NR1H4,MAP4,SREBF1,THRA,CHGB,NR2C2,NRIP2,TRIM24,TAF11,CAMK2B,NUPR1,ATP5I,NCOA6,CACNA1B,SRC,NRIP1,RBP7,DNMT1,DHRS2,RBP5,ABCC2,NDRG2,NAMPT,RGCC,SLC6A4,SP3,OPN3,HOPX,BDKRB2,NEUROD2,FNDC5,SCD5,SLC8B1,AGTR2,JAK3,GNB2L1,ANAPC2,OSBPL8,WNT11,JUNB,IGF1,AVPR1A,ALOX15B,ELOVL3,CDC20,DNAJA3,FASLG,KCNA5,CACNA1A,PLA2R1,ITGB3,COL1A1,METRNL,ELOVL1,MAPK9,NR1D1,MAP4K4,ANXA13,SLC3A2,PAXBP1,OPN4,STX3,SOX15,OXER1,ELOVL7,PNPLA2,CAPN3,PAX7,SCD,ABCA1,NAPRT,CYP4F2,MECOM,PLCB1,ABHD5,PTGIS,CNST,SLC8A2,GJD4,SLC8A3,SNCA,ADIPOQ,RYR2,NODAL,CPLX2,MYL2,ALOX15,SLC35G1,NOS1,TRPM8,PAWR,S100A9,HEG1,FABP3,ITGAV,GNAT2,MYOD1,ELOVL6,ABCG1,PRDM16,PTPN2,S100A8,ALOX5AP,CCM2L,AVP,TRPA1 |
| 2 | Adenosine | ADORA2A,ADORA3,ADORA1,ADORA2B,SLC25A6,SLC25A5,TACR2,RIPK1,CX3CR1,ADA,HAP1,ABCC2,ACVR1,ASNA1,ADRBK2,ACSS2,PRKAA1,ABCC9,ACSS1,ABL1,AFG3L2,ACVRL1,ADCY1,CDK15,NT5C2,AMHR2,TNK2,ASS1,ASNS,ACVR1B,ACSL1,ABCA1,ABL2,ADRBK1,NAE1,APAF1,ABCC8,ALK,ABCB11,ABCB1,ABCG1,ARAF,SLC25A4,TYR,TYMS,FBP1,PRKAB1,RRM2,POLE,CREB1,PIM1,PYGL,POLE2,POLE3,HINT1,POLA1,RRM2B,ADK,PDE4B,PNP,PRKAB2,POLE4,RRM1,PDE4D,CACNB1,CACNA1D,SLC6A4,CACNB3,KCNH2,CACNA1F,AKT3,CACNA1A,ABCB4,CACNA1I,CACNA1G,CACNB2,KCNJ11,CACNA1S,PRKAA2,CACNB4,AKT2,CACNA1B,CBSL,MAT1A,CBS,COMT,DCK,MAT2A,AMD1,GNMT,PDE5A,UTS2R,HMGCR,DGKI,FURIN,UTS2,KHDRBS1,SLC18A3,CRH,ATP1A2,HOMER1,SHANK3,AVPR2,SPX,ATP2A1,SMO,ADRA2A,CETN2,CRP,CETN1,PIK3CG,IDO1,AGT,KCNB1,DRD1,SORCS3,LTA,SLC44A4,GHRL,NCBP2,PCSK6,DRD2,DNM3,CHRNA3,SCN9A,PTGDR2,PLAT,PTGR2,CFTR,PTGER2,PTGS1,PLA2G2A,THBD,PTGS2,GLO1,PTGER1,PPARA,BCL2,FABP2,TPK1,SLC19A2,TOP2A,DHFR,TOP2B,SLC6A3,PDE7B,PDE7A,PDE9A,PECR,ADRA1A,PDE4A,RYR1,SLC18A2,ITPR3,SLC12A2,MAPK1,DRD4,PDE4C,PDE3A,HTR7,SLC12A7,SLC12A6,SLC12A5,ADRB2,HTR1A,ADRA1B,ACACB,MTAP,ITPR1,ADRA2C,DBH,PDE6A,PTGER3,PDE10A,PDE1C,SLC25A31,DRD3,PRKDC,PIK3CD,HTR1B,ACSL5,DRD5,ATM,SRPK2,PDE3B,SLC12A1,PTGIS,PDE6B,PIK3CA,ACVR1C,PIK3R3,PDE2A,PIK3R2,ADRB1,PIK3R1,PDE1A,PTGER4,APRT,ADRB3,TGFBR1,PDE8B,SLC18A1,PIK3CB,ACP1,SLC12A4,ABCG4,ADRA1D,PDE6C,ITPR2,ADRA2B,PDE11A,P2RY12,PTGIR,PDE1B,PAH,SLC6A2,PDE8A,CACNG1,ACSL6,CUBN,TCN1,AMN,MUT,MMACHC,MMAB,MTRR,POLB,MMAA,DNMT1,MTR,FBP2,ATF4,SAXO1,XBP1,AK9,RALA,BICD1,UGCG,JAG1,ASNSD1,SMAD3,CASP9,TGFBR2,VLDLR,ABCA7,UBA3,GRB2 |
| 3 | Choline | PLD1,PCYT1A,PCYT1B,PHOSPHO1,ACHE,PLD2,BCHE,COLQ,SLC5A7,HMOX1,NRG1,DMGDH,SIX3,RAP1GAP,CRP,NAPEPLD,FSCN1,PRSS12,KLF5,SLC44A4,ALDH7A1,DNM3,PLD4,ASCL1,GPLD1,ENPP6,CDH8,PLD3,PODXL,FNTA,AGRN,CHKA,CHDH,ATP8B1,GRIN1 |
| 4 | Ergosterol | VDR,CYP27B1,GC,SNW1,NFKB1,AKR1C3,CYP24A1,GPBAR1,SNAI2,MED1,SNAI1,CYP3A4,CALB1,ESR1,FGF23,GFI1,PGR,LANCL2,WNT4,TCF3,BAX,KL,CYP2R1,PML,B4GALT1,S100G,CYP27A1,KANK2,IRX5,RXRA,NR1H4,TRIM24 |
| 5 | Pachymic acid | PGR,AR,NR3C2,HMGCR,ESR1,PTGER4,NR3C1,ITGB2,PTGER2,PTGER3,PTGFR,ANXA1,ITGAL,CYP19A1,HDAC2,LTK,CAMK2A,CRP,TIGAR,CAMK2D,ADORA1,RYR1,MAPK1,PIK3CA,CREB1 |
| 6 | Ergotamine | ADRA1A,HTR2A,ADRA2A,HTR1B,HTR2C,ADRA1D,HTR1D,DRD2,HTR1F,HTR1A,ADRA1B,DRD5,DRD1,ADRA2B,SLC6A2,HTR2B,DRD4,HTR7,ADRA2C,DRD3,DNMT3A,PHKG2,APLP1,CHRNA7,CNR1,SLC22A1,CD34,CALCA,HTR4,GABRA2,GABRD,GABRB1,GABRG3,HTR6,GABRE,GABRA3,GABRG1,GABRP,GABRA4,GABRB2,HTR3D,GABRQ,GABRB3,HTR3B,GABRA5,HTR3E,HTR1E,GABRA6,HTR3A,GABRA1,GABRG2,SLCO2B1,HTR3C,TNFSF11,SULF2,EPM2A,GNB5,DGKI,FEZF2,NAV2,CYGB,P2RX2,ATP1A3,ARX,FLNA,CNTN2,ADRB2,AVPR1A,DTNBP1,GSK3A,MAPK8IP2,SULF1,CRHR1,LILRB1,CETN2,CETN1,OPRL1,TRPV1,ADCY5,PPP1R1B,CAV2,CABP1,MAOB,P2RX3,HMBS,LHX6,ARRB2,IL13,ADRB1,FLOT1,PDE4D,FGFR2,HRH3,APLN,GHRL,TBR1,RPS6KA1,OXTR,KCNK4,LEP,CHRNB2,ARRB1,NCOA3,SRI,RAB3B,CRH,SHANK1,AVPR2,UROS,LRRK2,TACR2,PDGFA,FGFR1,ASS1,ZP3,ATP2B4,ARRDC3,ADA,PARK2,KCNMA1,ADORA1,ADAM17,PINK1 |
| 7 | Turanose | CXCR4 |
| 8 | Lauric Aldehyde | SCN11A,SCN2B,ABAT,SCN1A,SCN3B,SCN3A,SCN7A,SCN10A,AKR1D1,ALDH5A1,SCN2A,HDAC9,TYR,SCN9A,SCN4A,SCN4B,SRD5A2,ACADSB,SCN8A,SCN1B,OGDH,HDAC2 |
| 9 | Palmitic Acid | LALBA,PPT1,PAEP,CYP2C8,PMP2,SEC14L2,RHO,TRAPPC3,HNF4G,AKR1D1,TYR,SRD5A2,SCN11A,SCN2B,ABAT,SCN1A,ESRRG,SCN3B,COX6C,COX5B,COX7C,COX1,SCN3A,AKR1C2,SCN7A,SCN10A,ALDH5A1,SCN2A,COX5A,COX3,AR,COX7A1,FECH,COX4I1,HDAC9,PLA2G1B,COX6A2,SCN9A,SCN4A,SCN4B,ADH1C,COX6B1,FABP6,CES1,ACADSB,SCN8A,COX7B,SCN1B,COX2,OGDH,HDAC2,COX8A,NR1H4,PLD1,PCYT1A,PCYT1B,PHOSPHO1,ACHE,PLD2,BCHE,SUCLG2,SLC13A2,SLC13A1,SUCLG1,HSD17B6,SLC25A10,P3H3,OXCT2,TMLHE,PLOD1,SUCNR1,SLC13A3,ASPH,BBOX1,SDHB,OXCT1,PLOD3,P4HA1,SDHC,P3H2,SDHA,P4HA2,SUCLA2,SDHD,P3H1,DCT,AKR1C3,CRTAP,SLIT2,ACO2,JMJD6,SLC13A4,SDHAF2,SLC1A3,SALL1,HSD17B11,TYRP1,HIF1AN,UROS,SLC13A5,P4HB,CACNA2D1,PLAT,SLC7A2,CACNA1A,GRIN3B,CACNA2D2,GRIN2A,TOP1,PLG,CACNA1B,GRIN2C,GRIN2B,SLC7A3,KARS,GRIN3A,SLC7A1,SLC7A4,GRIN2D,ADORA1,GRIN1,RDH11,SLC25A20,RBP3,RETSAT,RDH13,DHRS3,RDH5,MPO,SLC25A29,ALDH1A3,RDH12,SLC22A4,CPT2,DHRS4,RBP1,ALDH1A1,RLBP1,RDH14,CPT1A,XDH,RDH8,CROT,LRAT,SLC22A5,ALDH1A2,CRAT,HDAC1,GPD1L,SRD5A1,FBXO45,SIRT1,SHMT1,SUGCT,ACSS2,ARV1,SIX1,BDH1,COLGALT2,SIX4,MRPS36,OSBPL8,SPI1,SUOX,CCL2,BCKDK,UBE2B,CYP1A2,CCL5,ANK2,HSD17B2,STIM2,PKD2,ALDH9A1,TH,EPO,COLGALT1,NEDD4,CYP11A1,DHODH,CLN3,ARID1A,EGLN2,ACAT1,GALC,PLCG2,ACOT4,PHGDH,PNLIPRP2,CYP39A1,BCKDHA,SLC25A12,YBX3,44628,STIM1,GAL3ST1,CDH11,CACNA1D,DDC,DHTKD1,NEDD4L,BDKRB2,PRPH,BCKDHB,KCNE5,SRD5A3,SMAD7,PRDM8,UROD,UGT8,ETHE1,WNT10B,OGFOD1,SLC38A7,ACSS1,CYP4B1,STAR,DLST,PF4,CRACR2A,HSD17B1,CAMK2D,AP3D1,MYO5A,ILVBL,CAV3,HTT,IL10,EGLN3,OGDHL,KCNA5,TST,SLC1A6,NPPA,ACADM,SQRDL,TMEM110,CDC42,ARHGEF2,EGLN1,ETFDH,EPHA4,OCA2,HSD17B8,P4HA3,EDA,HACL1,RYR2,HAP1,PCSK9,KCNQ1,CASQ2,GJA5,DHRS9,GPR143,UGT1A1,DLD,GNAS,SLC25A13,SFXN5,PLOD2,CYP1A1,SULT2A1,NFX1,DBT,SLC25A1 |
| 10 | Lauric acid | AKR1D1,TYR,SRD5A2,SCN11A,SCN2B,ABAT,SCN1A,ESRRG,SCN3B,COX6C,COX5B,COX7C,COX1,SCN3A,AKR1C2,SCN7A,SCN10A,ALDH5A1,SCN2A,COX5A,COX3,AR,COX7A1,FECH,COX4I1,HDAC9,PLA2G1B,COX6A2,SCN9A,SCN4A,SCN4B,ADH1C,COX6B1,FABP6,CES1,ACADSB,SCN8A,COX7B,SCN1B,COX2,OGDH,HDAC2,COX8A,NR1H4,FASN,SUCLG2,SLC13A2,SLC13A1,SUCLG1,HSD17B6,SLC25A10,P3H3,OXCT2,TMLHE,PLOD1,SUCNR1,SLC13A3,ASPH,BBOX1,SDHB,OXCT1,PLOD3,P4HA1,SDHC,P3H2,SDHA,P4HA2,SUCLA2,SDHD,P3H1,DCT,AKR1C3,CRTAP,SLIT2,ACO2,JMJD6,SLC13A4,SDHAF2,SLC1A3,SALL1,HSD17B11,TYRP1,HIF1AN,UROS,SLC13A5,P4HB,CACNA2D1,PLAT,SLC7A2,CACNA1A,GRIN3B,CACNA2D2,GRIN2A,TOP1,PLG,CACNA1B,GRIN2C,GRIN2B,SLC7A3,KARS,GRIN3A,SLC7A1,SLC7A4,GRIN2D,ADORA1,GRIN1,SLC8A1,TRPV1,FFAR1,FADS1,PPARD,PTGS1,ACSL4,PTGS2,PPARA,ACSL3,HDAC1,GPD1L,SRD5A1,FBXO45,SIRT1,SHMT1,SUGCT,ACSS2,ARV1,SIX1,BDH1,COLGALT2,SIX4,MRPS36,OSBPL8,SPI1,SUOX,CCL2,BCKDK,UBE2B,CYP1A2,CCL5,ANK2,HSD17B2,STIM2,PKD2,ALDH9A1,TH,EPO,COLGALT1,NEDD4,CYP11A1,DHODH,CLN3,ARID1A,EGLN2,ACAT1,GALC,PLCG2,ACOT4,PHGDH,PNLIPRP2,CYP39A1,BCKDHA,SLC25A12,YBX3,44628,STIM1,GAL3ST1,CDH11,CACNA1D,DDC,DHTKD1,NEDD4L,BDKRB2,PRPH,BCKDHB,KCNE5,SRD5A3,SMAD7,PRDM8,UROD,UGT8,ETHE1,WNT10B,OGFOD1,SLC38A7,ACSS1,CYP4B1,STAR,DLST,PF4,CRACR2A,HSD17B1,CAMK2D,AP3D1,MYO5A,ILVBL,CAV3,HTT,IL10,EGLN3,OGDHL,KCNA5,TST,SLC1A6,NPPA,ACADM,SQRDL,TMEM110,CDC42,ARHGEF2,EGLN1,ETFDH,EPHA4,OCA2,HSD17B8,P4HA3,EDA,HACL1,RYR2,HAP1,PCSK9,KCNQ1,CASQ2,GJA5,DHRS9,GPR143,UGT1A1,DLD,GNAS,SLC25A13,SFXN5,PLOD2,CYP1A1,SULT2A1,NFX1,DBT,SLC25A1 |
| 11 | Caprylic acid | AKR1D1,TYR,SRD5A2,SCN11A,SCN2B,ABAT,SCN1A,ESRRG,SCN3B,COX6C,COX5B,COX7C,COX1,SCN3A,AKR1C2,SCN7A,SCN10A,ALDH5A1,SCN2A,COX5A,COX3,AR,COX7A1,FECH,COX4I1,HDAC9,PLA2G1B,COX6A2,SCN9A,SCN4A,SCN4B,ADH1C,COX6B1,FABP6,CES1,ACADSB,SCN8A,COX7B,SCN1B,COX2,OGDH,HDAC2,COX8A,NR1H4,SUCLG2,SLC13A2,SLC13A1,SUCLG1,HSD17B6,SLC25A10,P3H3,OXCT2,TMLHE,PLOD1,SUCNR1,SLC13A3,ASPH,BBOX1,SDHB,OXCT1,PLOD3,P4HA1,SDHC,P3H2,SDHA,P4HA2,SUCLA2,SDHD,P3H1,DCT,AKR1C3,CRTAP,SLIT2,ACO2,JMJD6,SLC13A4,SDHAF2,SLC1A3,SALL1,HSD17B11,TYRP1,HIF1AN,UROS,SLC13A5,P4HB,CACNA2D1,PLAT,SLC7A2,CACNA1A,GRIN3B,CACNA2D2,GRIN2A,TOP1,PLG,CACNA1B,GRIN2C,GRIN2B,SLC7A3,KARS,GRIN3A,SLC7A1,SLC7A4,GRIN2D,ADORA1,GRIN1,SLC25A20,MPO,SLC25A29,SLC22A4,CPT2,CPT1A,XDH,CROT,SLC22A5,CRAT,HDAC1,GPD1L,SRD5A1,FBXO45,SIRT1,SHMT1,SUGCT,ACSS2,ARV1,SIX1,BDH1,COLGALT2,SIX4,MRPS36,OSBPL8,SPI1,SUOX,CCL2,BCKDK,UBE2B,CYP1A2,CCL5,ANK2,HSD17B2,STIM2,PKD2,ALDH9A1,TH,EPO,COLGALT1,NEDD4,CYP11A1,DHODH,CLN3,ARID1A,EGLN2,ACAT1,GALC,PLCG2,ACOT4,PHGDH,PNLIPRP2,CYP39A1,BCKDHA,SLC25A12,YBX3,44628,STIM1,GAL3ST1,CDH11,CACNA1D,DDC,DHTKD1,NEDD4L,BDKRB2,PRPH,BCKDHB,KCNE5,SRD5A3,SMAD7,PRDM8,UROD,UGT8,ETHE1,WNT10B,OGFOD1,SLC38A7,ACSS1,CYP4B1,STAR,DLST,PF4,CRACR2A,HSD17B1,CAMK2D,AP3D1,MYO5A,ILVBL,CAV3,HTT,IL10,EGLN3,OGDHL,KCNA5,TST,SLC1A6,NPPA,ACADM,SQRDL,TMEM110,CDC42,ARHGEF2,EGLN1,ETFDH,EPHA4,OCA2,HSD17B8,P4HA3,EDA,HACL1,RYR2,HAP1,PCSK9,KCNQ1,CASQ2,GJA5,DHRS9,GPR143,UGT1A1,DLD,GNAS,SLC25A13,SFXN5,PLOD2,CYP1A1,SULT2A1,NFX1,DBT,SLC25A1 |
| 12 | Tumulosic Acid | NR3C1,SRD5A1,CYP17A1,SLC8A1,F12,PRLR,ESR1,TRPV1,PTGER1,PTGER4,PGR,AR,PTGIR,OPRK1,FADS2,FADS1,PTGER2,PTGS1,PTGER3,PTGS2,ELOVL4,PTGFR,ANXA1,NR3C2,ESRRG,COX6C,COX5B,COX7C,COX1,AKR1C2,ADH1B,COX5A,COX3,COX7A1,FECH,COX4I1,PLA2G1B,AKR1C1,COX6A2,HMGCR,ADH1C,COX6B1,FABP6,CES1,COX7B,COX2,COX8A,NR1H4 |
| 13 | L-uridine | TYMS,NT5C2,ADK,IMPDH1,ENPP1,POLA1,PNP,POLB,TERT,GSR,GCDH,ERO1B,IVD,NQO2,DPYD,POR,CYB5R1,MAOB,FDXR,DAO,TXNRD1,ACADS,CYB5R3,MAOA,IL4I1,AIFM1,ACOX1,ACADM,NQO1,XDH,DNMT1,ACAD8,NOS1,DLD,GFER,AK5,GUK1,ANKH,ENPP3,APRT,AK9,NUDT12,IMPDH2 |
| 14 | Pinocembrin | VKORC1,PROC,NQO1,VKORC1L1,F2,HTR4,GABRA2,AVPR1A,HTR1B,GABRA3,JUN,OPRM1,AGTR2,PTGS1,AVPR2,HTR1A,PTGS2,HTR3A |
| 15 | Nardosinone | CNR2,TYR,CNR1 |
| 16 | 2-Methoxycinnamic acid | ADORA2A,ADORA2B,ADORA3,ADORA1,FBP1,PRKAB1,ACSS2,POLE,CBS,POLE3,ADCY1,COMT,POLA1,DCK,RRM2B,ADK,GNMT,PNP,PDE4D,CBSL,RRM2,PRKAA1,MAT1A,CREB1,ACSS1,PIM1 |
| 17 | Dehydroeburicoic acid | AKR1D1,TYR,SRD5A2,SCN11A,SCN2B,ABAT,SCN1A,ESRRG,SCN3B,COX6C,COX5B,COX7C,COX1,SCN3A,AKR1C2,SCN7A,SCN10A,ALDH5A1,SCN2A,COX5A,COX3,AR,COX7A1,FECH,COX4I1,HDAC9,PLA2G1B,COX6A2,SCN9A,SCN4A,SCN4B,ADH1C,COX6B1,FABP6,CES1,ACADSB,SCN8A,COX7B,SCN1B,COX2,OGDH,HDAC2,COX8A,NR1H4,SUCLG2,SLC13A2,SLC13A1,SUCLG1,HSD17B6,SLC25A10,P3H3,OXCT2,TMLHE,PLOD1,SUCNR1,SLC13A3,ASPH,BBOX1,SDHB,OXCT1,PLOD3,P4HA1,SDHC,P3H2,SDHA,P4HA2,SUCLA2,SDHD,P3H1,DCT,AKR1C3,CRTAP,SLIT2,ACO2,JMJD6,SLC13A4,SDHAF2,SLC1A3,SALL1,HSD17B11,TYRP1,HIF1AN,UROS,SLC13A5,P4HB,CACNA2D1,PLAT,SLC7A2,CACNA1A,GRIN3B,CACNA2D2,GRIN2A,TOP1,PLG,CACNA1B,GRIN2C,GRIN2B,SLC7A3,KARS,GRIN3A,SLC7A1,SLC7A4,GRIN2D,ADORA1,GRIN1,SLC25A20,MPO,SLC25A29,SLC22A4,CPT2,CPT1A,XDH,CROT,SLC22A5,CRAT,HDAC1,GPD1L,SRD5A1,FBXO45,SIRT1,SHMT1,SUGCT,ACSS2,ARV1,SIX1,BDH1,COLGALT2,SIX4,MRPS36,OSBPL8,SPI1,SUOX,CCL2,BCKDK,UBE2B,CYP1A2,CCL5,ANK2,HSD17B2,STIM2,PKD2,ALDH9A1,TH,EPO,COLGALT1,NEDD4,CYP11A1,DHODH,CLN3,ARID1A,EGLN2,ACAT1,GALC,PLCG2,ACOT4,PHGDH,PNLIPRP2,CYP39A1,BCKDHA,SLC25A12,YBX3,44628,STIM1,GAL3ST1,CDH11,CACNA1D,DDC,DHTKD1,NEDD4L,BDKRB2,PRPH,BCKDHB,KCNE5,SRD5A3,SMAD7,PRDM8,UROD,UGT8,ETHE1,WNT10B,OGFOD1,SLC38A7,ACSS1,CYP4B1,CYP4B1 |
