# Supplementary material for: Fushenmu treatment ameliorates RyR2 with related metabolites in a zebrafish model of barium chloride induced arrhythmia
Source: Chin Med. 2023 Aug 19;18:103. doi: 10.1186/s13020-023-00812-x (PMC10439546; doi:10.1186/s13020-023-00812-x)
Supplement: Supplementary file 2 — Additional file 2: Table S3. Arrhythmia associated targets dataset in LA-PV junction and LAA samples. Table S4. GSE41177 GSEA C5 ontology gene analysis (p.adjust value < 0.05, arrhythmia related top terms). Table S5. GSE41177 GO analysis (p.adjust value < 0.05, arrhythmia related top terms). [file 13020_2023_812_MOESM2_ESM.docx]

**Additional File 2 for arrhythmia related targets set**

*1.* *Arrhythmia associated targets dataset*

**Table S3** Arrhythmia associated targets dataset in LA-PV junction and LAA samples

| NO. | Gene.Symbol | logFC | adj.P.Val |
| --- | --- | --- | --- |
| LAPV (226 DEGs) | | | |
| 1 | DNER | 2.539165 | 0.002095 |
| 2 | S100A12 | -2.41516 | 0.036905 |
| 3 | SOD2 | 2.095925 | 0.002723 |
| 4 | CXCR2 | -2.05422 | 0.033071 |
| 5 | LIN9 | 1.989859 | 0.000957 |
| 6 | CPNE4 | 1.95497 | 0.022038 |
| 7 | NEB | 1.8526 | 0.025212 |
| 8 | SLC3A1 | 1.805728 | 4.27E-05 |
| 9 | CHL1 | 1.791405 | 0.008539 |
| 10 | CLC | -1.78674 | 0.036551 |
| 11 | SLC35G3 | 1.763705 | 0.001948 |
| 12 | C1QC | -1.69472 | 0.010368 |
| 13 | LYVE1 | -1.65033 | 0.004025 |
| 14 | FAM83B | 1.648719 | 0.002149 |
| 15 | RNF128 | 1.639891 | 0.036453 |
| 16 | PEX1 | 1.627397 | 0.00023 |
| 17 | EMR2 | -1.62277 | 0.012086 |
| 18 | CADM2 | 1.585529 | 0.003865 |
| 19 | MUM1L1 | 1.555327 | 0.030251 |
| 20 | VNN2 | -1.54201 | 0.038096 |
| 21 | LOC101926960 | 1.536559 | 0.048577 |
| 22 | RLN1 | -1.53406 | 0.000223 |
| 23 | KLB | 1.533275 | 0.020295 |
| 24 | LRRC7 | 1.533225 | 0.016889 |
| 25 | TYROBP | -1.49705 | 0.023413 |
| 26 | LOC101926975 | 1.492618 | 0.045931 |
| 27 | SHISA3 | 1.483212 | 0.012744 |
| 28 | ZBED9 | 1.472024 | 0.004119 |
| 29 | ANK3 | 1.444184 | 0.007853 |
| 30 | MTPN | 1.439067 | 0.002335 |
| 31 | LRIG1 | 1.430924 | 0.000983 |
| 32 | DDIT4 | -1.42397 | 0.001826 |
| 33 | LIN7A | 1.412243 | 0.005405 |
| 34 | OTOGL | 1.410039 | 0.002384 |
| 35 | C18orf54 | 1.408138 | 0.000668 |
| 36 | SLC7A11 | 1.40129 | 0.007912 |
| 37 | SLA | -1.39536 | 0.0039 |
| 38 | CYBA | -1.38896 | 0.022619 |
| 39 | LRRC49 | 1.385447 | 0.029614 |
| 40 | TPSB2 | -1.3853 | 0.025564 |
| 41 | OR2F2 | 1.385228 | 0.001554 |
| 42 | RP11-73K9.2 | 1.384189 | 0.001305 |
| 43 | CTSZ | -1.38409 | 0.001596 |
| 44 | XK | 1.379552 | 0.008316 |
| 45 | BEX2 | 1.368486 | 0.015738 |
| 46 | TGFB1 | -1.36814 | 0.010369 |
| 47 | TMEM159 | -1.36509 | 0.033071 |
| 48 | SLC27A6 | 1.360987 | 0.007542 |
| 49 | MIR302B | 1.356233 | 0.008694 |
| 50 | GPR85 | 1.354324 | 0.004933 |
| 51 | NPC2 | -1.35313 | 0.001199 |
| 52 | CXCL17 | -1.3443 | 5.24E-05 |
| 53 | C1QA | -1.33644 | 0.004879 |
| 54 | FCER1G | -1.3313 | 0.02419 |
| 55 | ANXA6 | -1.32918 | 0.004119 |
| 56 | NT5DC4 | -1.32778 | 9.58E-07 |
| 57 | ABCB11 | -1.32737 | 7.88E-05 |
| 58 | ATP13A3 | 1.32116 | 0.017662 |
| 59 | MAL2 | 1.318479 | 0.025663 |
| 60 | RCN3 | -1.31242 | 0.002857 |
| 61 | C5AR1 | -1.31221 | 0.017007 |
| 62 | ZNF682 | 1.311862 | 0.02849 |
| 63 | SPTSSB | 1.303638 | 0.000279 |
| 64 | FOLR2 | -1.30328 | 0.011908 |
| 65 | RORB | 1.303215 | 0.002581 |
| 66 | LOC101928614 | 1.301362 | 0.005455 |
| 67 | MGC70870 | 1.301333 | 0.00704 |
| 68 | VWC2 | 1.298247 | 0.016889 |
| 69 | FTCDNL1 | 1.284974 | 0.019669 |
| 70 | LOC728690 | 1.283632 | 0.000279 |
| 71 | PPIB | -1.27716 | 0.004003 |
| 72 | NCKAP5 | 1.275563 | 0.006248 |
| 73 | USP32P2 | 1.275272 | 0.002201 |
| 74 | MS4A4A | -1.27521 | 0.009132 |
| 75 | GPR171 | -1.27452 | 0.001129 |
| 76 | IFI6 | -1.27348 | 0.032519 |
| 77 | CTD-3080P12.3 | -1.27256 | 0.000446 |
| 78 | NKAIN2 | 1.27244 | 0.007841 |
| 79 | TMEM207 | -1.26294 | 0.000349 |
| 80 | ALOX5AP | -1.26258 | 0.019592 |
| 81 | ARF5 | -1.25908 | 0.000279 |
| 82 | NKAIN1 | -1.25754 | 0.000279 |
| 83 | ZNF124 | -1.24883 | 0.026633 |
| 84 | RNASE6 | -1.24547 | 0.045902 |
| 85 | FLJ36848 | 1.243167 | 0.000509 |
| 86 | LOC100652824 | 1.241116 | 0.000688 |
| 87 | ISOC2 | -1.24053 | 0.002617 |
| 88 | LOC100288675 | 1.236288 | 0.004202 |
| 89 | FCN1 | -1.23206 | 0.044839 |
| 90 | CYBB | -1.23006 | 0.038806 |
| 91 | BRINP3 | 1.229511 | 0.021695 |
| 92 | MCOLN1 | -1.22887 | 0.000584 |
| 93 | BTG2 | -1.22414 | 0.010942 |
| 94 | APOOL | 1.222476 | 0.000734 |
| 95 | C9orf16 | -1.22045 | 0.000982 |
| 96 | OOSP2 | -1.21952 | 0.000983 |
| 97 | MYO5B | 1.218037 | 0.017643 |
| 98 | PTGFRN | 1.217057 | 0.001676 |
| 99 | TSPAN15 | -1.21497 | 0.001238 |
| 100 | LOC100131303 | 1.214379 | 0.000803 |
| 101 | NAP1L2 | 1.212488 | 0.004188 |
| 102 | SPI1 | -1.208 | 0.033837 |
| 103 | PGAM5 | -1.20648 | 0.000138 |
| 104 | ZDBF2 | 1.205182 | 0.008228 |
| 105 | RP11-379H18.1 | 1.203742 | 0.002305 |
| 106 | NPHP1 | 1.201484 | 0.025461 |
| 107 | KIRREL3 | 1.199215 | 0.005571 |
| 108 | VAX2 | -1.1897 | 0.000523 |
| 109 | RPS11 | 1.188363 | 0.000279 |
| 110 | ESRRG | 1.185075 | 0.023212 |
| 111 | ND6 | 1.18283 | 0.022756 |
| 112 | MGAM | -1.1826 | 0.03931 |
| 113 | DNAJC9-AS1 | 1.182351 | 5.24E-05 |
| 114 | RP11-672L10.6 | 1.181266 | 0.00173 |
| 115 | MIF | -1.18082 | 0.000803 |
| 116 | EGLN3 | 1.180477 | 0.022381 |
| 117 | PROKR2 | -1.18009 | 0.001042 |
| 118 | STT3A | -1.17848 | 0.025663 |
| 119 | CSRP1 | -1.17603 | 0.04228 |
| 120 | ABCD2 | 1.175122 | 0.036946 |
| 121 | SET | -1.17201 | 0.004804 |
| 122 | LINC00354 | -1.16945 | 0.002955 |
| 123 | CLIC5 | 1.167465 | 0.011983 |
| 124 | MIR3682 | 1.159266 | 0.000726 |
| 125 | ERBB4 | 1.158787 | 0.020998 |
| 126 | NFKBIA | -1.15795 | 0.016414 |
| 127 | NXPE4 | -1.15585 | 4.89E-05 |
| 128 | TMEM171 | -1.15402 | 4.27E-05 |
| 129 | DNAH14 | 1.150454 | 0.002869 |
| 130 | SLC39A8 | 1.148833 | 0.005821 |
| 131 | CTD-2528L19.6 | 1.147666 | 0.000279 |
| 132 | GYPC | -1.14719 | 0.012581 |
| 133 | CDK9 | -1.14631 | 0.000523 |
| 134 | IGFBP6 | -1.14311 | 0.048493 |
| 135 | CST3 | -1.1405 | 0.005915 |
| 136 | SMAP2 | -1.13535 | 0.041927 |
| 137 | ZNF837 | -1.1343 | 0.000279 |
| 138 | LILRA6 | -1.13203 | 0.01068 |
| 139 | NRSN1 | -1.13152 | 0.000138 |
| 140 | NECAP1 | 1.129839 | 2.56E-06 |
| 141 | LOC100190986 | -1.12781 | 0.006266 |
| 142 | PCNXL4 | 1.123825 | 0.006497 |
| 143 | FOXA3 | 1.121365 | 0.003484 |
| 144 | NLGN4Y | 1.120325 | 0.034246 |
| 145 | CFLAR-AS1 | 1.120258 | 0.00754 |
| 146 | RAB8A | -1.1156 | 0.029223 |
| 147 | FCGR2C | -1.11387 | 0.036349 |
| 148 | SFRP1 | 1.110858 | 0.008637 |
| 149 | GLRB | 1.106337 | 0.029833 |
| 150 | TREH | -1.10572 | 7.34E-05 |
| 151 | GNB2 | -1.10174 | 0.014514 |
| 152 | LINC00622 | 1.097446 | 0.003558 |
| 153 | KCNA5 | 1.096732 | 0.048577 |
| 154 | TSC22D3 | -1.09567 | 0.004745 |
| 155 | ESRP1 | 1.095512 | 0.00124 |
| 156 | ALG1 | -1.09195 | 0.002866 |
| 157 | PDAP1 | -1.09074 | 0.010229 |
| 158 | BC113958 | -1.08998 | 0.004097 |
| 159 | ZNF684 | 1.088533 | 0.045171 |
| 160 | PPP1R9A | 1.087369 | 0.035015 |
| 161 | LINC01300 | 1.087292 | 0.002866 |
| 162 | RP11-250B2.6 | 1.085459 | 0.029282 |
| 163 | GPR1 | 1.084229 | 0.048915 |
| 164 | LOC339975 | -1.08363 | 7.98E-05 |
| 165 | CAPN6 | 1.082818 | 0.00604 |
| 166 | PLEKHH1 | 1.079496 | 0.012756 |
| 167 | STK17B | -1.07727 | 0.007223 |
| 168 | TNFSF8 | -1.0722 | 0.031811 |
| 169 | CDCA2 | 1.071422 | 0.025212 |
| 170 | DMC1 | 1.071393 | 0.003558 |
| 171 | KLHL10 | -1.06912 | 0.001305 |
| 172 | PARD6B | 1.064705 | 0.030047 |
| 173 | LOC284788 | -1.06245 | 8.59E-05 |
| 174 | SELM | -1.05593 | 0.008258 |
| 175 | ASB4 | 1.05513 | 0.043444 |
| 176 | PRMT1 | -1.05233 | 0.002738 |
| 177 | LOC101926918 | 1.051802 | 0.001199 |
| 178 | LOC100505978 | -1.05001 | 0.000523 |
| 179 | ROPN1B | 1.048909 | 0.009941 |
| 180 | TMEM63C | 1.047691 | 0.002007 |
| 181 | GGTA1P | -1.04633 | 0.025418 |
| 182 | S100A4 | -1.04586 | 0.011751 |
| 183 | MGC12488 | 1.043879 | 0.002593 |
| 184 | HOXC10 | -1.0432 | 0.04471 |
| 185 | GJC1 | 1.042578 | 0.003062 |
| 186 | MAST1 | -1.04112 | 5.24E-05 |
| 187 | C15orf37 | 1.040054 | 0.003147 |
| 188 | KCNK1 | 1.039648 | 0.026908 |
| 189 | PCDHB9 | 1.038834 | 0.025212 |
| 190 | KRT40 | -1.03872 | 0.000825 |
| 191 | LINC00997 | 1.038649 | 0.007998 |
| 192 | ERCC6L | 1.037465 | 0.002227 |
| 193 | SAMD12 | 1.035438 | 0.041063 |
| 194 | KLF2 | -1.0351 | 0.004209 |
| 195 | HIST1H2AK | 1.034028 | 0.004119 |
| 196 | AX748292 | -1.03079 | 0.00166 |
| 197 | EGF | 1.028389 | 0.019669 |
| 198 | HCN4 | 1.025983 | 0.00173 |
| 199 | SNORA72 | 1.02397 | 0.00174 |
| 200 | CYP1A2 | 1.023249 | 0.007769 |
| 201 | PDE3A | 1.022124 | 0.021458 |
| 202 | DNAJC7 | -1.02154 | 0.001948 |
| 203 | ACSL4 | 1.020821 | 0.016898 |
| 204 | PPP3R2 | 1.020815 | 0.003346 |
| 205 | PTPLAD2 | -1.0196 | 0.034385 |
| 206 | CCR3 | -1.01896 | 0.004003 |
| 207 | CEP97 | 1.017637 | 0.003226 |
| 208 | PPDPF | -1.01702 | 0.009614 |
| 209 | ZNF205-AS1 | 1.016369 | 0.002194 |
| 210 | KANK1 | 1.0159 | 0.000803 |
| 211 | ARRB2 | -1.01449 | 0.014579 |
| 212 | TTC30A | 1.013832 | 0.000245 |
| 213 | PRKG2 | 1.013773 | 0.037338 |
| 214 | CHIC1 | 1.013473 | 0.023242 |
| 215 | CASC15 | 1.012434 | 0.001096 |
| 216 | LOC285422 | -1.01181 | 0.000672 |
| 217 | RPPH1 | -1.00815 | 0.00112 |
| 218 | EDN3 | -1.00813 | 4.27E-05 |
| 219 | RP1-8B22.1 | -1.00785 | 0.000291 |
| 220 | INTS2 | 1.007568 | 0.002201 |
| 221 | MYH7 | 1.007018 | 0.004432 |
| 222 | LPP-AS2 | 1.006767 | 0.041179 |
| 223 | C9orf135 | 1.006357 | 0.042633 |
| 224 | FGF2 | 1.002933 | 0.022202 |
| 225 | DDX26B | 1.002605 | 0.003684 |
| 226 | SACS | 1.001863 | 0.047989 |
| LAA samples (818 DEGs) | | | |
| NO. | Gene.Symbol | logFC | adj.P.Val |
| 1 | S100A8 | -4.08104 | 0.001631 |
| 2 | PSPH | -4.0701 | 0.003834 |
| 3 | S100A9 | -3.22006 | 0.003208 |
| 4 | FCGR3B | -3.05548 | 0.004159 |
| 5 | S100A12 | -3.04149 | 0.004673 |
| 6 | CXCR2 | -2.79549 | 0.01201 |
| 7 | CFD | -2.76317 | 0.023973 |
| 8 | SOD1 | -2.67453 | 0.048084 |
| 9 | RPS21 | -2.5658 | 0.031411 |
| 10 | TYROBP | -2.50714 | 0.005492 |
| 11 | BCL2A1 | -2.50599 | 0.005487 |
| 12 | DPYSL2 | -2.45741 | 0.027588 |
| 13 | RPL36 | -2.4543 | 0.031629 |
| 14 | ECHS1 | -2.44861 | 0.011684 |
| 15 | HLA-DPB1 | -2.40872 | 0.004159 |
| 16 | IGKC | -2.37586 | 0.045282 |
| 17 | ZDHHC20 | -2.37578 | 0.023249 |
| 18 | RPA2 | -2.33488 | 0.026789 |
| 19 | RPS3 | -2.33369 | 0.043154 |
| 20 | SERPINA1 | -2.27255 | 0.004703 |
| 21 | TSC22D3 | -2.24294 | 0.008062 |
| 22 | SMARCE1 | -2.22233 | 0.047023 |
| 23 | COMP | -2.22216 | 0.030508 |
| 24 | NDUFV1 | -2.1916 | 0.046141 |
| 25 | LY96 | -2.1687 | 0.040752 |
| 26 | FAM96B | -2.15514 | 0.024956 |
| 27 | S100A4 | -2.13604 | 0.002516 |
| 28 | FIS1 | -2.13379 | 0.039482 |
| 29 | CLPTM1L | -2.12086 | 0.02114 |
| 30 | IGFBP3 | -2.11863 | 0.013593 |
| 31 | MRPS6 | -2.10351 | 0.017753 |
| 32 | MRPL54 | -2.08711 | 0.011125 |
| 33 | CHCHD2 | -2.08463 | 0.029826 |
| 34 | VIM | -2.08346 | 0.03069 |
| 35 | MSRB2 | -2.06334 | 0.036558 |
| 36 | NDUFA3 | -2.0567 | 0.037265 |
| 37 | PPIB | -2.05552 | 0.009162 |
| 38 | FLT1 | -2.0498 | 0.039641 |
| 39 | GNB2 | -2.04286 | 0.015232 |
| 40 | BOD1 | -2.04012 | 0.031209 |
| 41 | TUBB6 | -2.02703 | 0.030987 |
| 42 | FUNDC2 | -2.02571 | 0.034537 |
| 43 | RAC1 | -2.01132 | 0.046585 |
| 44 | DPYSL3 | -2.01038 | 0.033591 |
| 45 | FXYD1 | -2.00978 | 0.042473 |
| 46 | EPB41L2 | -2.00408 | 0.016473 |
| 47 | EIF3G | -2.00151 | 0.010964 |
| 48 | HIGD2A | -1.99535 | 0.036315 |
| 49 | SNX3 | -1.99218 | 0.040622 |
| 50 | CIAPIN1 | -1.98906 | 0.038186 |
| 51 | MPC2 | -1.98519 | 0.035317 |
| 52 | DUSP6 | -1.98049 | 0.037598 |
| 53 | SELL | -1.95439 | 0.00582 |
| 54 | TSC22D1 | -1.95134 | 0.036624 |
| 55 | CD81 | -1.95022 | 0.037693 |
| 56 | PROK2 | -1.94432 | 0.02087 |
| 57 | GUK1 | -1.93295 | 0.023641 |
| 58 | C1QB | -1.92817 | 0.009526 |
| 59 | HSPE1 | -1.92637 | 0.02176 |
| 60 | ATP1B3 | -1.91436 | 0.011097 |
| 61 | RGCC | -1.91118 | 0.01201 |
| 62 | NPR3 | -1.91078 | 0.043165 |
| 63 | CYFIP1 | -1.90607 | 0.026873 |
| 64 | NUDCD2 | -1.905 | 0.036138 |
| 65 | UFD1L | -1.90444 | 0.005701 |
| 66 | NDUFAF3 | -1.894 | 0.044981 |
| 67 | MNDA | -1.88343 | 0.023841 |
| 68 | GPI | -1.88075 | 0.039294 |
| 69 | PDIA3 | -1.86659 | 0.016124 |
| 70 | VWF | -1.86643 | 0.017549 |
| 71 | KDM4B | -1.86308 | 0.01937 |
| 72 | RPS14 | -1.85587 | 0.048772 |
| 73 | EMR2 | -1.85475 | 0.000863 |
| 74 | BCL2L13 | -1.8543 | 0.034786 |
| 75 | YIPF5 | -1.85352 | 0.036491 |
| 76 | BANF1 | -1.85218 | 0.039561 |
| 77 | C19orf10 | -1.85208 | 0.016162 |
| 78 | VNN2 | -1.84618 | 0.010273 |
| 79 | ATP5G2 | -1.84136 | 0.016199 |
| 80 | RPA3 | -1.83713 | 0.042442 |
| 81 | MYC | -1.83083 | 0.04231 |
| 82 | OST4 | -1.826 | 0.039991 |
| 83 | C1QA | -1.82557 | 0.011125 |
| 84 | MGMT | -1.82203 | 0.040001 |
| 85 | RILP | -1.81274 | 0.016277 |
| 86 | CSF1R | -1.81155 | 0.026789 |
| 87 | ETFB | -1.80465 | 0.036826 |
| 88 | RGS18 | -1.7982 | 0.038047 |
| 89 | KLF2 | -1.79686 | 0.006499 |
| 90 | PRDX4 | -1.79572 | 0.020448 |
| 91 | CST3 | -1.79555 | 0.008062 |
| 92 | IGFBP6 | -1.7908 | 0.007312 |
| 93 | MPEG1 | -1.78793 | 0.02087 |
| 94 | DAPL1 | -1.78668 | 0.03507 |
| 95 | SRP72 | -1.7842 | 0.029235 |
| 96 | IDH2 | -1.77861 | 0.021892 |
| 97 | PRMT1 | -1.77285 | 0.01291 |
| 98 | RNF130 | -1.77181 | 0.008062 |
| 99 | ITGB2 | -1.77028 | 0.016333 |
| 100 | AKAP12 | -1.7696 | 0.030402 |
| 101 | RABAC1 | -1.76234 | 0.012738 |
| 102 | CD74 | -1.75668 | 0.023471 |
| 103 | CRIP1 | -1.75625 | 0.033739 |
| 104 | NDUFB10 | -1.7491 | 0.028399 |
| 105 | SMAP1 | -1.74559 | 0.038203 |
| 106 | FBLN5 | -1.74396 | 0.019475 |
| 107 | ANP32B | -1.74137 | 0.023013 |
| 108 | CLC | -1.73631 | 0.017818 |
| 109 | RAC2 | -1.73444 | 0.026511 |
| 110 | NCKIPSD | -1.72309 | 0.049651 |
| 111 | RPS27 | -1.71718 | 0.047342 |
| 112 | ARPP19 | -1.7094 | 0.024799 |
| 113 | EIF5 | -1.70034 | 0.037265 |
| 114 | VAMP8 | -1.70008 | 0.038643 |
| 115 | RPS12 | -1.69112 | 0.034301 |
| 116 | HBB | -1.68915 | 0.036257 |
| 117 | COX16 | -1.68435 | 0.045558 |
| 118 | ITGB1BP1 | -1.67495 | 0.025799 |
| 119 | NQO2 | -1.67303 | 0.022631 |
| 120 | EVI2B | -1.67116 | 0.023821 |
| 121 | PECAM1 | -1.67063 | 0.034846 |
| 122 | NAIP | -1.65791 | 0.001631 |
| 123 | DRAP1 | -1.65722 | 0.006376 |
| 124 | DHRS9 | -1.65613 | 0.007742 |
| 125 | ACKR1 | -1.65238 | 0.008062 |
| 126 | C5AR1 | -1.64806 | 0.017112 |
| 127 | TPP1 | -1.64427 | 0.036765 |
| 128 | NCF2 | -1.64284 | 0.003834 |
| 129 | ID2 | -1.64266 | 0.044019 |
| 130 | CYBB | -1.63985 | 0.011947 |
| 131 | RPLP1 | -1.63335 | 0.039918 |
| 132 | C19orf70 | -1.63071 | 0.014915 |
| 133 | C19orf12 | -1.62341 | 0.027248 |
| 134 | FCN1 | -1.61956 | 0.003834 |
| 135 | ORC4 | -1.61565 | 0.032174 |
| 136 | CYGB | -1.60078 | 0.019233 |
| 137 | CDK2AP1 | -1.59346 | 0.043698 |
| 138 | PLAC8 | -1.5931 | 0.022585 |
| 139 | TSPAN15 | -1.5918 | 0.001631 |
| 140 | FCGR2A | -1.58851 | 0.007312 |
| 141 | WWC3 | -1.58353 | 0.007312 |
| 142 | NFIL3 | -1.58234 | 0.02674 |
| 143 | TRAPPC5 | -1.57818 | 0.022979 |
| 144 | COMMD6 | -1.57784 | 0.047224 |
| 145 | CD48 | -1.57749 | 0.02176 |
| 146 | PGAM2 | -1.57384 | 0.047508 |
| 147 | MYL6 | -1.5642 | 0.010273 |
| 148 | NDUFC1 | -1.56056 | 0.048106 |
| 149 | ALOX5AP | -1.55802 | 0.002161 |
| 150 | APOLD1 | -1.55594 | 0.04978 |
| 151 | TMEM256 | -1.55289 | 0.033901 |
| 152 | RNASE1 | -1.55163 | 0.014915 |
| 153 | ARAP3 | -1.54605 | 0.010273 |
| 154 | PID1 | -1.54375 | 0.010273 |
| 155 | FAU | -1.54237 | 0.02176 |
| 156 | PSMC3 | -1.53649 | 0.023013 |
| 157 | PYCARD | -1.53042 | 0.007825 |
| 158 | AP3S1 | -1.52767 | 0.041613 |
| 159 | SPOP | -1.52444 | 0.02508 |
| 160 | RRAD | -1.51894 | 0.028673 |
| 161 | POLB | -1.51609 | 0.0395 |
| 162 | RPS6 | -1.51224 | 0.038748 |
| 163 | F11R | -1.51005 | 0.028399 |
| 164 | COTL1 | -1.50657 | 0.003769 |
| 165 | GMDS | -1.49886 | 0.02399 |
| 166 | MRPL9 | -1.49743 | 0.034009 |
| 167 | ISOC2 | -1.49382 | 0.007742 |
| 168 | ALYREF | -1.49153 | 0.013796 |
| 169 | RRAGD | -1.48747 | 0.033737 |
| 170 | ANXA5 | -1.48585 | 0.029279 |
| 171 | SSR1 | -1.48549 | 0.048084 |
| 172 | CEBPD | -1.48375 | 0.026395 |
| 173 | UQCRC1 | -1.48375 | 0.02087 |
| 174 | NENF | -1.48078 | 0.046165 |
| 175 | NPC2 | -1.48067 | 0.000863 |
| 176 | CYBA | -1.47853 | 0.004159 |
| 177 | MTHFD2 | -1.47703 | 0.049094 |
| 178 | NDUFA11 | -1.47395 | 0.01817 |
| 179 | ID1 | -1.47303 | 0.009406 |
| 180 | RASIP1 | -1.47212 | 0.013569 |
| 181 | GABARAPL2 | -1.47186 | 0.036383 |
| 182 | FOXC2 | -1.47019 | 0.027248 |
| 183 | CRLF1 | -1.4651 | 0.033739 |
| 184 | LGALS1 | -1.46423 | 0.018557 |
| 185 | PGAM1 | -1.46343 | 0.005512 |
| 186 | TCEB2 | -1.46181 | 0.032004 |
| 187 | RPL36AL | -1.46169 | 0.046141 |
| 188 | PQBP1 | -1.45808 | 0.015522 |
| 189 | ERP44 | -1.45486 | 0.042766 |
| 190 | LYVE1 | -1.45324 | 0.003834 |
| 191 | PPP1R14B | -1.45021 | 0.027875 |
| 192 | ARF5 | -1.44463 | 0.001483 |
| 193 | RAB32 | -1.43722 | 0.010964 |
| 194 | RAP1B | -1.43497 | 0.03871 |
| 195 | BMP6 | -1.43456 | 0.043881 |
| 196 | CTBP1 | -1.43362 | 0.037265 |
| 197 | KPNB1 | -1.43318 | 0.012738 |
| 198 | CDK5 | -1.43205 | 0.032629 |
| 199 | RPS9 | -1.42835 | 0.03262 |
| 200 | SLIRP | -1.42193 | 0.033737 |
| 201 | GPR171 | -1.42151 | 0.02114 |
| 202 | SYNGR2 | -1.41997 | 0.02823 |
| 203 | C12orf57 | -1.41991 | 0.034846 |
| 204 | SFRP4 | -1.41966 | 0.030987 |
| 205 | KCTD21 | -1.41734 | 0.038186 |
| 206 | NOV | -1.41551 | 0.033582 |
| 207 | PSME1 | -1.4124 | 0.02087 |
| 208 | UBE2K | -1.41238 | 0.048345 |
| 209 | PHLDA3 | -1.40919 | 0.027898 |
| 210 | EDN1 | -1.39941 | 0.02638 |
| 211 | CHMP2A | -1.3965 | 0.022539 |
| 212 | RCAN1 | -1.39611 | 0.027809 |
| 213 | ALAS1 | -1.39569 | 0.035402 |
| 214 | IL13RA1 | -1.39515 | 0.045572 |
| 215 | PPT1 | -1.39299 | 0.049651 |
| 216 | LINC00493 | -1.39243 | 0.02849 |
| 217 | NT5C3B | -1.39211 | 0.027161 |
| 218 | CCL23 | -1.39198 | 0.035402 |
| 219 | RPL19 | -1.38941 | 0.037054 |
| 220 | PLAC9 | -1.38891 | 0.028336 |
| 221 | TMEM204 | -1.38866 | 0.027248 |
| 222 | COLGALT2 | -1.38701 | 0.032619 |
| 223 | MED25 | -1.37888 | 0.035805 |
| 224 | CSNK2B | -1.37841 | 0.040169 |
| 225 | ETS2 | -1.37627 | 0.016277 |
| 226 | ROMO1 | -1.37598 | 0.029517 |
| 227 | SLC6A8 | -1.37116 | 0.030974 |
| 228 | SLC25A5 | -1.37109 | 0.020805 |
| 229 | CD52 | -1.3675 | 0.047848 |
| 230 | KYNU | -1.36523 | 0.013219 |
| 231 | MAD2L2 | -1.36465 | 0.008279 |
| 232 | HN1 | -1.36247 | 0.019441 |
| 233 | NDRG1 | -1.36218 | 0.011947 |
| 234 | EFHD2 | -1.36117 | 0.037333 |
| 235 | MIF | -1.36008 | 0.003781 |
| 236 | PSMD5 | -1.35912 | 0.029509 |
| 237 | LAPTM5 | -1.35408 | 0.01201 |
| 238 | LINC00963 | -1.35337 | 0.003834 |
| 239 | HMOX2 | -1.34921 | 0.038019 |
| 240 | BLVRB | -1.34909 | 0.016277 |
| 241 | PSMB3 | -1.34832 | 0.048864 |
| 242 | YWHAH | -1.34779 | 0.032004 |
| 243 | GLIPR2 | -1.3462 | 0.007174 |
| 244 | FKBP11 | -1.34457 | 0.01201 |
| 245 | DNAJC15 | -1.34415 | 0.046585 |
| 246 | PIGT | -1.34414 | 0.006687 |
| 247 | COL1A2 | -1.34295 | 0.03998 |
| 248 | ITK | -1.34055 | 0.033721 |
| 249 | RPL18 | -1.33992 | 0.049731 |
| 250 | CSTB | -1.33531 | 0.014608 |
| 251 | ARPC2 | -1.33467 | 0.038473 |
| 252 | C1QC | -1.33358 | 0.007312 |
| 253 | GDI2 | -1.32782 | 0.032899 |
| 254 | PITPNA | -1.32608 | 0.009406 |
| 255 | CXCL17 | -1.32231 | 0.003039 |
| 256 | SLA | -1.32045 | 0.007825 |
| 257 | MID1IP1 | -1.31897 | 0.038748 |
| 258 | MTFMT | -1.3189 | 0.023183 |
| 259 | MTCH1 | -1.31887 | 0.020692 |
| 260 | MPG | -1.3153 | 0.036739 |
| 261 | ARCN1 | -1.3145 | 0.041139 |
| 262 | FTH1 | -1.30732 | 0.049684 |
| 263 | IFI6 | -1.30449 | 0.016385 |
| 264 | AQR | -1.30319 | 0.0395 |
| 265 | PQLC3 | -1.30169 | 0.030359 |
| 266 | C9orf3 | -1.29847 | 0.035126 |
| 267 | KRTCAP2 | -1.29642 | 0.013493 |
| 268 | RP11-736K20.5 | -1.29441 | 0.007825 |
| 269 | PGAM5 | -1.29038 | 0.000847 |
| 270 | AF007147 | -1.28489 | 0.039641 |
| 271 | GZMA | -1.28411 | 0.021248 |
| 272 | LIPA | -1.28312 | 0.038009 |
| 273 | TUFM | -1.28078 | 0.043154 |
| 274 | GLIS2 | -1.27826 | 0.01559 |
| 275 | MICAL2 | -1.2756 | 0.028993 |
| 276 | SNX20 | -1.27489 | 0.012529 |
| 277 | NFKBIA | -1.27354 | 0.036087 |
| 278 | NT5DC4 | -1.2726 | 0.005512 |
| 279 | DENND1A | -1.26954 | 0.010273 |
| 280 | GAP43 | -1.26795 | 0.036399 |
| 281 | HCG18 | -1.26464 | 0.017462 |
| 282 | PGD | -1.26457 | 0.025052 |
| 283 | NOSIP | -1.26455 | 0.032369 |
| 284 | CLEC10A | -1.26419 | 0.004873 |
| 285 | CSF2RB | -1.25932 | 0.025799 |
| 286 | CPNE2 | -1.259 | 0.022585 |
| 287 | FAM127A | -1.25542 | 0.028707 |
| 288 | ATXN10 | -1.25472 | 0.048718 |
| 289 | ZNHIT1 | -1.25374 | 0.00716 |
| 290 | SPI1 | -1.25333 | 0.022748 |
| 291 | ARHGEF10 | -1.25326 | 0.020987 |
| 292 | LRR1 | -1.25258 | 0.030987 |
| 293 | TRADD | -1.25081 | 0.025799 |
| 294 | DCTN1 | -1.24931 | 0.017774 |
| 295 | BID | -1.24928 | 0.025564 |
| 296 | MRPL41 | -1.24734 | 0.00563 |
| 297 | LITAF | -1.24667 | 0.017656 |
| 298 | UBE2L3 | -1.23985 | 0.023013 |
| 299 | SRRT | -1.23886 | 0.029143 |
| 300 | C1orf54 | -1.23859 | 0.00585 |
| 301 | MT2A | -1.2367 | 0.044896 |
| 302 | WBSCR22 | -1.23415 | 0.018186 |
| 303 | TXNL4A | -1.23262 | 0.008062 |
| 304 | C10orf11 | -1.23001 | 0.044701 |
| 305 | HSD17B11 | -1.22857 | 0.037265 |
| 306 | DDIT4 | -1.22484 | 0.007349 |
| 307 | PSMG3 | -1.22476 | 0.016189 |
| 308 | RPL7AL2 | -1.22044 | 0.015522 |
| 309 | LAMTOR4 | -1.21955 | 0.026778 |
| 310 | TMEM171 | -1.21743 | 0.003632 |
| 311 | HRAS | -1.21717 | 0.04188 |
| 312 | CD37 | -1.21676 | 0.034786 |
| 313 | GNAI2 | -1.21652 | 0.024489 |
| 314 | NKAIN1 | -1.21591 | 0.011397 |
| 315 | ATP8B4 | -1.21036 | 0.043698 |
| 316 | LST1 | -1.2093 | 0.006229 |
| 317 | SLC25A6 | -1.20775 | 0.007825 |
| 318 | VPS28 | -1.20647 | 0.041471 |
| 319 | CCT7 | -1.20517 | 0.036399 |
| 320 | PILRA | -1.20474 | 0.01201 |
| 321 | UBE2L6 | -1.20402 | 0.048345 |
| 322 | CTDSP1 | -1.20279 | 0.040783 |
| 323 | FBLN1 | -1.20007 | 0.043402 |
| 324 | PADI4 | -1.19833 | 0.026071 |
| 325 | SMCR8 | -1.19787 | 0.039087 |
| 326 | C2orf49 | -1.19598 | 0.035221 |
| 327 | FMO3 | -1.19471 | 0.034298 |
| 328 | HCK | -1.19322 | 0.015149 |
| 329 | PRELP | -1.1914 | 0.040552 |
| 330 | SIRPB2 | -1.18947 | 0.007742 |
| 331 | NXPE4 | -1.18763 | 0.004159 |
| 332 | GZMH | -1.18598 | 0.010555 |
| 333 | PRDX6 | -1.1858 | 0.048639 |
| 334 | QPCT | -1.18465 | 0.040792 |
| 335 | MAST1 | -1.184 | 0.012738 |
| 336 | IGFBP2 | -1.1828 | 0.031068 |
| 337 | GSDMD | -1.18205 | 0.02087 |
| 338 | SF3A3 | -1.18143 | 0.048437 |
| 339 | RNASET2 | -1.18126 | 0.017183 |
| 340 | TBXAS1 | -1.17887 | 0.000863 |
| 341 | MRPL10 | -1.17862 | 0.027875 |
| 342 | RP11-524D16__A.3 | -1.1786 | 0.004469 |
| 343 | COPRS | -1.17833 | 0.01937 |
| 344 | C4orf48 | -1.17832 | 0.049499 |
| 345 | FCER1G | -1.17557 | 0.016277 |
| 346 | AAED1 | -1.17523 | 0.039063 |
| 347 | ZNF667-AS1 | -1.16876 | 0.017809 |
| 348 | CLIC1 | -1.16625 | 0.004461 |
| 349 | RPA1 | -1.16429 | 0.018044 |
| 350 | CD163 | -1.16318 | 0.011386 |
| 351 | BHLHE22 | -1.15749 | 0.033175 |
| 352 | P2RY13 | -1.15699 | 0.024756 |
| 353 | PSMA7 | -1.15626 | 0.007825 |
| 354 | TICAM1 | -1.15422 | 0.022677 |
| 355 | FAM101B | -1.15403 | 0.048437 |
| 356 | RGS10 | -1.15139 | 0.010689 |
| 357 | STMN2 | -1.15098 | 0.025392 |
| 358 | NDUFS7 | -1.14939 | 0.013219 |
| 359 | FTO | -1.14892 | 0.028812 |
| 360 | RP1-8B22.1 | -1.14564 | 0.000535 |
| 361 | RAB13 | -1.14561 | 0.028596 |
| 362 | IL1R1 | -1.14315 | 0.044595 |
| 363 | ITGAM | -1.14142 | 0.004873 |
| 364 | COMMD3 | -1.13925 | 0.036326 |
| 365 | ECI1 | -1.1375 | 0.042635 |
| 366 | RBX1 | -1.13608 | 0.042979 |
| 367 | ENO3 | -1.13326 | 0.032004 |
| 368 | PGM5 | -1.13314 | 0.042635 |
| 369 | METRNL | -1.132 | 0.025415 |
| 370 | S100P | -1.1292 | 0.030918 |
| 371 | CCM2 | -1.12832 | 0.048774 |
| 372 | TSTD1 | -1.1269 | 0.033428 |
| 373 | SERP1 | -1.12486 | 0.012738 |
| 374 | MZB1 | -1.12433 | 0.035357 |
| 375 | CDKN1A | -1.12411 | 0.038384 |
| 376 | GZMB | -1.12315 | 0.027875 |
| 377 | OOSP2 | -1.12198 | 0.007312 |
| 378 | FZD4 | -1.12189 | 0.035825 |
| 379 | CSF3R | -1.11968 | 0.021248 |
| 380 | GNAQ | -1.11738 | 0.014308 |
| 381 | SNAPC4 | -1.11641 | 0.038965 |
| 382 | ZMIZ2 | -1.11637 | 0.004461 |
| 383 | PIK3R3 | -1.11532 | 0.030022 |
| 384 | FOLR2 | -1.11491 | 0.012153 |
| 385 | ADAM32 | -1.11383 | 0.037551 |
| 386 | MYD88 | -1.11344 | 0.025952 |
| 387 | SLC1A5 | -1.1132 | 0.044595 |
| 388 | LINC00354 | -1.11141 | 0.009217 |
| 389 | VAMP3 | -1.11052 | 0.022721 |
| 390 | ABCB11 | -1.10915 | 0.003039 |
| 391 | VSIG4 | -1.10768 | 0.011481 |
| 392 | SIVA1 | -1.10418 | 0.003834 |
| 393 | ABCB7 | -1.10322 | 0.027755 |
| 394 | ENTPD1 | -1.10311 | 0.006868 |
| 395 | MSL3 | -1.10157 | 0.039918 |
| 396 | PRDM4 | -1.10062 | 0.038981 |
| 397 | P2RY8 | -1.09987 | 0.013429 |
| 398 | CDK9 | -1.09916 | 0.004025 |
| 399 | DDX23 | -1.0984 | 0.02114 |
| 400 | LYN | -1.09816 | 0.022631 |
| 401 | GPX4 | -1.09802 | 0.016277 |
| 402 | PRADC1 | -1.09799 | 0.012443 |
| 403 | ARPC1A | -1.09743 | 0.024956 |
| 404 | CD3D | -1.0965 | 0.026395 |
| 405 | SQRDL | -1.09507 | 0.040464 |
| 406 | PREB | -1.09397 | 0.03085 |
| 407 | CTC-550B14.7 | -1.0922 | 0.003834 |
| 408 | MRPL12 | -1.09122 | 0.02399 |
| 409 | SPAG4 | -1.09085 | 0.014915 |
| 410 | LOC100505978 | -1.08939 | 0.004461 |
| 411 | CCDC170 | -1.08873 | 0.007825 |
| 412 | SHC1 | -1.08748 | 0.039663 |
| 413 | LRRC32 | -1.08707 | 0.013878 |
| 414 | PLA2G7 | -1.08685 | 0.016385 |
| 415 | ABCC5 | -1.08659 | 0.017219 |
| 416 | LOC101927722 | -1.08655 | 0.004025 |
| 417 | CTD-3080P12.3 | -1.08413 | 0.004053 |
| 418 | TCF21 | -1.08211 | 0.006487 |
| 419 | CSNK2A2 | -1.07989 | 0.017945 |
| 420 | PDCD6 | -1.07832 | 0.004159 |
| 421 | PFDN1 | -1.0783 | 0.008609 |
| 422 | SAP18 | -1.07637 | 0.034019 |
| 423 | EDNRB | -1.0751 | 0.038954 |
| 424 | MRPL43 | -1.07488 | 0.013963 |
| 425 | FARSA | -1.07229 | 0.041387 |
| 426 | RPPH1 | -1.07112 | 0.002216 |
| 427 | C1orf162 | -1.07001 | 0.007312 |
| 428 | EWSR1 | -1.0676 | 0.004873 |
| 429 | C1orf116 | -1.06639 | 0.003769 |
| 430 | EEF1D | -1.06589 | 0.040283 |
| 431 | ARRDC2 | -1.0654 | 0.03281 |
| 432 | AFMID | -1.06506 | 0.034846 |
| 433 | PHB | -1.06501 | 0.017864 |
| 434 | TOR1B | -1.06462 | 0.033939 |
| 435 | ANXA6 | -1.06458 | 0.044701 |
| 436 | SYNGR1 | -1.06224 | 0.014998 |
| 437 | VOPP1 | -1.0607 | 0.011499 |
| 438 | MRPS16 | -1.06069 | 0.01224 |
| 439 | HOXC10 | -1.05906 | 0.034778 |
| 440 | GUSB | -1.05854 | 0.015034 |
| 441 | PRCP | -1.05756 | 0.011601 |
| 442 | CHURC1 | -1.0563 | 0.044597 |
| 443 | LOC100129034 | -1.05419 | 0.011451 |
| 444 | MICALCL | -1.05201 | 0.004053 |
| 445 | CD47 | -1.04827 | 0.02176 |
| 446 | SCN4B | -1.04632 | 0.030987 |
| 447 | FAM168A | -1.04632 | 0.029316 |
| 448 | AKT3 | -1.04558 | 0.016258 |
| 449 | RGS3 | -1.04389 | 0.039482 |
| 450 | ALDH16A1 | -1.04364 | 0.012164 |
| 451 | MKNK2 | -1.0417 | 0.017759 |
| 452 | ZNF17 | -1.03991 | 0.002216 |
| 453 | TOMM22 | -1.03952 | 0.035894 |
| 454 | ACAD9 | -1.03927 | 0.033859 |
| 455 | CELA2B | -1.03876 | 0.031336 |
| 456 | SCUBE2 | -1.03588 | 0.009406 |
| 457 | BCAT1 | -1.03445 | 0.049665 |
| 458 | C16orf13 | -1.03237 | 0.030846 |
| 459 | S100A6 | -1.03209 | 0.011569 |
| 460 | CREM | -1.03165 | 0.049651 |
| 461 | STX10 | -1.03115 | 0.024956 |
| 462 | PSMB6 | -1.02956 | 0.036257 |
| 463 | CUEDC2 | -1.02955 | 0.007825 |
| 464 | IP6K2 | -1.02819 | 0.007825 |
| 465 | CBFB | -1.02574 | 0.037333 |
| 466 | LOC285422 | -1.02563 | 0.026395 |
| 467 | LOC100128840 | -1.02499 | 0.000847 |
| 468 | ATP2B4 | -1.02452 | 0.044033 |
| 469 | CCDC37-AS1 | -1.02226 | 0.00736 |
| 470 | ENO1 | -1.02074 | 0.043483 |
| 471 | AEBP1 | -1.01874 | 0.026395 |
| 472 | POU5F1P3 | -1.0187 | 0.003834 |
| 473 | FCGR2C | -1.01816 | 0.033829 |
| 474 | CD2 | -1.01776 | 0.040283 |
| 475 | UBL7 | -1.01733 | 0.048772 |
| 476 | NRSN1 | -1.01665 | 0.00991 |
| 477 | PEBP1 | -1.01487 | 0.030693 |
| 478 | ZNF837 | -1.01475 | 0.005701 |
| 479 | SMAP2 | -1.01312 | 0.021373 |
| 480 | C6orf48 | -1.01269 | 0.010273 |
| 481 | HNRNPF | -1.01261 | 0.046935 |
| 482 | IMP3 | -1.01054 | 0.023013 |
| 483 | CCHCR1 | -1.00991 | 0.023641 |
| 484 | RASGEF1C | -1.00867 | 0.008934 |
| 485 | LILRB4 | -1.00675 | 0.02114 |
| 486 | PREX1 | -1.00666 | 0.021299 |
| 487 | LIX1L | -1.00629 | 0.03507 |
| 488 | GJA4 | -1.00438 | 0.021553 |
| 489 | CTSB | -1.00414 | 0.026157 |
| 490 | RILPL2 | -1.00409 | 0.028842 |
| 491 | ARMC12 | -1.00359 | 0.001631 |
| 492 | IGFLR1 | -1.00274 | 0.00852 |
| 493 | TMEM258 | -1.00259 | 0.033237 |
| 494 | TAT | -1.00035 | 0.00266 |
| 495 | MPHOSPH8 | 1.001654 | 0.016258 |
| 496 | TRIM55 | 1.002036 | 0.017749 |
| 497 | ZIC4 | 1.002931 | 0.007742 |
| 498 | USP24 | 1.003467 | 0.020092 |
| 499 | CNGA1 | 1.003879 | 0.003834 |
| 500 | ASB4 | 1.003895 | 0.010677 |
| 501 | LRIG2 | 1.004139 | 0.00563 |
| 502 | UBA3 | 1.007032 | 0.017818 |
| 503 | CYB561 | 1.00744 | 0.015522 |
| 504 | LINC00880 | 1.011007 | 0.020092 |
| 505 | TTC30A | 1.011475 | 0.0166 |
| 506 | MYLK3 | 1.01269 | 0.027677 |
| 507 | RASSF3 | 1.014543 | 0.014959 |
| 508 | SNX22 | 1.015019 | 0.02176 |
| 509 | LARS | 1.015306 | 0.032003 |
| 510 | LAMC1 | 1.018588 | 0.020014 |
| 511 | DNAJC9-AS1 | 1.020129 | 0.007825 |
| 512 | NLN | 1.020632 | 0.033859 |
| 513 | RP4-714D9.5 | 1.023663 | 0.037054 |
| 514 | ARNT2 | 1.025983 | 0.040792 |
| 515 | DNM1P46 | 1.026363 | 0.011947 |
| 516 | ACSS1 | 1.026725 | 0.030918 |
| 517 | GFM1 | 1.026916 | 0.022631 |
| 518 | YWHAE | 1.028016 | 0.005512 |
| 519 | TFAP2C | 1.030431 | 0.041676 |
| 520 | MSS51 | 1.030653 | 0.018644 |
| 521 | EXOC6B | 1.03168 | 0.004159 |
| 522 | TXNDC15 | 1.03289 | 0.009162 |
| 523 | SNX14 | 1.03466 | 0.033061 |
| 524 | ARGLU1 | 1.036454 | 0.026117 |
| 525 | ITGA6 | 1.0372 | 0.034778 |
| 526 | SPOCK2 | 1.037367 | 0.033741 |
| 527 | CHTOP | 1.03778 | 0.009143 |
| 528 | PHF10 | 1.037992 | 0.027209 |
| 529 | RNF212B | 1.038148 | 0.004469 |
| 530 | RP11-399O19.9 | 1.039325 | 0.047823 |
| 531 | FZD1 | 1.039333 | 0.010902 |
| 532 | DLG5-AS1 | 1.041137 | 0.03423 |
| 533 | RMND5A | 1.042742 | 0.009738 |
| 534 | RP11-119F7.5 | 1.044222 | 0.047511 |
| 535 | MACF1 | 1.045303 | 0.011601 |
| 536 | OR2F2 | 1.045469 | 0.014229 |
| 537 | HSD17B12 | 1.045831 | 0.008863 |
| 538 | KLK7 | 1.046089 | 0.040676 |
| 539 | HSF2 | 1.050207 | 0.045544 |
| 540 | TUBGCP5 | 1.053437 | 0.018055 |
| 541 | CABP4 | 1.05402 | 0.012039 |
| 542 | PRDM11 | 1.054882 | 0.039663 |
| 543 | NPHP1 | 1.055167 | 0.048084 |
| 544 | TOP3B | 1.05704 | 0.030528 |
| 545 | PIFO | 1.058444 | 0.02114 |
| 546 | NPNT | 1.05895 | 0.038203 |
| 547 | HIBCH | 1.059624 | 0.033428 |
| 548 | GATA4 | 1.061175 | 0.013219 |
| 549 | TRAF3IP2 | 1.062388 | 0.008217 |
| 550 | FOXA3 | 1.065391 | 0.003769 |
| 551 | WWP1 | 1.066358 | 0.029517 |
| 552 | CPEB4 | 1.067004 | 0.046548 |
| 553 | EPC1 | 1.067032 | 0.004461 |
| 554 | ACAD8 | 1.068939 | 0.011808 |
| 555 | RP11-672L10.6 | 1.069072 | 0.017818 |
| 556 | TMEM179 | 1.069933 | 0.046301 |
| 557 | TMEM182 | 1.073153 | 0.01258 |
| 558 | ZADH2 | 1.073979 | 0.01705 |
| 559 | ING3 | 1.073998 | 0.008068 |
| 560 | KAT7 | 1.074418 | 0.02176 |
| 561 | SLITRK6 | 1.075593 | 0.045637 |
| 562 | SEPSECS-AS1 | 1.07613 | 0.044168 |
| 563 | CDON | 1.078361 | 0.019053 |
| 564 | RAC3 | 1.080707 | 0.037265 |
| 565 | LOC101929757 | 1.081503 | 0.018644 |
| 566 | PRIMA1 | 1.082024 | 0.043165 |
| 567 | DOCK4 | 1.083546 | 0.025926 |
| 568 | DSG2 | 1.084838 | 0.04628 |
| 569 | IKBKAP | 1.08766 | 0.004649 |
| 570 | CPS1-IT1 | 1.087842 | 0.014308 |
| 571 | LOC100652824 | 1.087929 | 0.004025 |
| 572 | MPP5 | 1.088047 | 0.020583 |
| 573 | FBXO10 | 1.088395 | 0.017462 |
| 574 | PDE4D | 1.089053 | 0.030918 |
| 575 | EIF2AK3 | 1.089071 | 0.03983 |
| 576 | MRPS22 | 1.091576 | 0.00991 |
| 577 | PRAME | 1.092699 | 0.013796 |
| 578 | LOC101927085 | 1.093264 | 0.034414 |
| 579 | LOC646762 | 1.093926 | 0.011601 |
| 580 | EMX1 | 1.095553 | 0.010273 |
| 581 | NHSL1 | 1.095749 | 0.018974 |
| 582 | PIM3 | 1.098582 | 0.007349 |
| 583 | LOC643711 | 1.098755 | 0.00266 |
| 584 | PDE7A | 1.101598 | 0.025538 |
| 585 | RASGRP3 | 1.102577 | 0.017788 |
| 586 | RNF183 | 1.102905 | 0.005512 |
| 587 | PXDNL | 1.103191 | 0.03871 |
| 588 | HMGB2 | 1.103715 | 0.033403 |
| 589 | FBXO32 | 1.103728 | 0.01705 |
| 590 | HELZ2 | 1.103863 | 0.032119 |
| 591 | MAGOHB | 1.106666 | 0.010388 |
| 592 | RNF19A | 1.106704 | 0.008618 |
| 593 | LRRN1 | 1.107042 | 0.045282 |
| 594 | ARHGAP44 | 1.109305 | 0.039285 |
| 595 | INTS6 | 1.109667 | 0.010964 |
| 596 | STEAP2 | 1.110346 | 0.04071 |
| 597 | KCTD1 | 1.1121 | 0.008068 |
| 598 | LOC101929622 | 1.113214 | 0.032198 |
| 599 | KIF5B | 1.115193 | 0.040792 |
| 600 | MEG9 | 1.116452 | 0.032849 |
| 601 | PPP1CB | 1.116499 | 0.012738 |
| 602 | TUFT1 | 1.122077 | 0.018644 |
| 603 | LINC00938 | 1.122106 | 0.044691 |
| 604 | LOC101928955 | 1.123212 | 0.015843 |
| 605 | CA5BP1 | 1.125495 | 0.025483 |
| 606 | LOC338620 | 1.125962 | 0.045282 |
| 607 | EPHA2 | 1.125967 | 0.015335 |
| 608 | MLH3 | 1.126026 | 0.02849 |
| 609 | LOC100996263 | 1.133112 | 0.004413 |
| 610 | CRIM1 | 1.133337 | 0.01961 |
| 611 | RSF1 | 1.134597 | 0.027898 |
| 612 | FAT4 | 1.135324 | 0.028336 |
| 613 | LOC101928446 | 1.135736 | 0.014308 |
| 614 | C20orf78 | 1.136353 | 0.009217 |
| 615 | LOC101928288 | 1.136838 | 0.018644 |
| 616 | MIR302B | 1.138045 | 0.046585 |
| 617 | GRIK3 | 1.138166 | 0.044597 |
| 618 | RINT1 | 1.139187 | 0.021166 |
| 619 | BBS4 | 1.140854 | 0.043154 |
| 620 | EDNRA | 1.141805 | 0.00852 |
| 621 | TMEM63C | 1.142017 | 0.007825 |
| 622 | PTCD2 | 1.142754 | 0.009738 |
| 623 | E2F8 | 1.143105 | 0.023203 |
| 624 | HFE2 | 1.143308 | 0.008454 |
| 625 | VWC2 | 1.143465 | 0.018202 |
| 626 | APOOL | 1.144274 | 0.00582 |
| 627 | ALG13 | 1.145201 | 0.040283 |
| 628 | RP11-1069G10.1 | 1.148243 | 0.007312 |
| 629 | ANKRA2 | 1.14843 | 0.037897 |
| 630 | IGSF5 | 1.151724 | 0.031336 |
| 631 | RBM11 | 1.152191 | 0.007825 |
| 632 | CNIH1 | 1.152748 | 0.016736 |
| 633 | NSUN7 | 1.155086 | 0.016926 |
| 634 | STRIP2 | 1.155562 | 0.010495 |
| 635 | KALRN | 1.158111 | 0.010555 |
| 636 | BMPR1B | 1.159624 | 0.029654 |
| 637 | ATMIN | 1.15968 | 0.010428 |
| 638 | ENOSF1 | 1.160862 | 0.030528 |
| 639 | ACADSB | 1.166701 | 0.033403 |
| 640 | ABCD3 | 1.167604 | 0.012232 |
| 641 | NBEA | 1.16865 | 0.0071 |
| 642 | GPR39 | 1.170178 | 0.023821 |
| 643 | LINC00905 | 1.172017 | 0.045282 |
| 644 | ZDHHC17 | 1.172075 | 0.038384 |
| 645 | RANBP6 | 1.172711 | 0.009738 |
| 646 | ARID1B | 1.176134 | 0.0345 |
| 647 | TMEM33 | 1.17659 | 0.02002 |
| 648 | ARFGEF2 | 1.176922 | 0.007259 |
| 649 | BC039487 | 1.177495 | 0.042766 |
| 650 | KDM7A | 1.180451 | 0.027065 |
| 651 | KIF1B | 1.181467 | 0.007742 |
| 652 | TCEAL7 | 1.181937 | 0.041765 |
| 653 | RORB | 1.182739 | 0.042635 |
| 654 | MGC12488 | 1.184732 | 0.015522 |
| 655 | PIN1P1 | 1.189962 | 0.038384 |
| 656 | SLC3A1 | 1.196009 | 0.022445 |
| 657 | TTN | 1.197138 | 0.005512 |
| 658 | PRNP | 1.198634 | 0.025564 |
| 659 | ARFGAP3 | 1.199318 | 0.019402 |
| 660 | PPARGC1B | 1.201091 | 0.015232 |
| 661 | MED23 | 1.20158 | 0.004461 |
| 662 | LRP4 | 1.203929 | 0.013267 |
| 663 | MCC | 1.205852 | 0.027349 |
| 664 | HCG4 | 1.207654 | 0.025392 |
| 665 | DYRK2 | 1.208403 | 0.023471 |
| 666 | DMD | 1.213259 | 0.020119 |
| 667 | LOC101928687 | 1.215562 | 0.018974 |
| 668 | FBXW12 | 1.218552 | 0.032646 |
| 669 | LOC728805 | 1.220311 | 0.046585 |
| 670 | PRKAA2 | 1.224517 | 0.044774 |
| 671 | NECAP1 | 1.227693 | 0.000847 |
| 672 | ADAMTS8 | 1.228505 | 0.038019 |
| 673 | REST | 1.232337 | 0.040824 |
| 674 | SCN2B | 1.233211 | 0.015411 |
| 675 | ZNF697 | 1.239464 | 0.005701 |
| 676 | GP5 | 1.242995 | 0.01355 |
| 677 | NKX3-1 | 1.243256 | 0.009143 |
| 678 | NCKAP5 | 1.244102 | 0.030813 |
| 679 | BNC2 | 1.244419 | 0.029562 |
| 680 | TCEAL2 | 1.245775 | 0.046857 |
| 681 | UNC45B | 1.246292 | 0.005701 |
| 682 | STOX2 | 1.246326 | 0.02399 |
| 683 | BMP2K | 1.246605 | 0.018958 |
| 684 | ND6 | 1.246979 | 0.031965 |
| 685 | RYR2 | 1.249656 | 0.007825 |
| 686 | 44627 | 1.249954 | 0.015522 |
| 687 | TRIM10 | 1.250197 | 0.047224 |
| 688 | PLEK2 | 1.250349 | 0.013593 |
| 689 | RP1-142L7.8 | 1.25195 | 0.017462 |
| 690 | TOB1 | 1.252782 | 0.033737 |
| 691 | SLCO4C1 | 1.261964 | 0.023203 |
| 692 | CAPS2 | 1.26425 | 0.049507 |
| 693 | CC2D2B | 1.264295 | 0.00852 |
| 694 | DPP4 | 1.265959 | 0.039063 |
| 695 | SOX9 | 1.267933 | 0.049943 |
| 696 | ADAMTSL4 | 1.275332 | 0.008937 |
| 697 | MGC70870 | 1.276622 | 0.034846 |
| 698 | HCN4 | 1.27916 | 0.004025 |
| 699 | NKTR | 1.280855 | 0.013796 |
| 700 | LOC100130964 | 1.28149 | 0.042696 |
| 701 | EIF4G3 | 1.281508 | 0.005512 |
| 702 | GRIN2A | 1.281845 | 0.011019 |
| 703 | COL6A6 | 1.283799 | 0.029143 |
| 704 | SUSD4 | 1.284572 | 0.022445 |
| 705 | SYT13 | 1.293979 | 0.020119 |
| 706 | UBR3 | 1.297338 | 0.032119 |
| 707 | LINC00355 | 1.29846 | 0.030022 |
| 708 | SAMD12 | 1.299798 | 0.048718 |
| 709 | SHB | 1.300945 | 0.02849 |
| 710 | HEG1 | 1.300956 | 0.003981 |
| 711 | GPSM2 | 1.302984 | 0.024015 |
| 712 | SPHKAP | 1.30509 | 0.024956 |
| 713 | C9orf173 | 1.305692 | 0.044581 |
| 714 | CLDN4 | 1.306316 | 0.033227 |
| 715 | NR3C2 | 1.312051 | 0.004156 |
| 716 | PCDHB9 | 1.313905 | 0.0071 |
| 717 | STT3B | 1.315622 | 0.046968 |
| 718 | NPAS3 | 1.31568 | 0.029669 |
| 719 | LOC100288675 | 1.318991 | 0.01705 |
| 720 | NDFIP2 | 1.324121 | 0.034778 |
| 721 | LOC100287290 | 1.324778 | 0.042722 |
| 722 | ALX3 | 1.327491 | 0.03507 |
| 723 | RP11-141M1.1 | 1.329886 | 0.022721 |
| 724 | SLC39A8 | 1.33104 | 0.033546 |
| 725 | PRSS36 | 1.336604 | 0.016049 |
| 726 | CDH13 | 1.346439 | 0.032899 |
| 727 | SUCO | 1.348313 | 0.033859 |
| 728 | STK38L | 1.348899 | 0.01948 |
| 729 | MATR3 | 1.356547 | 0.045673 |
| 730 | PPIL1 | 1.356566 | 0.007686 |
| 731 | XK | 1.358272 | 0.01161 |
| 732 | TDRP | 1.370324 | 0.010273 |
| 733 | KANK1 | 1.372681 | 0.00211 |
| 734 | KLHL41 | 1.379026 | 0.025052 |
| 735 | DNAJC16 | 1.38073 | 0.001631 |
| 736 | CAP2 | 1.385244 | 0.009253 |
| 737 | FTCDNL1 | 1.388284 | 0.038047 |
| 738 | LOC100507477 | 1.392331 | 0.018644 |
| 739 | LINC00839 | 1.394294 | 0.036257 |
| 740 | TAF1A | 1.400335 | 0.034301 |
| 741 | PIP5K1B | 1.401441 | 0.003834 |
| 742 | LRRC7 | 1.403266 | 0.008062 |
| 743 | ATP8A1 | 1.410147 | 0.033149 |
| 744 | SLC26A9 | 1.413073 | 0.0166 |
| 745 | GLG1 | 1.413459 | 0.033901 |
| 746 | GPR1 | 1.413965 | 0.036599 |
| 747 | MGARP | 1.416895 | 0.011052 |
| 748 | LOC101926918 | 1.418406 | 0.005512 |
| 749 | PCNXL4 | 1.421282 | 0.033134 |
| 750 | LOC100131303 | 1.423742 | 0.001631 |
| 751 | LINC00593 | 1.425188 | 0.017708 |
| 752 | NFATC2IP | 1.430841 | 0.027248 |
| 753 | VPS13A | 1.43138 | 0.038118 |
| 754 | FAM169A | 1.431923 | 0.01384 |
| 755 | METTL21A | 1.433428 | 0.046585 |
| 756 | ZBED9 | 1.441255 | 0.004873 |
| 757 | LINC00622 | 1.441781 | 0.022631 |
| 758 | BAI2 | 1.442093 | 0.008329 |
| 759 | MEIG1 | 1.442839 | 0.009526 |
| 760 | CHIC1 | 1.446902 | 0.016333 |
| 761 | PTPRZ1 | 1.452991 | 0.025779 |
| 762 | LINC00261 | 1.458237 | 0.046585 |
| 763 | COL4A6 | 1.461302 | 0.020119 |
| 764 | PRSS12 | 1.4699 | 0.046902 |
| 765 | NSF | 1.480878 | 0.008994 |
| 766 | KDR | 1.482142 | 0.013219 |
| 767 | NNT-AS1 | 1.484256 | 0.021248 |
| 768 | MMP24 | 1.486984 | 0.011601 |
| 769 | LINC00673 | 1.491936 | 0.033737 |
| 770 | MFN1 | 1.50626 | 0.008754 |
| 771 | CTD-2528L19.6 | 1.516712 | 0.011601 |
| 772 | GPM6A | 1.534461 | 0.031732 |
| 773 | LOC100129455 | 1.53542 | 0.039968 |
| 774 | LOC100506498 | 1.552169 | 0.037265 |
| 775 | ERBB4 | 1.556892 | 0.008454 |
| 776 | RP11-157B13.7 | 1.559636 | 0.016694 |
| 777 | PWAR6 | 1.564002 | 0.023607 |
| 778 | USP32P2 | 1.566309 | 0.007623 |
| 779 | HOOK1 | 1.569666 | 0.013796 |
| 780 | CGN | 1.574613 | 0.034253 |
| 781 | WIF1 | 1.586295 | 0.009738 |
| 782 | RP11-382B18.4 | 1.587078 | 0.043165 |
| 783 | BRINP3 | 1.590219 | 0.033198 |
| 784 | TAS2R13 | 1.590803 | 0.007742 |
| 785 | SLC27A6 | 1.598282 | 0.00518 |
| 786 | CCNB3 | 1.603894 | 0.007742 |
| 787 | CPNE4 | 1.609278 | 0.047511 |
| 788 | SMIM17 | 1.619409 | 0.037646 |
| 789 | ANK3 | 1.625752 | 0.000847 |
| 790 | FGF12 | 1.628728 | 0.035402 |
| 791 | SNORA72 | 1.628867 | 0.009406 |
| 792 | NAP1L2 | 1.630164 | 0.014959 |
| 793 | IBA57-AS1 | 1.638153 | 0.007825 |
| 794 | FAM71C | 1.642697 | 0.036506 |
| 795 | LOC101930415 | 1.65374 | 0.03085 |
| 796 | KRTAP17-1 | 1.68731 | 0.037993 |
| 797 | SBSPON | 1.695117 | 0.018137 |
| 798 | CAPN6 | 1.698222 | 0.011288 |
| 799 | AQP4 | 1.70179 | 0.046857 |
| 800 | MYO5B | 1.713786 | 0.023013 |
| 801 | OTOGL | 1.724812 | 0.027473 |
| 802 | PDE3A | 1.730113 | 0.009217 |
| 803 | ETNPPL | 1.760876 | 0.048084 |
| 804 | MGAT4C | 1.786009 | 0.008994 |
| 805 | LINC00326 | 1.828348 | 0.009526 |
| 806 | DPY19L1P1 | 1.839938 | 0.032174 |
| 807 | KCNJ3 | 1.850444 | 0.004051 |
| 808 | SLC35G3 | 1.859634 | 0.001631 |
| 809 | NEB | 1.886325 | 0.010689 |
| 810 | SOSTDC1 | 1.941516 | 0.022151 |
| 811 | FLRT3 | 2.019075 | 0.043683 |
| 812 | FAM83B | 2.107116 | 0.002161 |
| 813 | LINC01018 | 2.172572 | 0.015955 |
| 814 | RNF128 | 2.177052 | 0.00736 |
| 815 | PEX1 | 2.240483 | 0.007973 |
| 816 | DNER | 2.247172 | 0.035825 |
| 817 | BNC1 | 2.379249 | 0.040283 |
| 818 | NMU | 2.830385 | 0.012305 |
| Combined arrhythmia associated targets dataset in LA-PV junction and LAA samples (932 DEGs) | | | |
| AAED1,ABCB11,ABCB7,ABCC5,ABCD2,ABCD3,ACAD8,ACAD9,ACADSB,ACKR1,ACSL4,ACSS1,ADAM32,ADAMTS8,ADAMTSL4,AEBP1,AF007147,AFMID,AKAP12,AKT3,ALAS1,ALDH16A1,ALG1,ALG13,ALOX5AP,ALX3,ALYREF,ANK3,ANKRA2,ANP32B,ANXA5,ANXA6,AP3S1,APOLD1,APOOL,AQP4,AQR,ARAP3,ARCN1,ARF5,ARFGAP3,ARFGEF2,ARGLU1,ARHGAP44,ARHGEF10,ARID1B,ARMC12,ARNT2,ARPC1A,ARPC2,ARPP19,ARRB2,ARRDC2,ASB4,ATMIN,ATP13A3,ATP1B3,ATP2B4,ATP5G2,ATP8A1,ATP8B4,ATXN10,AX748292,BAI2,BANF1,BBS4,BC039487,BC113958,BCAT1,BCL2A1,BCL2L13,BEX2,BHLHE22,BID,BLVRB,BMP2K,BMP6,BMPR1B,BNC1,BNC2,BOD1,BRINP3,BTG2,C10orf11,C12orf57,C15orf37,C16orf13,C18orf54,C19orf10,C19orf12,C19orf70,C1orf116,C1orf162,C1orf54,C1QA,C1QB,C1QC,C20orf78,C2orf49,C4orf48,C5AR1,C6orf48,C9orf135,C9orf16,C9orf173,C9orf3,CA5BP1,CABP4,CADM2,CAP2,CAPN6,CAPS2,CASC15,CBFB,CC2D2B,CCDC170,CCDC37-AS1,CCHCR1,CCL23,CCM2,CCNB3,CCR3,CCT7,CD163,CD2,CD37,CD3D,CD47,CD48,CD52,CD74,CD81,CDCA2,CDH13,CDK2AP1,CDK5,CDK9,CDKN1A,CDON,CEBPD,CELA2B,CEP97,CFD,CFLAR-AS1,CGN,CHCHD2,CHIC1,CHL1,CHMP2A,CHTOP,CHURC1,CIAPIN1,CLC,CLDN4,CLEC10A,CLIC1,CLIC5,CLPTM1L,CNGA1,CNIH1,COL1A2,COL4A6,COL6A6,COLGALT2,COMMD3,COMMD6,COMP,COPRS,COTL1,COX16,CPEB4,CPNE2,CPNE4,CPS1-IT1,CREM,CRIM1,CRIP1,CRLF1,CSF1R,CSF2RB,CSF3R,CSNK2A2,CSNK2B,CSRP1,CST3,CSTB,CTBP1,CTC-550B14.7,CTD-2528L19.6,CTD-3080P12.3,CTDSP1,CTSB,CTSZ,CUEDC2,CXCL17,CXCR2,CYB561,CYBA,CYBB,CYFIP1,CYGB,CYP1A2,DAPL1,DCTN1,DDIT4,DDX23,DDX26B,DENND1A,DHRS9,DLG5-AS1,DMC1,DMD,DNAH14,DNAJC15,DNAJC16,DNAJC7,DNAJC9-AS1,DNER,DNM1P46,DOCK4,DPP4,DPY19L1P1,DPYSL2,DPYSL3,DRAP1,DSG2,DUSP6,DYRK2,E2F8,ECHS1,ECI1,EDN1,EDN3,EDNRA,EDNRB,EEF1D,EFHD2,EGF,EGLN3,EIF2AK3,EIF3G,EIF4G3,EIF5,EMR2,EMX1,ENO1,ENO3,ENOSF1,ENTPD1,EPB41L2,EPC1,EPHA2,ERBB4,ERCC6L,ERP44,ESRP1,ESRRG,ETFB,ETNPPL,ETS2,EVI2B,EWSR1,EXOC6B,F11R,FAM101B,FAM127A,FAM168A,FAM169A,FAM71C,FAM83B,FAM96B,FARSA,FAT4,FAU,FBLN1,FBLN5,FBXO10,FBXO32,FBXW12,FCER1G,FCGR2A,FCGR2C,FCGR3B,FCN1,FGF12,FGF2,FIS1,FKBP11,FLJ36848,FLRT3,FLT1,FMO3,FOLR2,FOXA3,FOXC2,FTCDNL1,FTH1,FTO,FUNDC2,FXYD1,FZD1,FZD4,GABARAPL2,GAP43,GATA4,GDI2,GFM1,GGTA1P,GJA4,GJC1,GLG1,GLIPR2,GLIS2,GLRB,GMDS,GNAI2,GNAQ,GNB2,GP5,GPI,GPM6A,GPR1,GPR171,GPR39,GPR85,GPSM2,GPX4,GRIK3,GRIN2A,GSDMD,GUK1,GUSB,GYPC,GZMA,GZMB,GZMH,HBB,HCG18,HCG4,HCK,HCN4,HEG1,HELZ2,HFE2,HIBCH,HIGD2A,HIST1H2AK,HLA-DPB1,HMGB2,HMOX2,HN1,HNRNPF,HOOK1,HOXC10,HRAS,HSD17B11,HSD17B12,HSF2,HSPE1,IBA57-AS1,ID1,ID2,IDH2,IFI6,IGFBP2,IGFBP3,IGFBP6,IGFLR1,IGKC,IGSF5,IKBKAP,IL13RA1,IL1R1,IMP3,ING3,INTS2,INTS6,IP6K2,ISOC2,ITGA6,ITGAM,ITGB1BP1,ITGB2,ITK,KALRN,KANK1,KAT7,KCNA5,KCNJ3,KCNK1,KCTD1,KCTD21,KDM4B,KDM7A,KDR,KIF1B,KIF5B,KIRREL3,KLB,KLF2,KLHL10,KLHL41,KLK7,KPNB1,KRT40,KRTAP17-1,KRTCAP2,KYNU,LAMC1,LAMTOR4,LAPTM5,LARS,LGALS1,LILRA6,LILRB4,LIN7A,LIN9,LINC00261,LINC00326,LINC00354,LINC00355,LINC00493,LINC00593,LINC00622,LINC00673,LINC00839,LINC00880,LINC00905,LINC00938,LINC00963,LINC00997,LINC01018,LINC01300,LIPA,LITAF,LIX1L,LOC100128840,LOC100129034,LOC100129455,LOC100130964,LOC100131303,LOC100190986,LOC100287290,LOC100288675,LOC100505978,LOC100506498,LOC100507477,LOC100652824,LOC100996263,LOC101926918,LOC101926960,LOC101926975,LOC101927085,LOC101927722,LOC101928288,LOC101928446,LOC101928614,LOC101928687,LOC101928955,LOC101929622,LOC101929757,LOC101930415,LOC284788,LOC285422,LOC338620,LOC339975,LOC643711,LOC646762,LOC728690,LOC728805,LPP-AS2,LRIG1,LRIG2,LRP4,LRR1,LRRC32,LRRC49,LRRC7,LRRN1,LST1,LY96,LYN,LYVE1,MACF1,MAD2L2,MAGOHB,MAL2,MAST1,MATR3,MCC,MCOLN1,MED23,MED25,MEG9,MEIG1,METRNL,METTL21A,MFN1,MGAM,MGARP,MGAT4C,MGC12488,MGC70870,MGMT,MICAL2,MICALCL,MID1IP1,MIF,MIR302B,MIR3682,MKNK2,MLH3,MMP24,MNDA,MPC2,MPEG1,MPG,MPHOSPH8,MPP5,MRPL10,MRPL12,MRPL41,MRPL43,MRPL54,MRPL9,MRPS16,MRPS22,MRPS6,MS4A4A,MSL3,MSRB2,MSS51,MT2A,MTCH1,MTFMT,MTHFD2,MTPN,MUM1L1,MYC,MYD88,MYH7,MYL6,MYLK3,MYO5B,MZB1,NAIP,NAP1L2,NBEA,NCF2,NCKAP5,NCKIPSD,ND6,NDFIP2,NDRG1,NDUFA11,NDUFA3,NDUFAF3,NDUFB10,NDUFC1,NDUFS7,NDUFV1,NEB,NECAP1,NENF,NFATC2IP,NFIL3,NFKBIA,NHSL1,NKAIN1,NKAIN2,NKTR,NKX3-1,NLGN4Y,NLN,NMU,NNT-AS1,NOSIP,NOV,NPAS3,NPC2,NPHP1,NPNT,NPR3,NQO2,NR3C2,NRSN1,NSF,NSUN7,NT5C3B,NT5DC4,NUDCD2,NXPE4,OOSP2,OR2F2,ORC4,OST4,OTOGL,P2RY13,P2RY8,PADI4,PARD6B,PCDHB9,PCNXL4,PDAP1,PDCD6,PDE3A,PDE4D,PDE7A,PDIA3,PEBP1,PECAM1,PEX1,PFDN1,PGAM1,PGAM2,PGAM5,PGD,PGM5,PHB,PHF10,PHLDA3,PID1,PIFO,PIGT,PIK3R3,PILRA,PIM3,PIN1P1,PIP5K1B,PITPNA,PLA2G7,PLAC8,PLAC9,PLEK2,PLEKHH1,POLB,POU5F1P3,PPARGC1B,PPDPF,PPIB,PPIL1,PPP1CB,PPP1R14B,PPP1R9A,PPP3R2,PPT1,PQBP1,PQLC3,PRADC1,PRAME,PRCP,PRDM11,PRDM4,PRDX4,PRDX6,PREB,PRELP,PREX1,PRIMA1,PRKAA2,PRKG2,PRMT1,PRNP,PROK2,PROKR2,PRSS12,PRSS36,PSMA7,PSMB3,PSMB6,PSMC3,PSMD5,PSME1,PSMG3,PSPH,PTCD2,PTGFRN,PTPLAD2,PTPRZ1,PWAR6,PXDNL,PYCARD,QPCT,RAB13,RAB32,RAB8A,RABAC1,RAC1,RAC2,RAC3,RANBP6,RAP1B,RASGEF1C,RASGRP3,RASIP1,RASSF3,RBM11,RBX1,RCAN1,RCN3,REST,RGCC,RGS10,RGS18,RGS3,RILP,RILPL2,RINT1,RLN1,RMND5A,RNASE1,RNASE6,RNASET2,RNF128,RNF130,RNF183,RNF19A,RNF212B,ROMO1,ROPN1B,RORB,RP11-1069G10.1,RP11-119F7.5,RP11-141M1.1,RP11-157B13.7,RP11-250B2.6,RP11-379H18.1,RP11-382B18.4,RP11-399O19.9,RP1-142L7.8,RP11-524D16__A.3,RP11-672L10.6,RP11-736K20.5,RP11-73K9.2,RP1-8B22.1,RP4-714D9.5,RPA1,RPA2,RPA3,RPL18,RPL19,RPL36,RPL36AL,RPL7AL2,RPLP1,RPPH1,RPS11,RPS12,RPS14,RPS21,RPS27,RPS3,RPS6,RPS9,RRAD,RRAGD,RSF1,RYR2,S100A12,S100A4,S100A6,S100A8,S100A9,S100P,SACS,SAMD12,SAP18,SBSPON,SCN2B,SCN4B,SCUBE2,SELL,SELM,SEPSECS-AS1,SERP1,SERPINA1,SET,SF3A3,SFRP1,SFRP4,SHB,SHC1,SHISA3,SIRPB2,SIVA1,SLA,SLC1A5,SLC25A5,SLC25A6,SLC26A9,SLC27A6,SLC35G3,SLC39A8,SLC3A1,SLC6A8,SLC7A11,SLCO4C1,SLIRP,SLITRK6,SMAP1,SMAP2,SMARCE1,SMCR8,SMIM17,SNAPC4,SNORA72,SNX14,SNX20,SNX22,SNX3,SOD1,SOD2,SOSTDC1,SOX9,SPAG4,SPHKAP,SPI1,SPOCK2,SPOP,SPTSSB,SQRDL,SRP72,SRRT,SSR1,STEAP2,STK17B,STK38L,STMN2,STOX2,STRIP2,STT3A,STT3B,STX10,SUCO,SUSD4,SYNGR1,SYNGR2,SYT13,TAF1A,TAS2R13,TAT,TBXAS1,TCEAL2,TCEAL7,TCEB2,TCF21,TDRP,TFAP2C,TGFB1,TICAM1,TMEM159,TMEM171,TMEM179,TMEM182,TMEM204,TMEM207,TMEM256,TMEM258,TMEM33,TMEM63C,TNFSF8,TOB1,TOMM22,TOP3B,TOR1B,TPP1,TPSB2,TRADD,TRAF3IP2,TRAPPC5,TREH,TRIM10,TRIM55,TSC22D1,TSC22D3,TSPAN15,TSTD1,TTC30A,TTN,TUBB6,TUBGCP5,TUFM,TUFT1,TXNDC15,TXNL4A,TYROBP,UBA3,UBE2K,UBE2L3,UBE2L6,UBL7,UBR3,UFD1L,UNC45B,UQCRC1,USP24,USP32P2,VAMP3,VAMP8,VAX2,VIM,VNN2,VOPP1,VPS13A,VPS28,VSIG4,VWC2,VWF,WBSCR22,WIF1,WWC3,WWP1,XK,YIPF5,YWHAE,YWHAH,ZADH2,ZBED9,ZDBF2,ZDHHC17,ZDHHC20,ZIC4,ZMIZ2,ZNF124,ZNF17,ZNF205-AS1,ZNF667-AS1,ZNF682,ZNF684,ZNF697,ZNF837,ZNHIT1 | | | |
| **708 of arrhythmia associated targets dataset from database** | | | |
| SCN5A,KCNH2,KCNQ1,RYR2,KCNJ2,KCNE2,CACNA1C,KCNE1,LMNA,PKP2,MYH7,DSP,AKAP9,SNTA1,TTN,CAV3,CASQ2,SCN4B,DSG2,KCNJ5,JUP,TMEM43,HCN4,TANGO2,CACNB2,MYBPC3,SCN1B,TNNT2,MYH6,DSC2,GJA5,DMD,NKX2-5,TRDN,RANGRF,NPPA,TNNI3,CACNA2D1,GJA1,GNB5,EMD,PLN,NOS1AP,KCND3,KCNJ8,KCNE3,DES,TGFB3,GPD1L,LDB3,CALM2,PRKAG2,MYPN,RBM20,CALM1,ABCC9,RYR1,GATA4,CPT2,TECRL,CACNA1S,ACTN2,SYNE2,TRPM4,SLC25A20,NEXN,KCNE5,TPM1,CACNA1D,DTNA,ACTC1,SYNE1,TAZ,CTNNA3,LAMP2,TTR,LAMA4,LAMA2,GATA6,TCAP,ACADVL,CALM3,TNNC1,SDHA,GYG1,GATA5,PPARG,FLNC,ACE,CSRP3,HFE,GAA,HADHA,FHL1,NUP155,AKAP10,MYL3,CAVIN1,SLMAP,RAF1,IFNG,DPP6,PTPN11,TBX5,XK,VCL,PRDM16,TNNI3K,MYL4,NPPB,GATAD1,HLA-DRB1,BAG3,LOC110121269,KCNQ1-AS1,SLC25A4,MECP2,DMPK,FOS,ACADM,ALG10B,MT-TL1,EYA4,ADRB1,BMP2,CPT1A,TBX3,ANKRD1,KCNQ1OT1,CTLA4,DNAJC19,MYOT,MYLK2,NAA10,MT-ND4,MT-ND5,SGCD,ELN,CRYAB,CAV1,TP53,TBX20,PITX2,FKBP1B,POLG,TWNK,MT-CYB,KCNJ18,SOS1,MYL2,IL6,KCNJ11,MT-CO1,MT-TK,ERCC6,ACADL,POLG2,CACNA1C-AS1,APOE,HRAS,AGPAT2,BSCL2,TMEM70,HJV,BVES,MT-ND1,FKTN,SLC25A35,GLA,GSN,PEX1,NR2F2,NDUFS2,TNF,TMPO,AGTR1,BRAF,NDUFB11,SGO1,ALB,PIK3CA,HADHB,SLC19A2,RRM2B,SCNN1A,PEX6,TTPA,MT-ATP6,JPH2,ATFB2,SDHB,TOP3A,PHYH,PEX7,CNBP,KRAS,ATFB1,ATFB5,SLC40A1,PSEN1,REN,MT-CO3,FBLN5,PEX14,PEX10,PEX2,PEX3,PEX5,PEX26,PEX12,PEX13,PEX16,EDN1,MYOZ2,PDGFRA,DYSF,KCND2,HLA-B,ADRB2,COL4A1,CLCNKB,PSEN2,PPCS,CIZ1,PSMB8,NRAS,CASR,GATA1,HBB,PRTN3,ERCC8,INS,FBN1,MT-CO2,CDH23,SLC12A3,CD2AP,SLC26A4,MT-ND6,MT-ND4L,MEFV,COL1A1,HAND2,RNF207,CHKB,RET,SDHC,EHMT1,ETFDH,PTH,FBXL4,SLC8A1,KCNIP2,IL1B,ABCC8,GPX4,LARS2,RPL35,TNXB,MTO1,PEX11B,MGME1,MT-TE,ADRA2C,KCNK3,GABRA3,KIT,NR3C2,GNAS,HAMP,TSC2,F2,AGT,NOS3,AKT1,PLEKHM2,CMH21,CD40LG,CRELD1,ZIC3,CITED2,MT-ND2,MT-TS1,MT-TF,BANF1,MIR133A1,APOB,LGALS3,TAB2,SLN,VHL,KCNJ12,ZMPSTE24,HLA-DPB1,TFR2,COL5A1,CAT,CAMK2G,ICAM1,SUN2,MIR499A,MIR29A,PLAT,VWF,KCNE4,FGFR3,EP300,SCNN1B,TSHR,RPS19,RPL5,PTPN22,SCNN1G,SOS2,FOXC2,EFEMP2,GTPBP3,RASA2,WIPI2,TDP2,A2ML1,TRPM6,KNG1,SOD1,GPC3,CPOX,FLAD1,FAS,HRC,TSC1,MAP2K1,MAP2K2,SELP,IGF1,PF4,CDH2,CXCL8,TET2,FKRP,CBL,PLEC,SHOC2,MIR196A2,MT-TT,CALR,CYP2C9,PIK3C2A,MT-TS2,MT-TV,MT-TQ,MT-TW,MT-TH,PHOX2B,PPA2,ATP2A2,TPM3,ANK3,NAV1,HMBS,PGM1,TPM2,ACAD9,SDHD,SGCA,SUN1,IL17A,KCNH1,GPT,PPARA,MIR21,RPS24,TGFB1,NSUN6,KCNQ2,HSPG2,PCCA,SALL4,COX7B,HCCS,HLADPA1,TLR4,IGHMBP2,IL2,MMP3,HMGCR,GNAQ,WAS,RPL11,GNA11,PCCB,SOX10,MRAS,GPC4,CSF2RA,RIT1,RPL15,RPS10,RPL35A,WIPF1,RRAS,PEX19,RPL26,RPL18,GDAP1,KAT6B,RPS26,RPS27,RPS7,LZTR1,RPS15A,RPS17,RPL27,TSPYL1,RPS29,RPS28,SPECC1L,BTNL2,TSR2,ADA2,CYP2D6,XIRP1,LDLR,HDAC4,TUBB1,CHAT,ERCC1,UQCRFS1,MIPEP,MIR199A1,PSMC4,CYP11B2,THBD,MEN1,KCNJ3,CAPN3,CAMK2D,MPO,ADORA1,CD4,LMNB1,IL4,FBXW7,SERPINA3,TTNAS1,CACNA1CAS2,HMGCL,CTNNB1,HMOX1,ALOX5,TGFBR2,MYBPC1,SLC22A5,EPO,SGCB,CXADR,MB,HMOX2,LEMD3,LMO7,NEBL,DCAF8,TPTE,H2AC18,FXN,FHL2,CDKL5,SELE,B2M,FH,TMEM127,ADM,CACNG1,CEP85L,NOS1,APLN,KCNQ3,DNMT3A,LMNB2,TNNT1,DSC3,SERPINC1,F10,CYCS,IL1RN,GDF15,TGFBR1,HSPD1,MMP9,FGFR1,CCND1,SCN3A,PMS2,TLR5,ELANE,IFIH1,DDX58,ABCA3,ADAMTS13,PPBP,SEC23B,SBDS,KDM4C,INPP5E,FKBP14,PAEP,MT-ND3,MT-ATP8,MALAT1,MIR378A,MTTL2,DYT23,PTEN,GDNF,COQ9,LIPT1,RNASE3,CHRM2,MAPK14,TLR2,ATP2A1,KCNA4,GMPR,MMP1,MT-TC,NOS2,APOA1,FABP3,ADIPOQ,HLA-DQB1,MYBPC2,PDE3A,MIR130A,SOD2,SLC9A1,OBSCN,IL18,SMAD3,ATRX,SDHAF2,VCAM1,VKORC1,ANXA5,CD34,NOTCH1,ATP2B4,DCHS1,KCNQ4,ZFHX3,AVP,TIMP1,KCNN3,ALG10,ARVD3,FKBP1A,FN1,VEGFA,LBR,CD8A,CASP9,ACADS,DAG1,ACTA1,CCR6,TFAM,GBE1,CCL16,PDLIM3,CALR3,MYOZ1,SYNE3,SYNE4,LEXM,MIR126,MIR214,CSF2,CYP4F2,ATP1B1,CSF3,LIMK1,FGF12,TGFB2,JAG1,KCNJ4,AKAP6,TLL1,CLIC2,MFAP5,ECE1,G6PD,FGF2,GH1,XIRP2,GBA,IKZF1,PAX8,ENPP1,OAS1,SLC2A10,PPOX,GTF2I,GTF2IRD1,BAZ1B,COQ4,CLIP2,GJC3,MAPK1,CACNA1H,ITGB1,CREB1,GYS1,ACTG1,ACTN1,NF1,CTNNA1,ITGB6,ITGAV,IL1R1,CKM,IL5,KCNA2,AMPD1,ACOX1,PSMD4,TBX2,HP,CD86,CCL5,ITGA1,SLC27A1,RPS5,ICOSLG,MYOM1,NRAP,UNC80,SSUH2,SELENON,MIR29B1,CFTR,CYP3A4,ENPEP,NPPA-AS1,LOC114827827,CDK4,MYC,NKX2-6,MMP2,CASP3,KCNB1,PTGS1,CACNB1,PDE4D,KCNJ6,ITPR2,PDGFRB,SGK1,FBXO32,PRKAA2,IDH1,AKT3,IDH2,PTCH1,CASP1,PIK3R2,COL2A1,ITGA7,TNFRSF1B,GABBR2,MIB1,PRF1,RTN4,ERCC4,LOX,ETFA,ETFB,CS,PSMB4,FOXG1,CXCL10,SUFU,SPTB,GCM2,LTBP4,TINF2,CDAN1,MIB2,MYH7B,C15orf41,NAT9,DNAAF3,TERC,MIR320A,MT-TI,MHRT,MT-TA,HBB-LCR,ARVD4,LVNC2,ATFB8,PRL,DRD2,ILK,CACNA1F,SERPINE1,PRKAA1,KLF4,RYR3,DNAH8,KL | | | |
| **1597 of targets combined from GSE41177 and databases** | | | |
| ENO3,ENOSF1,ENTPD1,EPB41L2,EPC1,EPHA2,ERBB4,ERCC6L,ERP44,ESRP1,ESRRG,ETNPPL,ETS2,EVI2B,EWSR1,EXOC6B,F11R,FAM101B,FAM127A,FAM168A,FAM169A,FAM71C,FAM83B,FAM96B,FARSA,FAT4,FAU,FBLN1,FBXO10,FBXW12,FCER1G,FCGR2A,FCGR2C,FCGR3B,FCN1,FIS1,FKBP11,FLJ36848,FLRT3,FLT1,FMO3,FOLR2,FOXA3,FTCDNL1,FTH1,FTO,FUNDC2,FXYD1,FZD1,FZD4,GABARAPL2,GAP43,GDI2,GFM1,GGTA1P,GJA4,GJC1,GLG1,GLIPR2,GLIS2,GLRB,GMDS,GNAI2,GNB2,GP5,GPI,GPM6A,GPR1,GPR171,GPR39,GPR85,GPSM2,GRIK3,GRIN2A,GSDMD,GUK1,GUSB,GYPC,GZMA,GZMB,GZMH,HCG18,HCG4,HCK,HEG1,HELZ2,HFE2,HIBCH,HIGD2A,HIST1H2AK,HMGB2,HN1,HNRNPF,HOOK1,HOXC10,HSD17B11,HSD17B12,HSF2,HSPE1,IBA57-AS1,ID1,ID2,IFI6,IGFBP2,IGFBP3,IGFBP6,IGFLR1,IGKC,IGSF5,IKBKAP,IL13RA1,IMP3,ING3,INTS2,INTS6,IP6K2,ISOC2,ITGA6,ITGAM,ITGB1BP1,ITGB2,ITK,KALRN,KANK1,KAT7,KCNA5,KCNK1,KCTD1,KCTD21,KDM4B,KDM7A,KDR,KIF1B,KIF5B,KIRREL3,KLB,KLF2,KLHL10,KLHL41,KLK7,KPNB1,KRT40,KRTAP17-1,KRTCAP2,KYNU,LAMC1,LAMTOR4,LAPTM5,LARS,LGALS1,LILRA6,LILRB4,LIN7A,LIN9,LINC00261,LINC00326,LINC00354,LINC00355,LINC00493,LINC00593,LINC00622,LINC00673,LINC00839,LINC00880,LINC00905,LINC00938,LINC00963,LINC00997,LINC01018,LINC01300,LIPA,LITAF,LIX1L,LOC100128840,LOC100129034,LOC100129455,LOC100130964,LOC100131303,LOC100190986,LOC100287290,LOC100288675,LOC100505978,LOC100506498,LOC100507477,LOC100652824,LOC100996263,LOC101926918,LOC101926960,LOC101926975,LOC101927085,LOC101927722,LOC101928288,LOC101928446,LOC101928614,LOC101928687,LOC101928955,LOC101929622,LOC101929757,LOC101930415,LOC284788,LOC285422,LOC338620,LOC339975,LOC643711,LOC646762,LOC728690,LOC728805,LPP-AS2,LRIG1,LRIG2,LRP4,LRR1,LRRC32,LRRC49,LRRC7,LRRN1,LST1,LY96,LYN,LYVE1,MACF1,MAD2L2,MAGOHB,MAL2,MAST1,MATR3,MCC,MCOLN1,MED23,MED25,MEG9,MEIG1,METRNL,METTL21A,MFN1,MGAM,MGARP,MGAT4C,MGC12488,MGC70870,MGMT,MICAL2,MICALCL,MID1IP1,MIF,MIR302B,MIR3682,MKNK2,MLH3,MMP24,MNDA,MPC2,MPEG1,MPG,MPHOSPH8,MPP5,MRPL10,MRPL12,MRPL41,MRPL43,MRPL54,MRPL9,MRPS16,MRPS22,MRPS6,MS4A4A,MSL3,MSRB2,MSS51,MT2A,MTCH1,MTFMT,MTHFD2,MTPN,MUM1L1,MYD88,MYL6,MYLK3,MYO5B,MZB1,NAIP,NAP1L2,NBEA,NCF2,NCKAP5,NCKIPSD,ND6,NDFIP2,NDRG1,NDUFA11,NDUFA3,NDUFAF3,NDUFB10,NDUFC1,NDUFS7,NDUFV1,NEB,NECAP1,NENF,NFATC2IP,NFIL3,NFKBIA,NHSL1,NKAIN1,NKAIN2,NKTR,NKX3-1,NLGN4Y,NLN,NMU,NNT-AS1,NOSIP,NOV,NPAS3,NPC2,NPHP1,NPNT,NPR3,NQO2,NRSN1,NSF,NSUN7,NT5C3B,NT5DC4,NUDCD2,NXPE4,OOSP2,OR2F2,ORC4,OST4,OTOGL,P2RY13,P2RY8,PADI4,PARD6B,PCDHB9,PCNXL4,PDAP1,PDCD6,PDE7A,PDIA3,PEBP1,PECAM1,PFDN1,PGAM1,PGAM2,PGAM5,PGD,PGM5,PHB,PHF10,PHLDA3,PID1,PIFO,PIGT,PIK3R3,PILRA,PIM3,PIN1P1,PIP5K1B,PITPNA,PLA2G7,PLAC8,PLAC9,PLEK2,PLEKHH1,POLB,POU5F1P3,PPARGC1B,PPDPF,PPIB,PPIL1,PPP1CB,PPP1R14B,PPP1R9A,PPP3R2,PPT1,PQBP1,PQLC3,PRADC1,PRAME,PRCP,PRDM11,PRDM4,PRDX4,PRDX6,PREB,PRELP,PREX1,PRIMA1,PRKG2,PRMT1,PRNP,PROK2,PROKR2,PRSS12,PRSS36,PSMA7,PSMB3,PSMB6,PSMC3,PSMD5,PSME1,PSMG3,PSPH,PTCD2,PTGFRN,PTPLAD2,PTPRZ1,PWAR6,PXDNL,PYCARD,QPCT,RAB13,RAB32,RAB8A,RABAC1,RAC1,RAC2,RAC3,RANBP6,RAP1B,RASGEF1C,RASGRP3,RASIP1,RASSF3,RBM11,RBX1,RCAN1,RCN3,REST,RGCC,RGS10,RGS18,RGS3,RILP,RILPL2,RINT1,RLN1,RMND5A,RNASE1,RNASE6,RNASET2,RNF128,RNF130,RNF183,RNF19A,RNF212B,ROMO1,ROPN1B,RORB,RP11-1069G10.1,RP11-119F7.5,RP11-141M1.1,RP11-157B13.7,RP11-250B2.6,RP11-379H18.1,RP11-382B18.4,RP11-399O19.9,RP1-142L7.8,RP11-524D16__A.3,RP11-672L10.6,RP11-736K20.5,RP11-73K9.2,RP1-8B22.1,RP4-714D9.5,RPA1,RPA2,RPA3,RPL19,RPL36,RPL36AL,RPL7AL2,RPLP1,RPPH1,RPS11,RPS12,RPS14,RPS21,RPS3,RPS6,RPS9,RRAD,RRAGD,RSF1,S100A12,S100A4,S100A6,S100A8,S100A9,S100P,SACS,SAMD12,SAP18,SBSPON,SCN2B,SCUBE2,SELL,SELM,SEPSECS-AS1,SERP1,SERPINA1,SET,SF3A3,SFRP1,SFRP4,SHB,SHC1,SHISA3,SIRPB2,SIVA1,SLA,SLC1A5,SLC25A5,SLC25A6,SLC26A9,SLC27A6,SLC35G3,SLC39A8,SLC3A1,SLC6A8,SLC7A11,SLCO4C1,SLIRP,SLITRK6,SMAP1,SMAP2,SMARCE1,SMCR8,SMIM17,SNAPC4,SNORA72,SNX14,SNX20,SNX22,SNX3,SOSTDC1,SOX9,SPAG4,SPHKAP,SPI1,SPOCK2,SPOP,SPTSSB,SQRDL,SRP72,SRRT,SSR1,STEAP2,STK17B,STK38L,STMN2,STOX2,STRIP2,STT3A,STT3B,STX10,SUCO,SUSD4,SYNGR1,SYNGR2,SYT13,TAF1A,TAS2R13,TAT,TBXAS1,TCEAL2,TCEAL7,TCEB2,TCF21,TDRP,TFAP2C,TICAM1,TMEM159,TMEM171,TMEM179,TMEM182,TMEM204,TMEM207,TMEM256,TMEM258,TMEM33,TMEM63C,TNFSF8,TOB1,TOMM22,TOP3B,TOR1B,TPP1,TPSB2,TRADD,TRAF3IP2,TRAPPC5,TREH,TRIM10,TRIM55,TSC22D1,TSC22D3,TSPAN15,TSTD1,TTC30A,TUBB6,TUBGCP5,TUFM,TUFT1,TXNDC15,TXNL4A,TYROBP,UBA3,UBE2K,UBE2L3,UBE2L6,UBL7,UBR3,UFD1L,UNC45B,UQCRC1,USP24,USP32P2,VAMP3,VAMP8,VAX2,VIM,VNN2,VOPP1,VPS13A,VPS28,VSIG4,VWC2,WBSCR22,WIF1,WWC3,WWP1,YIPF5,YWHAE,YWHAH,ZADH2,ZBED9,ZDBF2,ZDHHC17,ZDHHC20,ZIC4,ZMIZ2,ZNF124,ZNF17,ZNF205-AS1,ZNF667-AS1,ZNF682,ZNF684,ZNF697,ZNF837,ZNHIT1,TUBB6,TUBGCP5,TUFM,TUFT1,TXNDC15,TXNL4A,TYROBP,UBA3,UBE2K,UBE2L3,UBE2L6,UBL7,UBR3,UFD1L,UNC45B,UQCRC1,USP16,USP24,USP32P2,VAMP3,VAMP8,VASH2,VAX2,VIM,VNN2,VOPP1,VPS13A,VPS28,VSIG4,VWC2,VWDE,WBSCR22,WIF1,WWC3,WWP1,YIPF5,YWHAE,YWHAH,ZADH2,ZBED9,ZDBF2,ZDHHC17,ZDHHC20,ZIC4,ZMIZ2,ZNF124,ZNF17,ZNF205-AS1,ZNF667-AS1,ZNF682,ZNF684,ZNF697,ZNF708,ZNF837,ZNHIT1 | | | |

**Table S4** GSE41177 GSEA C5 ontology gene analysis (*p*.adjust value < 0.05, arrhythmia related top terms)

| ID | Description | setSize | enrichmentScore | NES | pvalue | p.adjust | qvalues | rank | leading_edge | core_enrichment |
| --- | --- | --- | --- | --- | --- | --- | --- | --- | --- | --- |
| GO_CATION_CHANNEL_COMPLEX | CC | 11 | 0.573979 | 2.282604 | 0.002611 | 0.016383 | 0.01032 | 304 | tags=91%, list=36%, signal=59% | KCNJ3/GRIN2A/RYR2/SCN2B/VWC2/HCN4/GRIK3/KCNA5/PDE4D/KCNK1 |
| GO_TRANSPORTER_COMPLEX | CC | 18 | 0.400357 | 2.033663 | 0.008671 | 0.033351 | 0.021008 | 260 | tags=72%, list=30%, signal=51% | KCNJ3/LRRC7/CLDN4/GRIN2A/RYR2/SCN2B/VWC2/CLIC5/HCN4/GRIK3/GLRB/KCNA5/PDE4D |
| GO_CARDIAC_MUSCLE_CELL_CONTRACTION | BP | 11 | 0.577135 | 2.247059 | 0.002519 | 0.021411 | 0.014853 | 302 | tags=91%, list=35%, signal=60% | KCNJ3/FGF12/RYR2/SCN2B/HCN4/KCNA5/PDE4D/DSG2/GATA4/GJC1 |
| GO_CARDIAC_MUSCLE_CONTRACTION | BP | 14 | 0.546356 | 2.35682 | 0.005155 | 0.0338 | 0.023448 | 343 | tags=93%, list=40%, signal=57% | KCNJ3/FGF12/RYR2/SCN2B/DMD/TTN/HCN4/KCNA5/PDE4D/DSG2/GATA4/GJC1/MYH7 |
| GO_CARDIAC_MUSCLE_CELL_ACTION_POTENTIAL | BP | 12 | 0.563689 | 2.249594 | 0.005236 | 0.033847 | 0.023481 | 321 | tags=92%, list=38%, signal=58% | KCNJ3/FGF12/ANK3/RYR2/SCN2B/DMD/HCN4/KCNA5/DSG2/GJC1/YWHAE |
| GO_REGULATION_OF_HEART_RATE | BP | 14 | 0.462676 | 1.995849 | 0.007732 | 0.043658 | 0.030287 | 401 | tags=93%, list=47%, signal=50% | NMU/KCNJ3/RYR2/SCN2B/DMD/HCN4/KCNA5/PDE4D/DSG2/YWHAE/MYH7/EDN3/SCN4B |

**Table S5** GSE41177 GO analysis (*p*.adjust value <0.05, arrhythmia related top terms)

| ONTOLOGY | ID | Description | GeneRatio | BgRatio | pvalue | p.adjust | qvalue | geneID | Count | zscore |
| --- | --- | --- | --- | --- | --- | --- | --- | --- | --- | --- |
| BP | GO:0006936 | muscle contraction | 35/764 | 360/18670 | 2.085E-06 | 0.000950952 | 0.0008202 | ANXA6/ATP2B4/C12orf57/COMP/DMD/DOCK4/DSG2/EDN1/EDN3/EDNRA/EDNRB/ENO1/FGF12/FXYD1/GATA4/GJC1/HCN4/KCNA5/KCNJ3/KLHL41/MYH7/MYL6/NEB/NMU/NPNT/PDE4D/PGAM2/PROK2/RYR2/SCN2B/SCN4B/SLC6A8/SOD1/TTN/VIM | 35 | 0.5070926 |
| BP | GO:0070252 | actin-mediated cell contraction | 16/764 | 116/18670 | 2.011E-05 | 0.003363143 | 0.0029007 | DMD/DSG2/FGF12/GATA4/GJC1/KCNA5/KCNJ3/MYH7/MYL6/NEB/PDE4D/RYR2/SCN2B/SCN4B/TTN/VIM | 16 | 2.5 |
| BP | GO:0003012 | muscle system process | 38/764 | 465/18670 | 4.283E-05 | 0.005269261 | 0.0045448 | ANXA6/ATP2B4/C12orf57/CDK9/COMP/CYBA/DMD/DOCK4/DSG2/EDN1/EDN3/EDNRA/EDNRB/ENO1/FGF12/FXYD1/GATA4/GJC1/HCN4/KCNA5/KCNJ3/KLHL41/MTPN/MYH7/MYL6/NEB/NMU/NPNT/PDE4D/PGAM2/PROK2/RYR2/SCN2B/SCN4B/SLC6A8/SOD1/TTN/VIM | 38 | 0.3244428 |
| BP | GO:0086001 | cardiac muscle cell action potential | 12/764 | 74/18670 | 4.307E-05 | 0.005269261 | 0.0045448 | ANK3/DMD/DSG2/FGF12/GJC1/HCN4/KCNA5/KCNJ3/RYR2/SCN2B/SCN4B/YWHAE | 12 | 2.8867513 |
| BP | GO:0002027 | regulation of heart rate | 14/764 | 101/18670 | 6.193E-05 | 0.006555502 | 0.0056541 | DMD/DSG2/EDN1/EDN3/HCN4/KCNA5/KCNJ3/MYH7/NMU/PDE4D/RYR2/SCN2B/SCN4B/YWHAE | 14 | 2.1380899 |
| CC | GO:0005911 | cell-cell junction | 34/784 | 459/19717 | 0.0003974 | 0.012172461 | 0.0103033 | ABCB11/ANK3/CD2/CDH13/CGN/CLDN4/DPP4/DSG2/EPHA2/F11R/FLRT3/FXYD1/FZD4/GJA4/GJC1/HEG1/IGSF5/ITGA6/ITK/KCNA5/LIN7A/LRRC7/LYN/MPP5/NDRG1/NPHP1/PARD6B/PECAM1/PGM5/RAB13/RAP1B/RASIP1/SCN4B/YWHAH | 34 | 0.3429972 |
| CC | GO:0014704 | intercalated disc | 8/784 | 50/19717 | 0.0007362 | 0.021250024 | 0.017987 | ANK3/DSG2/FXYD1/GJC1/KCNA5/PGM5/SCN4B/YWHAH | 8 | 0 |
| CC | GO:0044291 | cell-cell contact zone | 9/784 | 71/19717 | 0.0019453 | 0.032347958 | 0.0273808 | ANK3/DSG2/FXYD1/GJC1/KCNA5/PECAM1/PGM5/SCN4B/YWHAH | 9 | -0.333333 |
| MF | GO:0050786 | RAGE receptor binding | 6/745 | 11/17697 | 2.102E-06 | 0.001772366 | 0.0016488 | HMGB2/S100A12/S100A4/S100A8/S100A9/S100P | 6 | -1.632993 |
| MF | GO:0003735 | structural constituent of ribosome | 22/745 | 202/17697 | 4.435E-05 | 0.018694772 | 0.017391 | MRPL10/MRPL12/MRPL41/MRPL43/MRPL54/MRPL9/MRPS16/MRPS22/MRPS6/RPL18/RPL19/RPL36/RPL36AL/RPLP1/RPS11/RPS12/RPS14/RPS21/RPS27/RPS3/RPS6/RPS9 | 22 | -3.837613 |
| MF | GO:0002020 | protease binding | 16/745 | 128/17697 | 9.502E-05 | 0.026700384 | 0.0248383 | ADAMTSL4/CHL1/COL1A2/COMP/CST3/CSTB/DPP4/IL1R1/MIF/NDUFS7/PRNP/PYCARD/SELL/SERPINA1/TTN/VWF | 16 | -1.5 |
| KEGG | hsa04015 | Rap1 signaling pathway | 23/394 | 210/8076 | 0.0002207 | 0.012133042 | 0.00948 | AKT3/ARAP3/CSF1R/DOCK4/EGF/EPHA2/FGF2/FLT1/GNAI2/GNAQ/GRIN2A/HRAS/ID1/ITGAM/ITGB2/KDR/PARD6B/PIK3R3/RAC1/RAC2/RAC3/RAP1B/RASGRP3 | 23 | -1.042572 |
| KEGG | hsa04024 | cAMP signaling pathway | 23/394 | 216/8076 | 0.0003336 | 0.012133042 | 0.00948 | AKT3/ARAP3/ATP1B3/ATP2B4/CNGA1/EDN1/EDN3/EDNRA/FXYD1/GNAI2/GRIN2A/HCN4/NFKBIA/PDE3A/PDE4D/PIK3R3/PPP1CB/RAC1/RAC2/RAC3/RAP1B/RYR2/SOX9 | 23 | -0.625543 |
| KEGG | hsa05022 | Pathways of neurodegeneration - multiple diseases | 40/394 | 475/8076 | 0.0004527 | 0.014638223 | 0.0114374 | BID/CDK5/CSNK2A2/CSNK2B/CYBB/DCTN1/DNAH14/EIF2AK3/FZD1/FZD4/GABARAPL2/GNAQ/GRIN2A/HRAS/KIF5B/MFN1/ND6/NDUFA11/NDUFA3/NDUFB10/NDUFC1/NDUFS7/NDUFV1/PPP3R2/PRNP/PSMA7/PSMB3/PSMB6/PSMC3/RAB8A/RAC1/RYR2/SLC25A5/SLC25A6/SMCR8/SOD1/TUBB6/UBE2L3/UBE2L6/UQCRC1 | 40 | -3.162278 |
