# Supplementary material for: Fushenmu treatment ameliorates RyR2 with related metabolites in a zebrafish model of barium chloride induced arrhythmia
Source: Chin Med. 2023 Aug 19;18:103. doi: 10.1186/s13020-023-00812-x (PMC10439546; doi:10.1186/s13020-023-00812-x)
Supplement: Supplementary file 3 — Additional file 3: Table S6. 161 common targets between ‘FSM—compounds—targets’ and ‘arrhythmia—targets’. Table S7. Node degree results from 161 targets. Table S8. Interaction combined score results of 161 targets. Table S9. ‘FSM—compounds – arrhythmia– targets’ network node. Table S10. GO analysis for 33 candidate targets (p.adjust value < 0.05, arrhythmia related top terms). Table S11. ‘FSM—compounds – arrhythmia– targets’ network node. Table S11. Most enrichment targets and compounds from ‘FSM—compounds – arrhythmia– targets’ network. [file 13020_2023_812_MOESM3_ESM.docx]

**Additional File 3 for ‘FSM - compounds – arrhythmia– targets’ network construction**

**Table S6** 161 common targets between ‘FSM - compounds - targets’ and ‘arrhythmia - targets’

| NO. | Symbol | Entrez ID | Name |
| --- | --- | --- | --- |
| 1 | ABCB11 | 8647 | ATP binding cassette subfamily B member 11 |
| 2 | ABCC8 | 6833 | ATP binding cassette subfamily C member 8 |
| 3 | ABCC9 | 10060 | ATP binding cassette subfamily C member 9 |
| 4 | ACAD8 | 27034 | acyl-CoA dehydrogenase family member 8 |
| 5 | ACADM | 34 | acyl-CoA dehydrogenase medium chain |
| 6 | ACADS | 35 | acyl-CoA dehydrogenase short chain |
| 7 | ACADSB | 36 | acyl-CoA dehydrogenase short/branched chain |
| 8 | ACOX1 | 51 | acyl-CoA oxidase 1 |
| 9 | ACSL4 | 2182 | acyl-CoA synthetase long chain family member 4 |
| 10 | ACSS1 | 84532 | acyl-CoA synthetase short chain family member 1 |
| 11 | ADIPOQ | 9370 | "adiponectin, C1Q and collagen domain containing" |
| 12 | ADORA1 | 134 | adenosine A1 receptor |
| 13 | ADRA2C | 152 | adrenoceptor alpha 2C |
| 14 | ADRB1 | 153 | adrenoceptor beta 1 |
| 15 | ADRB2 | 154 | adrenoceptor beta 2 |
| 16 | AGT | 183 | angiotensinogen |
| 17 | AKT3 | 10000 | AKT serine/threonine kinase 3 |
| 18 | ALOX5 | 240 | arachidonate 5-lipoxygenase |
| 19 | ALOX5AP | 241 | arachidonate 5-lipoxygenase activating protein |
| 20 | APLN | 8862 | apelin |
| 21 | ARRB2 | 409 | arrestin beta 2 |
| 22 | ATP2A1 | 487 | ATPase sarcoplasmic/endoplasmic reticulum Ca2+ transporting 1 |
| 23 | ATP2B4 | 493 | ATPase plasma membrane Ca2+ transporting 4 |
| 24 | AVP | 551 | arginine vasopressin |
| 25 | CACNA1D | 776 | calcium voltage-gated channel subunit alpha1 D |
| 26 | CACNA1F | 778 | calcium voltage-gated channel subunit alpha1 F |
| 27 | CACNA1S | 779 | calcium voltage-gated channel subunit alpha1 S |
| 28 | CACNA2D1 | 781 | calcium voltage-gated channel auxiliary subunit alpha2delta 1 |
| 29 | CACNB1 | 782 | calcium voltage-gated channel auxiliary subunit beta 1 |
| 30 | CACNB2 | 783 | calcium voltage-gated channel auxiliary subunit beta 2 |
| 31 | CACNG1 | 786 | calcium voltage-gated channel auxiliary subunit gamma 1 |
| 32 | CAMK2D | 817 | calcium/calmodulin dependent protein kinase II delta |
| 33 | CAPN3 | 825 | calpain 3 |
| 34 | CASP9 | 842 | caspase 9 |
| 35 | CASQ2 | 845 | calsequestrin 2 |
| 36 | CAV3 | 859 | caveolin 3 |
| 37 | CCL5 | 6352 | C-C motif chemokine ligand 5 |
| 38 | CD34 | 947 | CD34 molecule |
| 39 | CFTR | 1080 | CF transmembrane conductance regulator |
| 40 | CKM | 1158 | "creatine kinase, M-type" |
| 41 | COL1A1 | 1277 | collagen type I alpha 1 chain |
| 42 | COLGALT2 | 23127 | collagen beta(1-O)galactosyltransferase 2 |
| 43 | COX7B | 1349 | cytochrome c oxidase subunit 7B |
| 44 | CPT1A | 1374 | carnitine palmitoyltransferase 1A |
| 45 | CPT2 | 1376 | carnitine palmitoyltransferase 2 |
| 46 | CREB1 | 1385 | cAMP responsive element binding protein 1 |
| 47 | CYGB | 114757 | cytoglobin |
| 48 | CYP1A2 | 1544 | cytochrome P450 family 1 subfamily A member 2 |
| 49 | CYP3A4 | 1576 | cytochrome P450 family 3 subfamily A member 4 |
| 50 | CYP4F2 | 8529 | cytochrome P450 family 4 subfamily F member 2 |
| 51 | DHRS9 | 10170 | dehydrogenase/reductase 9 |
| 52 | DNMT3A | 1788 | DNA methyltransferase 3 alpha |
| 53 | DRD2 | 1813 | dopamine receptor D2 |
| 54 | EGLN3 | 112399 | egl-9 family hypoxia inducible factor 3 |
| 55 | ENPP1 | 5167 | ectonucleotide pyrophosphatase/phosphodiesterase 1 |
| 56 | EPO | 2056 | erythropoietin |
| 57 | ESRRG | 2104 | estrogen related receptor gamma |
| 58 | ETFDH | 2110 | electron transfer flavoprotein dehydrogenase |
| 59 | F2 | 2147 | "coagulation factor II, thrombin" |
| 60 | FABP3 | 2170 | fatty acid binding protein 3 |
| 61 | FAU | 2197 | FAU ubiquitin like and ribosomal protein S30 fusion |
| 62 | FGF2 | 2247 | fibroblast growth factor 2 |
| 63 | FGFR1 | 2260 | fibroblast growth factor receptor 1 |
| 64 | FKBP1A | 2280 | FKBP prolyl isomerase 1A |
| 65 | FKBP1B | 2281 | FKBP prolyl isomerase 1B |
| 66 | FN1 | 2335 | fibronectin 1 |
| 67 | GABRA3 | 2556 | gamma-aminobutyric acid type A receptor subunit alpha3 |
| 68 | GJA5 | 2702 | gap junction protein alpha 5 |
| 69 | GNAS | 2778 | GNAS complex locus |
| 70 | GNB5 | 10681 | G protein subunit beta 5 |
| 71 | GPD1L | 23171 | glycerol-3-phosphate dehydrogenase 1 like |
| 72 | GRIN2A | 2903 | glutamate ionotropic receptor NMDA type subunit 2A |
| 73 | GUK1 | 2987 | guanylate kinase 1 |
| 74 | HDAC4 | 9759 | histone deacetylase 4 |
| 75 | HEG1 | 57493 | heart development protein with EGF like domains 1 |
| 76 | HMBS | 3145 | hydroxymethylbilane synthase |
| 77 | HMGCR | 3156 | 3-hydroxy-3-methylglutaryl-CoA reductase |
| 78 | HMOX1 | 3162 | heme oxygenase 1 |
| 79 | HSD17B11 | 51170 | hydroxysteroid 17-beta dehydrogenase 11 |
| 80 | IGF1 | 3479 | insulin like growth factor 1 |
| 81 | ITGAV | 3685 | integrin subunit alpha V |
| 82 | ITGB2 | 3689 | integrin subunit beta 2 |
| 83 | ITPR2 | 3709 | "inositol 1,4,5-trisphosphate receptor type 2" |
| 84 | JAG1 | 182 | jagged canonical Notch ligand 1 |
| 85 | KCNA5 | 3741 | potassium voltage-gated channel subfamily A member 5 |
| 86 | KCNB1 | 3745 | potassium voltage-gated channel subfamily B member 1 |
| 87 | KCNE1L | 23630 | Potassium Voltage-Gated Channel Subfamily E Regulatory Subunit 5 |
| 88 | KCNH2 | 3757 | potassium voltage-gated channel subfamily H member 2 |
| 89 | KCNJ11 | 3767 | potassium inwardly rectifying channel subfamily J member 11 |
| 90 | KCNQ1 | 3784 | potassium voltage-gated channel subfamily Q member 1 |
| 91 | KCNQ2 | 3785 | potassium voltage-gated channel subfamily Q member 2 |
| 92 | KCNQ3 | 3786 | potassium voltage-gated channel subfamily Q member 3 |
| 93 | KL | 9365 | klotho |
| 94 | MAPK1 | 5594 | mitogen-activated protein kinase 1 |
| 95 | MED25 | 81857 | mediator complex subunit 25 |
| 96 | METRNL | 284207 | "meteorin like, glial cell differentiation regulator" |
| 97 | MPO | 4353 | myeloperoxidase |
| 98 | MRPL12 | 6182 | mitochondrial ribosomal protein L12 |
| 99 | MYL2 | 4633 | myosin light chain 2 |
| 100 | NOS1 | 4842 | nitric oxide synthase 1 |
| 101 | NOS2 | 4843 | nitric oxide synthase 2 |
| 102 | NOTCH1 | 4851 | notch receptor 1 |
| 103 | NPPA | 4878 | natriuretic peptide A |
| 104 | NQO2 | 4835 | N-ribosyldihydronicotinamide:quinone reductase 2 |
| 105 | NR2F2 | 7026 | nuclear receptor subfamily 2 group F member 2 |
| 106 | NR3C2 | 4306 | nuclear receptor subfamily 3 group C member 2 |
| 107 | PAEP | 5047 | progestagen associated endometrial protein |
| 108 | PDE3A | 5139 | phosphodiesterase 3A |
| 109 | PDE4D | 5144 | phosphodiesterase 4D |
| 110 | PDE7A | 5150 | phosphodiesterase 7A |
| 111 | PF4 | 5196 | platelet factor 4 |
| 112 | PIK3CA | 5290 | "phosphatidylinositol-4,5-bisphosphate 3-kinase catalytic subunit alpha" |
| 113 | PIK3R2 | 5296 | phosphoinositide-3-kinase regulatory subunit 2 |
| 114 | PIK3R3 | 8503 | phosphoinositide-3-kinase regulatory subunit 3 |
| 115 | PLAT | 5327 | "plasminogen activator, tissue type" |
| 116 | POLB | 5423 | DNA polymerase beta |
| 117 | PPARA | 5465 | peroxisome proliferator activated receptor alpha |
| 118 | PPT1 | 5538 | palmitoyl-protein thioesterase 1 |
| 119 | PRDM16 | 63976 | PR/SET domain 16 |
| 120 | PRDX6 | 9588 | peroxiredoxin 6 |
| 121 | PRKAA1 | 5562 | protein kinase AMP-activated catalytic subunit alpha 1 |
| 122 | PRKAA2 | 5563 | protein kinase AMP-activated catalytic subunit alpha 2 |
| 123 | PRSS12 | 8492 | serine protease 12 |
| 124 | PTGS1 | 5742 | prostaglandin-endoperoxide synthase 1 |
| 125 | RET | 5979 | ret proto-oncogene |
| 126 | RGCC | 28984 | regulator of cell cycle |
| 127 | RRM2B | 50484 | ribonucleotide reductase regulatory TP53 inducible subunit M2B |
| 128 | RYR1 | 6261 | ryanodine receptor 1 |
| 129 | RYR2 | 6262 | ryanodine receptor 2 |
| 130 | S100A8 | 6279 | S100 calcium binding protein A8 |
| 131 | S100A9 | 6280 | S100 calcium binding protein A9 |
| 132 | S100P | 6286 | S100 calcium binding protein P |
| 133 | SCN1B | 6324 | sodium voltage-gated channel beta subunit 1 |
| 134 | SCN2B | 6327 | sodium voltage-gated channel beta subunit 2 |
| 135 | SCN3A | 6328 | sodium voltage-gated channel alpha subunit 3 |
| 136 | SCN4B | 6330 | sodium voltage-gated channel beta subunit 4 |
| 137 | SDHA | 6389 | succinate dehydrogenase complex flavoprotein subunit A |
| 138 | SDHAF2 | 54949 | succinate dehydrogenase complex assembly factor 2 |
| 139 | SDHB | 6390 | succinate dehydrogenase complex iron sulfur subunit B |
| 140 | SDHC | 6391 | succinate dehydrogenase complex subunit C |
| 141 | SDHD | 6392 | succinate dehydrogenase complex subunit D |
| 142 | SLC19A2 | 10560 | solute carrier family 19 member 2 |
| 143 | SLC22A5 | 6584 | solute carrier family 22 member 5 |
| 144 | SLC25A20 | 788 | solute carrier family 25 member 20 |
| 145 | SLC25A4 | 291 | solute carrier family 25 member 4 |
| 146 | SLC25A5 | 292 | solute carrier family 25 member 5 |
| 147 | SLC25A6 | 293 | solute carrier family 25 member 6 |
| 148 | SLC6A8 | 6535 | solute carrier family 6 member 8 |
| 149 | SLC8A1 | 6546 | solute carrier family 8 member A1 |
| 150 | SLC9A1 | 6548 | solute carrier family 9 member A1 |
| 151 | SMAD3 | 4088 | SMAD family member 3 |
| 152 | SOS2 | 6655 | SOS Ras/Rho guanine nucleotide exchange factor 2 |
| 153 | SOX9 | 6662 | SRY-box transcription factor 9 |
| 154 | SPI1 | 6688 | Spi-1 proto-oncogene |
| 155 | SPOP | 8405 | speckle type BTB/POZ protein |
| 156 | SQRDL | 58472 | Sulfide Quinone Oxidoreductase |
| 157 | TGFBR1 | 7046 | transforming growth factor beta receptor 1 |
| 158 | TGFBR2 | 7048 | transforming growth factor beta receptor 2 |
| 159 | THBD | 7056 | thrombomodulin |
| 160 | UBA3 | 9039 | ubiquitin like modifier activating enzyme 3 |
| 161 | VKORC1 | 79001 | vitamin K epoxide reductase complex subunit 1 |

**Table S7** Node degree results from 161 targets

| #node | node_degree | #node | node_degree | #node | node_degree | #node | node_  degree | #node | node_  degree |
| --- | --- | --- | --- | --- | --- | --- | --- | --- | --- |
| RYR2 | 33 | ABCC9 | 16 | TGFBR1 | 12 | GRIN2A | 7 | PDE7A | 3 |
| FN1 | 32 | ADRB2 | 16 | ABCC8 | 11 | HDAC4 | 7 | RRM2B | 3 |
| KCNH2 | 29 | CD34 | 16 | ARRB2 | 11 | ITPR2 | 7 | SLC19A2 | 3 |
| CREB1 | 28 | CPT2 | 16 | ATP2A1 | 11 | PRKAA1 | 7 | APLN | 2 |
| NOTCH1 | 28 | HMOX1 | 16 | CACNG1 | 11 | S100A8 | 7 | COX7B | 2 |
| CAV3 | 27 | SCN2B | 16 | FABP3 | 11 | SLC22A5 | 7 | CYGB | 2 |
| PPARA | 27 | TGFBR2 | 16 | GJA5 | 11 | SLC25A4 | 7 | GABRA3 | 2 |
| CACNB2 | 26 | ACOX1 | 15 | KCNQ3 | 11 | ACSS1 | 6 | HMBS | 2 |
| FGF2 | 26 | CACNB1 | 15 | SLC8A1 | 11 | CYP1A2 | 6 | METRNL | 2 |
| KCNQ1 | 26 | CFTR | 15 | ACSL4 | 10 | ENPP1 | 6 | PPT1 | 2 |
| MAPK1 | 26 | FGFR1 | 15 | CCL5 | 10 | FKBP1B | 6 | PRDX6 | 2 |
| CACNA1D | 25 | NOS1 | 15 | HMGCR | 10 | PRKAA2 | 6 | SLC25A6 | 2 |
| IGF1 | 25 | NPPA | 15 | ITGAV | 10 | SLC9A1 | 6 | ADRA2C | 1 |
| CACNA2D1 | 24 | RET | 15 | KL | 10 | SQRDL | 6 | COLGALT2 | 1 |
| PDE4D | 22 | SDHC | 15 | MYL2 | 10 | ABCB11 | 5 | ESRRG | 1 |
| PIK3CA | 22 | ACADS | 14 | PIK3R2 | 10 | ACAD8 | 5 | MED25 | 1 |
| RYR1 | 22 | CPT1A | 14 | SCN3A | 10 | CYP4F2 | 5 | NQO2 | 1 |
| SMAD3 | 22 | GPD1L | 14 | SDHD | 10 | DNMT3A | 5 | PAEP | 1 |
| CASQ2 | 20 | ITGB2 | 14 | ACADSB | 9 | PLAT | 5 | PRSS12 | 1 |
| GNAS | 20 | KCNE1L | 14 | CASP9 | 9 | PRDM16 | 5 | RGCC | 1 |
| KCNA5 | 20 | KCNQ2 | 14 | NOS2 | 9 | PTGS1 | 5 | SPOP | 1 |
| SCN1B | 20 | MPO | 14 | PIK3R3 | 9 | S100A9 | 5 | UBA3 | 1 |
| ADIPOQ | 18 | SOX9 | 14 | SLC25A20 | 9 | SDHAF2 | 5 | DHRS9 | 0 |
| CACNA1S | 18 | CACNA1F | 13 | ALOX5 | 8 | SLC25A5 | 5 | HEG1 | 0 |
| CAMK2D | 18 | COL1A1 | 13 | ATP2B4 | 8 | VKORC1 | 5 | HSD17B11 | 0 |
| F2 | 18 | EPO | 13 | AVP | 8 | ADORA1 | 4 | MRPL12 | 0 |
| GNB5 | 18 | ADRB1 | 12 | CYP3A4 | 8 | NR2F2 | 4 | POLB | 0 |
| KCNJ11 | 18 | AKT3 | 12 | PDE3A | 8 | SOS2 | 4 | S100P | 0 |
| ACADM | 17 | ETFDH | 12 | PF4 | 8 | CAPN3 | 3 | SLC6A8 | 0 |
| AGT | 17 | JAG1 | 12 | THBD | 8 | CKM | 3 |  |  |
| FKBP1A | 17 | KCNB1 | 12 | ALOX5AP | 7 | EGLN3 | 3 |  |  |
| SCN4B | 17 | SDHB | 12 | DRD2 | 7 | GUK1 | 3 |  |  |
| SDHA | 17 | SPI1 | 12 | FAU | 7 | NR3C2 | 3 |  |  |

*33 candidate targets are highlighted in yellow.

**Table S8** Interaction combined score results of 161 targets

| #node1 | node2 | combined_score | #node1 | node2 | combined_score | #node1 | node2 | combined_score |
| --- | --- | --- | --- | --- | --- | --- | --- | --- |
| ACADM | CASQ2 | 0.577 | CREB1 | NOTCH1 | 0.497 | KCNQ1 | KCNJ11 | 0.725 |
| ACADM | SDHA | 0.607 | CREB1 | IGF1 | 0.621 | KCNQ1 | KCNH2 | 0.987 |
| ACADM | PPARA | 0.973 | CREB1 | SMAD3 | 0.519 | MAPK1 | PDE4D | 0.403 |
| ADIPOQ | FGF2 | 0.785 | CREB1 | CAMK2D | 0.918 | MAPK1 | GNAS | 0.442 |
| ADIPOQ | IGF1 | 0.756 | CREB1 | PDE4D | 0.515 | MAPK1 | NOTCH1 | 0.486 |
| ADIPOQ | FN1 | 0.414 | CREB1 | FN1 | 0.457 | MAPK1 | PIK3CA | 0.603 |
| ADIPOQ | AGT | 0.652 | CREB1 | GNAS | 0.523 | MAPK1 | FN1 | 0.773 |
| ADIPOQ | PPARA | 0.824 | CREB1 | PPARA | 0.672 | MAPK1 | F2 | 0.918 |
| ADIPOQ | CREB1 | 0.429 | CREB1 | ADIPOQ | 0.429 | MAPK1 | GNB5 | 0.923 |
| AGT | MAPK1 | 0.926 | F2 | MAPK1 | 0.918 | MAPK1 | AGT | 0.926 |
| AGT | PIK3CA | 0.906 | F2 | FGF2 | 0.498 | MAPK1 | PPARA | 0.941 |
| AGT | IGF1 | 0.519 | F2 | IGF1 | 0.939 | MAPK1 | FGF2 | 0.942 |
| AGT | F2 | 0.962 | F2 | FN1 | 0.811 | MAPK1 | IGF1 | 0.945 |
| AGT | SMAD3 | 0.442 | F2 | GNAS | 0.912 | MAPK1 | CREB1 | 0.972 |
| AGT | FN1 | 0.582 | F2 | PPARA | 0.918 | MAPK1 | SMAD3 | 0.981 |
| AGT | ADIPOQ | 0.652 | F2 | AGT | 0.962 | NOTCH1 | MAPK1 | 0.486 |
| AGT | GNAS | 0.916 | FGF2 | MAPK1 | 0.942 | NOTCH1 | PIK3CA | 0.764 |
| AGT | PPARA | 0.939 | FGF2 | PIK3CA | 0.651 | NOTCH1 | FGF2 | 0.753 |
| CACNA1D | KCNQ1 | 0.483 | FGF2 | SMAD3 | 0.497 | NOTCH1 | SDHA | 0.462 |
| CACNA1D | KCNA5 | 0.569 | FGF2 | F2 | 0.498 | NOTCH1 | GNAS | 0.464 |
| CACNA1D | GNB5 | 0.903 | FGF2 | CREB1 | 0.543 | NOTCH1 | CREB1 | 0.497 |
| CACNA1D | KCNH2 | 0.559 | FGF2 | NOTCH1 | 0.753 | NOTCH1 | IGF1 | 0.708 |
| CACNA1D | KCNJ11 | 0.545 | FGF2 | ADIPOQ | 0.785 | NOTCH1 | FN1 | 0.72 |
| CACNA1D | PDE4D | 0.63 | FGF2 | FN1 | 0.93 | NOTCH1 | SMAD3 | 0.991 |
| CACNA1D | CAMK2D | 0.702 | FGF2 | IGF1 | 0.976 | PDE4D | MAPK1 | 0.403 |
| CACNA1D | CAV3 | 0.703 | FKBP1A | PIK3CA | 0.496 | PDE4D | CACNA1D | 0.63 |
| CACNA1D | FKBP1A | 0.729 | FKBP1A | CACNA1D | 0.729 | PDE4D | CACNB2 | 0.563 |
| CACNA1D | RYR2 | 0.789 | FKBP1A | CACNB2 | 0.72 | PDE4D | CAMK2D | 0.684 |
| CACNA1D | RYR1 | 0.884 | FKBP1A | SMAD3 | 0.926 | PDE4D | CREB1 | 0.515 |
| CACNA1D | CACNA1S | 0.952 | FKBP1A | PDE4D | 0.6 | PDE4D | FKBP1A | 0.6 |
| CACNA1D | CACNB2 | 0.982 | FKBP1A | CACNA2D1 | 0.724 | PDE4D | CACNA2D1 | 0.61 |
| CACNA1D | CACNA2D1 | 0.989 | FKBP1A | RYR1 | 0.999 | PDE4D | CACNA1S | 0.621 |
| CACNA1S | GNB5 | 0.611 | FKBP1A | CACNA1S | 0.87 | PDE4D | GNAS | 0.631 |
| CACNA1S | KCNH2 | 0.464 | FKBP1A | RYR2 | 0.995 | PDE4D | RYR2 | 0.656 |
| CACNA1S | CACNA1D | 0.952 | FN1 | MAPK1 | 0.773 | PDE4D | RYR1 | 0.742 |
| CACNA1S | CACNB2 | 0.991 | FN1 | FGF2 | 0.93 | PIK3CA | MAPK1 | 0.603 |
| CACNA1S | CAMK2D | 0.692 | FN1 | NOTCH1 | 0.72 | PIK3CA | GNB5 | 0.608 |
| CACNA1S | CAV3 | 0.791 | FN1 | IGF1 | 0.978 | PIK3CA | SMAD3 | 0.489 |
| CACNA1S | PDE4D | 0.621 | FN1 | F2 | 0.811 | PIK3CA | FKBP1A | 0.496 |
| CACNA1S | CACNA2D1 | 0.989 | FN1 | SMAD3 | 0.766 | PIK3CA | FGF2 | 0.651 |
| CACNA1S | RYR1 | 0.998 | FN1 | ADIPOQ | 0.414 | PIK3CA | GNAS | 0.694 |
| CACNA1S | RYR2 | 0.796 | FN1 | CREB1 | 0.457 | PIK3CA | NOTCH1 | 0.764 |
| CACNA1S | FKBP1A | 0.87 | FN1 | AGT | 0.582 | PIK3CA | AGT | 0.906 |
| CACNA2D1 | KCNQ1 | 0.59 | GNAS | KCNQ1 | 0.503 | PIK3CA | CREB1 | 0.926 |
| CACNA2D1 | CASQ2 | 0.407 | GNAS | MAPK1 | 0.442 | PIK3CA | IGF1 | 0.95 |
| CACNA2D1 | KCNH2 | 0.66 | GNAS | GNB5 | 0.839 | PPARA | MAPK1 | 0.941 |
| CACNA2D1 | CACNA1D | 0.989 | GNAS | PIK3CA | 0.694 | PPARA | IGF1 | 0.58 |
| CACNA2D1 | CACNB2 | 0.997 | GNAS | NOTCH1 | 0.464 | PPARA | F2 | 0.918 |
| CACNA2D1 | SCN4B | 0.515 | GNAS | F2 | 0.912 | PPARA | SMAD3 | 0.52 |
| CACNA2D1 | CAMK2D | 0.7 | GNAS | CAV3 | 0.624 | PPARA | AGT | 0.939 |
| CACNA2D1 | CAV3 | 0.403 | GNAS | PDE4D | 0.631 | PPARA | ACADM | 0.973 |
| CACNA2D1 | PDE4D | 0.61 | GNAS | AGT | 0.916 | PPARA | CREB1 | 0.672 |
| CACNA2D1 | KCNJ11 | 0.493 | GNAS | CREB1 | 0.523 | PPARA | ADIPOQ | 0.824 |
| CACNA2D1 | RYR2 | 0.563 | GNB5 | MAPK1 | 0.923 | RYR1 | CASQ2 | 0.832 |
| CACNA2D1 | SCN1B | 0.683 | GNB5 | PIK3CA | 0.608 | RYR1 | KCNH2 | 0.444 |
| CACNA2D1 | FKBP1A | 0.724 | GNB5 | CACNA1S | 0.611 | RYR1 | CACNA1D | 0.884 |
| CACNA2D1 | RYR1 | 0.905 | GNB5 | GNAS | 0.839 | RYR1 | CACNB2 | 0.75 |
| CACNA2D1 | CACNA1S | 0.989 | GNB5 | CACNB2 | 0.9 | RYR1 | CAV3 | 0.771 |
| CACNB2 | KCNQ1 | 0.632 | GNB5 | CACNA1D | 0.903 | RYR1 | PDE4D | 0.742 |
| CACNB2 | KCNA5 | 0.417 | IGF1 | MAPK1 | 0.945 | RYR1 | CACNA2D1 | 0.905 |
| CACNB2 | CASQ2 | 0.568 | IGF1 | PIK3CA | 0.95 | RYR1 | RYR2 | 0.955 |
| CACNB2 | GNB5 | 0.9 | IGF1 | FGF2 | 0.976 | RYR1 | CACNA1S | 0.998 |
| CACNB2 | KCNH2 | 0.655 | IGF1 | NOTCH1 | 0.708 | RYR1 | FKBP1A | 0.999 |
| CACNB2 | CACNA1D | 0.982 | IGF1 | SMAD3 | 0.479 | RYR2 | KCNQ1 | 0.69 |
| CACNB2 | KCNJ11 | 0.434 | IGF1 | AGT | 0.519 | RYR2 | KCNA5 | 0.521 |
| CACNB2 | CAV3 | 0.452 | IGF1 | PPARA | 0.58 | RYR2 | CASQ2 | 0.995 |
| CACNB2 | SCN4B | 0.529 | IGF1 | CREB1 | 0.621 | RYR2 | KCNH2 | 0.788 |
| CACNB2 | PDE4D | 0.563 | IGF1 | ADIPOQ | 0.756 | RYR2 | CACNA1D | 0.789 |
| CACNB2 | RYR2 | 0.588 | IGF1 | F2 | 0.939 | RYR2 | CACNB2 | 0.588 |
| CACNB2 | SCN1B | 0.671 | IGF1 | FN1 | 0.978 | RYR2 | SCN4B | 0.577 |
| CACNB2 | FKBP1A | 0.72 | KCNA5 | KCNQ1 | 0.71 | RYR2 | CAMK2D | 0.941 |
| CACNB2 | CAMK2D | 0.732 | KCNA5 | CACNB2 | 0.417 | RYR2 | CAV3 | 0.89 |
| CACNB2 | RYR1 | 0.75 | KCNA5 | SCN4B | 0.446 | RYR2 | PDE4D | 0.656 |
| CACNB2 | CACNA1S | 0.991 | KCNA5 | CASQ2 | 0.472 | RYR2 | KCNJ11 | 0.406 |
| CACNB2 | CACNA2D1 | 0.997 | KCNA5 | SCN1B | 0.517 | RYR2 | CACNA2D1 | 0.563 |
| CAMK2D | CACNA1D | 0.702 | KCNA5 | RYR2 | 0.521 | RYR2 | RYR1 | 0.955 |
| CAMK2D | CACNB2 | 0.732 | KCNA5 | KCNJ11 | 0.535 | RYR2 | CACNA1S | 0.796 |
| CAMK2D | SCN4B | 0.669 | KCNA5 | CACNA1D | 0.569 | RYR2 | SCN1B | 0.528 |
| CAMK2D | SCN1B | 0.603 | KCNA5 | CAV3 | 0.591 | RYR2 | FKBP1A | 0.995 |
| CAMK2D | PDE4D | 0.684 | KCNA5 | KCNH2 | 0.903 | SCN1B | KCNQ1 | 0.603 |
| CAMK2D | CACNA1S | 0.692 | KCNH2 | KCNQ1 | 0.987 | SCN1B | KCNA5 | 0.517 |
| CAMK2D | CACNA2D1 | 0.7 | KCNH2 | KCNA5 | 0.903 | SCN1B | CASQ2 | 0.429 |
| CAMK2D | CREB1 | 0.918 | KCNH2 | CASQ2 | 0.66 | SCN1B | KCNH2 | 0.661 |
| CAMK2D | RYR2 | 0.941 | KCNH2 | RYR1 | 0.444 | SCN1B | CACNB2 | 0.671 |
| CASQ2 | KCNQ1 | 0.614 | KCNH2 | CACNA1S | 0.464 | SCN1B | SCN4B | 0.991 |
| CASQ2 | KCNA5 | 0.472 | KCNH2 | CACNA1D | 0.559 | SCN1B | CAMK2D | 0.603 |
| CASQ2 | CACNA2D1 | 0.407 | KCNH2 | KCNJ11 | 0.578 | SCN1B | CAV3 | 0.476 |
| CASQ2 | SCN1B | 0.429 | KCNH2 | CACNB2 | 0.655 | SCN1B | KCNJ11 | 0.476 |
| CASQ2 | KCNJ11 | 0.449 | KCNH2 | CACNA2D1 | 0.66 | SCN1B | CACNA2D1 | 0.683 |
| CASQ2 | SCN4B | 0.499 | KCNH2 | SCN1B | 0.661 | SCN1B | RYR2 | 0.528 |
| CASQ2 | CACNB2 | 0.568 | KCNH2 | SCN4B | 0.725 | SCN4B | KCNQ1 | 0.683 |
| CASQ2 | ACADM | 0.577 | KCNH2 | CAV3 | 0.774 | SCN4B | KCNA5 | 0.446 |
| CASQ2 | KCNH2 | 0.66 | KCNH2 | RYR2 | 0.788 | SCN4B | CASQ2 | 0.499 |
| CASQ2 | CAV3 | 0.767 | KCNJ11 | KCNQ1 | 0.725 | SCN4B | KCNH2 | 0.725 |
| CASQ2 | RYR1 | 0.832 | KCNJ11 | KCNA5 | 0.535 | SCN4B | CACNB2 | 0.529 |
| CASQ2 | RYR2 | 0.995 | KCNJ11 | CASQ2 | 0.449 | SCN4B | CACNA2D1 | 0.515 |
| CAV3 | KCNQ1 | 0.665 | KCNJ11 | KCNH2 | 0.578 | SCN4B | RYR2 | 0.577 |
| CAV3 | KCNA5 | 0.591 | KCNJ11 | CACNA1D | 0.545 | SCN4B | CAMK2D | 0.669 |
| CAV3 | CASQ2 | 0.767 | KCNJ11 | CACNB2 | 0.434 | SCN4B | CAV3 | 0.716 |
| CAV3 | KCNH2 | 0.774 | KCNJ11 | CAV3 | 0.512 | SCN4B | SCN1B | 0.991 |
| CAV3 | CACNA1D | 0.703 | KCNJ11 | RYR2 | 0.406 | SDHA | NOTCH1 | 0.462 |
| CAV3 | CACNB2 | 0.452 | KCNJ11 | SCN1B | 0.476 | SDHA | ACADM | 0.607 |
| CAV3 | SCN4B | 0.716 | KCNJ11 | CACNA2D1 | 0.493 | SMAD3 | MAPK1 | 0.981 |
| CAV3 | CACNA2D1 | 0.403 | KCNQ1 | CACNA1D | 0.483 | SMAD3 | PIK3CA | 0.489 |
| CAV3 | SCN1B | 0.476 | KCNQ1 | GNAS | 0.503 | SMAD3 | FGF2 | 0.497 |
| CAV3 | KCNJ11 | 0.512 | KCNQ1 | CACNA2D1 | 0.59 | SMAD3 | NOTCH1 | 0.991 |
| CAV3 | GNAS | 0.624 | KCNQ1 | SCN1B | 0.603 | SMAD3 | IGF1 | 0.479 |
| CAV3 | RYR1 | 0.771 | KCNQ1 | CASQ2 | 0.614 | SMAD3 | AGT | 0.442 |
| CAV3 | CACNA1S | 0.791 | KCNQ1 | CACNB2 | 0.632 | SMAD3 | CREB1 | 0.519 |
| CAV3 | RYR2 | 0.89 | KCNQ1 | CAV3 | 0.665 | SMAD3 | PPARA | 0.52 |
| CREB1 | MAPK1 | 0.972 | KCNQ1 | SCN4B | 0.683 | SMAD3 | FN1 | 0.766 |
| CREB1 | PIK3CA | 0.926 | KCNQ1 | RYR2 | 0.69 | SMAD3 | FKBP1A | 0.926 |
| CREB1 | FGF2 | 0.543 | KCNQ1 | KCNA5 | 0.71 |  |  |  |

**Table S9** ‘FSM - compounds – arrhythmia– targets’ network node

| #node | node_degree | related FSM compounds |
| --- | --- | --- |
| RYR2 | 33 | Vitamin A acid,Palmitic Acid,lauric acid,caprylic acid |
| FN1 | 32 | Vitamin A acid |
| KCNH2 | 29 | Adenosine |
| CREB1 | 28 | Adenosine,Pachymic acid |
| NOTCH1 | 28 | Vitamin A acid |
| PPARA | 27 | Vitamin A acid,Adenosine,lauric acid |
| CAV3 | 27 | caprylic acid,lauric acid,Palmitic Acid |
| CACNB2 | 26 | Adenosine,Pachymic acid |
| FGF2 | 26 | Vitamin A acid |
| KCNQ1 | 26 | Palmitic Acid,lauric acid,caprylic acid |
| MAPK1 | 26 | Adenosine,Pachymic acid |
| CACNA1D | 25 | Palmitic Acid,lauric acid,caprylic acid |
| IGF1 | 25 | Vitamin A acid |
| CACNA2D1 | 24 | Palmitic Acid,lauric acid,caprylic acid |
| SMAD3 | 22 | Vitamin A acid,Adenosine |
| PDE4D | 22 | Adenosine,Ergotamine |
| PIK3CA | 22 | Adenosine,Pachymic acid |
| RYR1 | 22 | Adenosine,Pachymic acid |
| CASQ2 | 20 | Palmitic Acid,lauric acid,caprylic acid |
| GNAS | 20 | Palmitic Acid,lauric acid,caprylic acid |
| KCNA5 | 20 | caprylic acid,lauric acid,Palmitic Acid,Vitamin A acid |
| SCN1B | 20 | Palmitic Acid,lauric acid,caprylic acid |
| ADIPOQ | 18 | Vitamin A acid |
| CACNA1S | 18 | Adenosine,Pachymic acid |
| CAMK2D | 18 | Palmitic Acid,lauric acid,caprylic acid |
| F2 | 18 | Pinocembrin |
| GNB5 | 18 | Ergotamine |
| KCNJ11 | 18 | Adenosine |
| ACADM | 17 | L-uridine,caprylic acid,Palmitic Acid,lauric acid |
| AGT | 17 | Adenosine |
| FKBP1A | 17 | Vitamin A acid |
| SCN4B | 17 | Lauric Aldehyde,Dehydroeburicoic acid,lauric acid,caprylic acid,Palmitic Acid |
| SDHA | 17 | Dehydroeburicoic acid,caprylic acid,lauric acid,Palmitic Acid |

**Table S10** GO analysis for 33 candidate targets (*p*.adjust value <0.05, arrhythmia related top terms)

| ONTOLOGY | ID | Description | GeneRatio | BgRatio | pvalue | p.adjust | qvalue | geneID | Count |
| --- | --- | --- | --- | --- | --- | --- | --- | --- | --- |
| BP | GO:0003012 | muscle system process | 21/33 | 465/18670 | 3.62E-26 | 8.32E-23 | 3.5077E-23 | AGT/CACNA1D/CACNA1S/CACNA2D1/CACNB2/CAMK2D/CASQ2/CAV3/IGF1/KCNA5/KCNH2/KCNQ1/SMAD3/NOTCH1/PDE4D/PIK3CA/PPARA/RYR1/RYR2/SCN1B/SCN4B | 21 |
| BP | GO:0060047 | heart contraction | 18/33 | 280/18670 | 7.21E-25 | 5.53E-22 | 2.33008E-22 | AGT/CACNA1D/CACNA1S/CACNA2D1/CACNB2/CAMK2D/CASQ2/CAV3/KCNA5/KCNH2/KCNJ11/KCNQ1/PDE4D/PIK3CA/RYR1/RYR2/SCN1B/SCN4B | 18 |
| BP | GO:0003015 | heart process | 18/33 | 290/18670 | 1.37E-24 | 7.89E-22 | 3.32608E-22 | AGT/CACNA1D/CACNA1S/CACNA2D1/CACNB2/CAMK2D/CASQ2/CAV3/KCNA5/KCNH2/KCNJ11/KCNQ1/PDE4D/PIK3CA/RYR1/RYR2/SCN1B/SCN4B | 18 |
| BP | GO:0008016 | regulation of heart contraction | 17/33 | 251/18670 | 8.54E-24 | 3.27E-21 | 1.38061E-21 | AGT/CACNA1D/CACNA1S/CACNA2D1/CACNB2/CAMK2D/CASQ2/CAV3/KCNA5/KCNH2/KCNJ11/KCNQ1/PDE4D/RYR1/RYR2/SCN1B/SCN4B | 17 |
| BP | GO:1903522 | regulation of blood circulation | 17/33 | 297/18670 | 1.57E-22 | 4.51E-20 | 1.90167E-20 | AGT/CACNA1D/CACNA1S/CACNA2D1/CACNB2/CAMK2D/CASQ2/CAV3/KCNA5/KCNH2/KCNJ11/KCNQ1/PDE4D/RYR1/RYR2/SCN1B/SCN4B | 17 |
| CC | GO:0034703 | cation channel complex | 15/33 | 220/19717 | 2.78E-21 | 3.69E-19 | 1.95711E-19 | CACNA1D/CACNA1S/CACNA2D1/CACNB2/CASQ2/FKBP1A/KCNA5/KCNH2/KCNJ11/KCNQ1/PDE4D/RYR1/RYR2/SCN1B/SCN4B | 15 |
| CC | GO:0034702 | ion channel complex | 15/33 | 301/19717 | 3.26E-19 | 2.17E-17 | 1.14885E-17 | CACNA1D/CACNA1S/CACNA2D1/CACNB2/CASQ2/FKBP1A/KCNA5/KCNH2/KCNJ11/KCNQ1/PDE4D/RYR1/RYR2/SCN1B/SCN4B | 15 |
| CC | GO:1902495 | transmembrane transporter complex | 15/33 | 324/19717 | 9.89E-19 | 4.38E-17 | 2.32421E-17 | CACNA1D/CACNA1S/CACNA2D1/CACNB2/CASQ2/FKBP1A/KCNA5/KCNH2/KCNJ11/KCNQ1/PDE4D/RYR1/RYR2/SCN1B/SCN4B | 15 |
| CC | GO:1990351 | transporter complex | 15/33 | 332/19717 | 1.43E-18 | 4.74E-17 | 2.51599E-17 | CACNA1D/CACNA1S/CACNA2D1/CACNB2/CASQ2/FKBP1A/KCNA5/KCNH2/KCNJ11/KCNQ1/PDE4D/RYR1/RYR2/SCN1B/SCN4B | 15 |
| CC | GO:0034704 | calcium channel complex | 9/33 | 66/19717 | 1.08E-15 | 2.88E-14 | 1.5268E-14 | CACNA1D/CACNA1S/CACNA2D1/CACNB2/CASQ2/FKBP1A/PDE4D/RYR1/RYR2 | 9 |
| MF | GO:0005261 | cation channel activity | 12/33 | 319/17697 | 2.42E-13 | 1.64E-11 | 7.81716E-12 | CACNA1D/CACNA1S/CACNA2D1/CACNB2/KCNA5/KCNH2/KCNJ11/KCNQ1/RYR1/RYR2/SCN1B/SCN4B | 12 |
| MF | GO:0022839 | ion gated channel activity | 12/33 | 334/17697 | 4.17E-13 | 2.12E-11 | 1.01033E-11 | CACNA1D/CACNA1S/CACNA2D1/CACNB2/KCNA5/KCNH2/KCNJ11/KCNQ1/RYR1/RYR2/SCN1B/SCN4B | 12 |
| MF | GO:0022836 | gated channel activity | 12/33 | 343/17697 | 5.71E-13 | 2.32E-11 | 1.10682E-11 | CACNA1D/CACNA1S/CACNA2D1/CACNB2/KCNA5/KCNH2/KCNJ11/KCNQ1/RYR1/RYR2/SCN1B/SCN4B | 12 |
| MF | GO:0044325 | ion channel binding | 9/33 | 124/17697 | 1.02E-12 | 3.43E-11 | 1.63863E-11 | CAMK2D/CAV3/FKBP1A/KCNJ11/KCNQ1/PDE4D/RYR2/SCN1B/SCN4B | 9 |
| MF | GO:0005216 | ion channel activity | 12/33 | 416/17697 | 5.52E-12 | 1.4E-10 | 6.68585E-11 | CACNA1D/CACNA1S/CACNA2D1/CACNB2/KCNA5/KCNH2/KCNJ11/KCNQ1/RYR1/RYR2/SCN1B/SCN4B | 12 |
| KEGG | hsa04261 | Adrenergic signaling in cardiomyocytes | 13/30 | 150/8076 | 1.69E-15 | 3.15E-13 | 1.12185E-13 | AGT/CACNA1D/CACNA1S/CACNA2D1/CACNB2/CAMK2D/CREB1/GNAS/KCNQ1/MAPK1/RYR2/SCN1B/SCN4B | 13 |
| KEGG | hsa05414 | Dilated cardiomyopathy | 8/30 | 96/8076 | 1.4E-09 | 9.44E-08 | 3.36603E-08 | AGT/CACNA1D/CACNA1S/CACNA2D1/CACNB2/GNAS/IGF1/RYR2 | 8 |
| KEGG | hsa04713 | Circadian entrainment | 8/30 | 97/8076 | 1.52E-09 | 9.44E-08 | 3.36603E-08 | CACNA1D/CAMK2D/CREB1/GNAS/MAPK1/RYR1/RYR2/GNB5 | 8 |
| KEGG | hsa04921 | Oxytocin signaling pathway | 9/30 | 154/8076 | 2.68E-09 | 1.07E-07 | 3.79938E-08 | CACNA1D/CACNA1S/CACNA2D1/CACNB2/CAMK2D/GNAS/MAPK1/RYR1/RYR2 | 9 |

**Table S11** ‘FSM - compounds – arrhythmia– targets’ network node

| Group | Name | Degree | ClosenessCentrality | NeighborhoodConnectivity | AverageShortestPathLength | BetweennessCentrality |
| --- | --- | --- | --- | --- | --- | --- |
| HERB | FSM | 11 | 0.473282 | 7.818182 | 2.112903 | 0.06248 |
| compound | Adenosine | 13 | 0.553571 | 20.84615 | 1.806452 | 0.025628 |
| compound | caprylic acid | 14 | 0.53913 | 23.64286 | 1.854839 | 0.02008 |
| compound | Dehydroeburicoic acid | 3 | 0.433566 | 15.66667 | 2.306452 | 0.001802 |
| compound | Ergotamine | 3 | 0.424658 | 14.33333 | 2.354839 | 0.0029 |
| compound | lauric acid | 15 | 0.558559 | 22.93333 | 1.790323 | 0.025235 |
| compound | Lauric Aldehyde | 2 | 0.421769 | 20.5 | 2.370968 | 6.89E-04 |
| compound | L-uridine | 2 | 0.352273 | 9 | 2.83871 | 5.02E-04 |
| compound | Pachymic acid | 7 | 0.516667 | 21.85714 | 1.935484 | 0.007978 |
| compound | Pinocembrin | 2 | 0.354286 | 9.5 | 2.822581 | 0.001957 |
| compound | Vitamin A acid | 11 | 0.54386 | 15.36364 | 1.83871 | 0.039379 |
| compound | Palmitic Acid | 14 | 0.53913 | 23.64286 | 1.854839 | 0.02008 |
| TAR | RYR2 | 54 | 0.696629 | 19.47368 | 1.435484 | 0.05262 |
| TAR | FN1 | 19 | 0.430556 | 12.3 | 2.322581 | 0.002141 |
| TAR | CREB1 | 28 | 0.525424 | 13.5 | 1.903226 | 0.021122 |
| TAR | KCNH2 | 40 | 0.590476 | 22.14815 | 1.693548 | 0.00888 |
| TAR | NOTCH1 | 20 | 0.476923 | 13.63636 | 2.096774 | 0.012067 |
| TAR | PPARA | 21 | 0.5 | 12.92308 | 2 | 0.020413 |
| TAR | CAV3 | 37 | 0.579439 | 23.86957 | 1.725806 | 0.008931 |
| TAR | CACNB2 | 52 | 0.645833 | 19.91429 | 1.548387 | 0.035868 |
| TAR | FGF2 | 19 | 0.424658 | 12.3 | 2.354839 | 0.002259 |
| TAR | KCNQ1 | 42 | 0.62 | 20.2 | 1.612903 | 0.024309 |
| TAR | MAPK1 | 31 | 0.53913 | 12.55556 | 1.854839 | 0.027485 |
| TAR | CACNA1D | 48 | 0.632653 | 19.02941 | 1.580645 | 0.033671 |
| TAR | IGF1 | 24 | 0.484375 | 13.07692 | 2.064516 | 0.005854 |
| TAR | CACNA2D1 | 49 | 0.645833 | 20.20588 | 1.548387 | 0.024917 |
| TAR | SMAD3 | 23 | 0.508197 | 14.15385 | 1.967742 | 0.0077 |
| TAR | PDE4D | 36 | 0.590476 | 19.2 | 1.693548 | 0.050042 |
| TAR | PIK3CA | 26 | 0.529915 | 13.9375 | 1.887097 | 0.017317 |
| TAR | RYR1 | 37 | 0.584906 | 19.40741 | 1.709677 | 0.017511 |
| TAR | CASQ2 | 37 | 0.579439 | 21.36 | 1.725806 | 0.025919 |
| TAR | GNAS | 26 | 0.558559 | 15.3125 | 1.790323 | 0.03329 |
| TAR | KCNA5 | 37 | 0.601942 | 20.2963 | 1.66129 | 0.023584 |
| TAR | SCN1B | 40 | 0.607843 | 19.93103 | 1.645161 | 0.015774 |
| TAR | ADIPOQ | 13 | 0.416107 | 12.71429 | 2.403226 | 7.34E-04 |
| TAR | CACNA1S | 41 | 0.596154 | 18.23333 | 1.677419 | 0.027456 |
| TAR | CAMK2D | 30 | 0.584906 | 21.2381 | 1.709677 | 0.016019 |
| TAR | F2 | 15 | 0.427586 | 12.25 | 2.33871 | 0.012641 |
| TAR | GNB5 | 13 | 0.492063 | 21.71429 | 2.032258 | 0.006017 |
| TAR | KCNJ11 | 34 | 0.553571 | 20.91667 | 1.806452 | 0.008484 |
| TAR | ACADM | 10 | 0.459259 | 12.71429 | 2.177419 | 0.018242 |
| TAR | AGT | 25 | 0.504065 | 14.4375 | 1.983871 | 0.031392 |
| TAR | FKBP1A | 25 | 0.53913 | 21.3125 | 1.854839 | 0.019239 |
| TAR | SCN4B | 40 | 0.613861 | 18.6 | 1.629032 | 0.058505 |
| TAR | SDHA | 8 | 0.410596 | 10.66667 | 2.435484 | 0.005337 |
| BP | BP_GO:0003012 | 21 | 0.584906 | 24.61905 | 1.709677 | 0.046433 |
| BP | BP_GO:0060047 | 18 | 0.563636 | 27.27778 | 1.774194 | 0.00858 |
| BP | BP_GO:0003015 | 18 | 0.563636 | 27.27778 | 1.774194 | 0.00858 |
| BP | BP_GO:0008016 | 17 | 0.548673 | 27.94118 | 1.822581 | 0.00534 |
| BP | BP_GO:1903522 | 17 | 0.548673 | 27.94118 | 1.822581 | 0.00534 |
| CC | CC_GO:0034703 | 15 | 0.508197 | 28.73333 | 1.967742 | 0.001765 |
| CC | CC_GO:0034702 | 15 | 0.508197 | 28.73333 | 1.967742 | 0.001765 |
| CC | CC_GO:1902495 | 15 | 0.508197 | 28.73333 | 1.967742 | 0.001765 |
| CC | CC_GO:1990351 | 15 | 0.508197 | 28.73333 | 1.967742 | 0.001765 |
| CC | CC_GO:0034704 | 9 | 0.476923 | 29.33333 | 2.096774 | 2.61E-04 |
| MF | MF_GO:0005261 | 12 | 0.469697 | 30.41667 | 2.129032 | 5.39E-04 |
| MF | MF_GO:0022839 | 12 | 0.469697 | 30.41667 | 2.129032 | 5.39E-04 |
| MF | MF_GO:0022836 | 12 | 0.469697 | 30.41667 | 2.129032 | 5.39E-04 |
| MF | MF_GO:0044325 | 9 | 0.469697 | 26.22222 | 2.129032 | 0.001101 |
| MF | MF_GO:0005216 | 12 | 0.469697 | 30.41667 | 2.129032 | 5.39E-04 |
| KEGG | KEGG_hsa04261 | 13 | 0.525424 | 26.69231 | 1.903226 | 0.009768 |
| KEGG | KEGG_hsa04921 | 9 | 0.496 | 28.11111 | 2.016129 | 0.002465 |
| KEGG | KEGG_hsa04024 | 10 | 0.516667 | 22.7 | 1.935484 | 0.004188 |

**Table S11** Most enrichment targets and compounds from ‘FSM - compounds – arrhythmia– targets’ network

| Group | Name | Degree | ClosenessCentrality | NeighborhoodConnectivity | AverageShortestPathLength | BetweennessCentrality |
| --- | --- | --- | --- | --- | --- | --- |
| HERB | FSM | 5 | 0.4 | 2.4 | 2.5 | 0.024102564 |
| compound | Palmitic Acid | 3 | 0.530612 | 16.33333 | 1.884615385 | 0.018534799 |
| compound | Vitamin A acid | 2 | 0.52 | 14 | 1.923076923 | 0.00952381 |
| compound | Pachymic acid | 2 | 0.509804 | 13 | 1.961538462 | 0.009010989 |
| compound | Caprylic acid | 3 | 0.530612 | 16.33333 | 1.884615385 | 0.018534799 |
| compound | Adenosine | 2 | 0.509804 | 13 | 1.961538462 | 0.009010989 |
| TAR | RYR2 | 25 | 0.896552 | 4.434783 | 1.115384615 | 0.356776557 |
| TAR | CACNA1S | 23 | 0.83871 | 4.714286 | 1.192307692 | 0.279047619 |
| TAR | CACNA1D | 23 | 0.83871 | 4.809524 | 1.192307692 | 0.210842491 |
| BP | BP_GO:1903522 | 3 | 0.52 | 21.66667 | 1.923076923 | 0 |
| BP | BP_GO:0008016 | 3 | 0.52 | 21.66667 | 1.923076923 | 0 |
| BP | BP_GO:0003015 | 3 | 0.52 | 21.66667 | 1.923076923 | 0 |
| BP | BP_GO:0060047 | 3 | 0.52 | 21.66667 | 1.923076923 | 0 |
| BP | BP_GO:0003012 | 3 | 0.52 | 21.66667 | 1.923076923 | 0 |
| CC | CC_GO:0042383 | 3 | 0.52 | 21.66667 | 1.923076923 | 0 |
| CC | CC_GO:1990351 | 3 | 0.52 | 21.66667 | 1.923076923 | 0 |
| CC | CC_GO:1902495 | 3 | 0.52 | 21.66667 | 1.923076923 | 0 |
| CC | CC_GO:0034702 | 3 | 0.52 | 21.66667 | 1.923076923 | 0 |
| CC | CC_GO:0034703 | 3 | 0.52 | 21.66667 | 1.923076923 | 0 |
| MF | MF_GO:0005216 | 3 | 0.52 | 21.66667 | 1.923076923 | 0 |
| MF | MF_GO:0044325 | 1 | 0.481481 | 23 | 2.076923077 | 0 |
| MF | MF_GO:0022836 | 3 | 0.52 | 21.66667 | 1.923076923 | 0 |
| MF | MF_GO:0022839 | 3 | 0.52 | 21.66667 | 1.923076923 | 0 |
| MF | MF_GO:0005261 | 3 | 0.52 | 21.66667 | 1.923076923 | 0 |
| KEGG | KEGG_hsa04260 | 3 | 0.52 | 21.66667 | 1.923076923 | 0 |
| KEGG | KEGG_hsa04024 | 3 | 0.52 | 21.66667 | 1.923076923 | 0 |
| KEGG | KEGG_hsa04261 | 3 | 0.52 | 21.66667 | 1.923076923 | 0 |
