# Supplementary material for: Fushenmu treatment ameliorates RyR2 with related metabolites in a zebrafish model of barium chloride induced arrhythmia
Source: Chin Med. 2023 Aug 19;18:103. doi: 10.1186/s13020-023-00812-x (PMC10439546; doi:10.1186/s13020-023-00812-x)
Supplement: Supplementary file 4 — Additional file 4: Table S13. FSM effects on BaCl2 induced pericardia edema and cardiomyocytes apoptosis (n = 6, mean ± s.d.). [file 13020_2023_812_MOESM4_ESM.docx]

**Additional File 4 for protective action of FSM against the BaCl_2_ induced arrhythmia phenotypes**

**Table S13** FSM effects on BaCl_2_ induced pericardia edema and cardiomyocytes apoptosis (n=6, mean ± s.d.)

|  | **Control** | **Model** | **Positive** | **FSM-H** | **FSM-M** | **FSM-L** |
| --- | --- | --- | --- | --- | --- | --- |
| SVBA | 141.57±3.64 | 223.23±4.94** | 155.61±5.62^NA,##^ | 159.25±2.99^**,##^ | 164.23±3.46^**,##^ | 175.98±3.11^**,##^ |
| Area | 3754.67±542.44 | 32035.67±495.26^**^ | 4761.83±1011.44^**,##^ | 6737.83±419.48^**,##^ | 8178.17±557.52^**,##^ | 12232.83±2064.27^**,##^ |
| AO Staining Density | 0.11±0.01 | 0.19±0.02^**^ | 0.13±0.01^NA,##^ | 0.13±0.01^*,##^ | 0.14±0.02^*,##^ | 0.16±0.01^**,##^ |

*p < 0.05, **p < 0.005 compared with control gruop; #p < 0.05, ##p < 0.005 compared with model group. NS, not significant.
