# Supplementary material for: Fushenmu treatment ameliorates RyR2 with related metabolites in a zebrafish model of barium chloride induced arrhythmia
Source: Chin Med. 2023 Aug 19;18:103. doi: 10.1186/s13020-023-00812-x (PMC10439546; doi:10.1186/s13020-023-00812-x)
Supplement: Supplementary file 5 — Additional file 5: Table S14. Biomarkers selection between control group and model group from negative ion mode (p < 0.05, (|Log2-fold change |> 1, VIP > 1). Table S15. Biomarkers selection between control group and model group from positive ion mode (p < 0.05, (|Log2-fold change |> 1, VIP > 1). Table S16. FSM rescued effects among the control, model and treatment groups from negative mode. Table S17. FSM rescued effects among the control, model and treatment groups from positive mode. Table S18. 194 FSM therapeutic markers. Table S19. Pathway analysis from the FSM therapeutic metabolomic markers (Top 10 pathway, the pathway of either negative or positive ion metabolite markers with the cutoff value of Holm p < 0.05 is highlighted in yellow. The pathway of combined metabolite markers with the cutoff value of Holm p < 0.05 and Impact > 0.1 is highlighted in yellow). Figure S2. Pathway analysis of FSM therapeutic markers of (A) negative ion and (B) positive ion. [file 13020_2023_812_MOESM5_ESM.docx]

**Additional File 5 for metabolomic analysis**

**Untargeted metabolomic analysis**

**Table S14 Biomarkers selection between control group and model group from negative ion mode (p<0.05, (|Log2-fold change | > 1, VIP>1).**

| **p<0.05** | | | |
| --- | --- | --- | --- |
| **Metabolites** | | **t.stat** | **p.value** |
| D-Glutamine | | 114.11 | 1.01E-24 |
| L-Glutamine | | 114.11 | 1.01E-24 |
| L-Phenylalanine | | 109.39 | 1.99E-24 |
| 3,4-Dihydroxybenzeneacetic acid | | -102.94 | 5.24E-24 |
| Homogentisic acid | | -102.94 | 5.24E-24 |
| Vanillic acid | | -102.94 | 5.24E-24 |
| L-Tryptophan | | 94.925 | 1.91E-23 |
| 7a-Hydroxy-oxo-5b-cholanoic acid | | 82.298 | 1.87E-22 |
| Fructose 6-phosphate | | 80.081 | 2.89E-22 |
| Glucose 1-phosphate | | 80.081 | 2.89E-22 |
| Glucose 6-phosphate | | 80.081 | 2.89E-22 |
| Mannose 6-phosphate | | 80.081 | 2.89E-22 |
| Chenodeoxycholic acid | | 79.973 | 2.96E-22 |
| Deoxycholic acid | | 79.973 | 2.96E-22 |
| Hyodeoxycholic acid | | 79.973 | 2.96E-22 |
| Ursodeoxycholic acid | | 79.973 | 2.96E-22 |
| 2,2-Dimethylsuccinic acid | | 66.396 | 5.75E-21 |
| 2-Methylglutaric acid | | 66.396 | 5.75E-21 |
| Methylglutaric acid | | 66.396 | 5.75E-21 |
| Monomethyl glutaric acid | | 66.396 | 5.75E-21 |
| Gluconolactone | | 65.831 | 6.59E-21 |
| Allose | | 62.112 | 1.67E-20 |
| Alpha-D-Glucose | | 62.112 | 1.67E-20 |
| D-Fructose | | 62.112 | 1.67E-20 |
| D-Galactose | | 62.112 | 1.67E-20 |
| D-Glucose1 | | 62.112 | 1.67E-20 |
| D-Mannose | | 62.112 | 1.67E-20 |
| D-Tagatose | | 62.112 | 1.67E-20 |
| L-Sorbose | | 62.112 | 1.67E-20 |
| Myoinositol | | 62.112 | 1.67E-20 |
| Scyllitol | | 62.112 | 1.67E-20 |
| Glycerol 3-phosphate | | 58.147 | 4.77E-20 |
| Theobromine | | 57.291 | 6.04E-20 |
| Indoxyl | | -55.914 | 8.89E-20 |
| Indole-carbinol | | -55.334 | 1.05E-19 |
| Flavin Mononucleotide | | 53.922 | 1.58E-19 |
| 3-Phenoxypropionic acid | | -52.922 | 2.13E-19 |
| Desaminotyrosine | | -52.922 | 2.13E-19 |
| L-Phenyllactic acid | | -52.922 | 2.13E-19 |
| Canrenone | | 50.218 | 4.92E-19 |
| L-Glutamic acid | | 50.188 | 4.96E-19 |
| L-Tyrosine | | 46.478 | 1.68E-18 |
| o-Tyrosine | | 46.478 | 1.68E-18 |
| dCMP | | 45.117 | 2.70E-18 |
| 4-Hydroxyphenylpyruvic acid | | 44.243 | 3.68E-18 |
| Caffeic acid | | 44.243 | 3.68E-18 |
| Azelaic acid | | 44.034 | 3.97E-18 |
| Nicotinuric acid | | 43.859 | 4.23E-18 |
| Niacinamide | | -41.996 | 8.42E-18 |
| Arachidic acid | | 41.916 | 8.68E-18 |
| 5-Methoxytryptophan | | -41.836 | 8.94E-18 |
| Hippuric acid | | 40.59 | 1.44E-17 |
| Fumaric acid | | -39.826 | 1.95E-17 |
| Elaidic acid | | 38.643 | 3.15E-17 |
| Oleic acid | | 38.643 | 3.15E-17 |
| Vaccenic acid | | 38.643 | 3.15E-17 |
| Nutriacholic acid | | 38.112 | 3.92E-17 |
| 3-Methylindole | | -37.513 | 5.04E-17 |
| 3-Methoxybenzenepropanoic acid | | -37.282 | 5.55E-17 |
| Rhamnose | | 36.2 | 8.85E-17 |
| Sebacic acid | | 35.94 | 9.92E-17 |
| Phenylpropiolic acid | | 35.849 | 1.03E-16 |
| Linoleic acid | | 35.44 | 1.24E-16 |
| 5-Phenylvaleric acid | | 34.9 | 1.58E-16 |
| Phosphocreatine | | 34.186 | 2.19E-16 |
| Guanidinosuccinic acid | | -33.536 | 2.96E-16 |
| Guanosine | | 33.387 | 3.18E-16 |
| FAD | | 33.24 | 3.41E-16 |
| 7-Methylxanthine | | 32.986 | 3.84E-16 |
| Indoleacetic acid | | -32.95 | 3.91E-16 |
| 3-Methoxytyrosine | | 31.548 | 7.76E-16 |
| ADP | | 31.265 | 8.95E-16 |
| Dodecanoic acid | | 31.155 | 9.46E-16 |
| 1,3-Dimethyluric acid | | 30.907 | 1.07E-15 |
| 1,9-Dimethyluric acid | | 30.907 | 1.07E-15 |
| L-Cystathionine | | -30.169 | 1.57E-15 |
| 6-Phosphogluconic acid | | 30.108 | 1.62E-15 |
| Menadione | | -29.93 | 1.78E-15 |
| Atenolol | | 29.361 | 2.41E-15 |
| Citric acid | | 28.962 | 2.98E-15 |
| D-threo-Isocitric acid | | 28.962 | 2.98E-15 |
| Isocitric acid | | 28.962 | 2.98E-15 |
| Galactonic acid | | 28.915 | 3.06E-15 |
| Gluconic acid | | 28.915 | 3.06E-15 |
| Alpha-Linolenic acid | | 28.79 | 3.28E-15 |
| Quinic acid | | -28.498 | 3.85E-15 |
| Deoxyadenosine monophosphate | | 27.753 | 5.83E-15 |
| 1-Methylguanine | | 27.493 | 6.76E-15 |
| 7-Methylguanine | | 27.493 | 6.76E-15 |
| Beta-Alanine | | -27.452 | 6.92E-15 |
| D-Alanine | | -27.452 | 6.92E-15 |
| L-Alanine | | -27.452 | 6.92E-15 |
| Sarcosine | | -27.452 | 6.92E-15 |
| 11a-Hydroxyprogesterone | | 27.283 | 7.62E-15 |
| 17-Hydroxyprogesterone | | 27.283 | 7.62E-15 |
| Deoxycorticosterone | | 27.283 | 7.62E-15 |
| Inosine | | 26.948 | 9.25E-15 |
| Adenosine 2',3'-cyclic phosphate | | 26.711 | 1.06E-14 |
| Cyclic AMP | | 26.711 | 1.06E-14 |
| Biotin | | -26.388 | 1.29E-14 |
| Guanosine diphosphate | | 26.361 | 1.31E-14 |
| 2-Isopropylmalic acid | | -26.088 | 1.54E-14 |
| L-Proline | | 25.846 | 1.78E-14 |
| Pantothenic acid | | 25.558 | 2.12E-14 |
| N-Acetylneuraminic acid | | 25.404 | 2.33E-14 |
| Alpha-Aspartyl-lysine | | -25.029 | 2.94E-14 |
| Myristic acid | | 24.998 | 3.00E-14 |
| Stearic acid | | 24.981 | 3.03E-14 |
| Phenylacetylglycine | | 24.86 | 3.27E-14 |
| Uridine 5'-monophosphate | | 24.556 | 3.96E-14 |
| Aminocaproic acid | | 24.552 | 3.97E-14 |
| Beta-Leucine | | 24.552 | 3.97E-14 |
| L-Alloisoleucine | | 24.552 | 3.97E-14 |
| L-Isoleucine | | 24.552 | 3.97E-14 |
| L-Leucine | | 24.552 | 3.97E-14 |
| L-Norleucine | | 24.552 | 3.97E-14 |
| Inosinic acid | | 24.297 | 4.67E-14 |
| N-Acetylserotonin | | -24.252 | 4.81E-14 |
| 3-Methyladenine | | -23.105 | 1.02E-13 |
| 6-Methyladenine | | -23.105 | 1.02E-13 |
| Pentadecanoic acid | | 22.715 | 1.33E-13 |
| D-Arginine | | 22.292 | 1.79E-13 |
| L-Arginine | | 22.292 | 1.79E-13 |
| Heptadecanoic acid | | 22.153 | 1.97E-13 |
| 2-Methylhippuric acid | | 21.972 | 2.23E-13 |
| Phenylglyoxylic acid | | 21.832 | 2.47E-13 |
| Melatonin | | -19.735 | 1.17E-12 |
| NADH | | 19.662 | 1.24E-12 |
| Uracil | | -19.566 | 1.34E-12 |
| 13-cis-Retinoic acid | | 19.486 | 1.43E-12 |
| All-trans-retinoic acid | | 19.486 | 1.43E-12 |
| Alpha-Hydroxyhippuric acid | | 19.178 | 1.83E-12 |
| Glycyl-glycine | | 18.684 | 2.73E-12 |
| L-Asparagine | | 18.684 | 2.73E-12 |
| Homovanillic acid | | -18.33 | 3.65E-12 |
| Hydroxyphenyllactic acid | | -18.33 | 3.65E-12 |
| Uridine diphosphate glucose | | 18.129 | 4.33E-12 |
| Oxoglutaric acid | | 17.988 | 4.87E-12 |
| Adenosine triphosphate | | 17.864 | 5.42E-12 |
| dGTP | | 17.864 | 5.42E-12 |
| Cholesterol sulfate | | 17.61 | 6.74E-12 |
| Ribonolactone | | 17.528 | 7.24E-12 |
| Galacturonic acid | | -17.423 | 7.94E-12 |
| Lipoamide | | 17.415 | 7.99E-12 |
| L-Aspartyl-L-phenylalanine | | 16.853 | 1.32E-11 |
| 1,11-Undecanedicarboxylic acid | | 16.834 | 1.34E-11 |
| N-Acetyl-D-glucosamine | | 16.585 | 1.68E-11 |
| N-Acetylgalactosamine | | 16.585 | 1.68E-11 |
| Cytidine triphosphate | | 16.572 | 1.70E-11 |
| 5-Aminopentanoic acid | | 15.956 | 3.02E-11 |
| Betaine | | 15.956 | 3.02E-11 |
| L-Valine | | 15.956 | 3.02E-11 |
| N-Methyl-a-aminoisobutyric acid | | 15.956 | 3.02E-11 |
| Xanthosine | | 15.806 | 3.48E-11 |
| L-Acetylcarnitine | | -15.68 | 3.92E-11 |
| 2-Methoxyestradiol | | -15.544 | 4.47E-11 |
| Uridine 5'-diphosphate | | 15.461 | 4.85E-11 |
| Malic acid | | 15.17 | 6.45E-11 |
| 4-Aminohippuric acid | | -14.416 | 1.38E-10 |
| D-Pantethine | | -14.278 | 1.60E-10 |
| D-Lysine | | 14.254 | 1.64E-10 |
| L-Lysine | | 14.254 | 1.64E-10 |
| L-Malic acid | | 14.228 | 1.68E-10 |
| Coproporphyrin I | | 14.078 | 1.97E-10 |
| Methylcysteine | | -13.588 | 3.33E-10 |
| Dodecanedioic acid | | 13.564 | 3.42E-10 |
| Ethenodeoxyadenosine | | 13.372 | 4.22E-10 |
| Lithocholic acid | | -13.346 | 4.35E-10 |
| D-Ribose 5-phosphate | | -12.859 | 7.51E-10 |
| Inosine triphosphate | | 12.2 | 1.62E-09 |
| Cortexolone | | -12.157 | 1.70E-09 |
| 3-Aminoisobutanoic acid | | -11.957 | 2.17E-09 |
| D-Alpha-aminobutyric acid | | -11.957 | 2.17E-09 |
| Dimethylglycine | | -11.957 | 2.17E-09 |
| Gamma-Aminobutyric acid | | -11.957 | 2.17E-09 |
| L-Alpha-aminobutyric acid | | -11.957 | 2.17E-09 |
| 2-Furoylglycine | | -11.893 | 2.34E-09 |
| Phloretin | | -11.816 | 2.57E-09 |
| Perillic acid | | -11.275 | 5.05E-09 |
| Pyroglutamic acid | | 11.213 | 5.46E-09 |
| Pyrrolidonecarboxylic acid | | 11.213 | 5.46E-09 |
| 3-Phosphoglyceric acid | | 10.935 | 7.82E-09 |
| Allantoic acid | | -10.855 | 8.67E-09 |
| 3-Oxocholic acid | | -10.848 | 8.75E-09 |
| Glycerophosphocholine | | -10.811 | 9.19E-09 |
| 9-Methyluric acid | | -10.514 | 1.36E-08 |
| Benzenebutanoic acid | | -10.344 | 1.71E-08 |
| Aspartame | | 10.201 | 2.08E-08 |
| Glucaric acid | | 10.111 | 2.35E-08 |
| N-Acetylmannosamine | | 10.027 | 2.65E-08 |
| Levulinic acid | | 9.9681 | 2.87E-08 |
| L-Methionine | | 9.8328 | 3.47E-08 |
| Raffinose | | -9.7274 | 4.03E-08 |
| 5-Thymidylic acid | | 9.6542 | 4.47E-08 |
| Taurine | | 9.4745 | 5.79E-08 |
| 4-Hydroxyproline | | 9.2735 | 7.76E-08 |
| 5-Aminolevulinic acid | | 9.2735 | 7.76E-08 |
| N-Acetyl-L-alanine | | 9.2735 | 7.76E-08 |
| Methionine sulfoxide | | -9.2613 | 7.90E-08 |
| D-Sorbitol | | 9.1229 | 9.70E-08 |
| Galactitol | | 9.1229 | 9.70E-08 |
| Mannitol | | 9.1229 | 9.70E-08 |
| Traumatic acid | | 8.9002 | 1.35E-07 |
| 5'-Methylthioadenosine | | 8.8859 | 1.38E-07 |
| 4-Hydroxycyclohexylcarboxylic acid | | -8.7755 | 1.64E-07 |
| 3-Indolepropionic acid | | -8.6949 | 1.85E-07 |
| Glycyl-L-leucine | | -8.6895 | 1.87E-07 |
| N-Alpha-acetyllysine | | -8.6895 | 1.87E-07 |
| Hematoporphyrin IX | | 8.6443 | 2.00E-07 |
| 2-Phenylglycine | | -8.5951 | 2.16E-07 |
| Indolelactic acid | | -8.5423 | 2.34E-07 |
| Phthalic acid | | -8.3844 | 3.00E-07 |
| Pipecolic acid | | 8.2118 | 3.94E-07 |
| N-Acetyl-L-phenylalanine | | -7.9182 | 6.34E-07 |
| Dopamine | | -7.8678 | 6.88E-07 |
| 16-Dehydroprogesterone | | -7.7848 | 7.88E-07 |
| Indoleacrylic acid | | -7.7143 | 8.86E-07 |
| Quinaldic acid | | -7.6446 | 9.95E-07 |
| 6-Dimethylaminopurine | | -7.6312 | 1.02E-06 |
| L-Histidine | | 7.6078 | 1.06E-06 |
| Shikimic acid | | -7.5511 | 1.16E-06 |
| Bilirubin | | -7.5116 | 1.24E-06 |
| FAPy-adenine | | -7.4277 | 1.43E-06 |
| 4-Methoxycinnamic acid | | -7.3449 | 1.65E-06 |
| L-Homoserine | | 7.3324 | 1.69E-06 |
| L-Threonine | | 7.3324 | 1.69E-06 |
| lipoic acid | | -7.273 | 1.87E-06 |
| Guanosine triphosphate | | 7.2491 | 1.94E-06 |
| 3-Chlorotyrosine | | -7.2462 | 1.95E-06 |
| Thymidine | | -7.1885 | 2.16E-06 |
| Cortisone | | -7.1411 | 2.34E-06 |
| Taurodeoxycholic acid | | -7.0072 | 2.96E-06 |
| m-Chlorobenzoic acid | | 7.0019 | 2.99E-06 |
| 3b-Hydroxy-5-cholenoic acid | | -6.8297 | 4.05E-06 |
| Maltotriose | | -6.8051 | 4.23E-06 |
| 2-Chlorobenzoic acid | | 6.7993 | 4.27E-06 |
| Glycocholic acid | | -6.7576 | 4.60E-06 |
| 2-Hydroxy-methylbutyric acid | | 6.6606 | 5.48E-06 |
| Ibuprofen | | -6.6567 | 5.51E-06 |
| Tryptamine | | 6.5711 | 6.43E-06 |
| Creatinine | | -6.5067 | 7.23E-06 |
| 2,5-Furandicarboxylic acid | | 6.4914 | 7.44E-06 |
| Homo-L-arginine | | -6.4084 | 8.66E-06 |
| Guaifenesin | | -6.4013 | 8.77E-06 |
| Phosphoenolpyruvic acid | | 6.347 | 9.69E-06 |
| Quinolinic acid | | -6.2157 | 1.24E-05 |
| Chlorogenic acid | | 6.2056 | 1.26E-05 |
| Methylmalonic acid | | 6.1561 | 1.38E-05 |
| Succinic acid | | 6.1561 | 1.38E-05 |
| Cholic acid | | -6.1482 | 1.40E-05 |
| D-Xylose | | -6.1484 | 1.40E-05 |
| Ketoleucine | | -6.1417 | 1.42E-05 |
| 2-Methyl-ketovaleric acid | | -6.0954 | 1.55E-05 |
| Acetylglycine | | 6.077 | 1.60E-05 |
| 2-Phenylbutyric acid | | -5.8731 | 2.36E-05 |
| 3-Phenylbutyric acid | | -5.8707 | 2.37E-05 |
| Glutathione | | 5.6462 | 3.65E-05 |
| Aminoadipic acid | | 5.4716 | 5.12E-05 |
| Diethylthiophosphate | | -5.4494 | 5.35E-05 |
| Corticosterone | | -5.3311 | 6.76E-05 |
| Dehydroascorbic acid | | 5.2752 | 7.55E-05 |
| trans-Aconitic acid | | 5.2752 | 7.55E-05 |
| Dimethylmalonic acid | | 5.219 | 8.44E-05 |
| Glutaric acid | | 5.219 | 8.44E-05 |
| Methylsuccinic acid | | 5.219 | 8.44E-05 |
| Monoethyl malonic acid | | 5.219 | 8.44E-05 |
| Erucic acid | | 5.1324 | 0.0001 |
| Danazol | | 5.1275 | 0.000101 |
| Genistein | | -5.1176 | 0.000103 |
| Glycylproline | | -4.9859 | 0.000135 |
| 17a-Ethynylestradiol | | -4.9654 | 0.00014 |
| 3-Cresotinic acid | | -4.9005 | 0.00016 |
| 4-Hydroxy-methylbenzoic acid | | -4.9005 | 0.00016 |
| Mandelic acid | | -4.9005 | 0.00016 |
| p-Anisic acid | | -4.9005 | 0.00016 |
| Allantoin | | -4.7519 | 0.000217 |
| 6-Hydroxynicotinic acid | | 4.693 | 0.000244 |
| Ascorbic acid | | 4.4768 | 0.000381 |
| D-Glucurono-6,3-lactone | | 4.4768 | 0.000381 |
| D-Lactic acid | | -4.2543 | 0.000606 |
| Hydroxypropionic acid | | -4.2543 | 0.000606 |
| Pregnenolone sulfate | | -4.1103 | 0.000819 |
| D-Xylulose | | -4.0777 | 0.000877 |
| N-Acetylglutamic acid | | -4.0749 | 0.000882 |
| Diaminopimelic acid | | 4.0319 | 0.000965 |
| Guanosine monophosphate | | 3.9255 | 0.001207 |
| 2,4-Dihydroxybenzoic acid | | -3.7761 | 0.001654 |
| 2,6-Dihydroxybenzoic acid | | -3.7761 | 0.001654 |
| 2-Pyrocatechuic acid | | -3.7761 | 0.001654 |
| Gentisic acid | | -3.7761 | 0.001654 |
| Protocatechuic acid | | -3.7761 | 0.001654 |
| Mesoporphyrin IX | | 3.7015 | 0.001936 |
| L-Serine | | -3.4869 | 0.003047 |
| Suberic acid | | 3.3522 | 0.004049 |
| Ornithine | | -3.1714 | 0.005923 |
| Citraconic acid | | 3.1077 | 0.00677 |
| Itaconic acid | | 3.1077 | 0.00677 |
| Mesaconic acid | | 3.1077 | 0.00677 |
| Gallic acid | | -3.0606 | 0.007472 |
| Alpha-Lactose | | -2.9701 | 0.009025 |
| Cellobiose | | -2.9701 | 0.009025 |
| Isomaltose | | -2.9701 | 0.009025 |
| Lactulose | | -2.9701 | 0.009025 |
| Melibiose | | -2.9701 | 0.009025 |
| Sucrose | | -2.9701 | 0.009025 |
| Trehalose | | -2.9701 | 0.009025 |
| Turanose | | -2.9701 | 0.009025 |
| L-Fucose | | 2.8777 | 0.010935 |
| Ureidosuccinic acid | | 2.661 | 0.017081 |
| Hesperidin | | 2.4279 | 0.027355 |
| Biocytin | | -2.418 | 0.027902 |
| **(\|Log2-fold change \| > 1** | | | |
| **Metabolites** | | **Fold Change** | **log2(FC)** |
| 7a-Hydroxy-oxo-5b-cholanoic acid | | 150.92 | 7.2377 |
| dCMP | | 137.76 | 7.106 |
| Chenodeoxycholic acid | | 125.33 | 6.9696 |
| Deoxycholic acid | | 125.33 | 6.9696 |
| Hyodeoxycholic acid | | 125.33 | 6.9696 |
| Ursodeoxycholic acid | | 125.33 | 6.9696 |
| Adenosine triphosphate | | 69.361 | 6.1161 |
| dGTP | | 69.361 | 6.1161 |
| 2,2-Dimethylsuccinic acid | | 56.584 | 5.8223 |
| 2-Methylglutaric acid | | 56.584 | 5.8223 |
| Methylglutaric acid | | 56.584 | 5.8223 |
| Monomethyl glutaric acid | | 56.584 | 5.8223 |
| 1-Methylguanine | | 35.462 | 5.1482 |
| 7-Methylguanine | | 35.462 | 5.1482 |
| Fructose 6-phosphate | | 35.183 | 5.1368 |
| Glucose 1-phosphate | | 35.183 | 5.1368 |
| Glucose 6-phosphate | | 35.183 | 5.1368 |
| Mannose 6-phosphate | | 35.183 | 5.1368 |
| Nutriacholic acid | | 33.877 | 5.0822 |
| Nicotinuric acid | | 33.175 | 5.052 |
| Sebacic acid | | 31.72 | 4.9873 |
| Cytidine triphosphate | | 30.293 | 4.9209 |
| 5-Phenylvaleric acid | | 29.048 | 4.8604 |
| L-Phenylalanine | | 26.982 | 4.7539 |
| FAD | | 23.594 | 4.5603 |
| Canrenone | | 20.937 | 4.388 |
| Inosine triphosphate | | 20.36 | 4.3476 |
| Uridine diphosphate glucose | | 20.017 | 4.3232 |
| L-Aspartyl-L-phenylalanine | | 19.499 | 4.2853 |
| Deoxyadenosine monophosphate | | 18.24 | 4.1891 |
| Gluconolactone | | 18.061 | 4.1748 |
| Arachidic acid | | 17.815 | 4.155 |
| D-Glutamine | | 17.231 | 4.107 |
| L-Glutamine | | 17.231 | 4.107 |
| Pentadecanoic acid | | 16.13 | 4.0117 |
| trans-Aconitic acid | | 15.367 | 3.9418 |
| m-Chlorobenzoic acid | | 14.996 | 3.9065 |
| Dehydroascorbic acid | | 14.012 | 3.8086 |
| Phenylacetylglycine | | 13.999 | 3.8073 |
| Alpha-Hydroxyhippuric acid | | 13.808 | 3.7874 |
| Allose | | 13.489 | 3.7537 |
| Alpha-D-Glucose | | 13.489 | 3.7537 |
| D-Fructose | | 13.489 | 3.7537 |
| D-Galactose | | 13.489 | 3.7537 |
| D-Glucose | | 13.489 | 3.7537 |
| D-Mannose | | 13.489 | 3.7537 |
| D-Tagatose | | 13.489 | 3.7537 |
| L-Sorbose | | 13.489 | 3.7537 |
| Myoinositol | | 13.489 | 3.7537 |
| Scyllitol | | 13.489 | 3.7537 |
| 4-Hydroxyphenylpyruvic acid | | 12.913 | 3.6907 |
| Caffeic acid | | 12.913 | 3.6907 |
| 2-Chlorobenzoic acid | | 12.767 | 3.6744 |
| Ascorbic acid | | 12.698 | 3.6665 |
| D-Glucurono-6,3-lactone | | 12.698 | 3.6665 |
| Inosinic acid | | 12.494 | 3.6431 |
| Alpha-Linolenic acid | | 11.86 | 3.568 |
| Myristic acid | | 11.83 | 3.5644 |
| Inosine | | 11.62 | 3.5385 |
| Glycerol 3-phosphate | | 11.602 | 3.5363 |
| Adenosine 2',3'-cyclic phosphate | | 11.547 | 3.5295 |
| Cyclic AMP | | 11.547 | 3.5295 |
| Stearic acid | | 10.803 | 3.4334 |
| Citric acid | | 9.85 | 3.3001 |
| D-threo-Isocitric acid | | 9.85 | 3.3001 |
| Isocitric acid | | 9.85 | 3.3001 |
| Phenylpropiolic acid | | 9.6778 | 3.2747 |
| Theobromine | | 9.6082 | 3.2643 |
| Dodecanoic acid | | 9.5236 | 3.2515 |
| Hippuric acid | | 9.1358 | 3.1915 |
| Aspartame | | 8.9302 | 3.1587 |
| NADH | | 8.8286 | 3.1422 |
| Linoleic acid | | 8.7007 | 3.1211 |
| 13-cis-Retinoic acid | | 8.4041 | 3.0711 |
| All-trans-retinoic acid | | 8.4041 | 3.0711 |
| Rhamnose | | 7.885 | 2.9791 |
| L-Tryptophan | | 7.6872 | 2.9424 |
| Uridine 5'-monophosphate | | 7.6285 | 2.9314 |
| Guanosine diphosphate | | 7.4426 | 2.8958 |
| Elaidic acid | | 7.0511 | 2.8178 |
| Oleic acid | | 7.0511 | 2.8178 |
| Vaccenic acid | | 7.0511 | 2.8178 |
| Uridine 5'-diphosphate | | 6.9638 | 2.7999 |
| L-Tyrosine | | 6.6886 | 2.7417 |
| o-Tyrosine | | 6.6886 | 2.7417 |
| 6-Hydroxynicotinic acid | | 6.6028 | 2.7231 |
| Oxoglutaric acid | | 6.3486 | 2.6664 |
| Phenylglyoxylic acid | | 5.764 | 2.5271 |
| Phosphocreatine | | 5.7284 | 2.5181 |
| Azelaic acid | | 5.6306 | 2.4933 |
| 6-Phosphogluconic acid | | 5.6013 | 2.4858 |
| Flavin Mononucleotide | | 5.5202 | 2.4647 |
| 11a-Hydroxyprogesterone | | 5.1334 | 2.3599 |
| 17-Hydroxyprogesterone | | 5.1334 | 2.3599 |
| Deoxycorticosterone | | 5.1334 | 2.3599 |
| 3-Methoxytyrosine | | 5.0382 | 2.3329 |
| Pantothenic acid | | 4.9766 | 2.3152 |
| N-Acetylneuraminic acid | | 4.8529 | 2.2788 |
| ADP | | 4.7178 | 2.2381 |
| Ribonolactone | | 4.4359 | 2.1492 |
| L-Glutamic acid | | 4.4289 | 2.147 |
| Dodecanedioic acid | | 4.3523 | 2.1218 |
| Galactonic acid | | 4.2594 | 2.0906 |
| Gluconic acid | | 4.2594 | 2.0906 |
| Glutathione | | 4.1054 | 2.0375 |
| Guanosine | | 4.0761 | 2.0272 |
| Heptadecanoic acid | | 4.0322 | 2.0116 |
| Cholesterol sulfate | | 3.8917 | 1.9604 |
| 1,3-Dimethyluric acid | | 3.8881 | 1.9591 |
| 1,9-Dimethyluric acid | | 3.8881 | 1.9591 |
| Lipoamide | | 3.7778 | 1.9175 |
| L-Proline | | 3.7362 | 1.9016 |
| Coproporphyrin I | | 3.614 | 1.8536 |
| D-Arginine | | 3.3767 | 1.7556 |
| L-Arginine | | 3.3767 | 1.7556 |
| 3-Phosphoglyceric acid | | 3.324 | 1.7329 |
| 5-Thymidylic acid | | 3.2632 | 1.7063 |
| Atenolol | | 3.2158 | 1.6852 |
| 7-Methylxanthine | | 3.0627 | 1.6148 |
| Xanthosine | | 3.0604 | 1.6137 |
| 2,5-Furandicarboxylic acid | | 3.0322 | 1.6004 |
| Guanosine triphosphate | | 2.9737 | 1.5723 |
| Glucaric acid | | 2.8696 | 1.5209 |
| 4-Hydroxyproline | | 2.8642 | 1.5181 |
| 5-Aminolevulinic acid | | 2.8642 | 1.5181 |
| N-Acetyl-L-alanine | | 2.8642 | 1.5181 |
| Ethenodeoxyadenosine | | 2.802 | 1.4864 |
| Traumatic acid | | 2.7793 | 1.4747 |
| L-Fucose | | 2.7727 | 1.4713 |
| Erucic acid | | 2.6888 | 1.4269 |
| Glycyl-glycine | | 2.553 | 1.3522 |
| L-Asparagine | | 2.553 | 1.3522 |
| 1,11-Undecanedicarboxylic acid | | 2.5443 | 1.3473 |
| Hematoporphyrin IX | | 2.5269 | 1.3373 |
| Malic acid | | 2.4989 | 1.3213 |
| D-Lysine | | 2.4774 | 1.3088 |
| L-Lysine | | 2.4774 | 1.3088 |
| L-Methionine | | 2.4425 | 1.2883 |
| 3-Hexenedioic acid | | 2.4372 | 1.2852 |
| Aminocaproic acid | | 2.4174 | 1.2734 |
| Beta-Leucine | | 2.4174 | 1.2734 |
| L-Alloisoleucine | | 2.4174 | 1.2734 |
| L-Isoleucine | | 2.4174 | 1.2734 |
| L-Leucine | | 2.4174 | 1.2734 |
| L-Norleucine | | 2.4174 | 1.2734 |
| Levulinic acid | | 2.4153 | 1.2722 |
| Phosphoenolpyruvic acid | | 2.361 | 1.2394 |
| Pyrrolidonecarboxylic acid | | 2.247 | 1.168 |
| Acetylglycine | | 2.2326 | 1.1587 |
| D-Sorbitol | | 2.2251 | 1.1539 |
| Galactitol | | 2.2251 | 1.1539 |
| Mannitol | | 2.2251 | 1.1539 |
| L-Malic acid | | 2.1579 | 1.1097 |
| 5'-Methylthioadenosine | | 2.1507 | 1.1048 |
| Pyroglutamic acid | | 2.1256 | 1.0879 |
| Pipecolic acid | | 2.0576 | 1.0409 |
| 5-Aminopentanoic acid | | 2.0547 | 1.0389 |
| Betaine | | 2.0547 | 1.0389 |
| L-Valine | | 2.0547 | 1.0389 |
| N-Methyl-a-aminoisobutyric acid | | 2.0547 | 1.0389 |
| Tryptamine | | 2.0345 | 1.0247 |
| 17a-Ethynylestradiol | | 0.42365 | -1.2391 |
| Glycerophosphocholine | | 0.41946 | -1.2534 |
| Allantoin | | 0.40658 | -1.2984 |
| Allantoic acid | | 0.39377 | -1.3446 |
| Phloretin | | 0.38346 | -1.3828 |
| Lithocholic acid | | 0.34833 | -1.5215 |
| 2-Phenylaminoadenosine | | 0.32686 | -1.6132 |
| 3-Chlorotyrosine | | 0.30291 | -1.723 |
| 3-Indolepropionic acid | | 0.29216 | -1.7752 |
| Taurocholic acid | | 0.28641 | -1.8038 |
| Indoleacrylic acid | | 0.27784 | -1.8477 |
| L-Cystathionine | | 0.25314 | -1.982 |
| Corticosterone | | 0.24895 | -2.0061 |
| Creatinine | | 0.2488 | -2.0069 |
| Quinolinic acid | | 0.24774 | -2.0131 |
| Beta-Alanine | | 0.24589 | -2.0239 |
| D-Alanine | | 0.24589 | -2.0239 |
| L-Alanine | | 0.24589 | -2.0239 |
| Sarcosine | | 0.24589 | -2.0239 |
| Homovanillic acid | | 0.24226 | -2.0454 |
| Hydroxyphenyllactic acid | | 0.24226 | -2.0454 |
| 9-Methyluric acid | | 0.24112 | -2.0522 |
| Galacturonic acid | | 0.23738 | -2.0747 |
| 3-Methylindole | | 0.23667 | -2.079 |
| Cortexolone | | 0.22339 | -2.1624 |
| Niacinamide | | 0.21716 | -2.2032 |
| Homo-L-arginine | | 0.21159 | -2.2407 |
| 2-Furoylglycine | | 0.20114 | -2.3138 |
| 2-Methyl-ketovaleric acid | | 0.18803 | -2.411 |
| Ketoleucine | | 0.18785 | -2.4123 |
| 3-Oxocholic acid | | 0.17027 | -2.5541 |
| 3-Cresotinic acid | | 0.15987 | -2.645 |
| 4-Hydroxy-methylbenzoic acid | | 0.15987 | -2.645 |
| Mandelic acid | | 0.15987 | -2.645 |
| p-Anisic acid | | 0.15987 | -2.645 |
| Glycyl-L-leucine | | 0.15814 | -2.6607 |
| N-Alpha-acetyllysine | | 0.15814 | -2.6607 |
| Glycocholic acid | | 0.15142 | -2.7233 |
| 2-Methoxyestradiol | | 0.15105 | -2.7269 |
| Taurodeoxycholic acid | | 0.15077 | -2.7296 |
| 4-Methoxycinnamic acid | | 0.15049 | -2.7323 |
| Uracil | | 0.14257 | -2.8103 |
| Alpha-Aspartyl-lysine | | 0.13213 | -2.9199 |
| Melatonin | | 0.13109 | -2.9314 |
| Cholic acid | | 0.12228 | -3.0317 |
| Fumaric acid | | 0.12154 | -3.0405 |
| N-Acetyl-L-phenylalanine | | 0.1179 | -3.0844 |
| Cortisone | | 0.11677 | -3.0983 |
| Indolelactic acid | | 0.115 | -3.1202 |
| Thymidine | | 0.11145 | -3.1655 |
| Methylcysteine | | 0.11056 | -3.1771 |
| D-Pantethine | | 0.10463 | -3.2566 |
| 5-Methoxytryptophan | | 0.10411 | -3.2638 |
| 3-Phenylbutyric acid | | 0.10001 | -3.3217 |
| 2-Phenylbutyric acid | | 0.10001 | -3.3218 |
| Guaifenesin | | 0.098917 | -3.3376 |
| D-Xylose | | 0.095655 | -3.386 |
| Benzenebutanoic acid | | 0.091645 | -3.4478 |
| 3-Methoxybenzenepropanoic acid | | 0.090763 | -3.4617 |
| Maltotriose | | 0.087302 | -3.5178 |
| L-Acetylcarnitine | | 0.087037 | -3.5222 |
| Raffinose | | 0.083722 | -3.5783 |
| Guanidinosuccinic acid | | 0.083266 | -3.5861 |
| D-Xylulose | | 0.072392 | -3.788 |
| Perillic acid | | 0.070187 | -3.8327 |
| Genistein | | 0.06913 | -3.8546 |
| Indoxyl | | 0.06826 | -3.8728 |
| 4-Hydroxycyclohexylcarboxylic acid | | 0.06551 | -3.9321 |
| 4-Aminohippuric acid | | 0.06514 | -3.9403 |
| N-Acetylserotonin | | 0.064578 | -3.9528 |
| FAPy-adenine | | 0.062373 | -4.0029 |
| Biotin | | 0.060343 | -4.0507 |
| D-Ribose 5-phosphate | | 0.060163 | -4.055 |
| Methionine sulfoxide | | 0.054121 | -4.2077 |
| 2-Phenylglycine | | 0.048989 | -4.3514 |
| Phthalic acid | | 0.047415 | -4.3985 |
| Quercetin | | 0.044502 | -4.49 |
| Shikimic acid | | 0.044299 | -4.4966 |
| 2-Isopropylmalic acid | | 0.04393 | -4.5086 |
| Indole-carbinol | | 0.039641 | -4.6569 |
| 3-Phenoxypropionic acid | | 0.037935 | -4.7203 |
| Desaminotyrosine | | 0.037935 | -4.7203 |
| L-Phenyllactic acid | | 0.037935 | -4.7203 |
| Biocytin | | 0.036812 | -4.7637 |
| Quinaldic acid | | 0.036023 | -4.795 |
| Menadione | | 0.035817 | -4.8032 |
| Bilirubin | | 0.033215 | -4.912 |
| 16-Dehydroprogesterone | | 0.030852 | -5.0185 |
| Quinic acid | | 0.030435 | -5.0381 |
| 3,4-Dihydroxybenzeneacetic acid | | 0.02794 | -5.1615 |
| Homogentisic acid | | 0.02794 | -5.1615 |
| Vanillic acid | | 0.02794 | -5.1615 |
| Citrulline | | 0.026557 | -5.2347 |
| Indoleacetic acid | | 0.021698 | -5.5263 |
| N-Formyl-L-methionine | | 0.019184 | -5.704 |
| Glycylproline | | 0.014645 | -6.0934 |
| 6-Dimethylaminopurine | | 0.010917 | -6.5172 |
| 3-Methyladenine | | 0.005744 | -7.4438 |
| 6-Methyladenine | | 0.005744 | -7.4438 |
| Phosphorylcholine | | 0.003822 | -8.0313 |
| Dopamine | | 0.001097 | -9.8324 |
| **VIP>1** | | | |
| **Metabolites** | | **Comp. 1** | **Comp. 2** |
| D-Glutamine | | 1.1765 | 1.1755 |
| L-Glutamine | | 1.1765 | 1.1755 |
| L-Phenylalanine | | 1.1764 | 1.1754 |
| 3,4-Dihydroxybenzeneacetic acid | | 1.1763 | 1.1753 |
| Homogentisic acid | | 1.1763 | 1.1753 |
| Vanillic acid | | 1.1763 | 1.1753 |
| L-Tryptophan | | 1.1761 | 1.1752 |
| 7a-Hydroxy-oxo-5b-cholanoic acid | | 1.1758 | 1.1748 |
| Chenodeoxycholic acid | | 1.1757 | 1.1748 |
| Deoxycholic acid | | 1.1757 | 1.1748 |
| Fructose 6-phosphate | | 1.1757 | 1.1747 |
| Glucose 1-phosphate | | 1.1757 | 1.1747 |
| Glucose 6-phosphate | | 1.1757 | 1.1747 |
| Hyodeoxycholic acid | | 1.1757 | 1.1748 |
| Mannose 6-phosphate | | 1.1757 | 1.1747 |
| Ursodeoxycholic acid | | 1.1757 | 1.1748 |
| 2,2-Dimethylsuccinic acid | | 1.1751 | 1.1741 |
| 2-Methylglutaric acid | | 1.1751 | 1.1741 |
| Methylglutaric acid | | 1.1751 | 1.1741 |
| Monomethyl glutaric acid | | 1.1751 | 1.1741 |
| Gluconolactone | | 1.175 | 1.174 |
| Allose | | 1.1748 | 1.1738 |
| Alpha-D-Glucose | | 1.1748 | 1.1738 |
| D-Fructose | | 1.1748 | 1.1738 |
| D-Galactose | | 1.1748 | 1.1738 |
| D-Glucose | | 1.1748 | 1.1738 |
| D-Mannose | | 1.1748 | 1.1738 |
| D-Tagatose | | 1.1748 | 1.1738 |
| L-Sorbose | | 1.1748 | 1.1738 |
| Myoinositol | | 1.1748 | 1.1738 |
| Scyllitol | | 1.1748 | 1.1738 |
| Glycerol 3-phosphate | | 1.1744 | 1.1734 |
| Theobromine | | 1.1743 | 1.1733 |
| Indoxyl | | 1.1742 | 1.1732 |
| Indole-carbinol | | 1.1741 | 1.1731 |
| Flavin Mononucleotide | | 1.174 | 1.173 |
| 3-Phenoxypropionic acid | | 1.1738 | 1.1729 |
| Desaminotyrosine | | 1.1738 | 1.1729 |
| L-Phenyllactic acid | | 1.1738 | 1.1729 |
| Canrenone | | 1.1735 | 1.1725 |
| L-Glutamic acid | | 1.1735 | 1.1725 |
| L-Tyrosine | | 1.1729 | 1.1719 |
| o-Tyrosine | | 1.1729 | 1.1719 |
| dCMP | | 1.1726 | 1.1716 |
| 4-Hydroxyphenylpyruvic acid | | 1.1724 | 1.1714 |
| Azelaic acid | | 1.1724 | 1.1714 |
| Caffeic acid | | 1.1724 | 1.1714 |
| Nicotinuric acid | | 1.1723 | 1.1713 |
| Arachidic acid | | 1.1719 | 1.1709 |
| Niacinamide | | 1.1719 | 1.1709 |
| 5-Methoxytryptophan | | 1.1718 | 1.1709 |
| Hippuric acid | | 1.1715 | 1.1706 |
| Fumaric acid | | 1.1713 | 1.1703 |
| Elaidic acid | | 1.1709 | 1.17 |
| Oleic acid | | 1.1709 | 1.17 |
| Vaccenic acid | | 1.1709 | 1.17 |
| Nutriacholic acid | | 1.1708 | 1.1698 |
| 3-Methylindole | | 1.1706 | 1.1696 |
| 3-Methoxybenzenepropanoic acid | | 1.1705 | 1.1695 |
| Rhamnose | | 1.1701 | 1.1691 |
| Sebacic acid | | 1.17 | 1.169 |
| Phenylpropiolic acid | | 1.1699 | 1.1689 |
| Linoleic acid | | 1.1698 | 1.1689 |
| 5-Phenylvaleric acid | | 1.1695 | 1.1686 |
| Phosphocreatine | | 1.1692 | 1.1682 |
| Guanidinosuccinic acid | | 1.1689 | 1.168 |
| FAD | | 1.1688 | 1.1678 |
| Guanosine | | 1.1688 | 1.1679 |
| 7-Methylxanthine | | 1.1686 | 1.1677 |
| Indoleacetic acid | | 1.1686 | 1.1677 |
| 3-Methoxytyrosine | | 1.1678 | 1.1669 |
| ADP | | 1.1677 | 1.1667 |
| Dodecanoic acid | | 1.1676 | 1.1666 |
| 1,3-Dimethyluric acid | | 1.1675 | 1.1665 |
| 1,9-Dimethyluric acid | | 1.1675 | 1.1665 |
| L-Cystathionine | | 1.167 | 1.166 |
| 6-Phosphogluconic acid | | 1.1669 | 1.1659 |
| Menadione | | 1.1668 | 1.1658 |
| Atenolol | | 1.1664 | 1.1655 |
| Citric acid | | 1.1661 | 1.1652 |
| D-threo-Isocitric acid | | 1.1661 | 1.1652 |
| Galactonic acid | | 1.1661 | 1.1651 |
| Gluconic acid | | 1.1661 | 1.1651 |
| Isocitric acid | | 1.1661 | 1.1652 |
| Alpha-Linolenic acid | | 1.166 | 1.165 |
| Quinic acid | | 1.1658 | 1.1648 |
| Deoxyadenosine monophosphate | | 1.1651 | 1.1642 |
| 1-Methylguanine | | 1.1649 | 1.1639 |
| 7-Methylguanine | | 1.1649 | 1.1639 |
| Beta-Alanine | | 1.1649 | 1.1639 |
| D-Alanine | | 1.1649 | 1.1639 |
| L-Alanine | | 1.1649 | 1.1639 |
| Sarcosine | | 1.1649 | 1.1639 |
| 11a-Hydroxyprogesterone | | 1.1647 | 1.1638 |
| 17-Hydroxyprogesterone | | 1.1647 | 1.1638 |
| Deoxycorticosterone | | 1.1647 | 1.1638 |
| Inosine | | 1.1644 | 1.1634 |
| Adenosine 2',3'-cyclic phosphate | | 1.1642 | 1.1632 |
| Cyclic AMP | | 1.1642 | 1.1632 |
| Biotin | | 1.1639 | 1.1629 |
| Guanosine diphosphate | | 1.1639 | 1.1629 |
| 2-Isopropylmalic acid | | 1.1636 | 1.1626 |
| L-Proline | | 1.1633 | 1.1624 |
| Pantothenic acid | | 1.163 | 1.162 |
| N-Acetylneuraminic acid | | 1.1629 | 1.1619 |
| Alpha-Aspartyl-lysine | | 1.1624 | 1.1615 |
| Myristic acid | | 1.1624 | 1.1614 |
| Stearic acid | | 1.1624 | 1.1614 |
| Phenylacetylglycine | | 1.1622 | 1.1613 |
| Aminocaproic acid | | 1.1619 | 1.161 |
| Beta-Leucine | | 1.1619 | 1.161 |
| L-Alloisoleucine | | 1.1619 | 1.161 |
| L-Isoleucine | | 1.1619 | 1.161 |
| L-Leucine | | 1.1619 | 1.161 |
| L-Norleucine | | 1.1619 | 1.161 |
| Uridine 5'-monophosphate | | 1.1619 | 1.1609 |
| Inosinic acid | | 1.1616 | 1.1606 |
| N-Acetylserotonin | | 1.1615 | 1.1606 |
| 3-Methyladenine | | 1.1599 | 1.159 |
| 6-Methyladenine | | 1.1599 | 1.159 |
| Pentadecanoic acid | | 1.1594 | 1.1584 |
| D-Arginine | | 1.1587 | 1.1577 |
| L-Arginine | | 1.1587 | 1.1577 |
| Heptadecanoic acid | | 1.1585 | 1.1575 |
| Phenylglyoxylic acid | | 1.1579 | 1.1571 |
| Melatonin | | 1.1537 | 1.1528 |
| NADH | | 1.1536 | 1.1527 |
| Uracil | | 1.1533 | 1.1524 |
| 13-cis-Retinoic acid | | 1.1531 | 1.1522 |
| All-trans-retinoic acid | | 1.1531 | 1.1522 |
| Alpha-Hydroxyhippuric acid | | 1.1524 | 1.1514 |
| Glycyl-glycine | | 1.1511 | 1.1502 |
| L-Asparagine | | 1.1511 | 1.1502 |
| Homovanillic acid | | 1.1501 | 1.1492 |
| Hydroxyphenyllactic acid | | 1.1501 | 1.1492 |
| Uridine diphosphate glucose | | 1.1495 | 1.1486 |
| Oxoglutaric acid | | 1.1491 | 1.1482 |
| Adenosine triphosphate | | 1.1487 | 1.1478 |
| dGTP | | 1.1487 | 1.1478 |
| Cholesterol sulfate | | 1.1479 | 1.1471 |
| Ribonolactone | | 1.1477 | 1.1467 |
| Galacturonic acid | | 1.1473 | 1.1464 |
| Lipoamide | | 1.1473 | 1.1463 |
| L-Aspartyl-L-phenylalanine | | 1.1454 | 1.1445 |
| 1,11-Undecanedicarboxylic acid | | 1.1453 | 1.1444 |
| N-Acetyl-D-glucosamine | | 1.1444 | 1.1436 |
| N-Acetylgalactosamine | | 1.1444 | 1.1436 |
| Cytidine triphosphate | | 1.1443 | 1.1434 |
| 5-Aminopentanoic acid | | 1.1419 | 1.1409 |
| Betaine | | 1.1419 | 1.1409 |
| L-Valine | | 1.1419 | 1.1409 |
| N-Methyl-a-aminoisobutyric acid | | 1.1419 | 1.1409 |
| Xanthosine | | 1.1412 | 1.1405 |
| L-Acetylcarnitine | | 1.1407 | 1.1397 |
| 2-Methoxyestradiol | | 1.14 | 1.1392 |
| Uridine 5'-diphosphate | | 1.1397 | 1.1387 |
| Malic acid | | 1.1383 | 1.1375 |
| 4-Aminohippuric acid | | 1.1343 | 1.1334 |
| D-Pantethine | | 1.1335 | 1.1326 |
| D-Lysine | | 1.1334 | 1.1325 |
| L-Lysine | | 1.1334 | 1.1325 |
| L-Malic acid | | 1.1333 | 1.1325 |
| Coproporphyrin I | | 1.1324 | 1.1314 |
| Methylcysteine | | 1.1293 | 1.1283 |
| Dodecanedioic acid | | 1.1291 | 1.1282 |
| Ethenodeoxyadenosine | | 1.1278 | 1.1269 |
| Lithocholic acid | | 1.1276 | 1.1267 |
| D-Ribose 5-phosphate | | 1.1241 | 1.1233 |
| Inosine triphosphate | | 1.1186 | 1.1177 |
| Cortexolone | | 1.1182 | 1.1173 |
| 3-Aminoisobutanoic acid | | 1.1164 | 1.1154 |
| D-Alpha-aminobutyric acid | | 1.1164 | 1.1154 |
| Dimethylglycine | | 1.1164 | 1.1154 |
| Gamma-Aminobutyric acid | | 1.1164 | 1.1154 |
| L-Alpha-aminobutyric acid | | 1.1164 | 1.1154 |
| 2-Furoylglycine | | 1.1158 | 1.1148 |
| Phloretin | | 1.115 | 1.1141 |
| Perillic acid | | 1.1094 | 1.1086 |
| Pyroglutamic acid | | 1.1088 | 1.1079 |
| Pyrrolidonecarboxylic acid | | 1.1088 | 1.1079 |
| 3-Phosphoglyceric acid | | 1.1055 | 1.1047 |
| Allantoic acid | | 1.1046 | 1.1037 |
| 3-Oxocholic acid | | 1.1045 | 1.1036 |
| Glycerophosphocholine | | 1.104 | 1.1031 |
| 9-Methyluric acid | | 1.1002 | 1.0993 |
| Benzenebutanoic acid | | 1.098 | 1.0971 |
| Aspartame | | 1.0959 | 1.095 |
| Glucaric acid | | 1.0946 | 1.0941 |
| N-Acetylmannosamine | | 1.0934 | 1.0937 |
| Levulinic acid | | 1.0925 | 1.0922 |
| L-Methionine | | 1.0904 | 1.0896 |
| Raffinose | | 1.0887 | 1.0885 |
| 5-Thymidylic acid | | 1.0875 | 1.0866 |
| Taurine | | 1.0845 | 1.0837 |
| 4-Hydroxyproline | | 1.0809 | 1.0801 |
| 5-Aminolevulinic acid | | 1.0809 | 1.0801 |
| N-Acetyl-L-alanine | | 1.0809 | 1.0801 |
| Methionine sulfoxide | | 1.0807 | 1.0806 |
| D-Sorbitol | | 1.0781 | 1.0772 |
| Galactitol | | 1.0781 | 1.0772 |
| Mannitol | | 1.0781 | 1.0772 |
| Traumatic acid | | 1.0737 | 1.0729 |
| 5'-Methylthioadenosine | | 1.0734 | 1.0725 |
| 4-Hydroxycyclohexylcarboxylic acid | | 1.0712 | 1.0711 |
| 3-Indolepropionic acid | | 1.0694 | 1.0688 |
| Glycyl-L-leucine | | 1.0693 | 1.0685 |
| N-Alpha-acetyllysine | | 1.0693 | 1.0685 |
| Hematoporphyrin IX | | 1.0684 | 1.0675 |
| 2-Phenylglycine | | 1.0673 | 1.0666 |
| Indolelactic acid | | 1.0661 | 1.0655 |
| Phthalic acid | | 1.0625 | 1.0624 |
| Pipecolic acid | | 1.0583 | 1.0591 |
| N-Acetyl-L-phenylalanine | | 1.0507 | 1.0502 |
| Dopamine | | 1.0494 | 1.0495 |
| 16-Dehydroprogesterone | | 1.0471 | 1.0462 |
| Indoleacrylic acid | | 1.0451 | 1.045 |
| Quinaldic acid | | 1.043 | 1.0431 |
| 6-Dimethylaminopurine | | 1.0426 | 1.0427 |
| L-Histidine | | 1.0419 | 1.0411 |
| Shikimic acid | | 1.0403 | 1.0405 |
| Bilirubin | | 1.0391 | 1.039 |
| FAPy-adenine | | 1.0365 | 1.0367 |
| 4-Methoxycinnamic acid | | 1.0338 | 1.0343 |
| L-Homoserine | | 1.0334 | 1.0326 |
| L-Threonine | | 1.0334 | 1.0326 |
| lipoic acid | | 1.0315 | 1.0306 |
| Guanosine triphosphate | | 1.0307 | 1.0298 |
| 3-Chlorotyrosine | | 1.0306 | 1.03 |
| Thymidine | | 1.0287 | 1.0291 |
| Cortisone | | 1.027 | 1.0263 |
| Taurodeoxycholic acid | | 1.0223 | 1.0223 |
| m-Chlorobenzoic acid | | 1.0222 | 1.0213 |
| 3b-Hydroxy-5-cholenoic acid | | 1.0158 | 1.0157 |
| Maltotriose | | 1.0149 | 1.0156 |
| 2-Chlorobenzoic acid | | 1.0146 | 1.0138 |
| Glycocholic acid | | 1.013 | 1.0136 |
| 2-Hydroxy-methylbutyric acid | | 1.0092 | 1.0083 |
| Ibuprofen | | 1.009 | 1.0087 |
| Tryptamine | | 1.0055 | 1.0047 |
| Creatinine | | 1.0028 | 1.0035 |
| 2,5-Furandicarboxylic acid | | 1.0022 | 1.0021 |
| **Final selective markers from negtive mode (224 metabolites)** | | | |
| 1,11-Undecanedicarboxylic acid | Aspartame | Glycerol 3-phosphate | Melatonin |
| 1,3-Dimethyluric acid | Atenolol | Glycerophosphocholine | Menadione |
| 1,9-Dimethyluric acid | Azelaic acid | Glycocholic acid | Methionine sulfoxide |
| 11a-Hydroxyprogesterone | Benzenebutanoic acid | Glycyl-glycine | Methylcysteine |
| 13-cis-Retinoic acid | Beta-Alanine | Glycyl-L-leucine | Methylglutaric acid |
| 16-Dehydroprogesterone | Betaine | Guanidinosuccinic acid | Monomethyl glutaric acid |
| 17-Hydroxyprogesterone | Beta-Leucine | Guanosine | Myoinositol |
| 1-Methylguanine | Bilirubin | Guanosine diphosphate | Myristic acid |
| 2,2-Dimethylsuccinic acid | Biotin | Guanosine triphosphate | N-Acetyl-L-alanine |
| 2,5-Furandicarboxylic acid | Caffeic acid | Hematoporphyrin IX | N-Acetyl-L-phenylalanine |
| 2-Chlorobenzoic acid | Canrenone | Heptadecanoic acid | N-Acetylneuraminic acid |
| 2-Furoylglycine | Chenodeoxycholic acid | Hippuric acid | N-Acetylserotonin |
| 2-Isopropylmalic acid | Cholesterol sulfate | Homogentisic acid | NADH |
| 2-Methoxyestradiol | Citric acid | Homovanillic acid | N-Alpha-acetyllysine |
| 2-Methylglutaric acid | Coproporphyrin I | Hydroxyphenyllactic acid | Niacinamide |
| 2-Phenylglycine | Cortexolone | Hyodeoxycholic acid | Nicotinuric acid |
| 3,4-Dihydroxybenzeneacetic acid | Cortisone | Indoleacetic acid | N-Methyl-a-aminoisobutyric acid |
| 3-Chlorotyrosine | Creatinine | Indoleacrylic acid | Nutriacholic acid |
| 3-Indolepropionic acid | Cyclic AMP | Indole-carbinol | Oleic acid |
| 3-Methoxybenzenepropanoic acid | Cytidine triphosphate | Indolelactic acid | o-Tyrosine |
| 3-Methoxytyrosine | D-Alanine | Indoxyl | Oxoglutaric acid |
| 3-Methyladenine | D-Arginine | Inosine | Pantothenic acid |
| 3-Methylindole | dCMP | Inosine triphosphate | Pentadecanoic acid |
| 3-Oxocholic acid | Deoxyadenosine monophosphate | Inosinic acid | Perillic acid |
| 3-Phenoxypropionic acid | Deoxycholic acid | Isocitric acid | Phenylacetylglycine |
| 3-Phosphoglyceric acid | Deoxycorticosterone | L-Acetylcarnitine | Phenylglyoxylic acid |
| 4-Aminohippuric acid | Desaminotyrosine | L-Alanine | Phenylpropiolic acid |
| 4-Hydroxycyclohexylcarboxylic acid | D-Fructose | L-Alloisoleucine | Phloretin |
| 4-Hydroxyphenylpyruvic acid | D-Galactose | L-Arginine | Phosphocreatine |
| 4-Hydroxyproline | D-Glutamine | L-Asparagine | Phthalic acid |
| 4-Methoxycinnamic acid | dGTP | L-Aspartyl-L-phenylalanine | Pipecolic acid |
| 5-Aminolevulinic acid | D-Lysine | L-Cystathionine | Pyroglutamic acid |
| 5-Aminopentanoic acid | D-Mannose | Levulinic acid | Pyrrolidonecarboxylic acid |
| 5-Methoxytryptophan | Dodecanedioic acid | L-Glutamic acid | Quinaldic acid |
| 5'-Methylthioadenosine | Dodecanoic acid | L-Glutamine | Quinic acid |
| 5-Phenylvaleric acid | Dopamine | Linoleic acid | Raffinose |
| 5-Thymidylic acid | D-Pantethine | Lipoamide | Rhamnose |
| 6-Dimethylaminopurine | D-Ribose 5-phosphate | L-Isoleucine | Ribonolactone |
| 6-Methyladenine | D-Sorbitol | Lithocholic acid | Sarcosine |
| 6-Phosphogluconic acid | D-Tagatose | L-Leucine | Scyllitol |
| 7a-Hydroxy-oxo-5b-cholanoic acid | D-threo-Isocitric acid | L-Lysine | Sebacic acid |
| 7-Methylguanine | Elaidic acid | L-Malic acid | Shikimic acid |
| 7-Methylxanthine | Ethenodeoxyadenosine | L-Methionine | Stearic acid |
| 9-Methyluric acid | FAD | L-Norleucine | Taurodeoxycholic acid |
| Adenosine 2',3'-cyclic phosphate | FAPy-adenine | L-Phenylalanine | Theobromine |
| Adenosine triphosphate | Flavin Mononucleotide | L-Phenyllactic acid | Thymidine |
| ADP | Fructose 6-phosphate | L-Proline | Traumatic acid |
| Allantoic acid | Fumaric acid | L-Sorbose | Tryptamine |
| Allose | Galactitol | L-Tryptophan | Uracil |
| All-trans-retinoic acid | Galactonic acid | L-Tyrosine | Uridine 5'-diphosphate |
| Alpha-Aspartyl-lysine | Galacturonic acid | L-Valine | Uridine 5'-monophosphate |
| Alpha-D-Glucose | Glucaric acid | Malic acid | Uridine diphosphate glucose |
| Alpha-Hydroxyhippuric acid | Gluconic acid | Maltotriose | Ursodeoxycholic acid |
| Alpha-Linolenic acid | Gluconolactone | Mannitol | Vaccenic acid |
| Aminocaproic acid | Glucose 1-phosphate | Mannose 6-phosphate | Vanillic acid |
| Arachidic acid | Glucose 6-phosphate | m-Chlorobenzoic acid | Xanthosine |

**Table S15 Biomarkers selection between control group and model group from positive ion mode (p<0.05, (|Log2-fold change | > 1, VIP>1).**

| **p<0.05** | | | |
| --- | --- | --- | --- |
| **Metabolites** | | **t.stat** | **p.value** |
| 3-Pyridylacetic acid | | 42.075 | 2.79E-15 |
| D-Arginine | | 41.329 | 3.51E-15 |
| L-Arginine | | 41.329 | 3.51E-15 |
| D-Lysine | | 33.197 | 5.92E-14 |
| L-Lysine | | 33.197 | 5.92E-14 |
| L-Tyrosine | | -33.168 | 5.98E-14 |
| o-Tyrosine | | -33.168 | 5.98E-14 |
| D-Sorbitol | | -30.906 | 1.48E-13 |
| Galactitol | | -30.906 | 1.48E-13 |
| Mannitol | | -30.906 | 1.48E-13 |
| Pyridoxamine | | 30.54 | 1.73E-13 |
| Guanosine | | -27.489 | 6.66E-13 |
| Beta-N-Acetylglucosamine | | -25.769 | 1.52E-12 |
| N-Acetyl-D-glucosamine | | -25.769 | 1.52E-12 |
| N-Acetylgalactosamine | | -25.769 | 1.52E-12 |
| N-Acetylmannosamine | | -25.769 | 1.52E-12 |
| Indoleacrylic acid | | -25.194 | 2.03E-12 |
| ADP | | 24.235 | 3.33E-12 |
| 3-Methylindole | | 24.225 | 3.34E-12 |
| Pipecolic acid | | 23.403 | 5.19E-12 |
| PA(16:0/16:0) | | 23.258 | 5.61E-12 |
| Deoxyguanosine | | -21.982 | 1.15E-11 |
| Undecanedioic acid | | 21.201 | 1.82E-11 |
| 3-Cresotinic acid | | 20.57 | 2.66E-11 |
| 4-Hydroxy-3-methylbenzoic acid | | 20.57 | 2.66E-11 |
| Mandelic acid | | 20.57 | 2.66E-11 |
| p-Anisic acid | | 20.57 | 2.66E-11 |
| Creatine | | 20.268 | 3.21E-11 |
| 2'-Deoxyguanosine 5'-monophosphate | | 19.842 | 4.20E-11 |
| Adenosine monophosphate | | 19.842 | 4.20E-11 |
| Retinal | | 19.191 | 6.39E-11 |
| L-Carnitine | | 18.686 | 8.94E-11 |
| D-Glutamine | | 18.588 | 9.55E-11 |
| L-Glutamine | | 18.588 | 9.55E-11 |
| 2-Phenylglycine | | 18.578 | 9.61E-11 |
| Glutathione | | 18.005 | 1.42E-10 |
| 7-Ketocholesterol | | -17.967 | 1.46E-10 |
| 4-Hydroxyproline | | 17.545 | 1.97E-10 |
| 5-Aminolevulinic acid | | 17.545 | 1.97E-10 |
| N-Acetyl-L-alanine | | 17.545 | 1.97E-10 |
| Sebacic acid | | 17.374 | 2.23E-10 |
| Phosphocreatine | | -16.87 | 3.21E-10 |
| Perillic acid | | -16.637 | 3.82E-10 |
| 3-Methoxytyramine | | -16.502 | 4.23E-10 |
| Phenylephrine | | -16.502 | 4.23E-10 |
| 3,4-Dihydroxybenzeneacetic acid | | 16.432 | 4.46E-10 |
| Homogentisic acid | | 16.432 | 4.46E-10 |
| Vanillic acid | | 16.432 | 4.46E-10 |
| L-Methionine | | -16.275 | 5.03E-10 |
| Arachidic acid | | 16.054 | 5.95E-10 |
| 7a-Hydroxy-3-oxo-5b-cholanoic acid | | 15.753 | 7.54E-10 |
| Nutriacholic acid | | 15.753 | 7.54E-10 |
| Phenylpropiolic acid | | -15.747 | 7.57E-10 |
| L-Histidine | | 15.615 | 8.40E-10 |
| Guanosine monophosphate | | 15.454 | 9.55E-10 |
| lipoic acid | | 14.988 | 1.39E-09 |
| Ureidosuccinic acid | | 14.978 | 1.41E-09 |
| Suberic acid | | 14.361 | 2.36E-09 |
| N-Formyl-L-methionine | | 14.157 | 2.81E-09 |
| 5-Aminopentanoic acid | | -14.007 | 3.20E-09 |
| Betaine | | -14.007 | 3.20E-09 |
| L-Valine | | -14.007 | 3.20E-09 |
| N-Methyl-a-aminoisobutyric acid | | -14.007 | 3.20E-09 |
| 2,2-Dimethylsuccinic acid | | 13.956 | 3.35E-09 |
| 2-Methylglutaric acid | | 13.956 | 3.35E-09 |
| Methylglutaric acid | | 13.956 | 3.35E-09 |
| Monomethyl glutaric acid | | 13.956 | 3.35E-09 |
| 1-Methyladenosine | | -13.395 | 5.53E-09 |
| Beta-Alanine | | 13.219 | 6.50E-09 |
| D-Alanine | | 13.219 | 6.50E-09 |
| L-Alanine | | 13.219 | 6.50E-09 |
| Sarcosine | | 13.219 | 6.50E-09 |
| N-Acetyl-L-phenylalanine | | -12.644 | 1.11E-08 |
| Inosinic acid | | 12.238 | 1.65E-08 |
| Inosine | | 12.15 | 1.80E-08 |
| Inosine triphosphate | | 12.086 | 1.92E-08 |
| 5-Thymidylic acid | | 12.06 | 1.97E-08 |
| L-Phenylalanine | | -11.613 | 3.09E-08 |
| Sphinganine | | -11.498 | 3.48E-08 |
| Methionine sulfoxide | | -11.368 | 3.99E-08 |
| DL-2-Aminooctanoic acid | | -11.232 | 4.60E-08 |
| Shikimic acid | | 10.947 | 6.23E-08 |
| Hexadecanedioic acid | | 10.939 | 6.29E-08 |
| Triamterene | | -10.906 | 6.52E-08 |
| 2-Ethyl-2-Hydroxybutyric acid | | 10.641 | 8.71E-08 |
| 2-Hydroxycaproic acid | | 10.641 | 8.71E-08 |
| Leucinic acid | | 10.641 | 8.71E-08 |
| Coproporphyrin I | | 10.434 | 1.10E-07 |
| 3-Phosphoglyceric acid | | 10.272 | 1.32E-07 |
| Pregnenolone sulfate | | 9.7964 | 2.28E-07 |
| Pyroglutamic acid | | 9.5949 | 2.90E-07 |
| Pyrrolidonecarboxylic acid | | 9.5949 | 2.90E-07 |
| Homovanillic acid | | -9.4134 | 3.61E-07 |
| Hydroxyphenyllactic acid | | -9.4134 | 3.61E-07 |
| Malonic acid | | 9.3514 | 3.89E-07 |
| Glycyl-glycine | | 9.1504 | 4.99E-07 |
| L-Asparagine | | 9.1504 | 4.99E-07 |
| Phenylglyoxylic acid | | 8.5979 | 1.01E-06 |
| Phthalic acid | | 8.3833 | 1.33E-06 |
| NADH | | 8.1783 | 1.76E-06 |
| Tryptamine | | 7.9659 | 2.34E-06 |
| Urocanic acid | | 7.9482 | 2.40E-06 |
| Adenosine | | 7.7218 | 3.29E-06 |
| Niacinamide | | -7.7039 | 3.37E-06 |
| 1,11-Undecanedicarboxylic acid | | -7.4933 | 4.54E-06 |
| Indoxyl | | 7.3188 | 5.84E-06 |
| Biocytin | | 7.2531 | 6.43E-06 |
| 3-Methoxytyrosine | | -7.2183 | 6.76E-06 |
| Allantoic acid | | 7.185 | 7.10E-06 |
| L-Glutamic acid | | -7.1139 | 7.89E-06 |
| cis,cis-Muconic acid | | 6.941 | 1.02E-05 |
| Xanthosine | | -6.8881 | 1.10E-05 |
| Adrenaline | | 6.8046 | 1.25E-05 |
| Normetanephrine | | 6.8046 | 1.25E-05 |
| L-Proline | | 6.7676 | 1.33E-05 |
| 3-Hydroxybenzoic acid | | 6.7095 | 1.45E-05 |
| 4-Hydroxybenzoic acid | | 6.7095 | 1.45E-05 |
| Flavin Mononucleotide | | -6.7087 | 1.45E-05 |
| Traumatic acid | | -6.6554 | 1.57E-05 |
| L-Acetylcarnitine | | 6.6305 | 1.64E-05 |
| L-Aspartyl-L-phenylalanine | | 6.5733 | 1.79E-05 |
| Ribonolactone | | 6.5552 | 1.84E-05 |
| Adenine | | -6.5474 | 1.86E-05 |
| 1-Methylguanine | | -6.4334 | 2.22E-05 |
| 7-Methylguanine | | -6.4334 | 2.22E-05 |
| 11a-Hydroxyprogesterone | | 6.4256 | 2.25E-05 |
| 17-Hydroxyprogesterone | | 6.4256 | 2.25E-05 |
| Deoxycorticosterone | | 6.4256 | 2.25E-05 |
| Isoxanthopterin | | 6.3841 | 2.40E-05 |
| Citrulline | | 6.29 | 2.79E-05 |
| Adenosine triphosphate | | 6.2452 | 2.99E-05 |
| dGTP | | 6.2452 | 2.99E-05 |
| Uridine 5'-monophosphate | | 5.9168 | 5.09E-05 |
| Methylcysteine | | 5.8968 | 5.26E-05 |
| Glucosamine 6-phosphate | | 5.7914 | 6.26E-05 |
| Glucaric acid | | 5.7601 | 6.60E-05 |
| Aminoadipic acid | | -5.7507 | 6.70E-05 |
| Creatinine | | -5.674 | 7.62E-05 |
| Azelaic acid | | 5.245 | 0.000158 |
| Uridine | | -5.2423 | 0.000159 |
| Alpha-Lactose | | 5.1012 | 0.000203 |
| Cellobiose | | 5.1012 | 0.000203 |
| Isomaltose | | 5.1012 | 0.000203 |
| Lactulose | | 5.1012 | 0.000203 |
| Melibiose | | 5.1012 | 0.000203 |
| Sucrose | | 5.1012 | 0.000203 |
| Trehalose | | 5.1012 | 0.000203 |
| Turanose | | 5.1012 | 0.000203 |
| Naproxen | | -5.0199 | 0.000235 |
| Guanosine diphosphate | | 5.0006 | 0.000243 |
| L-Tryptophan | | -4.9401 | 0.00027 |
| Homo-L-arginine | | 4.8869 | 0.000297 |
| Glycerophosphocholine | | -4.6856 | 0.000426 |
| L-Serine | | 4.6383 | 0.000464 |
| PC(16:0/16:0) | | 4.6343 | 0.000468 |
| N-Acetylglutamic acid | | -4.597 | 0.0005 |
| Allose | | -4.521 | 0.000575 |
| Alpha-D-Glucose | | -4.521 | 0.000575 |
| D-Fructose | | -4.521 | 0.000575 |
| D-Galactose | | -4.521 | 0.000575 |
| D-Glucose | | -4.521 | 0.000575 |
| D-Mannose | | -4.521 | 0.000575 |
| D-Tagatose | | -4.521 | 0.000575 |
| L-Sorbose | | -4.521 | 0.000575 |
| Myoinositol | | -4.521 | 0.000575 |
| Scyllitol | | -4.521 | 0.000575 |
| 5-Methoxytryptophan | | -4.5187 | 0.000577 |
| Nicotinuric acid | | -4.5112 | 0.000585 |
| Citric acid | | -4.4386 | 0.000669 |
| D-threo-Isocitric acid | | -4.4386 | 0.000669 |
| Isocitric acid | | -4.4386 | 0.000669 |
| Diaminopimelic acid | | 4.4375 | 0.00067 |
| 1,3,7-Trimethyluric acid | | -4.4034 | 0.000713 |
| Medroxyprogesterone | | -4.3541 | 0.000781 |
| L-Malic acid | | 4.2469 | 0.000953 |
| Malic acid | | 4.2469 | 0.000953 |
| L-Cystathionine | | 4.1825 | 0.001074 |
| L-Homoserine | | -4.1452 | 0.001152 |
| L-Threonine | | -4.1452 | 0.001152 |
| Chenodeoxycholic acid | | 4.1223 | 0.001202 |
| Deoxycholic acid | | 4.1223 | 0.001202 |
| Hyodeoxycholic acid | | 4.1223 | 0.001202 |
| Ursodeoxycholic acid | | 4.1223 | 0.001202 |
| Tetradecanedioic acid | | 3.9911 | 0.001538 |
| Purine | | -3.9543 | 0.001648 |
| Deoxycytidine | | -3.9301 | 0.001725 |
| Argininosuccinic acid | | 3.9141 | 0.001778 |
| D-Xylose | | -3.9026 | 0.001817 |
| D-Xylulose | | -3.9026 | 0.001817 |
| 1-Methylhistidine | | 3.768 | 0.002346 |
| 3-Methylhistidine | | 3.768 | 0.002346 |
| Linoleic acid | | 3.7115 | 0.002612 |
| 4-Ethylbenzoic acid | | 3.6823 | 0.002762 |
| Isoferulic acid | | -3.6251 | 0.00308 |
| trans-Ferulic acid | | -3.6251 | 0.00308 |
| Cortisone | | -3.555 | 0.003522 |
| L-Fucose | | -3.4523 | 0.004289 |
| Rhamnose | | -3.4523 | 0.004289 |
| Ibuprofen | | 3.4253 | 0.004517 |
| 4-Aminohippuric acid | | -3.3776 | 0.004951 |
| Biotin | | -3.3623 | 0.005099 |
| Androstanedione | | 3.3284 | 0.005443 |
| Dehydroepiandrosterone | | 3.3284 | 0.005443 |
| Testosterone | | 3.3284 | 0.005443 |
| Glycylproline | | -3.2967 | 0.005785 |
| Acetylcysteine | | 3.23 | 0.006577 |
| Melatonin | | -3.2028 | 0.00693 |
| Indolelactic acid | | 3.1991 | 0.00698 |
| 5-Hydroxymethyluracil | | 3.1408 | 0.007808 |
| Oxoglutaric acid | | -3.1403 | 0.007816 |
| 3-Indolepropionic acid | | 3.0145 | 0.009958 |
| 5-Methoxytryptamine | | 2.9934 | 0.01037 |
| Taurocholic acid | | -2.9243 | 0.011841 |
| Taurine | | 2.9212 | 0.011913 |
| Guaifenesin | | -2.9047 | 0.012297 |
| Vanillylmandelic acid | | 2.8955 | 0.012514 |
| Indole-3-carbinol | | 2.8739 | 0.013046 |
| 3,5-Diiodothyronine | | -2.8201 | 0.014462 |
| Glycyl-L-leucine | | 2.8189 | 0.014496 |
| N-Alpha-acetyllysine | | 2.8189 | 0.014496 |
| Lipoamide | | 2.676 | 0.019045 |
| Guanidinosuccinic acid | | -2.6338 | 0.020639 |
| Heptadecanoic acid | | 2.5875 | 0.022535 |
| 6-Dimethylaminopurine | | 2.5823 | 0.022758 |
| 3-Hexenedioic acid | | 2.5807 | 0.022825 |
| Dodecanoic acid | | 2.5537 | 0.024022 |
| 5-Phenylvaleric acid | | 2.5441 | 0.024463 |
| Guanosine triphosphate | | 2.5341 | 0.024931 |
| Cytidine monophosphate | | 2.5039 | 0.026394 |
| 5-Methoxydimethyltryptamine | | 2.4393 | 0.029804 |
| **\|Log2-fold change \| > 1** | | | |
| **Metabolites** | | **Fold Change** | **log2(FC)** |
| Undecanedioic acid | | 16.022 | 4.0019 |
| PA(16:0/16:0) | | 7.347 | 2.8771 |
| 3-Pyridylacetic acid | | 5.6011 | 2.4857 |
| ADP | | 5.1587 | 2.367 |
| D-Arginine | | 5.0132 | 2.3257 |
| L-Arginine | | 5.0132 | 2.3257 |
| Shikimic acid | | 4.8881 | 2.2893 |
| 11a-Hydroxyprogesterone | | 4.3041 | 2.1057 |
| 17-Hydroxyprogesterone | | 4.3041 | 2.1057 |
| Deoxycorticosterone | | 4.3041 | 2.1057 |
| Suberic acid | | 4.2132 | 2.0749 |
| Pyridoxamine | | 3.91 | 1.9672 |
| 2'-Deoxyguanosine 5'-monophosphate | | 3.792 | 1.923 |
| Adenosine monophosphate | | 3.792 | 1.923 |
| L-Histidine | | 3.7905 | 1.9224 |
| PC(16:0/16:0) | | 3.7589 | 1.9103 |
| Hexadecanedioic acid | | 3.7403 | 1.9032 |
| Tryptamine | | 3.7303 | 1.8993 |
| L-Cystathionine | | 3.502 | 1.8082 |
| L-Aspartyl-L-phenylalanine | | 3.3587 | 1.7479 |
| 5-Thymidylic acid | | 3.3062 | 1.7252 |
| 3-Cresotinic acid | | 3.2955 | 1.7205 |
| 4-Hydroxy-3-methylbenzoic acid | | 3.2955 | 1.7205 |
| Mandelic acid | | 3.2955 | 1.7205 |
| p-Anisic acid | | 3.2955 | 1.7205 |
| Inosine triphosphate | | 3.1618 | 1.6608 |
| Retinal | | 3.1311 | 1.6467 |
| D-Lysine | | 3.1161 | 1.6398 |
| L-Lysine | | 3.1161 | 1.6398 |
| Pipecolic acid | | 3.0499 | 1.6087 |
| Salicin | | 3.0361 | 1.6022 |
| Androstanedione | | 2.9069 | 1.5395 |
| Dehydroepiandrosterone | | 2.9069 | 1.5395 |
| Testosterone | | 2.9069 | 1.5395 |
| Ribonolactone | | 2.8178 | 1.4946 |
| Glucosamine 6-phosphate | | 2.7936 | 1.4821 |
| Indoxyl | | 2.7807 | 1.4755 |
| Elaidic acid | | 2.7627 | 1.4661 |
| Oleic acid | | 2.7627 | 1.4661 |
| Vaccenic acid | | 2.7627 | 1.4661 |
| 2-Phenylglycine | | 2.7557 | 1.4624 |
| Stearic acid | | 2.7417 | 1.4551 |
| Glutathione | | 2.665 | 1.4142 |
| Linoleic acid | | 2.6391 | 1.4 |
| L-Carnitine | | 2.5669 | 1.36 |
| Malonic acid | | 2.5626 | 1.3576 |
| Phenylglyoxylic acid | | 2.4282 | 1.2799 |
| 3,4-Dihydroxybenzeneacetic acid | | 2.3456 | 1.23 |
| Homogentisic acid | | 2.3456 | 1.23 |
| Vanillic acid | | 2.3456 | 1.23 |
| Biocytin | | 2.3309 | 1.2209 |
| Guanosine monophosphate | | 2.3134 | 1.21 |
| Ureidosuccinic acid | | 2.3129 | 1.2097 |
| Tryptophanol | | 2.3072 | 1.2061 |
| lipoic acid | | 2.298 | 1.2004 |
| Adrenaline | | 2.2772 | 1.1873 |
| Normetanephrine | | 2.2772 | 1.1873 |
| 3-Phosphoglyceric acid | | 2.2284 | 1.156 |
| Alpha-Lactose | | 2.2208 | 1.151 |
| Cellobiose | | 2.2208 | 1.151 |
| Isomaltose | | 2.2208 | 1.151 |
| Lactulose | | 2.2208 | 1.151 |
| Melibiose | | 2.2208 | 1.151 |
| Sucrose | | 2.2208 | 1.151 |
| Trehalose | | 2.2208 | 1.151 |
| Turanose | | 2.2208 | 1.151 |
| Urocanic acid | | 2.1963 | 1.135 |
| 3-Hydroxybenzoic acid | | 2.195 | 1.1342 |
| 4-Hydroxybenzoic acid | | 2.195 | 1.1342 |
| D-Glutamine | | 2.0826 | 1.0584 |
| L-Glutamine | | 2.0826 | 1.0584 |
| Arachidic acid | | 2.0417 | 1.0298 |
| Diaminopimelic acid | | 2.0252 | 1.0181 |
| 5-Phenylvaleric acid | | 2.005 | 1.0036 |
| Allantoic acid | | 2.0041 | 1.003 |
| Oxoglutaric acid | | 0.49511 | -1.0142 |
| Indoleacrylic acid | | 0.47697 | -1.068 |
| 1-Methylguanine | | 0.45648 | -1.1314 |
| 7-Methylguanine | | 0.45648 | -1.1314 |
| L-Methionine | | 0.45095 | -1.149 |
| L-Homoserine | | 0.43821 | -1.1903 |
| L-Threonine | | 0.43821 | -1.1903 |
| Niacinamide | | 0.4346 | -1.2022 |
| 5-Methoxytryptophan | | 0.38409 | -1.3805 |
| Traumatic acid | | 0.3781 | -1.4032 |
| Aminoadipic acid | | 0.34272 | -1.5449 |
| N-Acetyl-L-phenylalanine | | 0.30234 | -1.7258 |
| Homovanillic acid | | 0.29696 | -1.7517 |
| Hydroxyphenyllactic acid | | 0.29696 | -1.7517 |
| Methionine sulfoxide | | 0.28997 | -1.786 |
| 1-Methyladenosine | | 0.27847 | -1.8444 |
| Phenylpropiolic acid | | 0.25911 | -1.9483 |
| Beta-N-Acetylglucosamine | | 0.23794 | -2.0714 |
| N-Acetyl-D-glucosamine | | 0.23794 | -2.0714 |
| N-Acetylgalactosamine | | 0.23794 | -2.0714 |
| N-Acetylmannosamine | | 0.23794 | -2.0714 |
| L-Phenylalanine | | 0.23395 | -2.0957 |
| D-Sorbitol | | 0.21289 | -2.2318 |
| Galactitol | | 0.21289 | -2.2318 |
| Mannitol | | 0.21289 | -2.2318 |
| L-Tyrosine | | 0.17898 | -2.4821 |
| o-Tyrosine | | 0.17898 | -2.4821 |
| 3-Methoxytyramine | | 0.17389 | -2.5238 |
| Phenylephrine | | 0.17389 | -2.5238 |
| Perillic acid | | 0.10476 | -3.2548 |
| Phosphocreatine | | 0.058412 | -4.0976 |
| 4-Aminohippuric acid | | 0.044556 | -4.4882 |
| Guanosine | | 0.035846 | -4.8021 |
| L-Fucose | | 0.031691 | -4.9798 |
| Rhamnose | | 0.031691 | -4.9798 |
| Deoxyguanosine | | 0.016197 | -5.9481 |
| 1,3,7-Trimethyluric acid | | 0.012912 | -6.2752 |
| Nicotinuric acid | | 0.011694 | -6.4181 |
| Purine | | 0.005582 | -7.4849 |
| Allose | | 0.00504 | -7.6324 |
| Alpha-D-Glucose | | 0.00504 | -7.6324 |
| D-Fructose | | 0.00504 | -7.6324 |
| D-Galactose | | 0.00504 | -7.6324 |
| D-Glucose | | 0.00504 | -7.6324 |
| D-Mannose | | 0.00504 | -7.6324 |
| D-Tagatose | | 0.00504 | -7.6324 |
| L-Sorbose | | 0.00504 | -7.6324 |
| Myoinositol | | 0.00504 | -7.6324 |
| Scyllitol | | 0.00504 | -7.6324 |
| Deoxycytidine | | 0.0039 | -8.0025 |
| D-Xylose | | 0.003483 | -8.1655 |
| D-Xylulose | | 0.003483 | -8.1655 |
| Glycerophosphocholine | | 0.001023 | -9.9332 |
| **VIP>1** | | | |
| **Metabolites** | | **Comp. 1** | **Comp. 2** |
| 3-Pyridylacetic acid | | 1.3425 | 1.3404 |
| L-Arginine | | 1.3424 | 1.3402 |
| D-Arginine | | 1.3424 | 1.3402 |
| o-Tyrosine | | 1.3396 | 1.3377 |
| L-Tyrosine | | 1.3396 | 1.3377 |
| L-Lysine | | 1.3396 | 1.3374 |
| D-Lysine | | 1.3396 | 1.3374 |
| Mannitol | | 1.3384 | 1.3363 |
| Galactitol | | 1.3384 | 1.3363 |
| D-Sorbitol | | 1.3384 | 1.3363 |
| Pyridoxamine | | 1.3382 | 1.336 |
| Guanosine | | 1.336 | 1.3339 |
| N-Acetylmannosamine | | 1.3345 | 1.3323 |
| N-Acetylgalactosamine | | 1.3345 | 1.3323 |
| N-Acetyl-D-glucosamine | | 1.3345 | 1.3323 |
| Beta-N-Acetylglucosamine | | 1.3345 | 1.3323 |
| Indoleacrylic acid | | 1.3339 | 1.3319 |
| ADP | | 1.3328 | 1.3307 |
| 3-Methylindole | | 1.3328 | 1.3305 |
| Pipecolic acid | | 1.3317 | 1.3297 |
| PA(16:0/16:0) | | 1.3316 | 1.3296 |
| Deoxyguanosine | | 1.3297 | 1.3274 |
| Undecanedioic acid | | 1.3284 | 1.3262 |
| p-Anisic acid | | 1.3272 | 1.3251 |
| Mandelic acid | | 1.3272 | 1.3251 |
| 4-Hydroxy-3-methylbenzoic acid | | 1.3272 | 1.3251 |
| 3-Cresotinic acid | | 1.3272 | 1.3251 |
| Creatine | | 1.3266 | 1.3243 |
| Adenosine monophosphate | | 1.3257 | 1.3235 |
| 2'-Deoxyguanosine 5'-monophosphate | | 1.3257 | 1.3235 |
| Retinal | | 1.3243 | 1.3222 |
| L-Carnitine | | 1.3231 | 1.321 |
| L-Glutamine | | 1.3228 | 1.3206 |
| D-Glutamine | | 1.3228 | 1.3206 |
| 2-Phenylglycine | | 1.3228 | 1.3209 |
| Glutathione | | 1.3212 | 1.319 |
| 7-Ketocholesterol | | 1.3211 | 1.3192 |
| N-Acetyl-L-alanine | | 1.3199 | 1.3176 |
| 5-Aminolevulinic acid | | 1.3199 | 1.3176 |
| 4-Hydroxyproline | | 1.3199 | 1.3176 |
| Sebacic acid | | 1.3193 | 1.3173 |
| Phosphocreatine | | 1.3177 | 1.3157 |
| Perillic acid | | 1.3169 | 1.3149 |
| Phenylephrine | | 1.3164 | 1.3143 |
| 3-Methoxytyramine | | 1.3164 | 1.3143 |
| Vanillic acid | | 1.3161 | 1.3139 |
| Homogentisic acid | | 1.3161 | 1.3139 |
| 3,4-Dihydroxybenzeneacetic acid | | 1.3161 | 1.3139 |
| L-Methionine | | 1.3156 | 1.314 |
| Arachidic acid | | 1.3147 | 1.3127 |
| Phenylpropiolic acid | | 1.3135 | 1.3116 |
| Nutriacholic acid | | 1.3135 | 1.3113 |
| 7a-Hydroxy-3-oxo-5b-cholanoic acid | | 1.3135 | 1.3113 |
| L-Histidine | | 1.3129 | 1.3107 |
| Guanosine monophosphate | | 1.3122 | 1.3102 |
| lipoic acid | | 1.3101 | 1.3079 |
| Ureidosuccinic acid | | 1.31 | 1.3078 |
| Suberic acid | | 1.3069 | 1.3046 |
| N-Formyl-L-methionine | | 1.3058 | 1.3035 |
| N-Methyl-a-aminoisobutyric acid | | 1.3049 | 1.3045 |
| L-Valine | | 1.3049 | 1.3045 |
| Betaine | | 1.3049 | 1.3045 |
| 5-Aminopentanoic acid | | 1.3049 | 1.3045 |
| Monomethyl glutaric acid | | 1.3046 | 1.3027 |
| Methylglutaric acid | | 1.3046 | 1.3027 |
| 2-Methylglutaric acid | | 1.3046 | 1.3027 |
| 2,2-Dimethylsuccinic acid | | 1.3046 | 1.3027 |
| 1-Methyladenosine | | 1.3011 | 1.3001 |
| Sarcosine | | 1.3 | 1.2977 |
| L-Alanine | | 1.3 | 1.2977 |
| D-Alanine | | 1.3 | 1.2977 |
| Beta-Alanine | | 1.3 | 1.2977 |
| N-Acetyl-L-phenylalanine | | 1.2958 | 1.2936 |
| Inosinic acid | | 1.2925 | 1.2903 |
| Inosine | | 1.2918 | 1.2896 |
| Inosine triphosphate | | 1.2912 | 1.289 |
| 5-Thymidylic acid | | 1.291 | 1.2888 |
| L-Phenylalanine | | 1.2869 | 1.2851 |
| Sphinganine | | 1.2857 | 1.2858 |
| Methionine sulfoxide | | 1.2844 | 1.2822 |
| DL-2-Aminooctanoic acid | | 1.283 | 1.2811 |
| Shikimic acid | | 1.2798 | 1.2776 |
| Hexadecanedioic acid | | 1.2797 | 1.278 |
| Triamterene | | 1.2794 | 1.2772 |
| Leucinic acid | | 1.2762 | 1.2742 |
| 2-Hydroxycaproic acid | | 1.2762 | 1.2742 |
| 2-Ethyl-2-Hydroxybutyric acid | | 1.2762 | 1.2742 |
| Coproporphyrin I | | 1.2736 | 1.2714 |
| 3-Phosphoglyceric acid | | 1.2714 | 1.2694 |
| Pregnenolone sulfate | | 1.2645 | 1.2628 |
| Pyrrolidonecarboxylic acid | | 1.2613 | 1.2592 |
| Pyroglutamic acid | | 1.2613 | 1.2592 |
| Hydroxyphenyllactic acid | | 1.2583 | 1.2561 |
| Homovanillic acid | | 1.2583 | 1.2561 |
| Malonic acid | | 1.2572 | 1.2551 |
| L-Asparagine | | 1.2536 | 1.2515 |
| Glycyl-glycine | | 1.2536 | 1.2515 |
| Phenylglyoxylic acid | | 1.2426 | 1.2407 |
| Rhamnose | | 1.2413 | 1.2399 |
| L-Fucose | | 1.2413 | 1.2399 |
| Phthalic acid | | 1.2378 | 1.2357 |
| NADH | | 1.233 | 1.2312 |
| Tryptamine | | 1.2276 | 1.2257 |
| Urocanic acid | | 1.2271 | 1.225 |
| Adenosine | | 1.2209 | 1.2198 |
| Niacinamide | | 1.2204 | 1.2184 |
| 1,11-Undecanedicarboxylic acid | | 1.2142 | 1.2122 |
| Indoxyl | | 1.2087 | 1.2067 |
| Biocytin | | 1.2066 | 1.2046 |
| 3-Methoxytyrosine | | 1.2054 | 1.2044 |
| Allantoic acid | | 1.2043 | 1.2025 |
| L-Glutamic acid | | 1.2019 | 1.2022 |
| cis,cis-Muconic acid | | 1.1957 | 1.1942 |
| Xanthosine | | 1.1938 | 1.1925 |
| Normetanephrine | | 1.1906 | 1.1886 |
| Adrenaline | | 1.1906 | 1.1886 |
| L-Proline | | 1.1892 | 1.1873 |
| Flavin Mononucleotide | | 1.1869 | 1.1867 |
| 4-Hydroxybenzoic acid | | 1.1869 | 1.1849 |
| 3-Hydroxybenzoic acid | | 1.1869 | 1.1849 |
| Traumatic acid | | 1.1848 | 1.1846 |
| L-Acetylcarnitine | | 1.1838 | 1.1836 |
| L-Aspartyl-L-phenylalanine | | 1.1814 | 1.1794 |
| Ribonolactone | | 1.1806 | 1.1794 |
| Adenine | | 1.1803 | 1.1801 |
| 7-Methylguanine | | 1.1754 | 1.1734 |
| 1-Methylguanine | | 1.1754 | 1.1734 |
| Deoxycorticosterone | | 1.1751 | 1.1736 |
| 17-Hydroxyprogesterone | | 1.1751 | 1.1736 |
| 11a-Hydroxyprogesterone | | 1.1751 | 1.1736 |
| Isoxanthopterin | | 1.1733 | 1.1713 |
| Citrulline | | 1.169 | 1.167 |
| dGTP | | 1.1669 | 1.1654 |
| Adenosine triphosphate | | 1.1669 | 1.1654 |
| Uridine 5'-monophosphate | | 1.1506 | 1.1494 |
| Methylcysteine | | 1.1496 | 1.1478 |
| Glucosamine 6-phosphate | | 1.1439 | 1.142 |
| Glucaric acid | | 1.1421 | 1.1403 |
| Aminoadipic acid | | 1.1416 | 1.1401 |
| Creatinine | | 1.1373 | 1.1353 |
| Azelaic acid | | 1.1104 | 1.1086 |
| Uridine | | 1.1102 | 1.1087 |
| Turanose | | 1.1004 | 1.0992 |
| Trehalose | | 1.1004 | 1.0992 |
| Sucrose | | 1.1004 | 1.0992 |
| Melibiose | | 1.1004 | 1.0992 |
| Lactulose | | 1.1004 | 1.0992 |
| Isomaltose | | 1.1004 | 1.0992 |
| Cellobiose | | 1.1004 | 1.0992 |
| Alpha-Lactose | | 1.1004 | 1.0992 |
| Naproxen | | 1.0944 | 1.0928 |
| Guanosine diphosphate | | 1.093 | 1.0911 |
| L-Tryptophan | | 1.0884 | 1.0881 |
| Homo-L-arginine | | 1.0843 | 1.0829 |
| Glycerophosphocholine | | 1.0679 | 1.0732 |
| L-Serine | | 1.0638 | 1.0625 |
| PC(16:0/16:0) | | 1.0635 | 1.0629 |
| N-Acetylglutamic acid | | 1.0602 | 1.0584 |
| Scyllitol | | 1.0535 | 1.0594 |
| Myoinositol | | 1.0535 | 1.0594 |
| L-Sorbose | | 1.0535 | 1.0594 |
| D-Tagatose | | 1.0535 | 1.0594 |
| D-Mannose | | 1.0535 | 1.0594 |
| D-Glucose | | 1.0535 | 1.0594 |
| D-Galactose | | 1.0535 | 1.0594 |
| D-Fructose | | 1.0535 | 1.0594 |
| Alpha-D-Glucose | | 1.0535 | 1.0594 |
| Allose | | 1.0535 | 1.0594 |
| 5-Methoxytryptophan | | 1.0533 | 1.0516 |
| Nicotinuric acid | | 1.0526 | 1.0585 |
| Isocitric acid | | 1.0459 | 1.0441 |
| D-threo-Isocitric acid | | 1.0459 | 1.0441 |
| Citric acid | | 1.0459 | 1.0441 |
| Diaminopimelic acid | | 1.0458 | 1.044 |
| 1,3,7-Trimethyluric acid | | 1.0425 | 1.0485 |
| Medroxyprogesterone | | 1.0378 | 1.0362 |
| Malic acid | | 1.0272 | 1.0267 |
| L-Malic acid | | 1.0272 | 1.0267 |
| L-Cystathionine | | 1.0206 | 1.0192 |
| L-Threonine | | 1.0167 | 1.0152 |
| L-Homoserine | | 1.0167 | 1.0152 |
| Ursodeoxycholic acid | | 1.0142 | 1.0139 |
| Hyodeoxycholic acid | | 1.0142 | 1.0139 |
| Deoxycholic acid | | 1.0142 | 1.0139 |
| Chenodeoxycholic acid | | 1.0142 | 1.0139 |
| **Final selective markers from positive mode (109 metabolites)** | | | |
| 1,3,7-Trimethyluric acid | Biocytin | Lactulose | o-Tyrosine |
| 11a-Hydroxyprogesterone | Cellobiose | L-Arginine | PA(16:0/16:0) |
| 17-Hydroxyprogesterone | D-Arginine | L-Aspartyl-L-phenylalanine | p-Anisic acid |
| 1-Methyladenosine | Deoxycorticosterone | L-Carnitine | PC(16:0/16:0) |
| 1-Methylguanine | Deoxyguanosine | L-Cystathionine | Perillic acid |
| 2'-Deoxyguanosine 5'-monophosphate | D-Fructose | L-Glutamine | Phenylephrine |
| 2-Phenylglycine | D-Galactose | L-Histidine | Phenylglyoxylic acid |
| 3,4-Dihydroxybenzeneacetic acid | D-Glucose | L-Homoserine | Phenylpropiolic acid |
| 3-Cresotinic acid | D-Glutamine | lipoic acid | Phosphocreatine |
| 3-Hydroxybenzoic acid | Diaminopimelic acid | L-Lysine | Pipecolic acid |
| 3-Methoxytyramine | D-Lysine | L-Methionine | Pyridoxamine |
| 3-Phosphoglyceric acid | D-Mannose | L-Phenylalanine | Retinal |
| 3-Pyridylacetic acid | D-Sorbitol | L-Sorbose | Ribonolactone |
| 4-Hydroxy-3-methylbenzoic acid | D-Tagatose | L-Threonine | Scyllitol |
| 4-Hydroxybenzoic acid | Galactitol | L-Tyrosine | Shikimic acid |
| 5-Methoxytryptophan | Glucosamine 6-phosphate | Malonic acid | Suberic acid |
| 5-Thymidylic acid | Glutathione | Mandelic acid | Sucrose |
| 7-Methylguanine | Glycerophosphocholine | Mannitol | Traumatic acid |
| Adenosine monophosphate | Guanosine | Melibiose | Trehalose |
| ADP | Guanosine monophosphate | Methionine sulfoxide | Tryptamine |
| adrenaline | Hexadecanedioic acid | Myoinositol | Turanose |
| Allantoic acid | Homogentisic acid | N-Acetyl-D-glucosamine | Undecanedioic acid |
| Allose | Homovanillic acid | N-Acetylgalactosamine | Ureidosuccinic acid |
| Alpha-D-Glucose | Hydroxyphenyllactic acid | N-Acetyl-L-phenylalanine | Urocanic acid |
| Alpha-Lactose | Indoleacrylic acid | N-Acetylmannosamine | Vanillic acid |
| Aminoadipic acid | Indoxyl | Niacinamide |  |
| Arachidic acid | Inosine triphosphate | Nicotinuric acid |  |
| Beta-N-Acetylglucosamine | Isomaltose | Normetanephrine |  |

**Table S16** FSM rescued effects among the control, model and treatment groups from negative mode.

| **Metabolites** | **f.value** | **p.value** | **FDR** | **Fisher's LSD** |
| --- | --- | --- | --- | --- |
| 1,11-Undecanedicarboxylic acid | 22.941 | 1.14E-10 | 1.94E-10 | CON - fsm-h; CON - MODEL; fsm-l - fsm-h; fsm-m - fsm-h; fsm-h - MODEL; positive - fsm-h; fsm-l - MODEL; fsm-m - MODEL; positive - MODEL |
| 1,3-Dimethyluric acid | 73.082 | 9.59E-19 | 2.78E-18 | fsm-l - CON; CON - MODEL; positive - CON; fsm-l - fsm-h; fsm-h - MODEL; positive - fsm-h; fsm-l - fsm-m; fsm-l - MODEL; positive - fsm-l; fsm-m - MODEL; positive - fsm-m; positive - MODEL |
| 1,9-Dimethyluric acid | 73.082 | 9.59E-19 | 2.78E-18 | fsm-l - CON; CON - MODEL; positive - CON; fsm-l - fsm-h; fsm-h - MODEL; positive - fsm-h; fsm-l - fsm-m; fsm-l - MODEL; positive - fsm-l; fsm-m - MODEL; positive - fsm-m; positive - MODEL |
| 13-cis-Retinoic acid | 7.6074 | 4.56E-05 | 5.53E-05 | fsm-m - CON; CON - MODEL; fsm-h - MODEL; fsm-l - MODEL; fsm-m - MODEL; fsm-m - positive; positive - MODEL |
| 16-Dehydroprogesterone | 50.504 | 5.34E-16 | 1.32E-15 | MODEL - CON; MODEL - fsm-h; MODEL - fsm-l; MODEL - fsm-m; MODEL - positive |
| 1-Methylguanine | 117.07 | 2.12E-22 | 8.95E-22 | CON - fsm-h; CON - fsm-l; CON - fsm-m; CON - MODEL; CON - positive; fsm-m - fsm-h; fsm-h - MODEL; fsm-l - MODEL; fsm-l - positive; fsm-m - MODEL; fsm-m - positive; positive - MODEL |
| 2,2-Dimethylsuccinic acid | 641.55 | 2.49E-36 | 3.24E-35 | CON - fsm-h; CON - fsm-l; CON - fsm-m; CON - MODEL; CON - positive; fsm-l - fsm-h; fsm-m - fsm-h; fsm-h - MODEL; positive - fsm-h; fsm-l - MODEL; positive - fsm-l; fsm-m - MODEL; positive - fsm-m; positive - MODEL |
| 2,5-Furandicarboxylic acid | 4.5461 | 0.002319 | 0.002582 | fsm-m - CON; fsm-h - MODEL; fsm-l - MODEL; fsm-l - positive; fsm-m - MODEL; fsm-m - positive |
| 2-Chlorobenzoic acid | 8.5744 | 1.51E-05 | 1.92E-05 | fsm-h - CON; fsm-m - CON; CON - MODEL; fsm-h - MODEL; fsm-h - positive; fsm-l - MODEL; fsm-l - positive; fsm-m - MODEL; fsm-m - positive |
| 2-Isopropylmalic acid | 343.78 | 3.86E-31 | 2.44E-30 | MODEL - CON; MODEL - fsm-h; MODEL - fsm-l; MODEL - fsm-m; MODEL - positive |
| 2-Methylglutaric acid | 641.55 | 2.49E-36 | 3.24E-35 | CON - fsm-h; CON - fsm-l; CON - fsm-m; CON - MODEL; CON - positive; fsm-l - fsm-h; fsm-m - fsm-h; fsm-h - MODEL; positive - fsm-h; fsm-l - MODEL; positive - fsm-l; fsm-m - MODEL; positive - fsm-m; positive - MODEL |
| 2-Phenylglycine | 63.393 | 1.13E-17 | 3.02E-17 | MODEL - CON; MODEL - fsm-h; MODEL - fsm-l; MODEL - fsm-m; MODEL - positive |
| 3,4-Dihydroxybenzeneacetic acid | 1148 | 3.29E-41 | 1.76E-39 | fsm-h - CON; fsm-l - CON; fsm-m - CON; MODEL - CON; MODEL - fsm-h; MODEL - fsm-l; fsm-l - positive; MODEL - fsm-m; MODEL - positive |
| 3-Methoxybenzenepropanoic acid | 1354.9 | 1.33E-42 | 1.28E-40 | CON - fsm-h; CON - fsm-l; CON - fsm-m; MODEL - CON; MODEL - fsm-h; positive - fsm-h; MODEL - fsm-l; positive - fsm-l; MODEL - fsm-m; positive - fsm-m; MODEL - positive |
| 3-Methoxytyrosine | 16.658 | 8.67E-09 | 1.31E-08 | fsm-h - CON; fsm-l - CON; fsm-m - CON; fsm-l - fsm-h; fsm-h - MODEL; fsm-l - MODEL; fsm-l - positive; fsm-m - MODEL; fsm-m - positive; positive - MODEL |
| 3-Methyladenine | 423.18 | 7.32E-33 | 5.33E-32 | MODEL - CON; MODEL - fsm-h; MODEL - fsm-l; MODEL - fsm-m; MODEL - positive |
| 3-Methylindole | 423.22 | 7.31E-33 | 5.33E-32 | fsm-l - CON; MODEL - CON; positive - CON; fsm-l - fsm-h; MODEL - fsm-h; positive - fsm-h; fsm-l - fsm-m; MODEL - fsm-l; positive - fsm-l; MODEL - fsm-m; positive - fsm-m; MODEL - positive |
| 3-Phenoxypropionic acid | 841.47 | 1.34E-38 | 2.29E-37 | fsm-h - CON; fsm-l - CON; MODEL - CON; MODEL - fsm-h; fsm-h - positive; MODEL - fsm-l; fsm-l - positive; MODEL - fsm-m; MODEL - positive |
| 3-Phosphoglyceric acid | 40.098 | 2.35E-14 | 4.9E-14 | fsm-h - CON; CON - MODEL; CON - positive; fsm-h - fsm-l; fsm-h - fsm-m; fsm-h - MODEL; fsm-h - positive; fsm-l - MODEL; fsm-l - positive; fsm-m - MODEL; fsm-m - positive |
| 4-Aminohippuric acid | 120.17 | 1.32E-22 | 5.66E-22 | MODEL - CON; MODEL - fsm-h; MODEL - fsm-l; MODEL - fsm-m; MODEL - positive |
| 4-Hydroxycyclohexylcarboxylic acid | 61.858 | 1.72E-17 | 4.56E-17 | MODEL - CON; MODEL - fsm-h; MODEL - fsm-l; MODEL - fsm-m; MODEL - positive |
| 4-Hydroxyproline | 141.09 | 6.97E-24 | 3.16E-23 | fsm-l - CON; CON - MODEL; positive - CON; fsm-l - fsm-h; fsm-h - MODEL; positive - fsm-h; fsm-l - fsm-m; fsm-l - MODEL; positive - fsm-l; fsm-m - MODEL; positive - fsm-m; positive - MODEL |
| 5-Aminolevulinic acid | 141.09 | 6.97E-24 | 3.16E-23 | fsm-l - CON; CON - MODEL; positive - CON; fsm-l - fsm-h; fsm-h - MODEL; positive - fsm-h; fsm-l - fsm-m; fsm-l - MODEL; positive - fsm-l; fsm-m - MODEL; positive - fsm-m; positive - MODEL |
| 5-Aminopentanoic acid | 19.292 | 1.26E-09 | 2E-09 | CON - MODEL; CON - positive; fsm-l - fsm-h; fsm-h - MODEL; fsm-h - positive; fsm-l - MODEL; fsm-l - positive; fsm-m - MODEL; fsm-m - positive; MODEL - positive |
| 5-Methoxytryptophan | 1015.3 | 3.54E-40 | 7.1E-39 | CON - fsm-h; CON - fsm-m; MODEL - CON; MODEL - fsm-h; MODEL - fsm-l; MODEL - fsm-m; MODEL - positive |
| 5'-Methylthioadenosine | 37.418 | 7.12E-14 | 1.44E-13 | fsm-h - CON; fsm-l - CON; fsm-m - CON; CON - MODEL; positive - CON; fsm-m - fsm-h; fsm-h - MODEL; positive - fsm-h; fsm-l - MODEL; fsm-m - MODEL; positive - MODEL |
| 5-Thymidylic acid | 11.189 | 9.86E-07 | 1.38E-06 | fsm-l - CON; fsm-m - CON; CON - MODEL; positive - CON; fsm-m - fsm-h; fsm-h - MODEL; fsm-m - fsm-l; fsm-l - MODEL; fsm-m - MODEL; fsm-m - positive; positive - MODEL |
| 6-Dimethylaminopurine | 44.135 | 4.96E-15 | 1.1E-14 | MODEL - CON; MODEL - fsm-h; MODEL - fsm-l; MODEL - fsm-m; MODEL - positive |
| 6-Methyladenine | 423.18 | 7.32E-33 | 5.33E-32 | MODEL - CON; MODEL - fsm-h; MODEL - fsm-l; MODEL - fsm-m; MODEL - positive |
| 6-Phosphogluconic acid | 32.093 | 7.87E-13 | 1.51E-12 | fsm-h - CON; fsm-l - CON; fsm-m - CON; CON - MODEL; CON - positive; fsm-l - fsm-h; fsm-h - MODEL; fsm-h - positive; fsm-l - fsm-m; fsm-l - MODEL; fsm-l - positive; fsm-m - MODEL; fsm-m - positive; positive - MODEL |
| 7-Methylguanine | 117.07 | 2.12E-22 | 8.95E-22 | CON - fsm-h; CON - fsm-l; CON - fsm-m; CON - MODEL; CON - positive; fsm-m - fsm-h; fsm-h - MODEL; fsm-l - MODEL; fsm-l - positive; fsm-m - MODEL; fsm-m - positive; positive - MODEL |
| 7-Methylxanthine | 134.72 | 1.63E-23 | 7.19E-23 | fsm-h - CON; fsm-l - CON; fsm-m - CON; CON - MODEL; CON - positive; fsm-h - MODEL; fsm-h - positive; fsm-l - fsm-m; fsm-l - MODEL; fsm-l - positive; fsm-m - MODEL; fsm-m - positive |
| Adenosine 2',3'-cyclic phosphate | 8.2978 | 2.06E-05 | 2.6E-05 | CON - MODEL; fsm-h - MODEL; fsm-h - positive; fsm-l - MODEL; fsm-l - positive; fsm-m - MODEL; fsm-m - positive; positive - MODEL |
| Adenosine triphosphate | 61.836 | 1.73E-17 | 4.56E-17 | fsm-h - CON; fsm-l - CON; CON - MODEL; CON - positive; fsm-h - fsm-m; fsm-h - MODEL; fsm-h - positive; fsm-l - fsm-m; fsm-l - MODEL; fsm-l - positive; fsm-m - MODEL; fsm-m - positive |
| ADP | 137.77 | 1.08E-23 | 4.86E-23 | fsm-h - CON; fsm-l - CON; fsm-m - CON; CON - MODEL; positive - CON; fsm-h - fsm-l; fsm-h - fsm-m; fsm-h - MODEL; positive - fsm-h; fsm-l - MODEL; positive - fsm-l; fsm-m - MODEL; positive - fsm-m; positive - MODEL |
| Allantoic acid | 58.902 | 3.98E-17 | 1.04E-16 | fsm-h - CON; fsm-l - CON; fsm-m - CON; MODEL - CON; positive - CON; MODEL - fsm-h; fsm-l - fsm-m; MODEL - fsm-l; MODEL - fsm-m; positive - fsm-m; MODEL - positive |
| Allose | 525.23 | 1.17E-34 | 1.02E-33 | CON - fsm-h; CON - fsm-l; CON - fsm-m; CON - MODEL; CON - positive; fsm-l - fsm-h; fsm-h - MODEL; positive - fsm-h; fsm-l - fsm-m; fsm-l - MODEL; positive - fsm-l; fsm-m - MODEL; positive - fsm-m; positive - MODEL |
| All-trans-retinoic acid | 7.6074 | 4.56E-05 | 5.53E-05 | fsm-m - CON; CON - MODEL; fsm-h - MODEL; fsm-l - MODEL; fsm-m - MODEL; fsm-m - positive; positive - MODEL |
| Alpha-Aspartyl-lysine | 270.85 | 3.56E-29 | 2.04E-28 | MODEL - CON; MODEL - fsm-h; MODEL - fsm-l; fsm-l - positive; MODEL - fsm-m; fsm-m - positive; MODEL - positive |
| Alpha-D-Glucose | 525.23 | 1.17E-34 | 1.02E-33 | CON - fsm-h; CON - fsm-l; CON - fsm-m; CON - MODEL; CON - positive; fsm-l - fsm-h; fsm-h - MODEL; positive - fsm-h; fsm-l - fsm-m; fsm-l - MODEL; positive - fsm-l; fsm-m - MODEL; positive - fsm-m; positive - MODEL |
| Alpha-Hydroxyhippuric acid | 84.228 | 7.88E-20 | 2.58E-19 | fsm-l - CON; fsm-m - CON; CON - MODEL; positive - CON; fsm-l - fsm-h; fsm-m - fsm-h; fsm-h - MODEL; positive - fsm-h; fsm-l - MODEL; positive - fsm-l; fsm-m - MODEL; positive - MODEL |
| Alpha-Linolenic acid | 35.729 | 1.48E-13 | 2.93E-13 | CON - MODEL; positive - CON; fsm-h - MODEL; positive - fsm-h; positive - fsm-l; fsm-m - MODEL; positive - fsm-m; positive - MODEL |
| Arachidic acid | 15.554 | 2.07E-08 | 3.07E-08 | fsm-m - CON; CON - MODEL; fsm-h - MODEL; fsm-h - positive; fsm-l - MODEL; fsm-l - positive; fsm-m - MODEL; fsm-m - positive; positive - MODEL |
| Azelaic acid | 109.28 | 7.41E-22 | 2.95E-21 | CON - fsm-h; CON - fsm-l; CON - fsm-m; CON - MODEL; positive - CON; fsm-h - MODEL; positive - fsm-h; fsm-l - MODEL; positive - fsm-l; fsm-m - MODEL; positive - fsm-m; positive - MODEL |
| Benzenebutanoic acid | 93.439 | 1.24E-20 | 4.49E-20 | MODEL - CON; MODEL - fsm-h; MODEL - fsm-l; MODEL - fsm-m; MODEL - positive |
| Beta-Alanine | 273.26 | 3.01E-29 | 1.74E-28 | fsm-l - CON; MODEL - CON; positive - CON; MODEL - fsm-h; positive - fsm-h; MODEL - fsm-l; positive - fsm-l; MODEL - fsm-m; positive - fsm-m; MODEL - positive |
| Betaine | 19.292 | 1.26E-09 | 2E-09 | CON - MODEL; CON - positive; fsm-l - fsm-h; fsm-h - MODEL; fsm-h - positive; fsm-l - MODEL; fsm-l - positive; fsm-m - MODEL; fsm-m - positive; MODEL - positive |
| Bilirubin | 38.442 | 4.63E-14 | 9.51E-14 | MODEL - CON; MODEL - fsm-h; MODEL - fsm-l; MODEL - fsm-m; MODEL - positive |
| Biotin | 428.91 | 5.66E-33 | 4.39E-32 | MODEL - CON; MODEL - fsm-h; MODEL - fsm-l; MODEL - fsm-m; MODEL - positive |
| Canrenone | 18.991 | 1.55E-09 | 2.46E-09 | fsm-h - CON; fsm-l - CON; fsm-m - CON; CON - MODEL; positive - CON; fsm-h - MODEL; fsm-l - MODEL; fsm-m - MODEL; fsm-m - positive; positive - MODEL |
| Cholesterol sulfate | 6.0581 | 0.000306 | 0.000357 | fsm-m - CON; CON - MODEL; fsm-h - MODEL; fsm-l - MODEL; fsm-m - MODEL; fsm-m - positive; positive - MODEL |
| Citric acid | 87.853 | 3.73E-20 | 1.29E-19 | CON - fsm-h; CON - MODEL; CON - positive; fsm-l - fsm-h; fsm-m - fsm-h; fsm-h - MODEL; fsm-l - MODEL; fsm-m - MODEL; positive - MODEL |
| Cortexolone | 48.203 | 1.16E-15 | 2.77E-15 | MODEL - CON; positive - CON; MODEL - fsm-h; positive - fsm-h; MODEL - fsm-l; positive - fsm-l; MODEL - fsm-m; positive - fsm-m; MODEL - positive |
| Cortisone | 44.813 | 3.87E-15 | 8.65E-15 | MODEL - CON; MODEL - fsm-h; MODEL - fsm-l; MODEL - fsm-m; MODEL - positive |
| Creatinine | 45.124 | 3.45E-15 | 7.91E-15 | MODEL - CON; MODEL - fsm-h; MODEL - fsm-l; MODEL - fsm-m; MODEL - positive |
| Cyclic AMP | 8.2978 | 2.06E-05 | 2.6E-05 | CON - MODEL; fsm-h - MODEL; fsm-h - positive; fsm-l - MODEL; fsm-l - positive; fsm-m - MODEL; fsm-m - positive; positive - MODEL |
| Cytidine triphosphate | 51.273 | 4.15E-16 | 1.03E-15 | CON - fsm-m; CON - MODEL; CON - positive; fsm-h - MODEL; fsm-h - positive; fsm-l - MODEL; fsm-l - positive; fsm-m - MODEL; fsm-m - positive |
| D-Alanine | 273.26 | 3.01E-29 | 1.74E-28 | fsm-l - CON; MODEL - CON; positive - CON; MODEL - fsm-h; positive - fsm-h; MODEL - fsm-l; positive - fsm-l; MODEL - fsm-m; positive - fsm-m; MODEL - positive |
| D-Arginine | 84.852 | 6.91E-20 | 2.28E-19 | CON - fsm-h; CON - fsm-l; CON - fsm-m; CON - MODEL; CON - positive; fsm-h - MODEL; positive - fsm-h; positive - fsm-l; fsm-m - MODEL; positive - fsm-m; positive - MODEL |
| Deoxyadenosine monophosphate | 23.82 | 6.67E-11 | 1.15E-10 | CON - fsm-h; CON - fsm-l; CON - fsm-m; CON - MODEL; fsm-l - fsm-h; fsm-h - MODEL; positive - fsm-h; fsm-l - MODEL; positive - fsm-l; fsm-m - MODEL; positive - fsm-m; positive - MODEL |
| Desaminotyrosine | 841.47 | 1.34E-38 | 2.29E-37 | fsm-h - CON; fsm-l - CON; MODEL - CON; MODEL - fsm-h; fsm-h - positive; MODEL - fsm-l; fsm-l - positive; MODEL - fsm-m; MODEL - positive |
| D-Fructose | 525.23 | 1.17E-34 | 1.02E-33 | CON - fsm-h; CON - fsm-l; CON - fsm-m; CON - MODEL; CON - positive; fsm-l - fsm-h; fsm-h - MODEL; positive - fsm-h; fsm-l - fsm-m; fsm-l - MODEL; positive - fsm-l; fsm-m - MODEL; positive - fsm-m; positive - MODEL |
| D-Galactose | 525.23 | 1.17E-34 | 1.02E-33 | CON - fsm-h; CON - fsm-l; CON - fsm-m; CON - MODEL; CON - positive; fsm-l - fsm-h; fsm-h - MODEL; positive - fsm-h; fsm-l - fsm-m; fsm-l - MODEL; positive - fsm-l; fsm-m - MODEL; positive - fsm-m; positive - MODEL |
| dGTP | 61.836 | 1.73E-17 | 4.56E-17 | fsm-h - CON; fsm-l - CON; CON - MODEL; CON - positive; fsm-h - fsm-m; fsm-h - MODEL; fsm-h - positive; fsm-l - fsm-m; fsm-l - MODEL; fsm-l - positive; fsm-m - MODEL; fsm-m - positive |
| D-Lysine | 37.636 | 6.49E-14 | 1.32E-13 | CON - fsm-h; CON - fsm-l; CON - fsm-m; CON - MODEL; CON - positive; fsm-h - fsm-l; fsm-h - MODEL; fsm-h - positive; fsm-m - fsm-l; fsm-m - MODEL; fsm-m - positive |
| D-Mannose | 525.23 | 1.17E-34 | 1.02E-33 | CON - fsm-h; CON - fsm-l; CON - fsm-m; CON - MODEL; CON - positive; fsm-l - fsm-h; fsm-h - MODEL; positive - fsm-h; fsm-l - fsm-m; fsm-l - MODEL; positive - fsm-l; fsm-m - MODEL; positive - fsm-m; positive - MODEL |
| Dodecanedioic acid | 20.089 | 7.25E-10 | 1.18E-09 | CON - MODEL; CON - positive; fsm-m - fsm-h; fsm-h - MODEL; fsm-l - MODEL; fsm-m - MODEL; fsm-m - positive; positive - MODEL |
| Dodecanoic acid | 17.507 | 4.56E-09 | 7.05E-09 | CON - MODEL; positive - CON; fsm-h - MODEL; positive - fsm-h; fsm-l - MODEL; positive - fsm-l; fsm-m - MODEL; positive - fsm-m; positive - MODEL |
| Dopamine | 47.686 | 1.39E-15 | 3.27E-15 | MODEL - CON; MODEL - fsm-h; MODEL - fsm-l; MODEL - fsm-m; MODEL - positive |
| D-Pantethine | 64.598 | 8.18E-18 | 2.22E-17 | MODEL - CON; MODEL - fsm-h; MODEL - fsm-l; MODEL - fsm-m; MODEL - positive |
| D-Ribose 5-phosphate | 111.41 | 5.22E-22 | 2.13E-21 | MODEL - CON; MODEL - fsm-h; MODEL - fsm-l; MODEL - fsm-m; MODEL - positive |
| D-Sorbitol | 7.5551 | 4.85E-05 | 5.84E-05 | fsm-l - CON; fsm-m - CON; fsm-h - MODEL; fsm-h - positive; fsm-l - MODEL; fsm-l - positive; fsm-m - MODEL; fsm-m - positive |
| D-Tagatose | 525.23 | 1.17E-34 | 1.02E-33 | CON - fsm-h; CON - fsm-l; CON - fsm-m; CON - MODEL; CON - positive; fsm-l - fsm-h; fsm-h - MODEL; positive - fsm-h; fsm-l - fsm-m; fsm-l - MODEL; positive - fsm-l; fsm-m - MODEL; positive - fsm-m; positive - MODEL |
| D-threo-Isocitric acid | 87.853 | 3.73E-20 | 1.29E-19 | CON - fsm-h; CON - MODEL; CON - positive; fsm-l - fsm-h; fsm-m - fsm-h; fsm-h - MODEL; fsm-l - MODEL; fsm-m - MODEL; positive - MODEL |
| Elaidic acid | 6.7701 | 0.000125 | 0.000148 | positive - CON; fsm-h - MODEL; positive - fsm-h; positive - fsm-l; fsm-m - MODEL; positive - MODEL |
| Ethenodeoxyadenosine | 112.77 | 4.19E-22 | 1.74E-21 | CON - fsm-h; CON - MODEL; positive - CON; fsm-l - fsm-h; fsm-m - fsm-h; fsm-h - MODEL; positive - fsm-h; fsm-l - MODEL; positive - fsm-l; fsm-m - MODEL; positive - fsm-m; positive - MODEL |
| FAD | 43.463 | 6.38E-15 | 1.39E-14 | fsm-l - CON; CON - MODEL; positive - CON; fsm-l - fsm-h; fsm-h - MODEL; positive - fsm-h; fsm-l - MODEL; fsm-m - MODEL; positive - fsm-m; positive - MODEL |
| FAPy-adenine | 35.969 | 1.33E-13 | 2.66E-13 | MODEL - CON; MODEL - fsm-h; MODEL - fsm-l; MODEL - fsm-m; MODEL - positive |
| Flavin Mononucleotide | 21.774 | 2.38E-10 | 3.99E-10 | CON - MODEL; fsm-m - fsm-h; fsm-h - MODEL; fsm-l - MODEL; fsm-l - positive; fsm-m - MODEL; fsm-m - positive; positive - MODEL |
| Fructose 6-phosphate | 1099.3 | 7.61E-41 | 2.38E-39 | CON - fsm-h; CON - fsm-l; CON - fsm-m; CON - MODEL; CON - positive; fsm-h - fsm-l; fsm-h - MODEL; fsm-h - positive; fsm-l - MODEL; fsm-m - MODEL; positive - MODEL |
| Fumaric acid | 522.87 | 1.27E-34 | 1.09E-33 | fsm-l - CON; MODEL - CON; positive - CON; fsm-l - fsm-h; MODEL - fsm-h; positive - fsm-h; fsm-l - fsm-m; MODEL - fsm-l; MODEL - fsm-m; MODEL - positive |
| Galactitol | 7.5551 | 4.85E-05 | 5.84E-05 | fsm-l - CON; fsm-m - CON; fsm-h - MODEL; fsm-h - positive; fsm-l - MODEL; fsm-l - positive; fsm-m - MODEL; fsm-m - positive |
| Galactonic acid | 75.438 | 5.5E-19 | 1.68E-18 | fsm-l - CON; CON - MODEL; positive - CON; fsm-l - fsm-h; fsm-h - MODEL; positive - fsm-h; fsm-l - fsm-m; fsm-l - MODEL; positive - fsm-l; fsm-m - MODEL; positive - fsm-m; positive - MODEL |
| Galacturonic acid | 252.77 | 1.31E-28 | 7.34E-28 | MODEL - CON; MODEL - fsm-h; MODEL - fsm-l; MODEL - fsm-m; MODEL - positive |
| Glucaric acid | 146.52 | 3.48E-24 | 1.67E-23 | fsm-h - CON; fsm-l - CON; fsm-m - CON; CON - MODEL; CON - positive; fsm-h - fsm-l; fsm-h - fsm-m; fsm-h - MODEL; fsm-h - positive; fsm-l - MODEL; fsm-l - positive; fsm-m - MODEL; fsm-m - positive |
| Gluconic acid | 75.438 | 5.5E-19 | 1.68E-18 | fsm-l - CON; CON - MODEL; positive - CON; fsm-l - fsm-h; fsm-h - MODEL; positive - fsm-h; fsm-l - fsm-m; fsm-l - MODEL; positive - fsm-l; fsm-m - MODEL; positive - fsm-m; positive - MODEL |
| Gluconolactone | 551.94 | 4.5E-35 | 5.03E-34 | CON - fsm-h; CON - fsm-l; CON - fsm-m; CON - MODEL; CON - positive; fsm-l - fsm-h; fsm-h - MODEL; positive - fsm-h; fsm-l - fsm-m; fsm-l - MODEL; positive - fsm-l; fsm-m - MODEL; positive - fsm-m; positive - MODEL |
| Glucose 1-phosphate | 1099.3 | 7.61E-41 | 2.38E-39 | CON - fsm-h; CON - fsm-l; CON - fsm-m; CON - MODEL; CON - positive; fsm-h - fsm-l; fsm-h - MODEL; fsm-h - positive; fsm-l - MODEL; fsm-m - MODEL; positive - MODEL |
| Glucose 6-phosphate | 1099.3 | 7.61E-41 | 2.38E-39 | CON - fsm-h; CON - fsm-l; CON - fsm-m; CON - MODEL; CON - positive; fsm-h - fsm-l; fsm-h - MODEL; fsm-h - positive; fsm-l - MODEL; fsm-m - MODEL; positive - MODEL |
| Glycerol 3-phosphate | 319.33 | 1.57E-30 | 9.8E-30 | CON - fsm-h; CON - fsm-l; CON - fsm-m; CON - MODEL; CON - positive; fsm-h - MODEL; positive - fsm-h; fsm-l - MODEL; positive - fsm-l; fsm-m - MODEL; positive - fsm-m; positive - MODEL |
| Glycocholic acid | 23.208 | 9.68E-11 | 1.66E-10 | MODEL - CON; MODEL - fsm-h; MODEL - fsm-l; MODEL - fsm-m; MODEL - positive |
| Glycyl-glycine | 44.932 | 3.7E-15 | 8.36E-15 | CON - fsm-h; CON - fsm-l; CON - fsm-m; CON - MODEL; CON - positive; fsm-l - fsm-h; fsm-h - MODEL; fsm-h - positive; fsm-l - MODEL; fsm-l - positive; fsm-m - MODEL; fsm-m - positive |
| Guanidinosuccinic acid | 399.01 | 2.25E-32 | 1.62E-31 | MODEL - CON; positive - CON; MODEL - fsm-h; MODEL - fsm-l; MODEL - fsm-m; positive - fsm-m; MODEL - positive |
| Guanosine diphosphate | 76.611 | 4.2E-19 | 1.29E-18 | fsm-h - CON; fsm-l - CON; fsm-m - CON; CON - MODEL; positive - CON; fsm-h - MODEL; positive - fsm-h; fsm-l - MODEL; positive - fsm-l; fsm-m - MODEL; positive - fsm-m; positive - MODEL |
| Guanosine triphosphate | 32.606 | 6.16E-13 | 1.2E-12 | fsm-h - CON; fsm-l - CON; CON - MODEL; CON - positive; fsm-h - fsm-m; fsm-h - MODEL; fsm-h - positive; fsm-l - fsm-m; fsm-l - MODEL; fsm-l - positive; fsm-m - MODEL; fsm-m - positive |
| Guanosine | 23.153 | 1E-10 | 1.71E-10 | CON - MODEL; positive - CON; fsm-h - MODEL; positive - fsm-h; fsm-l - MODEL; positive - fsm-l; fsm-m - MODEL; positive - fsm-m; positive - MODEL |
| Hematoporphyrin IX | 19.446 | 1.13E-09 | 1.82E-09 | CON - fsm-h; CON - fsm-l; fsm-m - CON; CON - MODEL; positive - CON; fsm-m - fsm-h; positive - fsm-h; fsm-m - fsm-l; positive - fsm-l; fsm-m - MODEL; positive - MODEL |
| Heptadecanoic acid | 4.4568 | 0.002627 | 0.002919 | fsm-m - CON; fsm-h - MODEL; fsm-l - MODEL; fsm-m - MODEL; positive - MODEL |
| Hippuric acid | 16.65 | 8.72E-09 | 1.31E-08 | fsm-h - CON; fsm-l - CON; fsm-m - CON; CON - MODEL; fsm-h - MODEL; fsm-h - positive; fsm-l - MODEL; fsm-l - positive; fsm-m - MODEL; fsm-m - positive; positive - MODEL |
| Homogentisic acid | 1148 | 3.29E-41 | 1.76E-39 | fsm-h - CON; fsm-l - CON; fsm-m - CON; MODEL - CON; MODEL - fsm-h; MODEL - fsm-l; fsm-l - positive; MODEL - fsm-m; MODEL - positive |
| Hydroxyphenyllactic acid | 132.51 | 2.21E-23 | 9.56E-23 | fsm-h - CON; fsm-l - CON; fsm-m - CON; MODEL - CON; positive - CON; fsm-h - fsm-l; fsm-h - MODEL; fsm-h - positive; fsm-m - fsm-l; fsm-l - MODEL; fsm-l - positive; fsm-m - MODEL; fsm-m - positive; MODEL - positive |
| Indole-carbinol | 1049.1 | 1.88E-40 | 4.11E-39 | MODEL - CON; MODEL - fsm-h; MODEL - fsm-l; MODEL - fsm-m; MODEL - positive |
| Indoleacetic acid | 462.8 | 1.32E-33 | 1.08E-32 | MODEL - CON; MODEL - fsm-h; MODEL - fsm-l; MODEL - fsm-m; MODEL - positive |
| Indolelactic acid | 54.456 | 1.51E-16 | 3.78E-16 | MODEL - CON; MODEL - fsm-h; MODEL - fsm-l; MODEL - fsm-m; MODEL - positive |
| Indoxyl | 954.82 | 1.16E-39 | 2.24E-38 | fsm-h - CON; fsm-l - CON; fsm-m - CON; MODEL - CON; positive - CON; fsm-h - fsm-m; MODEL - fsm-h; fsm-h - positive; MODEL - fsm-l; fsm-l - positive; MODEL - fsm-m; fsm-m - positive; MODEL - positive |
| Inosine triphosphate | 25.112 | 3.11E-11 | 5.46E-11 | fsm-m - CON; CON - MODEL; CON - positive; fsm-m - fsm-h; fsm-h - MODEL; fsm-h - positive; fsm-m - fsm-l; fsm-l - MODEL; fsm-l - positive; fsm-m - MODEL; fsm-m - positive |
| Inosine | 56.949 | 7.07E-17 | 1.78E-16 | CON - fsm-h; CON - fsm-m; CON - MODEL; positive - CON; fsm-l - fsm-h; fsm-h - MODEL; positive - fsm-h; fsm-l - MODEL; positive - fsm-l; fsm-m - MODEL; positive - fsm-m; positive - MODEL |
| Isocitric acid | 87.853 | 3.73E-20 | 1.29E-19 | CON - fsm-h; CON - MODEL; CON - positive; fsm-l - fsm-h; fsm-m - fsm-h; fsm-h - MODEL; fsm-l - MODEL; fsm-m - MODEL; positive - MODEL |
| L-Phenyllactic acid | 841.47 | 1.34E-38 | 2.29E-37 | fsm-h - CON; fsm-l - CON; MODEL - CON; MODEL - fsm-h; fsm-h - positive; MODEL - fsm-l; fsm-l - positive; MODEL - fsm-m; MODEL - positive |
| L-Acetylcarnitine | 178.63 | 8.83E-26 | 4.57E-25 | MODEL - CON; MODEL - fsm-h; MODEL - fsm-l; MODEL - fsm-m; MODEL - positive |
| L-Alanine | 273.26 | 3.01E-29 | 1.74E-28 | fsm-l - CON; MODEL - CON; positive - CON; MODEL - fsm-h; positive - fsm-h; MODEL - fsm-l; positive - fsm-l; MODEL - fsm-m; positive - fsm-m; MODEL - positive |
| L-Arginine | 84.852 | 6.91E-20 | 2.28E-19 | CON - fsm-h; CON - fsm-l; CON - fsm-m; CON - MODEL; CON - positive; fsm-h - MODEL; positive - fsm-h; positive - fsm-l; fsm-m - MODEL; positive - fsm-m; positive - MODEL |
| L-Asparagine | 44.932 | 3.7E-15 | 8.36E-15 | CON - fsm-h; CON - fsm-l; CON - fsm-m; CON - MODEL; CON - positive; fsm-l - fsm-h; fsm-h - MODEL; fsm-h - positive; fsm-l - MODEL; fsm-l - positive; fsm-m - MODEL; fsm-m - positive |
| L-Aspartyl-L-phenylalanine | 68.878 | 2.69E-18 | 7.62E-18 | CON - fsm-h; CON - fsm-l; CON - fsm-m; CON - MODEL; CON - positive; fsm-m - fsm-h; fsm-l - MODEL; fsm-l - positive; fsm-m - MODEL; fsm-m - positive |
| Linoleic acid | 6.8034 | 0.00012 | 0.000143 | positive - CON; fsm-h - MODEL; positive - fsm-h; fsm-l - MODEL; positive - fsm-l; fsm-m - MODEL; positive - MODEL |
| Lipoamide | 46.145 | 2.39E-15 | 5.53E-15 | fsm-h - CON; fsm-l - CON; fsm-m - CON; CON - MODEL; fsm-h - MODEL; fsm-l - MODEL; fsm-l - positive; fsm-m - MODEL; fsm-m - positive; positive - MODEL |
| L-Lysine | 37.636 | 6.49E-14 | 1.32E-13 | CON - fsm-h; CON - fsm-l; CON - fsm-m; CON - MODEL; CON - positive; fsm-h - fsm-l; fsm-h - MODEL; fsm-h - positive; fsm-m - fsm-l; fsm-m - MODEL; fsm-m - positive |
| L-Malic acid | 57.578 | 5.86E-17 | 1.48E-16 | fsm-h - CON; fsm-l - CON; fsm-m - CON; CON - MODEL; positive - CON; fsm-l - fsm-h; fsm-h - MODEL; positive - fsm-h; fsm-l - fsm-m; fsm-l - MODEL; positive - fsm-l; fsm-m - MODEL; positive - fsm-m; positive - MODEL |
| L-Methionine | 99.316 | 4.16E-21 | 1.57E-20 | fsm-h - CON; fsm-l - CON; fsm-m - CON; CON - MODEL; CON - positive; fsm-l - fsm-h; fsm-m - fsm-h; fsm-h - MODEL; fsm-h - positive; fsm-l - MODEL; fsm-l - positive; fsm-m - MODEL; fsm-m - positive |
| L-Phenylalanine | 83.136 | 9.93E-20 | 3.18E-19 | CON - fsm-h; CON - fsm-l; CON - fsm-m; CON - MODEL; CON - positive; fsm-l - fsm-h; fsm-h - MODEL; fsm-l - MODEL; fsm-m - MODEL; positive - MODEL |
| L-Proline | 29.794 | 2.45E-12 | 4.5E-12 | CON - MODEL; CON - positive; fsm-l - fsm-h; fsm-h - MODEL; fsm-h - positive; fsm-l - MODEL; fsm-l - positive; fsm-m - MODEL; fsm-m - positive |
| L-Sorbose | 525.23 | 1.17E-34 | 1.02E-33 | CON - fsm-h; CON - fsm-l; CON - fsm-m; CON - MODEL; CON - positive; fsm-l - fsm-h; fsm-h - MODEL; positive - fsm-h; fsm-l - fsm-m; fsm-l - MODEL; positive - fsm-l; fsm-m - MODEL; positive - fsm-m; positive - MODEL |
| L-Tryptophan | 259.55 | 7.97E-29 | 4.51E-28 | CON - fsm-h; CON - fsm-m; CON - MODEL; positive - CON; fsm-l - fsm-h; fsm-m - fsm-h; fsm-h - MODEL; positive - fsm-h; fsm-l - fsm-m; fsm-l - MODEL; positive - fsm-l; fsm-m - MODEL; positive - fsm-m; positive - MODEL |
| L-Tyrosine | 15.413 | 2.32E-08 | 3.42E-08 | fsm-h - CON; fsm-l - CON; fsm-m - CON; CON - MODEL; fsm-h - MODEL; fsm-h - positive; fsm-l - MODEL; fsm-l - positive; fsm-m - MODEL; fsm-m - positive; positive - MODEL |
| L-Valine | 19.292 | 1.26E-09 | 2E-09 | CON - MODEL; CON - positive; fsm-l - fsm-h; fsm-h - MODEL; fsm-h - positive; fsm-l - MODEL; fsm-l - positive; fsm-m - MODEL; fsm-m - positive; MODEL - positive |
| Malic acid | 136.83 | 1.23E-23 | 5.46E-23 | fsm-h - CON; fsm-l - CON; fsm-m - CON; CON - MODEL; CON - positive; fsm-h - MODEL; fsm-h - positive; fsm-l - MODEL; fsm-l - positive; fsm-m - MODEL; fsm-m - positive; positive - MODEL |
| Mannitol | 7.5551 | 4.85E-05 | 5.84E-05 | fsm-l - CON; fsm-m - CON; fsm-h - MODEL; fsm-h - positive; fsm-l - MODEL; fsm-l - positive; fsm-m - MODEL; fsm-m - positive |
| Mannose 6-phosphate | 1099.3 | 7.61E-41 | 2.38E-39 | CON - fsm-h; CON - fsm-l; CON - fsm-m; CON - MODEL; CON - positive; fsm-h - fsm-l; fsm-h - MODEL; fsm-h - positive; fsm-l - MODEL; fsm-m - MODEL; positive - MODEL |
| m-Chlorobenzoic acid | 8.6783 | 1.34E-05 | 1.72E-05 | fsm-h - CON; fsm-m - CON; CON - MODEL; fsm-h - MODEL; fsm-h - positive; fsm-l - MODEL; fsm-l - positive; fsm-m - MODEL; fsm-m - positive |
| Menadione | 427.23 | 6.1E-33 | 4.66E-32 | MODEL - CON; MODEL - fsm-h; MODEL - fsm-l; MODEL - fsm-m; MODEL - positive |
| Methionine sulfoxide | 60.953 | 2.22E-17 | 5.8E-17 | MODEL - CON; MODEL - fsm-h; MODEL - fsm-l; MODEL - fsm-m; MODEL - positive |
| Methylcysteine | 110.88 | 5.69E-22 | 2.3E-21 | MODEL - CON; positive - CON; MODEL - fsm-h; MODEL - fsm-l; MODEL - fsm-m; MODEL - positive |
| Methylglutaric acid | 641.55 | 2.49E-36 | 3.24E-35 | CON - fsm-h; CON - fsm-l; CON - fsm-m; CON - MODEL; CON - positive; fsm-l - fsm-h; fsm-m - fsm-h; fsm-h - MODEL; positive - fsm-h; fsm-l - MODEL; positive - fsm-l; fsm-m - MODEL; positive - fsm-m; positive - MODEL |
| Monomethyl glutaric acid | 641.55 | 2.49E-36 | 3.24E-35 | CON - fsm-h; CON - fsm-l; CON - fsm-m; CON - MODEL; CON - positive; fsm-l - fsm-h; fsm-m - fsm-h; fsm-h - MODEL; positive - fsm-h; fsm-l - MODEL; positive - fsm-l; fsm-m - MODEL; positive - fsm-m; positive - MODEL |
| Myoinositol | 525.23 | 1.17E-34 | 1.02E-33 | CON - fsm-h; CON - fsm-l; CON - fsm-m; CON - MODEL; CON - positive; fsm-l - fsm-h; fsm-h - MODEL; positive - fsm-h; fsm-l - fsm-m; fsm-l - MODEL; positive - fsm-l; fsm-m - MODEL; positive - fsm-m; positive - MODEL |
| Myristic acid | 4.8757 | 0.001469 | 0.001647 | CON - MODEL; fsm-h - MODEL; fsm-l - MODEL; fsm-m - MODEL; fsm-m - positive; positive - MODEL |
| N-Acetyl-L-alanine | 141.09 | 6.97E-24 | 3.16E-23 | fsm-l - CON; CON - MODEL; positive - CON; fsm-l - fsm-h; fsm-h - MODEL; positive - fsm-h; fsm-l - fsm-m; fsm-l - MODEL; positive - fsm-l; fsm-m - MODEL; positive - fsm-m; positive - MODEL |
| N-Acetylneuraminic acid | 79.475 | 2.2E-19 | 6.83E-19 | fsm-l - CON; CON - fsm-m; CON - MODEL; fsm-l - fsm-h; fsm-h - MODEL; fsm-l - fsm-m; fsm-l - MODEL; fsm-l - positive; fsm-m - MODEL; positive - fsm-m; positive - MODEL |
| N-Acetylserotonin | 453.1 | 1.98E-33 | 1.59E-32 | MODEL - CON; MODEL - fsm-h; MODEL - fsm-l; MODEL - fsm-m; MODEL - positive |
| NADH | 48.851 | 9.3E-16 | 2.23E-15 | CON - fsm-h; CON - fsm-l; CON - fsm-m; CON - MODEL; fsm-l - fsm-h; fsm-h - MODEL; positive - fsm-h; fsm-l - MODEL; positive - fsm-l; fsm-m - MODEL; positive - fsm-m; positive - MODEL |
| Nicotinuric acid | 479.44 | 6.71E-34 | 5.56E-33 | CON - fsm-h; CON - fsm-l; CON - fsm-m; CON - MODEL; CON - positive; fsm-l - fsm-h; fsm-m - fsm-h; fsm-h - MODEL; positive - fsm-h; fsm-l - MODEL; positive - fsm-l; fsm-m - MODEL; positive - fsm-m; positive - MODEL |
| N-Methyl-a-aminoisobutyric acid | 19.292 | 1.26E-09 | 2E-09 | CON - MODEL; CON - positive; fsm-l - fsm-h; fsm-h - MODEL; fsm-h - positive; fsm-l - MODEL; fsm-l - positive; fsm-m - MODEL; fsm-m - positive; MODEL - positive |
| Oleic acid | 6.7701 | 0.000125 | 0.000148 | positive - CON; fsm-h - MODEL; positive - fsm-h; positive - fsm-l; fsm-m - MODEL; positive - MODEL |
| o-Tyrosine | 15.413 | 2.32E-08 | 3.42E-08 | fsm-h - CON; fsm-l - CON; fsm-m - CON; CON - MODEL; fsm-h - MODEL; fsm-h - positive; fsm-l - MODEL; fsm-l - positive; fsm-m - MODEL; fsm-m - positive; positive - MODEL |
| Oxoglutaric acid | 99.155 | 4.28E-21 | 1.61E-20 | CON - fsm-h; CON - fsm-l; CON - fsm-m; CON - MODEL; CON - positive; fsm-h - fsm-l; fsm-h - fsm-m; fsm-h - MODEL; fsm-h - positive; fsm-l - MODEL; fsm-l - positive; fsm-m - MODEL; fsm-m - positive |
| Pantothenic acid | 23.855 | 6.53E-11 | 1.13E-10 | fsm-l - CON; fsm-m - CON; CON - MODEL; positive - CON; fsm-l - fsm-h; fsm-h - MODEL; positive - fsm-h; fsm-l - MODEL; positive - fsm-l; fsm-m - MODEL; positive - fsm-m; positive - MODEL |
| Pentadecanoic acid | 7.8011 | 3.64E-05 | 4.45E-05 | fsm-m - CON; CON - MODEL; fsm-h - MODEL; fsm-l - MODEL; fsm-m - MODEL; fsm-m - positive; positive - MODEL |
| Perillic acid | 111.96 | 4.77E-22 | 1.96E-21 | MODEL - CON; MODEL - fsm-h; MODEL - fsm-l; MODEL - fsm-m; MODEL - positive |
| Phenylglyoxylic acid | 40.312 | 2.16E-14 | 4.52E-14 | CON - fsm-l; CON - MODEL; positive - CON; fsm-h - MODEL; positive - fsm-h; positive - fsm-l; fsm-m - MODEL; positive - fsm-m; positive - MODEL |
| Phenylpropiolic acid | 92.13 | 1.6E-20 | 5.69E-20 | CON - fsm-h; CON - fsm-l; CON - fsm-m; CON - MODEL; CON - positive; fsm-h - MODEL; fsm-l - MODEL; fsm-l - positive; fsm-m - MODEL; positive - MODEL |
| Phosphocreatine | 219 | 1.95E-27 | 1.04E-26 | fsm-h - CON; fsm-l - CON; fsm-m - CON; CON - MODEL; CON - positive; fsm-h - MODEL; fsm-h - positive; fsm-l - MODEL; fsm-l - positive; fsm-m - MODEL; fsm-m - positive |
| Phthalic acid | 47.817 | 1.33E-15 | 3.14E-15 | MODEL - CON; MODEL - fsm-h; MODEL - fsm-l; MODEL - fsm-m; MODEL - positive |
| Quinaldic acid | 41.902 | 1.16E-14 | 2.44E-14 | MODEL - CON; MODEL - fsm-h; MODEL - fsm-l; MODEL - fsm-m; MODEL - positive |
| Quinic acid | 529.61 | 9.95E-35 | 1.02E-33 | MODEL - CON; MODEL - fsm-h; MODEL - fsm-l; MODEL - fsm-m; MODEL - positive |
| Rhamnose | 97.498 | 5.79E-21 | 2.13E-20 | CON - fsm-h; CON - fsm-l; CON - fsm-m; CON - MODEL; CON - positive; fsm-l - fsm-h; fsm-h - MODEL; fsm-l - fsm-m; fsm-l - MODEL; fsm-l - positive; fsm-m - MODEL; positive - MODEL |
| Ribonolactone | 96.491 | 6.98E-21 | 2.54E-20 | CON - fsm-h; CON - fsm-l; CON - fsm-m; CON - MODEL; CON - positive; fsm-h - fsm-l; fsm-h - fsm-m; fsm-h - MODEL; fsm-h - positive; fsm-l - MODEL; fsm-l - positive; fsm-m - MODEL; fsm-m - positive |
| Sarcosine | 273.26 | 3.01E-29 | 1.74E-28 | fsm-l - CON; MODEL - CON; positive - CON; MODEL - fsm-h; positive - fsm-h; MODEL - fsm-l; positive - fsm-l; MODEL - fsm-m; positive - fsm-m; MODEL - positive |
| Scyllitol | 525.23 | 1.17E-34 | 1.02E-33 | CON - fsm-h; CON - fsm-l; CON - fsm-m; CON - MODEL; CON - positive; fsm-l - fsm-h; fsm-h - MODEL; positive - fsm-h; fsm-l - fsm-m; fsm-l - MODEL; positive - fsm-l; fsm-m - MODEL; positive - fsm-m; positive - MODEL |
| Sebacic acid | 37.537 | 6.77E-14 | 1.37E-13 | CON - fsm-h; CON - fsm-l; CON - fsm-m; CON - MODEL; CON - positive; fsm-l - fsm-h; fsm-m - fsm-h; fsm-h - MODEL; fsm-l - MODEL; fsm-l - positive; fsm-m - MODEL; fsm-m - positive; positive - MODEL |
| Shikimic acid | 41.919 | 1.15E-14 | 2.43E-14 | MODEL - CON; MODEL - fsm-h; MODEL - fsm-l; MODEL - fsm-m; MODEL - positive |
| Stearic acid | 12.292 | 3.46E-07 | 4.93E-07 | fsm-h - CON; fsm-l - CON; fsm-m - CON; CON - MODEL; fsm-h - MODEL; fsm-l - MODEL; fsm-m - MODEL; fsm-m - positive; positive - MODEL |
| Taurodeoxycholic acid | 38.983 | 3.7E-14 | 7.64E-14 | MODEL - CON; MODEL - fsm-h; MODEL - fsm-l; MODEL - fsm-m; MODEL - positive |
| Theobromine | 489.52 | 4.5E-34 | 3.8E-33 | CON - fsm-h; CON - fsm-l; CON - fsm-m; CON - MODEL; CON - positive; fsm-l - fsm-h; fsm-h - MODEL; positive - fsm-h; fsm-l - fsm-m; fsm-l - MODEL; positive - fsm-l; fsm-m - MODEL; positive - fsm-m; positive - MODEL |
| Thymidine | 42.134 | 1.06E-14 | 2.25E-14 | MODEL - CON; MODEL - fsm-h; MODEL - fsm-l; MODEL - fsm-m; MODEL - positive |
| Traumatic acid | 12.186 | 3.82E-07 | 5.43E-07 | CON - MODEL; CON - positive; fsm-m - fsm-h; fsm-h - MODEL; fsm-l - MODEL; fsm-m - MODEL; fsm-m - positive; positive - MODEL |
| Tryptamine | 7.339 | 6.27E-05 | 7.5E-05 | CON - fsm-h; CON - MODEL; positive - fsm-h; fsm-l - MODEL; fsm-m - MODEL; positive - MODEL |
| Uracil | 235.69 | 4.91E-28 | 2.71E-27 | MODEL - CON; MODEL - fsm-h; MODEL - fsm-l; MODEL - fsm-m; MODEL - positive |
| Uridine 5'-diphosphate | 35.561 | 1.59E-13 | 3.14E-13 | fsm-h - CON; fsm-m - CON; CON - MODEL; CON - positive; fsm-h - fsm-l; fsm-h - fsm-m; fsm-h - MODEL; fsm-h - positive; fsm-m - fsm-l; fsm-l - MODEL; fsm-m - MODEL; fsm-m - positive; positive - MODEL |
| Uridine diphosphate glucose | 79.67 | 2.11E-19 | 6.58E-19 | fsm-h - CON; CON - MODEL; CON - positive; fsm-h - fsm-l; fsm-h - fsm-m; fsm-h - MODEL; fsm-h - positive; fsm-l - MODEL; fsm-l - positive; fsm-m - MODEL; fsm-m - positive; positive - MODEL |
| Vaccenic acid | 6.7701 | 0.000125 | 0.000148 | positive - CON; fsm-h - MODEL; positive - fsm-h; positive - fsm-l; fsm-m - MODEL; positive - MODEL |
| Vanillic acid | 1148 | 3.29E-41 | 1.76E-39 | fsm-h - CON; fsm-l - CON; fsm-m - CON; MODEL - CON; MODEL - fsm-h; MODEL - fsm-l; fsm-l - positive; MODEL - fsm-m; MODEL - positive |
| Xanthosine | 36.183 | 1.21E-13 | 2.43E-13 | CON - fsm-h; CON - fsm-l; CON - fsm-m; CON - MODEL; CON - positive; fsm-h - MODEL; fsm-h - positive; fsm-l - MODEL; fsm-l - positive; fsm-m - MODEL; fsm-m - positive; MODEL - positive |

**Table S17** FSM rescued effects among the control, model and treatment groups from positive mode.

| **Metabolite** | **f.value** | **p.value** | **FDR** | **Fisher's LSD** |
| --- | --- | --- | --- | --- |
| 1,3,7-Trimethyluric acid | 4.5426 | 0.002591 | 0.003464 | model - con; model - fsm-h; model - fsm-l; model - positive |
| 1-Methyladenosine | 111.49 | 7.19E-21 | 6.36E-20 | model - con; positive - con; model - fsm-h; positive - fsm-h; model - fsm-l; positive - fsm-l; model - fsm-m; positive - fsm-m; model - positive |
| 1-Methylguanine | 11.08 | 1.64E-06 | 3.76E-06 | fsm-h - con; fsm-l - con; fsm-m - con; model - con; positive - con; fsm-l - fsm-h; fsm-h - positive; fsm-l - fsm-m; fsm-l - model; fsm-l - positive; model - positive |
| 3-Methoxytyramine | 316 | 1.12E-28 | 2.43E-27 | model - con; positive - con; model - fsm-h; positive - fsm-h; model - fsm-l; positive - fsm-l; model - fsm-m; positive - fsm-m; positive - model |
| 3-Pyridylacetic acid | 177.61 | 2.57E-24 | 3.84E-23 | fsm-h - con; fsm-l - con; fsm-m - con; con - model; positive - con; fsm-l - fsm-h; fsm-m - fsm-h; fsm-h - model; positive - fsm-h; fsm-m - fsm-l; fsm-l - model; fsm-m - model; fsm-m - positive; positive - model |
| 7-Methylguanine | 11.08 | 1.64E-06 | 3.76E-06 | fsm-h - con; fsm-l - con; fsm-m - con; model - con; positive - con; fsm-l - fsm-h; fsm-h - positive; fsm-l - fsm-m; fsm-l - model; fsm-l - positive; model - positive |
| ADP | 216.47 | 8.35E-26 | 1.62E-24 | con - fsm-h; con - fsm-l; con - fsm-m; con - model; con - positive; fsm-h - fsm-l; fsm-h - fsm-m; fsm-h - model; positive - fsm-h; positive - fsm-l; positive - fsm-m; positive - model |
| Adrenaline | 9.4569 | 8.00E-06 | 1.61E-05 | con - fsm-h; con - fsm-l; con - fsm-m; con - model; con - positive; fsm-l - fsm-h; fsm-m - fsm-h; fsm-l - positive; fsm-m - positive |
| Allose | 4.5455 | 0.002581 | 0.003462 | model - con; model - fsm-h; model - fsm-l; model - positive |
| Alpha-D-Glucose | 4.5455 | 0.002581 | 0.003462 | model - con; model - fsm-h; model - fsm-l; model - positive |
| Aminoadipic acid | 9.5765 | 7.09E-06 | 1.46E-05 | fsm-h - con; fsm-l - con; fsm-m - con; model - con; positive - con; fsm-l - fsm-h; model - fsm-h; fsm-l - fsm-m; model - fsm-m; model - positive |
| Arachidic acid | 21.149 | 8.13E-10 | 2.85E-09 | con - fsm-h; con - fsm-l; con - fsm-m; con - model; con - positive; positive - fsm-h; positive - fsm-l; positive - model |
| Beta-N-Acetylglucosamine | 384.28 | 3.59E-30 | 9.69E-29 | model - con; positive - con; model - fsm-h; positive - fsm-h; model - fsm-l; positive - fsm-l; model - fsm-m; positive - fsm-m; model - positive |
| Deoxyguanosine | 76.388 | 3.81E-18 | 3.09E-17 | fsm-h - con; fsm-l - con; fsm-m - con; model - con; positive - con; model - fsm-h; positive - fsm-h; model - fsm-l; positive - fsm-l; model - fsm-m; positive - fsm-m; positive - model |
| D-Fructose | 4.5455 | 0.002581 | 0.003462 | model - con; model - fsm-h; model - fsm-l; model - positive |
| D-Galactose | 4.5455 | 0.002581 | 0.003462 | model - con; model - fsm-h; model - fsm-l; model - positive |
| D-Glucose | 4.5455 | 0.002581 | 0.003462 | model - con; model - fsm-h; model - fsm-l; model - positive |
| D-Mannose | 4.5455 | 0.002581 | 0.003462 | model - con; model - fsm-h; model - fsm-l; model - positive |
| D-Sorbitol | 13.132 | 2.64E-07 | 6.75E-07 | fsm-h - con; fsm-l - con; fsm-m - con; model - con; positive - con; fsm-l - fsm-h; fsm-m - fsm-h; model - fsm-h; fsm-l - positive; fsm-m - positive; model - positive |
| D-Tagatose | 4.5455 | 0.002581 | 0.003462 | model - con; model - fsm-h; model - fsm-l; model - positive |
| Galactitol | 13.132 | 2.64E-07 | 6.75E-07 | fsm-h - con; fsm-l - con; fsm-m - con; model - con; positive - con; fsm-l - fsm-h; fsm-m - fsm-h; model - fsm-h; fsm-l - positive; fsm-m - positive; model - positive |
| Glycerophosphocholine | 5.7775 | 0.000515 | 0.000789 | model - con; model - fsm-h; model - fsm-l; model - positive |
| Guanosine monophosphate | 69.466 | 1.78E-17 | 1.26E-16 | con - fsm-h; con - fsm-l; con - fsm-m; con - model; con - positive; positive - fsm-h; positive - fsm-l; positive - fsm-m; positive - model |
| Guanosine | 18.558 | 4.35E-09 | 1.40E-08 | fsm-h - con; fsm-l - con; fsm-m - con; model - con; positive - con; fsm-l - fsm-h; model - fsm-h; positive - fsm-h; model - fsm-l; model - fsm-m; model - positive |
| Homovanillic acid | 4.9073 | 0.001588 | 0.002247 | fsm-m - con; model - con; positive - con; model - fsm-h; model - fsm-l; positive - fsm-l |
| Hydroxyphenyllactic acid | 4.9073 | 0.001588 | 0.002247 | fsm-m - con; model - con; positive - con; model - fsm-h; model - fsm-l; positive - fsm-l |
| Indoleacrylic acid | 57.981 | 3.23E-16 | 2.16E-15 | fsm-l - con; model - con; positive - con; model - fsm-h; positive - fsm-h; fsm-l - fsm-m; model - fsm-l; model - fsm-m; positive - fsm-m; model - positive |
| L-Histidine | 118.78 | 2.47E-21 | 2.40E-20 | con - fsm-h; con - fsm-l; con - fsm-m; con - model; con - positive; fsm-h - fsm-l; fsm-h - fsm-m; fsm-h - model; fsm-h - positive; fsm-l - positive; fsm-m - model; fsm-m - positive |
| L-Homoserine | 6.4738 | 0.000218 | 0.000358 | fsm-h - con; fsm-l - con; fsm-m - con; model - con; positive - con; fsm-l - fsm-m; fsm-l - positive |
| L-Phenylalanine | 18.015 | 6.31E-09 | 1.95E-08 | fsm-h - con; fsm-l - con; fsm-m - con; model - con; positive - con; fsm-l - fsm-h; fsm-l - fsm-m; fsm-l - positive; model - fsm-m; model - positive |
| L-Sorbose | 4.5455 | 0.002581 | 0.003462 | model - con; model - fsm-h; model - fsm-l; model - positive |
| L-Threonine | 6.4738 | 0.000218 | 0.000358 | fsm-h - con; fsm-l - con; fsm-m - con; model - con; positive - con; fsm-l - fsm-m; fsm-l - positive |
| Mannitol | 13.132 | 2.64E-07 | 6.75E-07 | fsm-h - con; fsm-l - con; fsm-m - con; model - con; positive - con; fsm-l - fsm-h; fsm-m - fsm-h; model - fsm-h; fsm-l - positive; fsm-m - positive; model - positive |
| Myoinositol | 4.5455 | 0.002581 | 0.003462 | model - con; model - fsm-h; model - fsm-l; model - positive |
| N-Acetyl-D-glucosamine | 384.28 | 3.59E-30 | 9.69E-29 | model - con; positive - con; model - fsm-h; positive - fsm-h; model - fsm-l; positive - fsm-l; model - fsm-m; positive - fsm-m; model - positive |
| N-Acetylgalactosamine | 384.28 | 3.59E-30 | 9.69E-29 | model - con; positive - con; model - fsm-h; positive - fsm-h; model - fsm-l; positive - fsm-l; model - fsm-m; positive - fsm-m; model - positive |
| N-Acetylmannosamine | 384.28 | 3.59E-30 | 9.69E-29 | model - con; positive - con; model - fsm-h; positive - fsm-h; model - fsm-l; positive - fsm-l; model - fsm-m; positive - fsm-m; model - positive |
| Niacinamide | 9.5515 | 7.27E-06 | 1.49E-05 | fsm-h - con; fsm-l - con; fsm-m - con; model - con; fsm-m - fsm-h; model - fsm-h; fsm-m - fsm-l; model - fsm-l; fsm-m - positive; model - positive |
| Nicotinuric acid | 4.5132 | 0.002697 | 0.003581 | model - con; model - fsm-h; model - fsm-l; model - positive |
| Perillic acid | 205.48 | 2.06E-25 | 3.82E-24 | model - con; positive - con; model - fsm-h; positive - fsm-h; model - fsm-l; positive - fsm-l; model - fsm-m; positive - fsm-m; positive - model |
| Phenylephrine | 316 | 1.12E-28 | 2.43E-27 | model - con; positive - con; model - fsm-h; positive - fsm-h; model - fsm-l; positive - fsm-l; model - fsm-m; positive - fsm-m; positive - model |
| Scyllitol | 4.5455 | 0.002581 | 0.003462 | model - con; model - fsm-h; model - fsm-l; model - positive |
| Traumatic acid | 20.181 | 1.49E-09 | 4.94E-09 | model - con; positive - con; fsm-l - fsm-h; model - fsm-h; positive - fsm-h; fsm-l - fsm-m; model - fsm-l; model - fsm-m; positive - fsm-m; model - positive |
| Undecanedioic acid | 22.738 | 3.12E-10 | 1.11E-09 | con - fsm-h; con - fsm-l; con - fsm-m; con - model; con - positive; fsm-h - fsm-l; fsm-h - model; fsm-m - fsm-l; fsm-l - model; positive - fsm-l; fsm-m - model; positive - model |

**Table S18** 194 FSM therapeutic markers.

| NO. | Metabolites | NO. | Metabolites | NO. | Metabolites |
| --- | --- | --- | --- | --- | --- |
| 1 | 1,11-Undecanedicarboxylic acid | 67 | Dodecanedioic acid | 133 | Methylglutaric acid |
| 2 | 1,3-Dimethyluric acid | 68 | Dodecanoic acid | 134 | Monomethyl glutaric acid |
| 3 | 1,9-Dimethyluric acid | 69 | Dopamine | 135 | Myoinositol |
| 4 | 13-cis-Retinoic acid | 70 | D-Pantethine | 136 | Myristic acid |
| 5 | 16-Dehydroprogesterone | 71 | D-Ribose 5-phosphate | 137 | N-Acetyl-L-alanine |
| 6 | 1-Methylguanine | 72 | D-Sorbitol | 138 | N-Acetylneuraminic acid |
| 7 | 2,2-Dimethylsuccinic acid | 73 | D-Tagatose | 139 | N-Acetylserotonin |
| 8 | 2,5-Furandicarboxylic acid | 74 | D-threo-Isocitric acid | 140 | NADH |
| 9 | 2-Chlorobenzoic acid | 75 | Elaidic acid | 141 | Nicotinuric acid |
| 10 | 2-Isopropylmalic acid | 76 | Ethenodeoxyadenosine | 142 | N-Methyl-a-aminoisobutyric acid |
| 11 | 2-Methylglutaric acid | 77 | FAD | 143 | Oleic acid |
| 12 | 2-Phenylglycine | 78 | FAPy-adenine | 144 | o-Tyrosine |
| 13 | 3,4-Dihydroxybenzeneacetic acid | 79 | Flavin Mononucleotide | 145 | Oxoglutaric acid |
| 14 | 3-Methoxybenzenepropanoic acid | 80 | Fructose 6-phosphate | 146 | Pantothenic acid |
| 15 | 3-Methoxytyrosine | 81 | Fumaric acid | 147 | Pentadecanoic acid |
| 16 | 3-Methyladenine | 82 | Galactitol | 148 | Perillic acid |
| 17 | 3-Methylindole | 83 | Galactonic acid | 149 | Phenylglyoxylic acid |
| 18 | 3-Phenoxypropionic acid | 84 | Galacturonic acid | 150 | Phenylpropiolic acid |
| 19 | 3-Phosphoglyceric acid | 85 | Glucaric acid | 151 | Phosphocreatine |
| 20 | 4-Aminohippuric acid | 86 | Gluconic acid | 152 | Phthalic acid |
| 21 | 4-Hydroxycyclohexylcarboxylic acid | 87 | Gluconolactone | 153 | Quinaldic acid |
| 22 | 4-Hydroxyproline | 88 | Glucose 1-phosphate | 154 | Quinic acid |
| 23 | 5-Aminolevulinic acid | 89 | Glucose 6-phosphate | 155 | Rhamnose |
| 24 | 5-Aminopentanoic acid | 90 | Glycerol 3-phosphate | 156 | Ribonolactone |
| 25 | 5-Methoxytryptophan | 91 | Glycocholic acid | 157 | Sarcosine |
| 26 | 5'-Methylthioadenosine | 92 | Glycyl-glycine | 158 | Scyllitol |
| 27 | 5-Thymidylic acid | 93 | Guanidinosuccinic acid | 159 | Sebacic acid |
| 28 | 6-Dimethylaminopurine | 94 | Guanosine diphosphate | 160 | Shikimic acid |
| 29 | 6-Methyladenine | 95 | Guanosine triphosphate | 161 | Stearic acid |
| 30 | 6-Phosphogluconic acid | 96 | Guanosine | 162 | Taurodeoxycholic acid |
| 31 | 7-Methylguanine | 97 | Hematoporphyrin IX | 163 | Theobromine |
| 32 | 7-Methylxanthine | 98 | Heptadecanoic acid | 164 | Thymidine |
| 33 | Adenosine 2',3'-cyclic phosphate | 99 | Hippuric acid | 165 | Traumatic acid |
| 34 | Adenosine triphosphate | 100 | Homogentisic acid | 166 | Tryptamine |
| 35 | ADP | 101 | Hydroxyphenyllactic acid | 167 | Uracil |
| 36 | Allantoic acid | 102 | Indole-carbinol | 168 | Uridine 5'-diphosphate |
| 37 | Allose | 103 | Indoleacetic acid | 169 | Uridine diphosphate glucose |
| 38 | All-trans-retinoic acid | 104 | Indolelactic acid | 170 | Vaccenic acid |
| 39 | Alpha-Aspartyl-lysine | 105 | Indoxyl | 171 | Vanillic acid |
| 40 | Alpha-D-Glucose | 106 | Inosine triphosphate | 172 | Xanthosine |
| 41 | Alpha-Hydroxyhippuric acid | 107 | Inosine | 173 | 1,3,7-Trimethyluric acid |
| 42 | Alpha-Linolenic acid | 108 | Isocitric acid | 174 | 1-Methyladenosine |
| 43 | Arachidic acid | 109 | L-Phenyllactic acid | 175 | 3-Methoxytyramine |
| 44 | Azelaic acid | 110 | L-Acetylcarnitine | 176 | 3-Pyridylacetic acid |
| 45 | Benzenebutanoic acid | 111 | L-Alanine | 177 | adrenaline |
| 46 | Beta-Alanine | 112 | L-Arginine | 178 | Aminoadipic acid |
| 47 | Betaine | 113 | L-Asparagine | 179 | Beta-N-Acetylglucosamine |
| 48 | Bilirubin | 114 | L-Aspartyl-L-phenylalanine | 180 | Deoxyguanosine |
| 49 | Biotin | 115 | Linoleic acid | 181 | D-Glucose |
| 50 | Canrenone | 116 | Lipoamide | 182 | Glycerophosphocholine |
| 51 | Cholesterol sulfate | 117 | L-Lysine | 183 | Guanosine monophosphate |
| 52 | Citric acid | 118 | L-Malic acid | 184 | Homovanillic acid |
| 53 | Cortexolone | 119 | L-Methionine | 185 | Indoleacrylic acid |
| 54 | Cortisone | 120 | L-Phenylalanine | 186 | L-Histidine |
| 55 | Creatinine | 121 | L-Proline | 187 | L-Homoserine |
| 56 | Cyclic AMP | 122 | L-Sorbose | 188 | L-Threonine |
| 57 | Cytidine triphosphate | 123 | L-Tryptophan | 189 | N-Acetyl-D-glucosamine |
| 58 | D-Alanine | 124 | L-Tyrosine | 190 | N-Acetylgalactosamine |
| 59 | D-Arginine | 125 | L-Valine | 191 | N-Acetylmannosamine |
| 60 | Deoxyadenosine monophosphate | 126 | Malic acid | 192 | Niacinamide |
| 61 | Desaminotyrosine | 127 | Mannitol | 193 | Phenylephrine |
| 62 | D-Fructose | 128 | Mannose 6-phosphate | 194 | Undecanedioic acid |
| 63 | D-Galactose | 129 | m-Chlorobenzoic acid |  |  |
| 64 | dGTP | 130 | Menadione |  |  |
| 65 | D-Lysine | 131 | Methionine sulfoxide |  |  |
| 66 | D-Mannose | 132 | Methylcysteine |  |  |

**Table S19** Pathway analysis from the FSM therapeutic metabolomic markers (Top 10 pathway, the pathway of either negative or positive ion metabolite markers with the cutoff value of Holm p <0.05 is highlighted in yellow. The pathway of combined metabolite markers with the cutoff value of Holm p <0.05 and Impact > 0.1 is highlighted in yellow).

| NO. | Pathway Name | Match Status | p | -log(p) | Holm p | FDR | Impact |
| --- | --- | --- | --- | --- | --- | --- | --- |
| Pathway enrichment analysis of negative ion data (172 metabolite markers) | | | | | | | |
| 1 | Purine metabolism | 13\66 | 3.99E-05 | 4.3986 | 0.003355 | 0.003355 | 0.08142 |
| 2 | Aminoacyl-tRNA biosynthesis | 10\48 | 2.10E-04 | 3.6786 | 0.017399 | 0.008804 | 0 |
| 3 | Galactose metabolism | 7\27 | 4.95E-04 | 3.3055 | 0.04058 | 0.013856 | 0.09435 |
| 4 | Citrate cycle (TCA cycle) | 5\20 | 0.00399 | 2.399 | 0.32319 | 0.082873 | 0.26785 |
| 5 | Amino sugar and nucleotide sugar metabolism | 7/39 | 0.004933 | 2.3069 | 0.39463 | 0.082873 | 0.22222 |
| 6 | Ascorbate and aldarate metabolism | 3\9 | 0.011327 | 1.9459 | 0.89487 | 0.13353 | 0 |
| 7 | Starch and sucrose metabolism | 4\17 | 0.012703 | 1.8961 | 0.99081 | 0.13353 | 0.31566 |
| 8 | Alanine, aspartate and glutamate metabolism | 5\27 | 0.015214 | 1.8177 | 1 | 0.13353 | 0.05899 |
| 9 | Pantothenate and CoA biosynthesis | 4\18 | 0.015643 | 1.8057 | 1 | 0.13353 | 0 |
| 10 | Riboflavin metabolism | 2\4 | 0.017487 | 1.7573 | 1 | 0.13353 | 0.5 |
| Pathway enrichment analysis of positive ion data (44 metabolite markers) | | | | | | | |
| 1 | Galactose metabolism | 5\27 | 5.29E-05 | 4.2761 | 0.004448 | 0.004448 | 0.08212 |
| 2 | Amino sugar and nucleotide sugar metabolism | 5\39 | 3.30E-04 | 3.4813 | 0.027401 | 0.013866 | 0.1352 |
| 3 | Purine metabolism | 4\66 | 0.021546 | 1.6666 | 1 | 0.60328 | 0.07693 |
| 4 | Tyrosine metabolism | 3\42 | 0.030661 | 1.5134 | 1 | 0.60569 | 0.03002 |
| 5 | Fructose and mannose metabolism | 2\20 | 0.042298 | 1.3737 | 1 | 0.60569 | 0.03313 |
| 6 | Aminoacyl-tRNA biosynthesis | 3\48 | 0.043263 | 1.3639 | 1 | 0.60569 | 0 |
| 7 | Phenylalanine, tyrosine and tryptophan biosynthesis | 1\4 | 0.065217 | 1.1856 | 1 | 0.71503 | 0.5 |
| 8 | Glycolysis / Gluconeogenesis | 2\26 | 0.068098 | 1.1669 | 1 | 0.71503 | 0.00105 |
| 9 | Valine, leucine and isoleucine biosynthesis | 1\8 | 0.12635 | 0.89843 | 1 | 1 | 0 |
| 10 | Phenylalanine metabolism | 1\8 | 0.12635 | 0.89843 | 1 | 1 | 0.35714 |
| Pathway enrichment analysis of combined data (194 metabolite markers) | | | | | | | |
| 1 | Purine metabolism | 15\66 | 1.11E-05 | 4.9549 | 0.000932 | 0.000932 | 0.13985 |
| 2 | Aminoacyl-tRNA biosynthesis | 12\48 | 3.34E-05 | 4.4768 | 0.002769 | 0.001401 | 0 |
| 3 | Amino sugar and nucleotide sugar metabolism | 9\39 | 0.000667 | 3.1762 | 0.054654 | 0.018662 | 0.35742 |
| 4 | Galactose metabolism | 7\27 | 0.001316 | 2.8807 | 0.10661 | 0.027638 | 0.09435 |
| 5 | Tyrosine metabolism | 8\42 | 0.004922 | 2.3078 | 0.39379 | 0.082695 | 0.39511 |
| 6 | Citrate cycle (TCA cycle) | 5\20 | 0.007955 | 2.0994 | 0.62843 | 0.11137 | 0.26785 |
| 7 | Ascorbate and aldarate metabolism | 3\9 | 0.017563 | 1.7554 | 1 | 0.19971 | 0 |
| 8 | Starch and sucrose metabolism | 4\17 | 0.021905 | 1.6595 | 1 | 0.19971 | 0.31566 |
| 9 | Riboflavin metabolism | 2\4 | 0.023775 | 1.6239 | 1 | 0.19971 | 0.5 |
| 10 | Phenylalanine, tyrosine and tryptophan biosynthesis | 2\4 | 0.023775 | 1.6239 | 1 | 0.19971 | 1 |

**
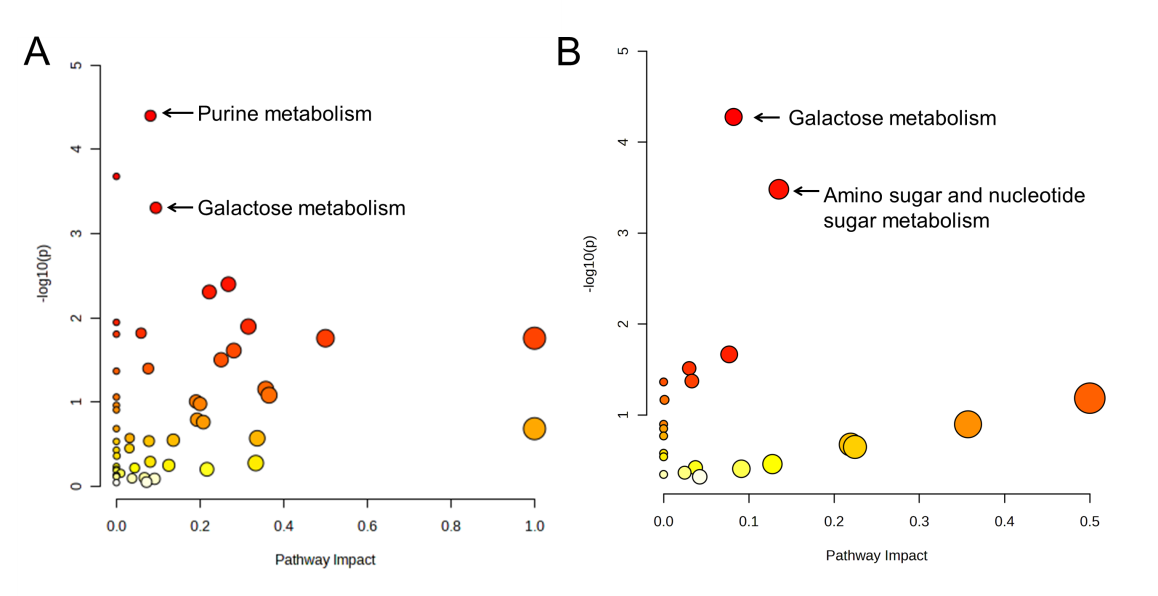
**

**Figure S2** Pathway analysis of FSM therapeutic markers of (A) negative ion and (B) positive ion.
