# Supplementary material for: Fushenmu treatment ameliorates RyR2 with related metabolites in a zebrafish model of barium chloride induced arrhythmia
Source: Chin Med. 2023 Aug 19;18:103. doi: 10.1186/s13020-023-00812-x (PMC10439546; doi:10.1186/s13020-023-00812-x)
Supplement: Supplementary file 6 — Additional file 6: Table S20. Joint pathway analysis from the FSM therapeutic ‘target- metabolite’ markers (Top15, Holm p < 0.05, Impact > 0.2). Table S21. Metabolites levels of adrenaline and cAMP. Figure S3. Adrenergic signaling in cardiomyocytes. Highly enrichment modules were pointed in red. C00575: Cyclic AMP, C00788: adrenaline. Figure S4. cAMP signaling pathway. Highly enrichment modules were pointed in red. C00575: Cyclic AMP, C00788: adrenaline. [file 13020_2023_812_MOESM6_ESM.docx]

**Additional File 6 for FSM therapeutic pathway location**

**Table S20** Joint pathway analysis from the FSM therapeutic ‘target- metabolite’ markers (Top15, Holm p <0.05, Impact > 0.2).

| pathway | Pathway Name | (M.,G.)/Total | p value | -log(p) | Holm p | FDR | Impact |
| --- | --- | --- | --- | --- | --- | --- | --- |
| 1 | ABC transporters | (2, 24)/183 | 2.65E-17 | 16.576 | 8.78E-15 | 8.78E-15 | 0 |
| 2 | Central carbon metabolism in cancer | (1, 19)/106 | 1.75E-16 | 15.758 | 5.77E-14 | 2.89E-14 | 0.053097 |
| 3 | Adrenergic signaling in cardiomyocytes | (2, 15)/159 | 3.72E-09 | 8.4293 | 1.22E-06 | 4.11E-07 | 0.43662 |
| 4 | Protein digestion and absorption | (1, 14)/142 | 7.14E-09 | 8.1462 | 2.34E-06 | 5.91E-07 | 0 |
| 5 | Aminoacyl-tRNA biosynthesis | (1, 12)/118 | 6.28E-08 | 7.2018 | 2.05E-05 | 3.50E-06 | 0.12371 |
| 6 | Purine metabolism | (3, 16)/225 | 6.34E-08 | 7.1976 | 2.07E-05 | 3.50E-06 | 0.55446 |
| 7 | Galactose metabolism | (1, 10)/77 | 7.96E-08 | 7.0993 | 2.59E-05 | 3.76E-06 | 0.39437 |
| 8 | Glucagon signaling pathway | (1, 12)/132 | 2.18E-07 | 6.6608 | 7.08E-05 | 9.04E-06 | 0.21687 |
| 9 | Insulin secretion | (1, 10)/98 | 7.96E-07 | 6.099 | 2.57E-04 | 2.93E-05 | 0.2807 |
| 10 | Dopaminergic synapse | (1, 11)/143 | 3.73E-06 | 5.4281 | 0.001198 | 1.12E-04 | 0.33333 |
| 11 | cAMP signaling pathway | (3, 13)/241 | 2.38E-05 | 4.6228 | 0.007533 | 4.93E-04 | 0.2459 |
| 12 | Cholinergic synapse | (1, 9)/124 | 4.64E-05 | 4.3333 | 0.014577 | 8.54E-04 | 0.34426 |
| 13 | Citrate cycle (TCA cycle) | (0, 6)/50 | 5.50E-05 | 4.2596 | 0.017215 | 9.58E-04 | 0.72727 |
| 14 | Oxytocin signaling pathway | (2, 10)/165 | 8.17E-05 | 4.0877 | 0.025333 | 0.00123 | 0.23684 |
| 15 | Tyrosine metabolism | (1, 8)/114 | 1.57E-04 | 3.8055 | 0.048203 | 0.002158 | 0.22581 |

**Table S21** Metabolites levels of adrenaline and cAMP.

|  | **Adrenaline** | **cAMP** |
| --- | --- | --- |
| Control | 1441.83±393.42 | 70.05±50.52 |
| Model | 2987.56±639.71 | 4234.22±665.19 |
| Positive | 1854.67±220.14 | 1313.81±173.84 |
| FSM-H | 2523.33±1506.50 | 2716.33±1740.40 |
| FSM-M | 3030.67±1388.21 | 4013.63±2878.76 |
| FSM-L | 2223.5±605.74 | 3208.65±2167.99 |


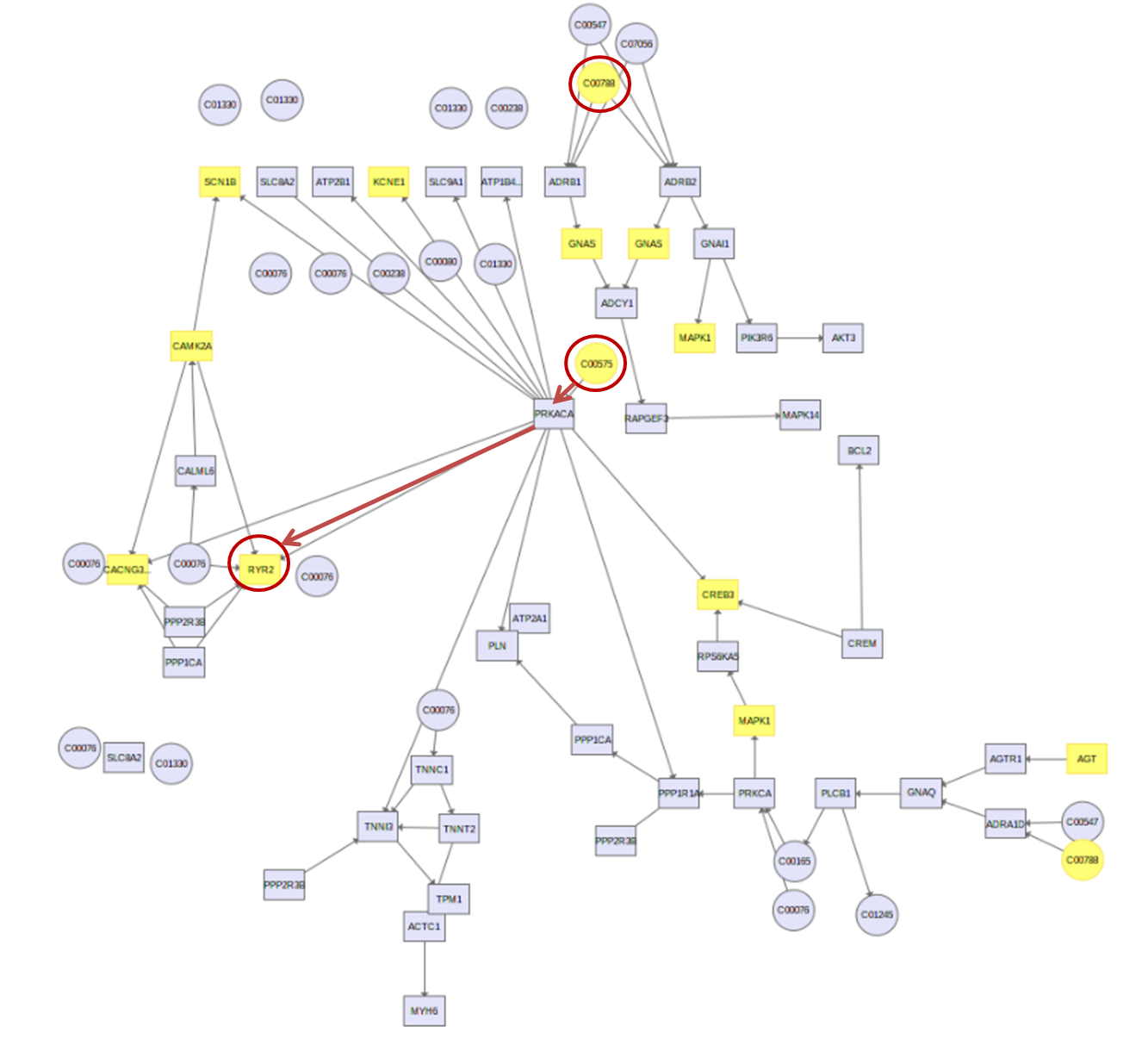


**Figure S3** Adrenergic signaling in cardiomyocytes. Highly enrichment modules were pointed in red. C00575: Cyclic AMP, C00788: adrenaline.


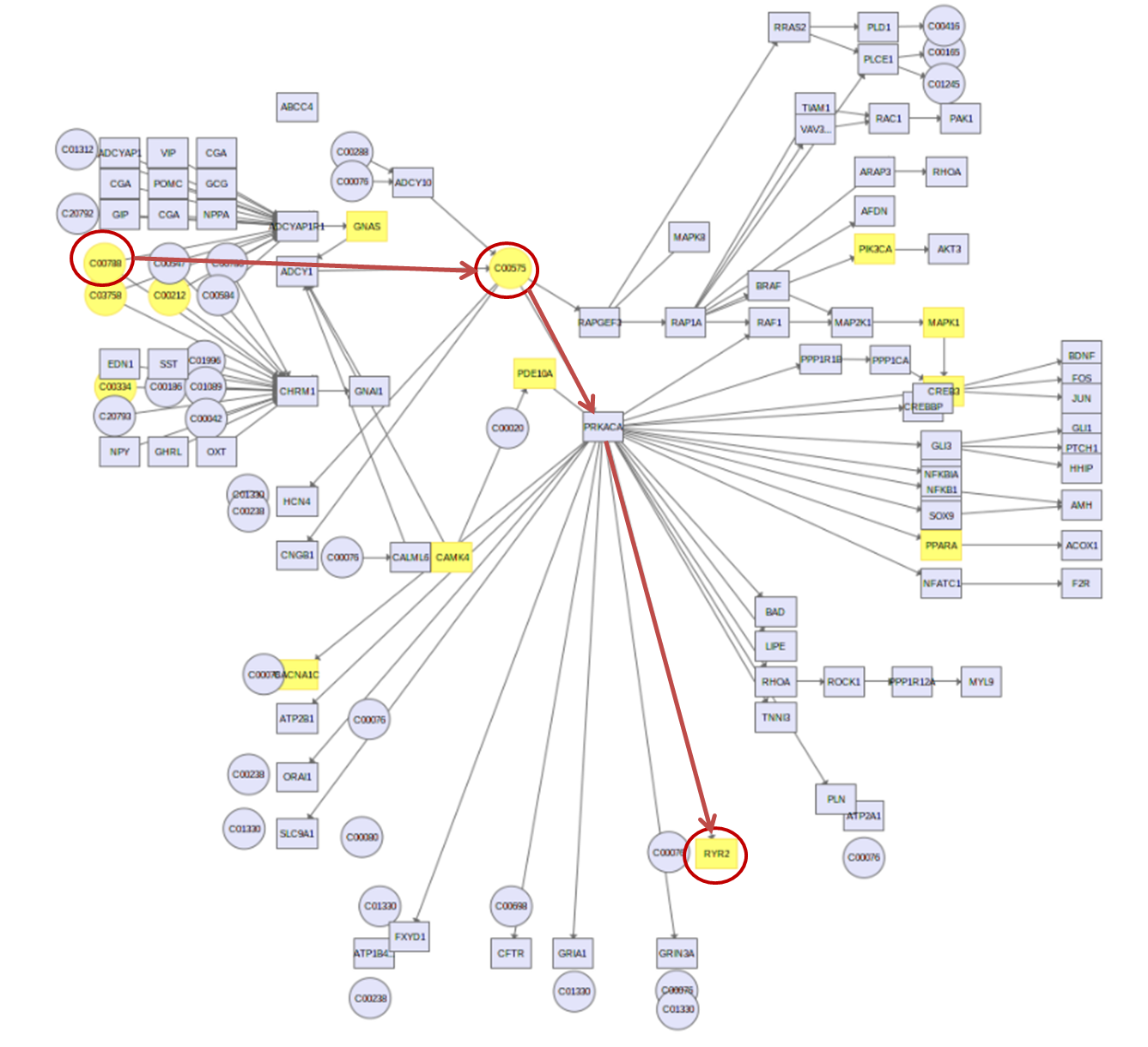


**Figure S4** cAMP signaling pathway. Highly enrichment modules were pointed in red. C00575: Cyclic AMP, C00788: adrenaline.
