# Supplementary material for: Fushenmu treatment ameliorates RyR2 with related metabolites in a zebrafish model of barium chloride induced arrhythmia
Source: Chin Med. 2023 Aug 19;18:103. doi: 10.1186/s13020-023-00812-x (PMC10439546; doi:10.1186/s13020-023-00812-x)
Supplement: Supplementary file 7 — Additional file 7: Table S22. Optimized MS parameters for the adrenaline and cAMP. Figure S5. The total ion chromatograms for adrenaline and cAMP. Table S23. Linear, repeatability, precision, and stability for adrenaline and cAMP. Table S24. Matrix effect for adrenaline and cAMP. Table S25. Simultaneous determination for adrenaline and cAMP in zebrafish embryos (n = 6, mean ± s.d.). Table S26. qRT-PCR analysis for RYR2 mRNA (n = 3, mean ± s.d.). [file 13020_2023_812_MOESM7_ESM.docx]

**Additional File 7 for FSM therapeutic actions on RyR2 and its related metabolites**

*1. Quantitative determination of FSM therapeutic markers*

*1.1 MRM parameters for* adrenaline and cAMP

**Table S22** Optimized MS parameters for the adrenaline and cAMP

| Compound | Precursor  Ion (m/z) | Product  Ion (m/z) | DP  (V) | CE  (V) | CXP  (V) |
| --- | --- | --- | --- | --- | --- |
| Adrenaline | 184.1 | 166.0 | 37.68 | 13.62 | 5.39 |
| cAMP | 328.1 | 134.1 | -90 | -30 | -30 |

DP: Declustering potential; CE: Collision energy; CXP: Export voltage


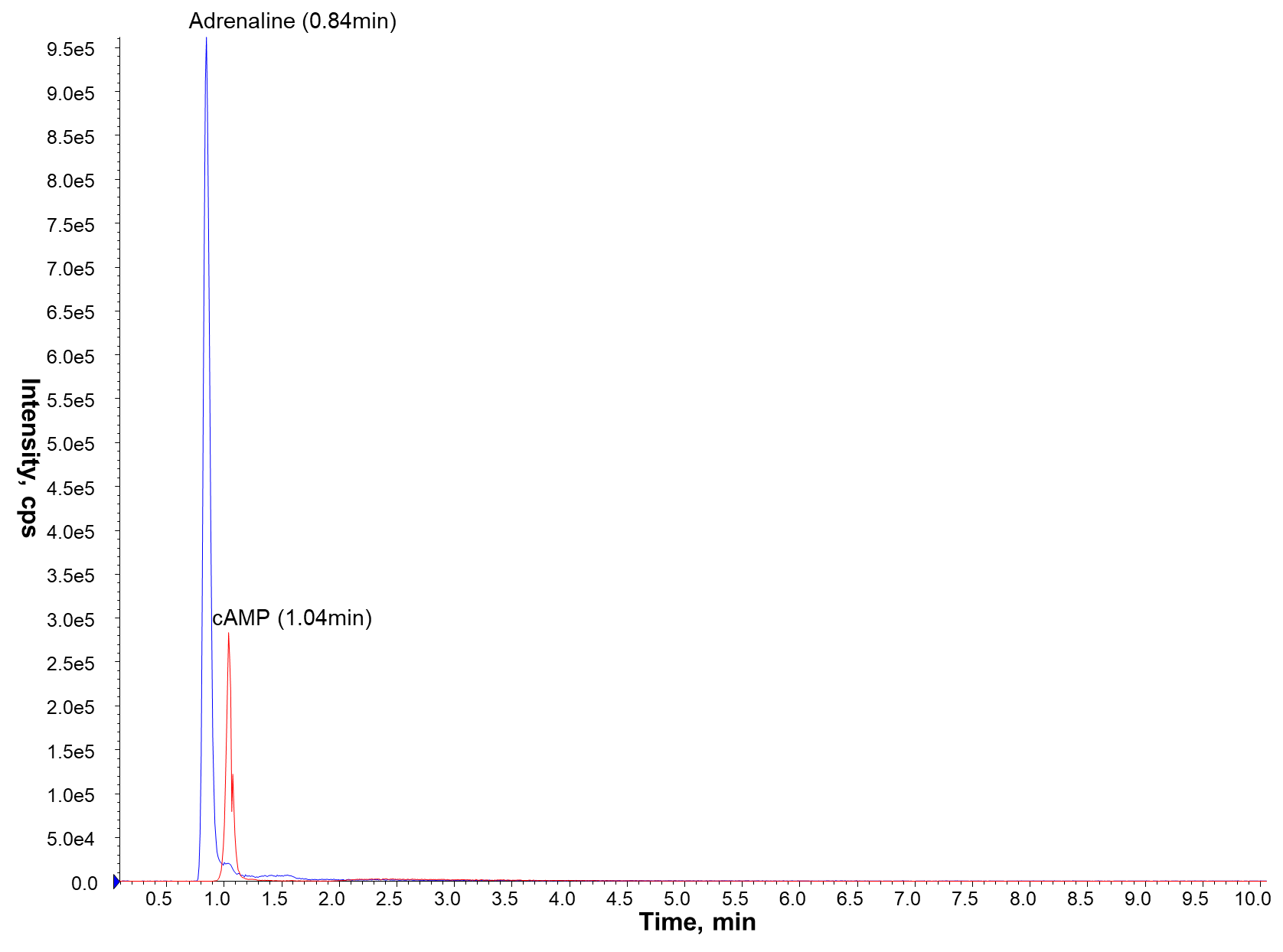


**Figure S5** The total ion chromatograms for adrenaline and cAMP.

*1.2 Simultaneous quantification methodologies for adrenaline and cAMP*

*1.2.1 Linearity and LOQs*

Adrenaline had a good linear relationship between 0.15~1.5 μg/ml, the linear equation is: Y ＝32785X +11851 (r =0.9978), cAMP linear relationship is good in the range of 0.001~0.1 μg/mL, the linear equation is: Y ＝4E+06x + 809.49 (r=0.9997), where Y represents the analyte peak area and X is the concentration of each calibration standard via linear regression analysis. The limit of quantifications (LOQs, signal to noise > 10) of the adrenaline and cAMP were 3 ng/mL and 0.1 ng/ mL, respectively (Table S23).

*1.2.2 Repeatability*

Adrenaline and cAMP were dissolved in methanol, dilute, and the concentrations of them were 125 μg/mL and 12.5 μg/mL respectively in the mixed standard solution. 10 μL of the mixed standard solution was added to 140 μL biological samples. Taking 2 μL prepared solution inject into UPLC, record peak area and retention time, then calculate the relative standard deviation. The RSDs of peak area were 2.68 % and 4.02 %, the RSDs of retention time were 1.38% and 1.10% (Table 23).

*1.2.3 Precision*

The samples prepared according to "repeatability" were used to exam the accuracy, both the peak area value and retention time of adrenaline and cAMP were recorded for 6 times. The RSDs of peak area were 2.18 % and 4.86 %, the RSDs of retention time were 1.38% and 1.38% (Table 23).

*1.2.4 Stability*

To determine the stability of biological samples, a sample prepared according to "repeatability" is placed in an automatic sampler at a temperature of 4℃, record peak area and retention time at 0 h, 2 h, 8 h, 12 h, 24h, all examinations were performed in triplicate at each time point. The RSDs of peak area was 2.67 % and 2.18 %, the RSDs of retention time were 0.48% and 0.00% (Table 23).

**Table S23** Linear, repeatability, precision, and stability for adrenaline and cAMP.

| Compounds | linear curve(coefficent) | linear range (ug/ml) | LOQs (ng/ml) | Repeatability  (Rt RSD%) | Precision(Rt RSD%) | Stability(Rt RSD%) |
| --- | --- | --- | --- | --- | --- | --- |
| Adrenaline | y = 32785x + 11851 (r=0.9978) | 0.15-1.5 | 3 | 2.68%  (1.38%) | 2.18% (1.38%) | 2.67% (0.48%) |
| cAMP | y = 4000000x + 809.49 (r=0.9997) | 0.001-0.1 | 0.1 | 4.02% (1.10%) | 4.86% (1.38%) | 2.18% (0.00%) |

*1.2.5 Matrix effect*

The contents of adrenaline and cAMP in the biological samples were detected using the method introducted in "repeatability", the peak area was recorded as A2; adrenaline and cAMP standard solution which has a same concentration with the biological sample was prepared, and record the peak area as A1, and the matrix effect was calculated by A2/A1 × 100%. The matrix effect of adrenaline and cAMP were 96.03 % and 93.12, respectively (Table S24).

**Table S24** Matrix effect for adrenaline and cAMP

| Component Name | Area-A2 | mean area of A2 | Area-A1 | mean area of A1 | A2/A1(100%) |
| --- | --- | --- | --- | --- | --- |
| Adrenaline | 1764000.00 | 1750333.33 | 1839000.00 | 1822666.67 | 96.03 |
|  | 1760000.00 |  | 1787000.00 |  |  |
|  | 1727000.00 |  | 1842000.00 |  |  |
| cAMP | 29520000.00 | 32310000.00 | 34270000.00 | 34696666.67 | 93.12 |
|  | 33530000.00 |  | 34610000.00 |  |  |
|  | 33880000.00 |  | 35210000.00 |  |  |

*1.2.6 Simultaneous determination for adrenaline and cAMP*

**Table S25** Simultaneous determination for adrenaline and cAMP in zebrafish embryos (n=6, mean ± s.d.)

|  | Control | Model | Positive | FSM-H | FSM-M | FSM-L |
| --- | --- | --- | --- | --- | --- | --- |
| Adrenaline | 1.135±0.034 | 3.026±0.201^**^ | 1.302±0.066^NS, ##^ | 1.405±0.039^*,##^ | 1.437±0.025^**,##^ | 2.161±0.302^**,##^ |
| cAMP | 0.0072±0.0002 | 0.0136±0.0004^**^ | 0.0077±0.0002^NS,##^ | 0.0084±0.0002^**,##^ | 0.0091±0.0005^**,##^ | 0.0116±0.0007^**.##^ |

*p < 0.05, **p < 0.005 compared with control gruop; #p < 0.05, ##p < 0.005 compared with model group. NS, not significant.

*2. qRT-PCR analysis for FSM therapeutic key targets*

**Table S26** qRT-PCR analysis for RYR2 mRNA (n=3, mean ± s.d.)

| mRNA | Control | Model | Metoprolol | FSM-H | FSM-M | FSM-L |
| --- | --- | --- | --- | --- | --- | --- |
| RYR2 mRNA level (2^-△△CT^) | 0.281±0.015 | 1.261±0.031^**^ | 0.371±0.009^NS,##^ | 0.404±0.025^NS,##^ | 0.522±0.081^**,##^ | 0.547±0.033^**,##^ |
